# Looking at Cerebellar Malformations through Text-Mined Interactomes of Mice and Humans

**DOI:** 10.1371/journal.pcbi.1000559

**Published:** 2009-11-06

**Authors:** Ivan Iossifov, Raul Rodriguez-Esteban, Ilya Mayzus, Kathleen J. Millen, Andrey Rzhetsky

**Affiliations:** 1 Cold Spring Harbor Laboratory, Cold Spring Harbor, New York, United States of America; 2 Biotherapeutics and Integrative Biology, Boehringer Ingelheim, Ridgefield, Connecticut, United States of America; 3 Center for Computational Biology and Bioinformatics, Columbia University, New York, New York, United States of America; 4 Department of Human Genetics, University of Chicago, Chicago, Illinois, United States of America; 5 Department of Medicine, Institute for Genomics and Systems Biology, Computation Institute, University of Chicago, Chicago, Illinois, United States of America; Weizmann Institute of Science, Israel

## Abstract

We have generated and made publicly available two very large networks of molecular interactions: 49,493 mouse-specific and 52,518 human-specific interactions. These networks were generated through automated analysis of 368,331 full-text research articles and 8,039,972 article abstracts from the PubMed database, using the GeneWays system. Our networks cover a wide spectrum of molecular interactions, such as *bind*, *phosphorylate*, *glycosylate*, and *activate*; 207 of these interaction types occur more than 1,000 times in our unfiltered, multi-species data set. Because mouse and human genes are linked through an orthological relationship, human and mouse networks are amenable to straightforward, joint computational analysis. Using our newly generated networks and known associations between mouse genes and cerebellar malformation phenotypes, we predicted a number of new associations between genes and five cerebellar phenotypes (small cerebellum, absent cerebellum, cerebellar degeneration, abnormal foliation, and abnormal vermis). Using a battery of statistical tests, we showed that genes that are associated with cerebellar phenotypes tend to form compact network clusters. Further, we observed that cerebellar malformation phenotypes tend to be associated with highly connected genes. This tendency was stronger for developmental phenotypes and weaker for cerebellar degeneration.

## Author Summary

We described *and made publicly available* the largest existing set of text-mined statements; we also presented its application to an important biological problem. We have extracted and purified two large molecular networks, one for humans and one for mouse. We characterized the data sets, described the methods we used to generate them, and presented a novel biological application of the networks to study the etiology of five cerebellum phenotypes. We demonstrated quantitatively that the development-related malformations differ in their system-level properties from degeneration-related genes. We showed that there is a high degree of overlap among the genes implicated in the developmental malformations, that these genes have a strong tendency to be highly connected within the molecular network, and that they also tend to be clustered together, forming a compact molecular network neighborhood. In contrast, the genes involved in malformations due to degeneration do not have a high degree of connectivity, are not strongly clustered in the network, and do not overlap significantly with the development related genes. In addition, taking into account the above-mentioned system-level properties and the gene-specific network interactions, we made highly confident predictions about novel genes that are likely also involved in the etiology of the analyzed phenotypes.

## Introduction

A quarter of century ago a (former) Hewlett-Packard executive famously complained: “If only HP knew what HP knows” [1]. This inability to access invaluable “collective wisdom” is by no means specific to a single community. It is felt acutely in every present-day endeavor involving multi-human exploration of complex phenomena. The problem is especially dramatic in the case of the explosively expanding molecular biology literature. There are thousands of existing biological periodicals and millions of potentially useful publications. New journals are emerging on a weekly basis and new articles accumulate as if deposited by an avalanche.

Understandably, no omniscient repository exists that lists *all* known (published) molecular events (such as protein–protein interactions) detected in human or murine cells. Although current text-mining tools are imperfect in their extraction accuracy and recall, they do help us to process huge amounts of unstructured text in nearly real time (which humans cannot do), moving us a bit closer to total awareness about the current state of knowledge [2].

Here we describe and make available two large new data sets derived through mining one-third of a million full-text research articles and a complete and up-to-date PubMed collection of journal abstracts. These data sets comprise mouse- and human-specific molecular interactions between genes and/or their products. We present here only the subset of text-mined interaction assertions that involve gene or protein names that we can link to unique identifiers in the standard sequence databases. This choice is determined by the goal of making our data immediately useful for applications that would have difficulty handling ambiguity in gene identity. The complete data are available through the Columbia University (http://wiki.c2b2.columbia.edu/workbench) and the University of Chicago (http://anya.igsb.anl.gov/genewaysApp).

We use our newly generated data to analyze genetic variation related to abnormal cerebellum phenotypes in mouse and human. Our analysis results in a compact set of statistically significant predictions that can be tested experimentally.

## Results/Discussion

### Gene-centric networks

Text mining with the GeneWays system [3],[4] allows us to capture multiple classes of relationships among biological entities, such as “A phosphorylates B,” “C activates D,” and “E is a part of F.” Table S1 displays the full list of relations that we can extract currently. The system also can recognize multiple classes of biological entities (terms) mentioned in the text: genes, proteins, mRNAs, small molecules, processes (such as *cell death* and *proliferation*), tissues, cell types, and phenotypes (such as *diabetes* and *hypertension*). While one can immediately think of a wide spectrum of applications where the full diversity of entities must be used, most of the current experimental methods are either gene-centric or genetic loci-centric (e.g., gene expression arrays, ChIP-on-chip, yeast two-hybrid, and genetic linkage or association data).

For this reason, the molecular networks we present here are gene-centric. This means that a given node in the network represents the union of the gene and its products (mRNA(s) and protein(s), if any); we exclude all other types of nodes (such as small molecules and phenotypes). Our practice of collapsing multiple nodes to a single node (gene plus mRNA plus protein) does not lead to a loss of information, because most of the physical interactions are defined for specific types of molecules. For example, in our restricted network relationship, “phosphorylate” can link only a pair of proteins, one acting as a protein kinase and another as the kinase's substrate, but not a gene and an mRNA. Furthermore, each original sentence used to extract the relation is preserved in the data set, along with the extracted fact and the reference to the appropriate paper, so that additional disambiguation can be conducted later, if required. We refer to each pair of extracted relationships and the original snippet of text as an *action mention*, as opposed to *action*, which is a relation disconnected from the source text and potentially mapped to multiple distinct action mentions.

A single pair of nodes in our text-mined network can be connected with multiple edges. These edges (interactions) can be undirected (we treat “A binds B” and “B binds A” as identical) or directed (“C activates D” is not the same as “D activates C”). We also subdivide edge types into two groups: *logical* and *physical*. Logical interactions include a family of regulatory relations that can be either direct (physical contact between two molecules) or indirect (mediated by one or more other molecules), such as *activate*, *inhibit*, and *regulate* (see Table S1). Physical interactions are by definition direct, such as *methylate*, *bind*, *glycosylate*, and *cleave* (see Table S1). The distinction between physical and logical interactions is important in understanding the data sets that we describe here. GeneWays ontology [5] includes a number of relationships between molecules that are neither physical nor logical interactions (for example, A *is an ortholog of* B, or C *is part of* D). We call this class of relations *other*.

### Reducing the noise level

In typical free text, gene names are dissociated from any references to gene-annotation databases. Furthermore, the “raw” text-mined molecular-interaction data are vast (GeneWays 7.0 comprises more than 8 million action mentions) but rather noisy: the error rate is close to 35% [6]. To get to smaller, cleaner, species-specific networks, we performed the following steps.

First, out of the complete network we retained only those gene names that can be linked to either human or mouse sequence database entries (normalization step) (see “Mapping names to genes” in the Text S1). Second, we filtered out relationships that are not molecular interactions and collapsed multiple edges between two nodes into a single edge. Third, we weeded out “raw” text-mined statements that did not meet our precision threshold (precision is defined as the proportion of correctly extracted statements among all those automatically extracted by a system). The third step was conducted automatically, using our automated curator engine [6], which has near-human curation precision (see the “Quality-of-extraction assessment” section in the Text S1.).

The first step resulted in the H70 and M70 networks (human- and mouse-specific GeneWays 7.0), in which nodes can be connected by multiple directed or undirected edges. The second step led to generation of the H70-PL and M70-PL networks (PL stands for *physical* and *logical*), where direction of edges was abandoned. The third step, assigned a precision threshold of 0.9 (90% of action mentions are correct), produced even smaller data sets, H70-PL0.9 and M70-PL0.9. Table 1 provides an overview of these networks at different levels of granularity. All intermediate data sets in this pipeline of data filtering are available for third-party computational analyses (see Datasets S2 to S5)

**Table 1:** Molecular networks and their properties.

Network tested in our analysis	How each network was generated	Ordered relations between genes	Node type	Node count	Interaction count	Action mention count (instances of relations mentioned in text)
GW70	Text-mining	Y	Name	1,759,377	5,934,024	8,424,449
H70	Filtering of GW70: all human-specific relations	Y	Gene	9,501	223,425	431,326
H70-PL	Filtering of GW70: all physical and logical human-specific interactions	N	Gene	8,186	63,449 **L**: 42,791 **P**: 8,934 **L**,**P**: 11,724	306,531
H70-PL0.9	Filtering of GW70: physical and logical human-specific interactions, 90% precision	N	Gene	7,793	52,518 **L**: 35,811 **P**: 7,385 **L**,**P**: 9,322	261,733
H70-P0.9	Filtering of GW70: physical human-specific interactions, 90% precision	N	Gene	5,453	16,707	61,826
M70	Filtering of GW70: all mouse-specific relations	Y	Gene	8,049	250,774	492,122
M70-PL	Filtering of GW70: all physical and logical mouse-specific interactions	N	Gene	7,975	70,445 **L**: 47,723 **P**: 9,586 **L**,**P**: 13,136	357,958
M70-PL0.9	Filtering of GW70: physical and logical mouse-specific interactions, 90% precision	N	Gene	7,600	57,786 **L**: 39,534 **P**: 7,860 **L**,**P**: 10,392	305,446
M70-P0.9	Filtering of GW70: physical mouse-specific interactions, 90% precision	N	Gene	5,356	18,252	69,360
HRPD	Manual curation of literature [7]	N	Gene	9,460	37,081	∼45,000

In addition, we produced networks with non-redundant edges and solely physical interactions, H70-P0.9 and M70-P0.9. As in the previous data sets, to filter these networks we used a precision threshold of 0.9.

### Evaluating the precision of the data

To evaluate the quality of the H70-PL0.9 network, we chose two random sets of logical and physical action mentions, a hundred mentions each, and asked an expert to evaluate their correctness. The expert commented on two steps of the process: whether the action mention is correctly extracted by the GeneWays system and, if the answer was “yes,” whether the corresponding gene names were correctly mapped to sequence identifiers. This allowed us to measure the absolute precision of the H70-PL0.9 network, the precision of term mapping, and the overall precision over the information extraction and term mapping stages.

The physical action mentions set indicated a precision of 0.8, with a confidence interval (CI) of [0.71, 0.87]. (We use CI at the 95% level of significance consistently throughout this paper.) The logical action mentions set showed a higher precision of 0.91, CI: [0.84, 0.95]. Because in our data set the number of logical interactions exceeds the number of physical interactions by more than two-to-one (2.49∶1), the overall precision of the HL70-PL0.9 data set is close to the target value of 0.9 (0.88). Term-to-sequence mapping precision was 0.89 (CI: [0.84, 0.93]) and 0.87 (CI: [0.81, 0.91]) for physical and logical action mentions, respectively (see Table 2).

**Table 2:** Evaluation of the precision of the H70-PL0.9 dataset.

Evaluation Set	Information extraction precision	Gene name mapping precision	Overall precision
**Physical** 100 action mentions from H70-PL0.9	*0.80 (0.71, 0.87)* 80/100 correctly extracted	*0.89 (0.84, 0.93)* 143/160 terms correctly mapped	*0.66 (0.56, 0.74)* 66/100 correctly extracted and mapped
**Logical** 100 action mentions from H70-PL0.9	*0.91 (0.84, 0.95)* 91/100 correctly extracted	*0.87 (0.81, 0.91)* 158/182 terms correctly mapped	*0.69 (0.59, 0.77)* 69/100 correctly extracted and mapped

100 physical and 100 logical action mentions were evaluated. The two steps of processing—GeneWays system extraction and the mapping of gene names—were evaluated separately in addition to the evaluation of the overall process.

Despite the favorable precision of the GeneWays extraction and the per-term mapping, the precision over both steps is less impressive: 0.66 (CI: [0.56, 0.74]) and 0.69 (CI: [0.59, 0.77]) for the physical and logical datasets, respectively. The reason for the lower overall result is the multiplicative calculus of the probability of not making an error: The overall precision of a term-mapped logical action is a product of the information extraction precision and the precision of two independent term mappings: 0.91×0.87×0.87 = 0.69.

Thus far we have evaluated the quality of extraction and mapping of *action mentions*. Recall that the same relation (action) between a pair of genes can be independently extracted from multiple sentences, generating distinct action mentions. Intuitively, the precision of an action (because an action is correctly extracted if at least one of its associated action mentions is correctly extracted) should be at least as high (or higher) than precision of the corresponding action mentions. To evaluate this precision, we sampled a hundred random *actions* from the H70-PL0.9 dataset, asked an expert to evaluate them at the levels of extraction and term mapping, and obtained an estimate of action-level two-stage precision of 0.74, CI: [0.65, 0.82]. This estimate is higher than the estimate of two-stage action mention precision (0.66 or 0.69). We believe that the action-level precision is more relevant to real-life applications in which scientists tend to care primarily about the precision of actions (statements distilled from multiple sources) rather than about their individual instances linked to text.

Note that the precision discussed in this section reflects only properties of our information extraction system and not the verity of published data.

### Comparison with HPRD

Several publicly accessible databases generated by manual analysis of research literature are available, including the Human Protein Reference Database (HPRD) [7],[8], Reactome [9], the Biomolecular Interaction Network Database [10], and the Database of Interacting Proteins [11]. These four data sets, along with a few others, were carefully compared in a recent study [12]. HPRD is by far the largest of the four.

As another quality control measure for our study, we compared our data with HPRD 7. The HPRD 7 network [7],[8] comprises 9,460 nodes (unique gene identifiers) and 37,081 edges, compared to 7,793 nodes and 52,518 edges in the H70-PL0.9 network. The H70-P0.9 network comprises 5,453 nodes and 16,707 edges; the node-wise and the edge-wise overlaps of H70-P0.9 with the HPRD networks are 4,543 and 4,877, respectively.

The HPRD and H70-PL0.9 networks share 5,945 unique gene-specific nodes. Out of the possible maximum of 17,668,540 interaction pairs between these nodes, the HPRD network has 23,662 and the H70-PL0.9 covers 43,496. We would expect a random overlap of about 58 interactions, while in reality we observe 7,577. The expected and the observed values are so far apart that the *p*-value (obtained with a hyper-geometric overlap test) is effectively zero—that is, the apparent overlap between the two sets of data is extremely non-random.

### Gold standard evaluation

Because human-curated databases may still harbor errors [13], we also compared our literature-mined dataset to a small set of high quality interactions produced by careful manual verification of a set of interactions shared by several human-curated databases [13].

In a recent study, Cusick *et al.* sought to evaluate the ultimate (truth) quality of the molecular interaction datasets generated via manual curation of the literature [13]. The authors selected two sets of curated interactions: one consisted of interactions that were curated in multiple databases and that were supported by multiple manuscripts and the other consisted of interactions supported by a single publication. They then carefully recurated the selected interactions and were able to estimate the corresponding error rates. As a byproduct of the evaluation, the authors produced two relatively small datasets, LC-multiple and LC-single, with 110 and 92 interacting pairs respectively, of exceptionally high-quality curated interactions. The LC-multiple set contained the interactions that were supported by multiple manuscripts even after the recuration and the LC-single set contained the interactions with one supporting manuscript that was confirmed during the recuration. The LC-multiple set subsequently was used as a gold standard for the evaluation of high-throughput yeast two-hybrid assays in a second manuscript by the same group, in which an additional random set of 188 supposedly non-interacting pairs (the Negative set) was selected [14].

We used the LC-multiple, LC-single, and Negative sets as comparison standards for our own literature-mined networks (see Table 3). It is reassuring that our H70-PL network covers nearly 70% (75 out of 110 pairs) and that our most filtered human network, H70-P0.9, covers more than 55% of the well-supported interactions in the LC-multiple set. The more obscure interactions from the LC-single set are not covered as well (i.e., H70-P0.9 contains about 20% of the LC-single set). However, given that we have processed only a small portion of all of the scientific literature with a system that highly favors precision over recall, being able to recover 20% of the interactions supported by a single article is surprisingly high. Finally, our networks do not contain any of the interactions listed in the Negative set. For comparison, the last two lines in Table 3 give the results for the two high-throughput assays MAPPIT and Y2H-CCSB evaluated in Figure 2 of [14].

**Table 3:** Overlap with the comparison standards.

	Test Set		
Network	LC-multiple (110 pairs)	LC-single (92 pairs)	Negative (188 pairs)
**H70-PL**	75	28	0
**H70-PL0.9**	73	27	0
**H70-P0.9**	61	19	0
**MAPPIT**	19	N/A	2
**Y2H-CCSB**	15	N/A	0

### Precision versus recall

The performance of text-mining methods is commonly evaluated using two metrics: precision and recall. For information-extraction systems, *precision* is defined as the proportion of correctly extracted statements among all those automatically extracted by a system. The *recall* is the ratio between the number of statements correctly extracted by the system and the total number of statements that *can* be extracted from the original text by a hypothetically perfect system. In a less than perfect system, recall and precision are antagonistic: one is increased at the expense of the other.

In this study we favored precision at the expense of recall: We explicitly used a statement precision threshold as a filtering criterion. We also excluded actions with ambiguous gene names and disqualified some 10^5^ potentially useful instances of text-mined intramolecular relations that fit neither physical nor logical categories (such as *contain* and *is a homolog of*), thus worsening recall and improving precision. In addition, we used only those actions that involve either genes or their products (and no other entity classes).

**Figure 1:**
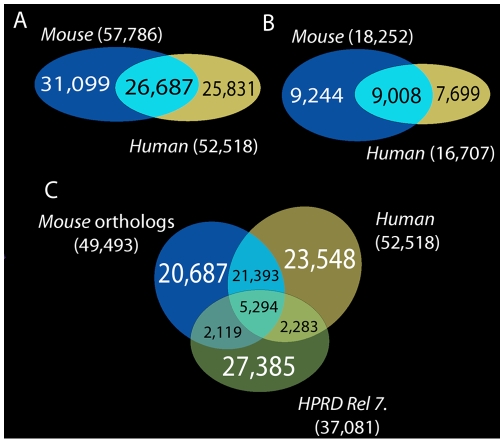
Network interaction-overlaps. A. Overlap between the human and mouse PL (physical and logical) networks. B. Overlap between the human and mouse P (physical) networks. Interactions in A and B are compared through gene orthology. C. Composition of the *union* network (GeneWays human (H70-PL0.9), HPRD network, and GeneWays mouse (M70-PL0.9) orthology).

While our human-specific network, which unifies physical and logical interactions (H70-PL0.9), is larger than HPRD 7, the relationship is reversed for the physical-interaction (H70-P0.9) data set and HPRD 7. This is because we filtered out from our data numerous physical action mentions that did not pass our precision threshold. (Note that HPRD 7 incorporates high-throughput interaction data that is probably distinct from the small-scale experimental data published in research papers, in terms of error patterns.) Nevertheless, the HPRD 7 data sets and our data sets are very different. The joint interaction coverage of HPRD 7, H70-P0.9, and M70-P0.9 ortholog data sets is more than twice as large as the coverage of HPRD 7 alone (Figure 1); this is enough to merit the use of a union of these networks in biological applications.

Because we are making the “raw” (unfiltered) statements publicly available, anyone interested in using our data can apply his/her own custom-made filtering process to achieve the desired balance between recall and precision in the output.

### Human and mouse networks

Two genes residing in genomes of distinct species can either share a common origin (homology) or be unrelated. Homologs come in at least in two flavors [15]: *orthologs* and *paralogs*. Two genes in, say, human and mouse, are orthologs if they were separated by a speciation event. If, in addition to speciation, an intragenomic gene duplication occurred, separating two genes from a common ancestral gene, they are paralogs. For example, human and mouse embryonic *β*-globins are orthologs, but mouse *α*-globin is a paralog of human *β*-globin.

Physical interactions between molecules are not immutable over long evolutionary intervals [16]. Nevertheless, an interaction between two proteins discovered in one species has a reasonable chance of existing between orthologs of these proteins in another species if the two species are closely related. Therefore, if we know of interacting molecules in one species and can identify orthologous molecules in another species, we can formulate hypotheses about the existence of orthologous interactions in the latter species. All such computationally formed hypotheses are subject to experimental validation.

Mouse and human genomes are separated by more than 100 million years of independent evolution [17], but mouse genetics and molecular biology are commonly used to understand human phenotypes in health and disease. Therefore, we decided to compile a molecular-interaction network summarizing the wealth of knowledge for humans and mice. We used orthology-mapping of human and mouse genes to connect the two networks. (Reactome's developers [9] used a similar strategy with their manual compilation of data.) Such a network could potentially have a multitude of practical applications.

We assembled our network by combining mouse- and human-specific networks extracted from the biomedical literature using text-mining tools. We used human-to-mouse gene orthology mapping provided by the Mouse Genome Database [18],[19]. Some of the mouse interactions could not be mapped to corresponding human interactions because at least one of the involved genes lacked known human orthologs. We transferred by mouse-to-human orthology-mapping 49,493 and 16,317 interactions for physical-logical and physical networks, respectively. These orthology-mapped interactions are subsets of the 57,786 and 18,252 interactions in the physical-logical and physical networks, respectively. Although a large number of interactions occur both in humans and mice individually (see Figure 1), the double-confirmed overlap constitutes only about a third of all interactions in the union network (see Figure 1 A and B). Figure 1 C shows a three-way Venn diagram for our text-mined interactomes (human and mouse) and the HPRD dataset. Clearly, all three networks contain a substantial number of unique interactions that merit their joint consideration in biological applications (see Dataset S6).

To illustrate an application of our data to the analysis of mammalian phenotypes, we performed mapping of mouse cerebellar phenotypes (related to ataxia) to the three-data set network.

### Mapping ataxia phenotypes to the mouse-human network

The word *ataxia* (αταξια—“lack of order”), in its English usage, refers to a lack of muscular coordination in an animal body. Humans with ataxia often have difficulty walking, talking, maintaining posture and balance, controlling eye movements, holding and manipulating objects, gesturing, and even swallowing food. In a mammalian brain, the cerebellum is predominantly responsible for spatial and temporal coordination of complex neuromuscular processes. Cerebellar function is also essential for cognition sensory discrimination [20]. Most cases of ataxia are associated with either environmental or genetic damage to this brain region. The typical environmental triggers of ataxia include head trauma, viral infections, and exposure to recreational or medicinal poisons, such as alcohol, lithium carbonate, tranquilizers, antipsychotics, and the anticonvulsant carbamazepine. Genetic factors include a diverse spectrum of genomic aberrations that cause abnormal development and/or premature degeneration of the cerebellum. Ataxia can be severely debilitating and, unfortunately, the phenotype is reversible in only a minority of cases (such as those caused by short-term alcohol intake).

**Figure 2:**
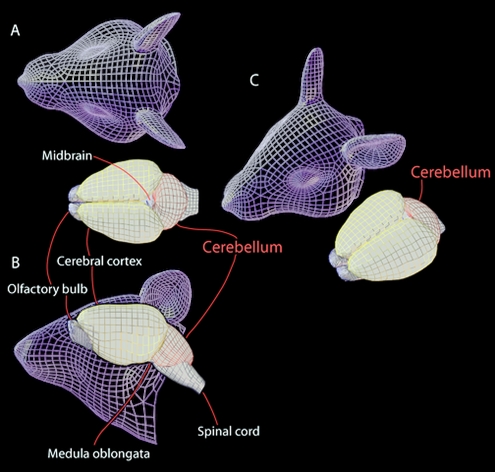
Morphology of mouse brain: olfactory bulbs, cerebral cortex, midbrain, cerebellum, and spinal cord are labeled. A. Top view: a mouse brain is shown next to an outline of a mouse head. B. Side view of a mouse brain superimposed with a mouse head. C. Perspective view of a mouse brain and head.

**Figure 3:**
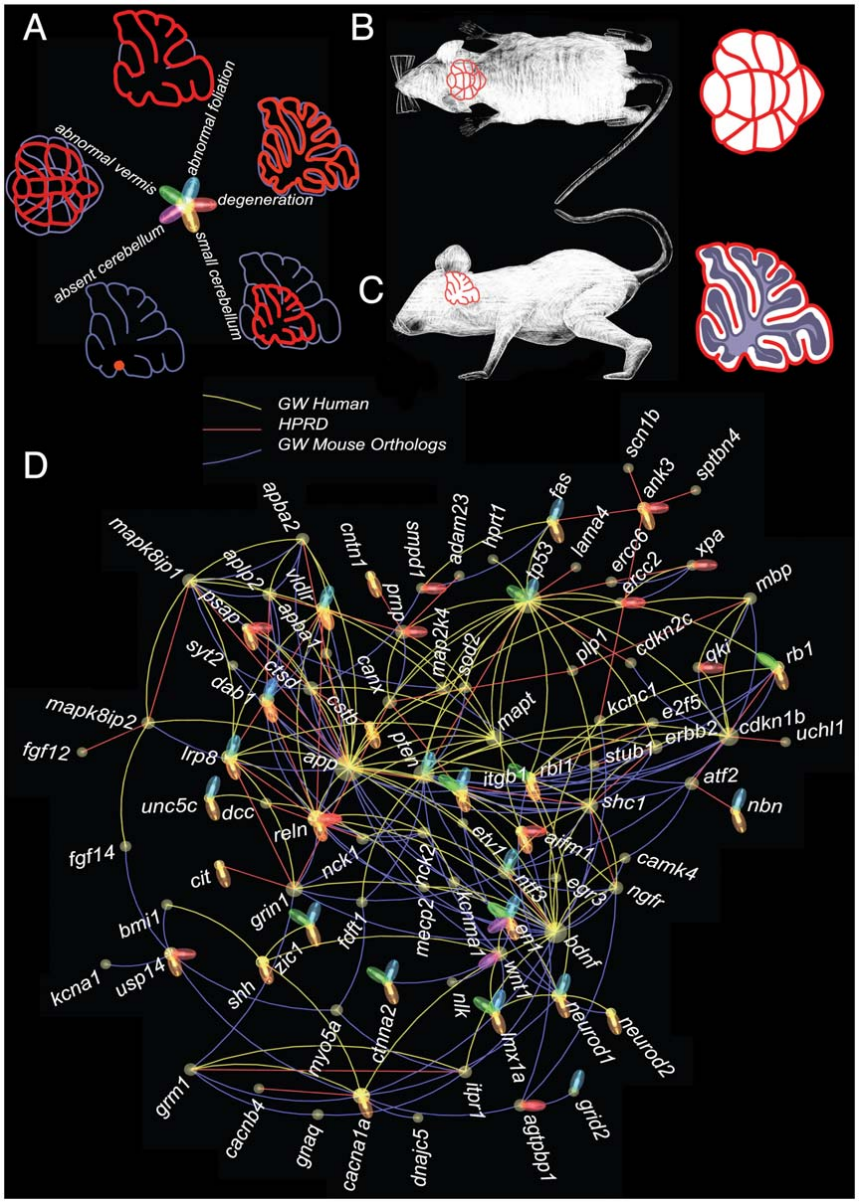
Molecular-interaction network integrating genes related to the ataxia phenotype. The network was identified by selecting the human orthologs of the mouse genes associated with ataxia in the MGI database. Interactions from the three different sources are indicated with different edge colors. The flower-like design of nodes indicates the specific subset of cerebellar phenotypes associated with each gene. A. Schematic representation of the abnormal cerebellum phenotypes and assignment of petal phenotypes. The thin gray line represents what a normal cerebellum should look like and the red line shows the observed cerebellum. B and C. Schematic representation of a normal cerebellum in relation to whole mouse body in top view B and in side view C. D. The largest connected component of the ataxia sub-network. The abnormal cerebellum phenotype assignments are shown with the flower petals, the size of a node represents the number of interactions it is involved in, and the color of the edges represents the source network as defined in the legend.

Mouse and human geneticists who study brain phenotypes typically group developmental malformations by the anatomical structures that are affected. As brain topology in three-dimensional space does not lend itself readily to verbal description, we provide three projections of a typical mouse brain in Figure 2 (see also the interactive model in Figure S1). Moving front-to-back in the external view of the mouse brain, there are two olfactory bulbs followed by hemispheres of cerebral cortex that are immediately adjacent to the cerebellum and brainstem (see Figure 2 A–C). We focus here on the cerebellum (literally, “little brain”) that contains involuted cortex with narrow leaf-like gyri (“folia,” see Figures 3 A and C). Like the brain itself, the cerebellum has two hemispheres with a *worm*-like medial structure, the *vermis*, between them (Figure 3 A and B).

In both humans and mice, a collection of genetic aberrations exist that are known to predispose the bearer to specific cerebellar abnormalities. For computational implementation it is convenient to represent phenotypic variations of cerebellar structure with hierarchically ordered categories in a mammalian phenotype (MP) ontology [21]. We focused on five broad anatomical/cerebellar causes of ataxia which can be observed as structural abnormalities in brain imaging studies (such as MRI scans) or histological analysis. These phenotypes are represented with MP concepts: degeneration (MP:0000876), abnormal foliation (MP:0000857 and MP:0000853), abnormal vermis (MP:0000864), small cerebellum/cerebellar hypoplasia (MP:0000852 and MP:0000851), and absent cerebellum (MP:0000850).

Cerebellar *degeneration* is abnormal death of cerebellar neurons—the cerebellar folia become narrower over time and are separated by irregular and wider spaces compared with those in a healthy brain (see Figure 3 A). As with other major insults to the cerebellum, degeneration reveals itself in abnormalities in body balance, jerky movements of limbs, unsteady (wide-legged) gait, and irregularities of speech (slurred or slow) and eye movement (*nystagmus*, or rapid involuntary eye movements). Most defined degenerative ataxias affect the fully mature cerebellum, but a small subset of degenerative ataxias have a developmental onset [22].

*Abnormal foliation* typically involves the absence of some of the cerebellar folia and irregular shape of those that are present. In normal individuals, cerebellar foliation is stereotypical, with the basic folial pattern conserved between mice and humans. Disruption of foliation disrupts the topographical map of incoming and outgoing neuronal connections [23].

An *abnormal vermis* is typically reduced (compressed and distorted) compared with a normal one, or it can even be completely missing (see Figure 3 A). Clinical outcome is variable [24]. Dandy-Walker malformation is one of the well-known birth defects in humans and mice that are defined by an abnormal vermis. In addition to an aberrant vermis, Dandy-Walker malformation frequently involves enlargement of the fourth brain ventricle and an increase in fluid-filled space around the brain [25]. It is not uncommon in clinical reports to find an abnormal vermis coupled with other cerebellar malformations [26],[27].

*Small cerebellum*, or cerebellar hypoplasia, refers to phenotypes in which the cerebellum, while present, does not develop to normal size (see Figure 3 A). In humans, cerebellar hypoplasia can lead to delayed or undeveloped speech, difficulties with walking and maintaining balance, mental retardation, floppy muscle tone, nystagmus, and seizures. In its worst forms, cerebellar hypoplasia can be completely debilitating and even lethal [28].

*Absent cerebellum* is infrequent in adult humans and mice, possibly because in most cases it causes early death. Rarely, individuals are only mildly affected. For example, a documented brain autopsy of a 38-year-old individual who accidentally electrocuted himself revealed a virtually absent cerebellum [29]. The individual was apparently functional and capable of conducting all common human activities, including gesturing, talking, performing complex manual work, and participating in social interactions. Some have proposed that an absent cerebellum is therefore less disabling than a present, but abnormal cerebellum [30].

Fortunately, the Mouse Genome Database (MGD, [18]) uses the MP ontology to link genetic variation in mouse genes to phenotypic aberrations that are causally related to known genomic changes. We were able to use the MGD to associate 244 human genes (with the help of the human and mouse orthology) with the five ataxia phenotypes described above and with ataxia (MP:0001393) (see Table S7). By integrating mouse (M70-PL0.9), human (H70-PL0.9), and HPRD (Release 7) networks through human–mouse gene orthology mapping, we obtained a larger network of interacting human genes with annotation of ataxia phenotypes generated in mouse studies. The largest connected component of the ataxia graph includes 88 human genes. These 88 genes are connected with 145, 147, and 72 interactions derived from human GeneWays, mouse GeneWays, and HPRD, respectively (see Figure 3 D).

Our analysis of ataxia-related phenotypes in the context of a molecular network generated rather curious and statistically significant results, as described in the following section.

### Observations and predictions derived from analysis of ataxia phenotypes in the context of a molecular-interaction network

#### Same-gene co-association of phenotypes

First, we looked at how often genetic aberrations causing distinct cerebellar phenotypes occur within the same gene (see Tables S2 and S3). Within the five phenotypes considered here, four appear to be associated with errors in development of the organ (abnormalities of foliation and vermis, and small or absent cerebellum), while the fifth phenotype is related to degeneration of the already-developed organ.

Genetic variation causing cerebellar degeneration does not seem to occur at more than random frequency in the same gene as variation causing the “developmental” phenotypes. Put differently, cerebellar degeneration appears to be statistically independent of the other cerebellar malformation phenotypes in terms of genetic variation, given our current sample size. In contrast, the four developmental phenotypes are highly significantly correlated pair-wise in terms of their gene associations (see Tables S2 and S3). This indicates that the developmental phenotypes are genetically entangled, in that they are associated with highly overlapping sets of genes. It is likely that pleiotropic genetic variation exists that can cause more than one of the four phenotypes simultaneously. Although cell death is a normal part of development, the lack of overlap between these two phenotypic classes is not surprising given our current understanding of these disorders. Many genes causing cerebellar degeneration when mutated are involved in neuronal metabolism and are widely expressed throughout the brain. The high metabolic rate of the large cerebellar Purkinje neurons may predispose these specific neurons to lethal metabolic stress when they are not functioning normally [31]. In contrast, developmental cerebellar genes are enriched for cell signaling and signal transduction molecules.

#### Network clustering of phenotype-specific genes

We next asked if phenotype-specific genetic variation in our data set maps to non-randomly clustered groups of genes within our text-mined molecular network. To test the significance of gene clustering, we used three different approaches: a parametric test of clustering (see Text S1) and two non-parametric approaches. Both non-parametric tests are based on estimation of a background distribution of a test statistic (under a no-clustering scenario) by stochastic permutation of the original network. We used the total number of interactions among phenotype-specific genes as test statistics for both non-parametric tests.

The simpler non-parametric test (Ini) involved stochastic sampling of 1,000 gene sets of the same cardinality as the original set of phenotype-associated genes. The slightly more sophisticated non-parametric test (Rwr) generated 1,000 randomly re-wired molecular networks with both node identities (phenotype mapping) and node connectivity values preserved exactly as in the original network. We computed empirical *p*-values for both tests by comparing the real value of our statistic against the appropriate background distribution.

The three tests are based on different sets of assumptions about the background model. For example, non-parametric tests attempt to emulate the empirical connectivity distributions of genes, whereas the parametric test does not.

We applied the three tests to the five phenotypes chosen for this study and a set of molecular networks: the union of human–mouse GeneWays and HPRD (the *union network*), HPRD alone, and subset of the union network including only physical interactions (we assumed that all HPRD interactions are physical) (see Table S5). All tests indicated significant clustering of phenotype-specific genes within our networks; the re-wiring test generated the most conservative (high) *p*-values.

The HPRD network and the physical network allowed detection of highly significant gene clustering in all groups of phenotypes except for *degeneration* and *absent cerebellum*. The whole network showed highly significant gene clustering for all studied groups of phenotypes; significance was most impressive for the largest (union) network (see Table S5). It is reassuring that genes for the same phenotype are brought close together in our largest network and this can be used as an argument for the necessity of the extended network. This result might be due in part to a publication bias: Genes that were discovered earlier tend to accumulate a larger number of published logical interactions. However, when we repeated the analysis of the union network with logical interactions removed, the phenotype-specific clustering was still present and significant, albeit at a lower significance level (see Table S5). We observed higher clustering for genes specific to developmental phenotypes. Finally, the most conservative rewiring test failed to detect significant clustering of genes associated with *abnormal vermis* and *absent cerebellum* in physical-interaction and HPRD networks.

#### Cerebellar malformations tend to be caused by highly connected genes

While examining the lists of genes associated with cerebellar malformations, we suspected that the highly connected genes appear in these lists with greater than random frequency. A parametric test designed to test this hypothesis confirmed that this trend is highly significant for most phenotypes, with the exception of *cerebellar degeneration* and *absent cerebellum*, analyzed in the context of physical interactions (see Table S4 and Text S1 for a description of the test).

We came up with four possible explanations for this finding. First, the genes involved in embryogenesis often have high connectivity. Second, genetic variation harbored by highly connected genes has elevated the odds of affecting the morphology of the cerebellum. Third, our knowledge about molecular interactions is skewed by the history of the field—that is, genes that were studied earlier are more likely to accumulate a large number of known links (especially *logical* ones) compared to their peers that were examined much later [32]. By the same logic, well-studied genes are more likely to be tested for causal association with a phenotype than their more obscure counterparts. Another explanation is that more than one of the above factors contributed to the observed abundance of highly connected genes in our lists of genes relevant to cerebellar development.

While all of these hypotheses are testable, we have to wait for adequate data to be available to distinguish among them. We are well aware that the historical knowledge bias is real, especially for logical interactions of well studied and conserved genetic pathways. To test if our degree preference results are due solely to the literature bias, we ran the same test using the protein–protein network produced by fusing two large-scale yeast two-hybrid assays (Y2H with 2936 nodes and 5722 edges) [33],[34]. The strong significance for high-degree preference was diminished in the Y2H network, suggesting that the literature bias could be a factor, but did not disappear completely. (Empirical *p*-values calculated for the “all” and “abnormal vermis” gene sets within Y2H network were smaller that 0.05, leaving room for the first and second explanation.) Therefore, at the moment, we favor the fourth (composite cause) explanation.

#### Predicting new phenotype-specific genes

We used a molecular triangulation technique [35] to identify additional candidate genes relevant to each phenotype by studying clusters of phenotype-specific genes. The molecular triangulation technique is designed to analyze the output of a genetic linkage analysis: it detects non-random gene clustering within a molecular-interaction network. Triangulation uses a null model in which the linkage signals are uninformative (unlinked to the phenotype) and any observed gene clustering within a molecular network is accidental. The hypothesis competing with the null assumes that the linkage signal is associated with a group of genes forming a functional cluster within the molecular network. In this application we used as input to molecular triangulation analysis phenotype-specific causal genes (rather than genetic linkage signals). The triangulation analysis generated a surprisingly large number of statistically significant predictions (results are not shown).

**Figure 4:**
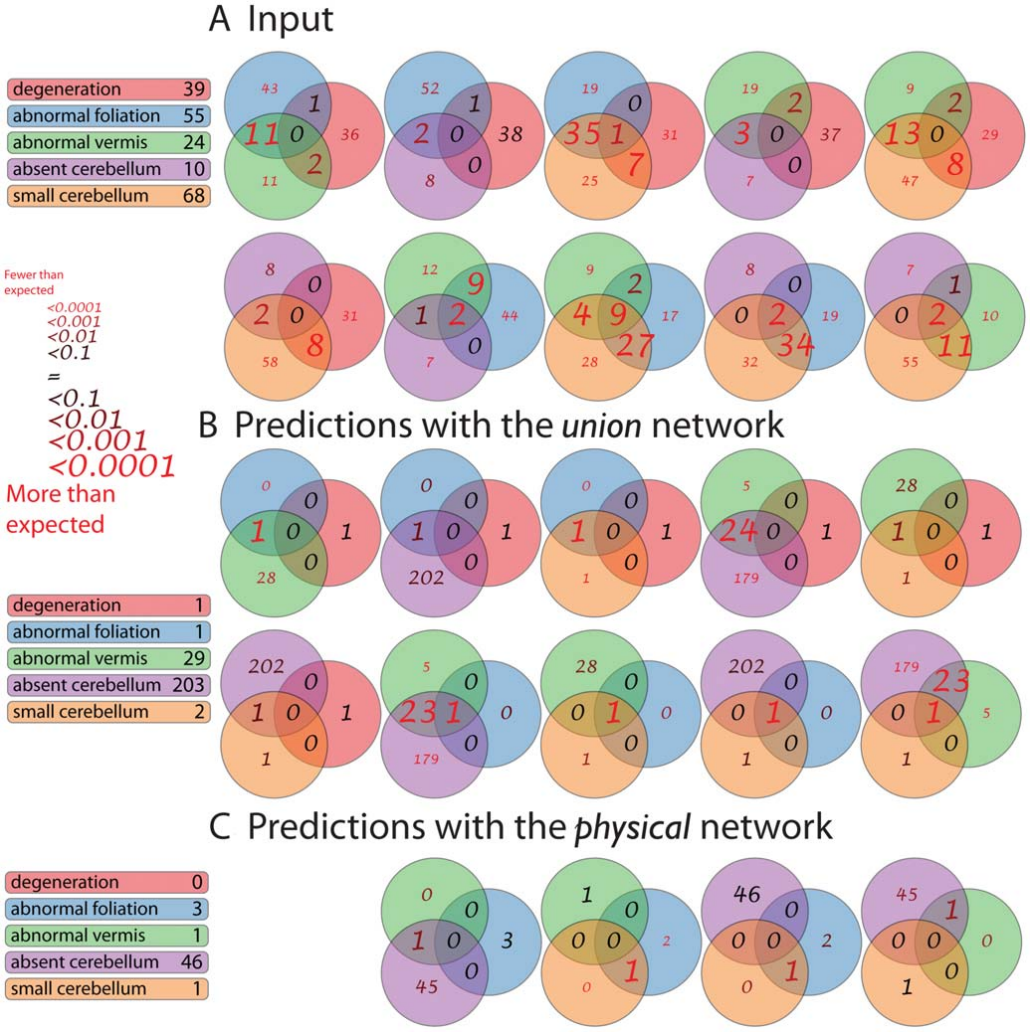
Overlap of genes associated with several cerebellar phenotypes. A. Venn diagrams for phenotype-specific gene sets retrieved from the Mouse Genome Database. B. Similar Venn diagram for newly predicted candidate genes for the same phenotypes generated through analysis of the *union* network (both logical and physical interactions). C. Analysis of the *union* network with only physical interactions retained.

We expect that a large number of genes—possibly thousands—contribute to shaping the architecture of an organ as complex as the human or mouse cerebellum. This is clearly supported by the large number of genes expressed in this region of the developing brain [36]. It is not productive, however, for an experimentalist to start with too many predictions. In addition, as long as we have firm evidence that highly connected nodes are over-represented among our phenotype-specific genes, some of the significant predictions may be artifacts of the excessive connectivity. For this reason we modified the triangulation technique (see Text S1) to take into account the apparent connectivities of genes within the molecular network in general and of potentially biased seed genes in particular. While retaining the major assumptions of the original molecular triangulation, this modified test involved node degree-preserving random rewiring of the network, not unlike our test applied to phenotype-specific gene clustering. The degree-preservation addition made the test much more conservative (see Tables 4 and S6 and Figure 4); instead of hundreds or thousands of predictions, only a few survived the significance threshold of a 0.5 level of false discovery rate (FDR) (see Tables 4 and S6 and Figure 4). (FDR is a computational technique used to adjust significance results of statistical testing for multiple tests [37],[38]) The 0.5 FDR threshold means that half our predictions are expected to be false positive. We also conducted a test with stricter FDR levels of 0.1 and 0.01 and indicated the genes that are significant at these levels with bold and underlined bolds gene symbols, respectively, in Tables 4 and S6. Further, the numerous genes that are significant at FDR level of 0.0001 based on our ini-trn analysis (see the Text S1) are listed in Table S9.

**Table 4:** A subset of the phenotype-specific gene predictions at 0.5 FDR level.

	Gene	Gene		*p*-value		
Phenotype	Id	symbol	Initial neighbors	whole	HPRD	physical
**degeneration**	412	sts		2×10^−5^		0.396
**abnormal foliation**	7471	**wnt1**	ccnd1, ccnd2, ctnna2, en1, gas1, lmx1a, pten	8×10^−7^	0.999	0.372
**abnormal vermis**	22943	**dkk1**	en1, fgfr1, msx2, tp53	5×10^−6^	0.962	0.983
	5076	**pax2**	en2, fgf8, pax5, rb1, tp53	8×10^−6^	0.041	0.012
	2253	fgf8^*^	en1, en2, fgf17, fgfr1, pax5, zic1	1×10^−5^	0.287	0.355
	4487	**msx1**	fgf8, msx2, tp53, zic1	1×10^−5^	0.003	0.020
	5077	pax3	fgf8, gli2, msx2, tp53, zic1	4×10^−5^	0.590	0.676
	8817	fgf18	en2, fgf8, fgfr1	6×10^−5^	0.115	0.194
	27330	rps6ka6	fgf8	7×10^−5^	0.332	0.294
	10253	spry2	fgf8, fgfr1	8×10^−5^	0.521	0.037
	81848	spry4	fgf8, fgfr1	9×10^−5^	0.391	0.026
	7471	wnt1	ctnna2, en1, fgf8, lmx1a	9×10^−5^	0.999	0.113
	655	bmp7	en1, fgf8, fgfr1, msx2, zic1	1×10^−6^	0.956	0.542
	268	amh	rbl1	0.0001	0.389	0.053
	1745	dlx1	fgf8	0.0002	0.143	0.035
	3223	hoxc6	fgf8, fgfr1	0.0002		
	5178	peg3	tp53	0.0003	0.582	0.380
	7476	wnt7a	en1, fgf8, lmx1a	0.0003	0.953	0.849
	2637	gbx2	fgf8, gli2	0.0004		
	2737	gli3	fgf8, fgfr1, gli2, zic1	0.0004	0.026	0.002
	4613	mycn	pax5, rb1, tp53	0.0005	0.578	0.032
	429	ascl1	fgf8	0.0005	0.197	0.151
	5727	ptch1	fgfr1, gli2	0.0005	0.600	0.533
	17	aavs1		0.0006		1×10^−4^
	3222	hoxc5	fgf8	0.0006		
	2535	fzd2		0.0006	0.486	0.133
	8646	chrd	en2, fgf8, fgfr1	0.0008	0.908	0.390
	54756	il17rd	fgf8, fgfr1	0.0009	0.044	0.046
	5081	pax7	fgf8, gli2	0.0010	0.593	0.340
	985	cdc2l2		0.0011		0.040
	6677	spam1	tp53	0.0012		
**small cerebellum**	1020	**cdk5**	ccnd1, ccnd2, cdk5r1, cdk5r2, dab1, dcx, erbb3, pura, rb1, reln	7×10^−6^	1×10^−5^	9×10^−7^
	7471	wnt1	ccnd1, ccnd2, ctnna2, en1, gas1, lmx1a, lmx1b, shh	2×10^−5^	0.999	0.794

The bold genes are significant at 0.1 FDR level and the bold and underlined genes are significant at 0.01 FDR level. For a complete list of our predictions see Table S6. The ^*^Fgf8 is among our initial genes for the abnormal vermis phenotype.

Our leave-one-out cross-validation experiments (see Text S1) demonstrated that the union network clearly outperformed the smaller networks in our comparison in predicting “missing” phenotype-associated gene sets.

As Figure 4 shows, absent and small cerebellum phenotypes were especially rich in gene candidate predictions, whereas cerebellar degeneration was the poorest. Following is an overview of a few selected genes that appeared in the prediction gene lists for both abnormal vermis and absent cerebellum phenotypes.

One of our top predictions, the *Ascl1* gene, is involved in neuronal commitment and differentiation [39]. Another gene, *Bmp7*, encodes a member of the bone morphogenic protein (BMP) family; genes of this family are implicated in a wide spectrum of developmental processes in vertebrates, including bone development [40],[41]. Both genes are expressed in the developing cerebellum (informatics.jax.org), but no cerebellar phenotypes have been described in available mouse mutants. Homozygous *Ascl1* mouse mutants die neonatally before extensive cerebellar development. Nevertheless, it has recently been reported that complete loss of *Ascl1* alters the development of cerebellar interneurons, oligodendrocytes and astrocytes during late embryogenesis [42] validating our prediction. There is extensive published evidence that *Bmp7* can influence multiple aspects of cerebellar development both in vitro and in vivo [43],[44], but again the cerebellum has not been the focus of published mouse mutant phenotypic analysis, hence Bmp7 is not yet associated with any MP category.

An exciting quadruplet of predictions (*Fgf8*, *Fgf18*, *Spry2*, and *Spry4*) is tightly linked to tissue differentiation pathways. *Spry2* and *Spry4* are inhibitors of fibroblast growth factor [45],[46] and of receptor-transduced mitogen-activated protein kinase (MAPK) signaling pathways [47]. Fibroblast growth factors (FGFs) and fibroblast growth factor receptors (FGFRs) participate in regulatory processes of tissue pattern formation and cell differentiation. They are implicated in the developmental regulation of all the major systems of the mammalian body, including the limbs, bones, and brain. There are multiple neurological phenotypes, including mood disorders and asocial behavior [48]–[50], linked to genetic variation harbored by genes representing these families. Fgfs have long been implicated in cerebellar development [23]. More recently, Basson et al. [51] have demonstrated that regulation of Fgf signaling by ectopic misexpression of *Spry2* has profound effects on cerebellar vermis development. The endogenous roles of these proteins in cerebellar development have not yet been the focus of any phenotypic investigation in the mouse. Our analysis here suggests a second look.

We also found a triplet of PAX genes: *Pax2*, *Pax3*, and *Pax7*. PAX (paired-box) family genes form a small yet critical family of developmental genes encoding transcription factors that regulate cell proliferation, cell-lineage specification, migration, and survival [52],[53]. There is experimental evidence linking PAX genes to the development and function of the cerebellum [54]. Although no *Pax3* cerebellar phenotype has yet been described in mice or humans, a Dandy-Walker malformation locus (abnormal cerebellar vermis) has recently been mapped to the upstream region of the *Pax3* gene [55], suggesting that *Pax3* misregulation may be involved in human cerebellar development. The superimposition of genetic locus information on the cerebellar malformation gene network thus is likely to generate lists of candidate genes for mapped phenotypes.

Yet another pair of predictions, the *Hoxc5* and *Hoxc6* genes, are neighbors on chromosome 12 in the human genome and belong to the HOX (homeo-box-containing) family. HOX genes are major players in vertebrate embryonic pattern formation, particularly (but not exclusively) in the central nervous system [56]. Again, no cerebellar phenotypes have been reported for these genes. Notably, however, these genes are never expressed in the developing cerebellum but do have well known roles in more posterior regions of the developing central nervous system. Thus, these particular Hox gene predictions highlight the importance of future work to integrate additional known biological information such as expression patterns (for example, GeneSat) into the gene network to generate more biologically relevant predictions.

#### Additional observations and predictions

See Text S1 and Dataset S1 for our Gene-set enrichment and Cross-Validation experiments.

### Conclusion

We have provided two very large molecular-interaction sets for human and mouse genes (Datasets S2, S3, S4, S5). The sets were integrated through gene orthology and are immediately applicable to a spectrum of experimental data analysis tasks (Dataset S6). Our analysis of mouse mutant cerebellar phenotypes, with the aid of our text-mined networks, lead to a number of intuitively reasonable and biologically testable predictions.

Our present study shares its spirit, goals, and some methods with other efforts in the field. For example, one of the most recent studies succeeded in integrating a diverse array of approaches to design a tool generating new disease-related hypotheses [57]. This group was able to combine information extraction [58], biomedical ontology mining, statistical analysis of sequences of natural language tokens, probabilistic analysis of error patterns across data types, computational reasoning, understanding of large-scale experimental datasets, and exploratory visualization in one application.

Because we are releasing our complete set of annotated data to public domain, these data might be instrumental for direct comparison for analogous text-mining results produced elsewhere [59]–[67].

Automated reasoning over text-mined, experimental, and machine-deduced data (Reading, Reasoning, and Reporting, as [57] put it), is likely to become a dominant mode of science in the near future, as size of experimental datasets and complexity of natural system under scrutiny grows.

## Methods

### GeneWays system and GeneWays 7.0 database

GeneWays is an information extraction (text-mining) system: It ingests computer-encoded full-text research articles or journal abstracts and distills from them a collection of biological relations. The architecture and implementation of the system are described in great detail elsewhere [3], [4], [6], [68]–[71], so here we provide just a brief outline of the system.

In a simplified view, the processing pipeline inside the GeneWays system is a sequence of just two steps. The first step deals with recognition of words or phrases representing important biological entities (such as *p53*, *Alzheimer's disease*, or *endoplasmic reticulum*; computer scientists call this step *named entity recognition*, NER). The second step deals with detecting relations among the entities (such as *p53 activates JAK*) and is called *information extraction* (IE).

Our NER module (MarkIt, [72]) works by following a hierarchy of rules defined by human experts. Our IE module (GENIES, [3],[68]) also is based on a collection of expert-encoded rules, but the underlying mathematical model is a bit more sophisticated (a deterministic context-free grammar). We use MarkIt to identify a spectrum of biological entities, such as disease, process, gene, protein, RNA, small molecule, tissue, and species. We apply GENIES to each individual sentence, trying to reconstruct the most probable steps that led to generation of the sentence. This reconstruction process is called *parsing*; besides satisfying our academic curiosity, parsing is useful for capturing complex relationships between entities. The results of parsing depend strongly on the formal grammar implemented in the parser.

**Figure 5:**
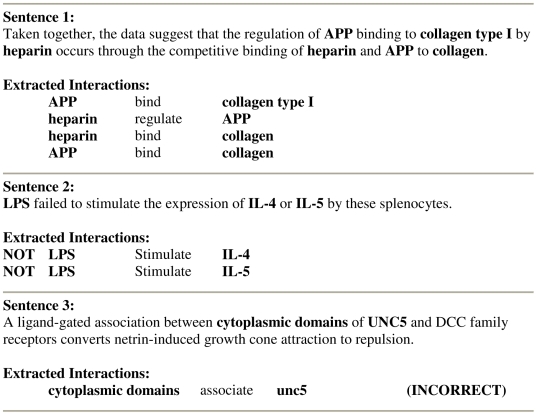
Examples of text-mined statements. Sentence 1: four correctly extracted interactions from one sentence. Sentence 2: two correctly extracted negative interactions. Sentence 3: an incorrectly extracted interaction.

Most of the relations that we can extract from biomedical texts are directional (*A activates B* is not the same as *B activates A*) and binary (only two entities are involved, which we call *upstream* and *downstream*, according to their position within the relation). A sentence can contain any number between none and dozens of relations. We can think of a typical binary relation as a quadruplet of values: [negation, upstream entity, action, downstream entity] (see Figure 5). Negation allows us to capture negative statements (“In our experiment, AKT failed to phosphorylate BAD”→[not, AKT, phosphorylate, BAD]) as well as positive statements. Relation type (action) indicates semantic connection between the two entities (*bind*, *activate*, *methylate*, *transport*, *is part of*, *is homolog of*, etc.).

To facilitate automatic reasoning over semantic groups of relations, we arrange them into an acyclic directed graph, where graph arcs represent the relation “is a.” The GeneWays system currently recognizes 391 different action types, 207 of which are frequent (see Table S1).

### Textual and text-mined data

To generate the molecular networks described in this study, we analyzed 368,331 full-text articles and 8,039,972 article abstracts from PubMed (see Table S8). The system identified 5,890,150 relations in the full text articles and 2,534,299 relations in the abstracts: in total, 5,934,024 unique relationships. The action types with the largest number of relationships are *induce* (695,615), *bind* (532,385), *inhibit* (386,523), *associate* (370,133), *contain* (366,654), and *activate* (332,336); the numbers in parentheses indicate the abundance of relations of each type in the GeneWays 7.0 database.

## Supporting Information

Text S1 Supplementary experiments; supplementary methods.
(0.34 MB DOC)

Figure S1 Interactive mouse brain head model.
(1.17 MB PDF)

Table S1 Complete list of Geneways action types.
(0.07 MB XLS)

Table S2 Pairwise phenotype overlap. The significance is measured based on a whole genome comprised of 25,000 genes.
(0.03 MB DOC)

Table S3 Pairwise phenotype overlap. The significance is measured based on a ataxia sub-network comprising 172 genes.
(0.03 MB DOC)

Table S4 Results of the tests for high connectivity of the phenotype genes.
(0.01 MB DOC)

Table S5 Results of the tests for clustering of the phenotype genes.
(0.02 MB PDF)

Table S6 Complete gene predictions.
(0.06 MB XLS)

Table S7 Initial genes.
(0.04 MB XLS)

Table S8 Text corpus description.
(0.03 MB XLS)

Table S9 Complete gene predictions (ini-trn, FDR 0.0001).
(1.72 MB XLS)

Dataset S1 All enrichment results.
(0.20 MB ZIP)

Dataset S2 Human network files.
(5.50 MB DOC)

Dataset S3 Human action mentions.
(8.87 MB ZIP)

Dataset S4 Mouse network files.
(6.41 MB ZIP)

Dataset S5 Mouse action mentions.
(10.07 MB ZIP)

Dataset S6 Combined human network.
(6.42 MB ZIP)
