# Supplementary material for: Looking at Cerebellar Malformations through Text-Mined Interactomes of Mice and Humans
Source: PLoS Comput Biol. 2009 Nov 6;5(11):e1000559. doi: 10.1371/journal.pcbi.1000559 (PMC2767227; doi:10.1371/journal.pcbi.1000559)
Supplement: Text S1 — Supplementary experiments; supplementary methods. (0.34 MB DOC) [file pcbi.1000559.s001.doc]

**Supplementary Text S1**

**Looking at cerebellar malformations through text-mined interactomes of mice and humans**

Ivan Iossifov, Raul Rodriguez-Esteban, Ilya Mayzus,

Kathleen J. Millen, and Andrey Rzhetsky

[Supplementary Experiments 1](#__RefHeading___Toc240351988)

[Cross-Validation Experiment 1](#__RefHeading___Toc240351989)

[Gene set enrichment 2](#__RefHeading___Toc240351990)

[Supplementary Methods 4](#__RefHeading___Toc240351991)

[Mapping names to genes 4](#__RefHeading___Toc240351992)

[Quality-of-extraction assessment 5](#__RefHeading___Toc240351993)

[Parametric model for a test for clustering of a gene list 6](#__RefHeading___Toc240351994)

[Test for a connectivity bias in a gene list 7](#__RefHeading___Toc240351995)

[Molecular triangulation for genes and gene sets 8](#__RefHeading___Toc240351996)

# Supplementary Experiments

## Cross-Validation Experiment

To address the question of whether the automatically extracted interactions improve our ability to predict phenotype related genes, we performed leave-one-out (LOO) cross-validation experiments to compare the prediction performances of our method based on the Human Protein Reference Database (HPRD) network [1,2] and that based on the union network built by extending HPRD with our automatically extracted interactions. For every phenotype we removed one gene at a time from the known genes, performed gene prediction based on the rest of the phenotype’s genes (initial genes), and examined how well the removed gene scored in the results based on the two networks. In total there were 615 removed-gene-from-a-phenotype experiments, including an artificial phenotype ‘all’ represented by the union of all analyzed phenotypes; thus, every gene was considered in at least two different contexts/phenotypes). In every experiment, all of the network genes were assigned a *p*-value based on number of interactions they have with the initial genes as compared to the number of interactions in a randomly rewired network; all genes were ranked based on their *p*-values and we considered the experiment a success if the removed gene was among the first 20 genes; otherwise it was considered a failure.

In this setup, not all of removed genes were part of the networks and the union network had a trivial advantage of containing more genes: We could perform 597 of the 615 experiments with the union network and only 534 of the experiments in the HPRD network. For a fairer comparison we only focused on the 534 experiments that could be scored in both networks. Of these, 5 experiments were positive with respect to both networks, 28 were positive only in with respect to the union network, 11 were positive only with respect to the HPRD network, and the other 490 experiments were negative with respect to both networks (See Table B).

We used these results in two different ways. First, we considered the 39 experiments where the two networks yielded different results. Cleary the union network produced more correct answers than the HPRD (28 versus 11). If the two networks were equally good for the purposes of prediction, we would have expected about an equal number of successes with respect to both networks. Thus, a binomial distribution with p=0.5 seems appropriate for the number of successful experiments for the union network. Under this null model (binomial distribution with p = 0.5 and n = 39) the observation of 28 leads to a *p*-value of 0.0047. The significance increases if we use the first 30, the first 40, or the first 50 genes with *p*-values of 4 × 10^–4^ (34 vs. 11 genes), 7 × 10^–5^ (39 vs. 11 genes), and 10^–5^ (45 vs. 12 genes), respectively. Second, we estimated the recall/sensitivity of the two networks. The union network recovered 33 (5 + 28) of the 534 experiments, which yields a recall of 0.06 (CI: [0.04, 0.09]), and the HPRD network recovered 16 of the 534 experiments, with an estimated recall of 0.03 (CI: [0.02, 0.05]). Although these two intervals overlap and we can not claim statistical significance when we consider the first 20 genes, the situation becomes clearer if we increase the cutoff: When we consider the first 50 genes the recall estimates become 0.11 (CI: [0.08, 0.14]) for the union network and 0.05 (CI: [0.03, 0.07]) for the HPRD networks. Both approaches support the union network as better suited for prediction of phenotype genes.

| **Table B. The number of successful leave-one-out experiments with respect to the union and HPRD networks under different rank cutoffs.** | | | | | |
| --- | --- | --- | --- | --- | --- |
| **Successful experiments in the** | | **Rank cutoff** | | | |
| **Union** | **HPRD** | **20** | **30** | **40** | **50** |
| **1** | **1** | 5 | 8 | 12 | 13 |
| **1** | **0** | 28 | 34 | 38 | 45 |
| **0** | **1** | 11 | 11 | 11 | 12 |
| **0** | **0** | 490 | 481 | 473 | 464 |

## Gene set enrichment

A complementary way to make gene candidate predictions is to look at sets of genes associated with biological themes. The approach is based on the following idea. Given a statistical technique that suggests candidate genes and a background model of noise, we can estimate the confidence of our prediction. If we have a set of genes grouped by a common theme in which multiple genes exhibit positive but weakly significant signals, multiple theme-related genes would produce a cumulative signal that has a higher statistical significance than that for any individual gene. To minimize the number of tests, we must start with predefined sets of genes, such as those provided by the MSigDB database [3]. Although the authors of MSigDB developed the database specifically for analysis of gene expression data, it is perfectly suited for use with molecular triangulation. Our test statistic when using gene sets with molecular triangulation was the total number of interactions the gene set had with our experimentally derived cerebellum malformation-specific genes. The predefined gene sets in MSigDB belong to the following categories: sets curated by human experts, sets derived by sequence motif analysis, sets generated by computational analysis of data other than sequences, and sets borrowed from Gene Ontology [4]. As with the initial triangulation, we were dealing with a multiple statistical testing problem and therefore had to adjust estimates of significance accordingly: We show here only sets with significance passing the FDR < 0.001 [5,6] (see Tables A to X in Dataset S1). To compute a background distribution of the test statistics we again used degree-preserving network rewiring.

As we hoped, significant gene sets were exceedingly more abundant than significant genes in the analogous single-gene test (see Tables A to X in Dataset S1). The most significant results for the *cerebellar degeneration* phenotype were associated with DNA binding, DNA damage detection, and DNA repair pathways. For example, several of the RAD family genes encode enzymes that facilitate DNA repair ad homologous recombination and maintaining the overall stability of the genome. In other words, malfunction of RAD-family proteins can lead to increased risk of DNA damage. The relationship between DNA damage and apoptosis (i.e., degeneration) is well established [7,8]. Other significant gene sets were associated with cell cycle: Regulation of cell proliferation and programmed cell death is implemented through an intricate and highly interconnected molecular network (see Tables A to X in Dataset S1).

Both *abnormal foliation* and *abnormal vermis* phenotypes appeared significantly associated with the “HSA04340 Hedgehog signaling pathway” set of genes. Because *Sonic hedgehog* (*Shh*) is one of the major players in vertebrate and invertebrate neural tube formation and is directly implicated in cerebellar development, these results are reasonable and somewhat expected. Similarly, *abnormal foliation* was significantly associated with sets of genes called “multicellular organism development” and “nervous system development,” while *abnormal vermis* was significantly associated with “brain development” genes. Furthermore, *small cerebellum* was associated with “anatomical structure development” and “pattern specification process” genes. These results appear to be reasonable and unsurprising; however, it is not easy to predict or explain why these specific sets of developmental genes were significantly associated with one phenotype and not another.

Other predictions generated by our analysis were more surprising. For example, the composite phenotype of *ataxia* was significantly linked to set of genes called “HSA05010 Alzheimer’s disease.” Furthermore, the *absent cerebellum* phenotype showed strong association with a set of genes that appear to have a *Pax4* regulatory sequence (set “V$PAX4_02”), thereby implicating the *Pax4* gene in association with the phenotype (see discussion of PAX family in the previous section).

Two sets of molecular triangulation analyses provided us with distinct but compatible projections of the complex pathway machinery linked to our phenotypes. For example, the gene set “anatomical structure development” includes members of the FGF and FGFR families (see discussion in the previous section). Our single-gene predictions can be mapped to one or more sets produced by the gene set analysis.

# Supplementary Methods

## Mapping names to genes

Connecting gene mentions scattered through scientific texts to unique gene sequence identifiers is difficult for a number of reasons. First, due to the globally uncoordinated nature of scientific discovery, a gene name’s origin can be traced to any of the world languages, to popular culture, historic events, biological phenomena, and even jokes [9]. There is no all-inclusive dictionary of gene names, and the overall gene name vocabulary is growing faster than any other vocabulary known to man. Second, also because of anarchy in gene naming, many genes have as many as a few dozen aliases (synonyms), and it is not uncommon for one gene name to refer to multiple distinct genes within the same organism (homonyms). Third, printed spelling of the same gene name can vary considerably, gaining or losing white spaces, hyphens, and slashes, suffering conversion of Arabic numerals into Roman (2 to *II*) or words (2 to *two*), and conversion of Greek characters into Roman (*α* to *a*) or words (*α* to *alpha*). Given the difficulty of exhaustive spell checking of gene names, typographical errors in gene names are not rare in the published texts. Fourth, to shorten the text, scientific writers often replace gene names with globally ambiguous abbreviations and pronouns.

Our term-to-ID mapping is dictionary-based and uses ideas that were proposed in two earlier studies [10,11]. Although there are multiple databases providing unique sequence identifiers, we have chosen to use GeneIDs of the GenBank database [12] maintained by the National Center for Biotechnology Information, NCBI.

We began our mapping by editing NCBI-generated dictionaries of human and mouse gene names and their synonyms. Our editing of the NCBI dictionary was guided by the goal of increasing the precision of the mapping (while sacrificing recall). We retained only unambiguously mapped gene names (i.e., those linked to a single human or mouse gene). We also removed short (fewer than three characters) gene synonyms and semantically ambiguous gene names, such as *CAMP*, *PKC*, and *ATP*. Although these are legitimate gene names (*CAMP* stands for cathelicidin antimicrobial peptide; *PKC* is paroxysmal kinesigenic choreoathetosis; and *ATP* is a synonym of ATP8A2, ATPase, aminophospholipid transporter-like, Class I, type 8A, member 2), in the research articles these abbreviations almost invariably refer to different molecules: cyclic adenosine monophosphate (cAMP), protein kinase C (PKC), and adenosine triphosphate (ATP).

We applied this pruning-matching strategy to both human and mouse gene names. For example, in the case of human genes, we started with 160,470 names derived from NCBI and pruned them to 155,072 names mapped to unique gene identifiers. Even after pruning the initial dictionary we still could not use exact matching of gene names because of spelling variation [10]: We had to use a set of string transformations, such as removing or adding spaces, converting the plural form of a name to singular one, and removing token “gene” or “protein” to obtain exact matching. Finally, having mapped all gene name aliases to a unique gene identifier (many-to-one relationship), we assigned unique gene identifiers to the names of genes or their products participating in text-derived relations.

We did not explicitly link action mentions to species (such as mouse or human) and did not disambiguate the semantic category of entities (gene, RNA, or protein within the gene-or-RNA-or-protein semantic class). In the former case, we relied on restrictions imposed by species-specific gene name dictionaries and the number of entities in each action mention. In the latter case, a partial semantic disambiguation came free as a by-product of biological constraints imposed on the semantic identities of arguments of each action.

## Quality-of-extraction assessment

Automated text mining can generate large amounts of imperfectly extracted data in a relatively short time. In contrast, human curators can extract information with rather high (albeit, not absolute) precision, but they work relatively slowly and their work is also rather expensive. We can fuse the advantages of both approaches by starting with a large collection of text-mined statements, asking several experts to annotate the automatically extracted facts as correctly or incorrectly extracted, and then building an artificial-intelligence curator that mimics the humans in distinguishing correctly extracted facts from incorrect ones. In a recent study we implemented exactly this approach [13], achieving near-human performance. We asked a group of curators to annotate a set of nearly 100,000 pairs of natural-text sentences and the corresponding automatically extracted statements. Using this large training corpus, we implemented a battery of automated classifiers and compared their performance with performance of experts. In the present study we used the maximum entropy classifier defined on pairs of features of extracted statements [13]. The classifier provided a quality score for every text-mined statement; using training data we identified the precision levels corresponding to a spectrum of cutoff scores. We were then able to automatically filter text-mined statements using a precision threshold of 0.9.

## Parametric model for a test for clustering of a gene list

The parametric test (Mod) involved comparison of two likelihood values computed for the same data using two nested probabilistic models. In both models, each pair of genes was stochastically joined with an edge with probability *θ* or remained disconnected with probability 1–*θ*. In the more complex model, the probability of interaction between a pair of *phenotype-associated* genes ($\theta_{P}$) was allowed to be distinct from the probability of interaction within the *remaining* gene pairs ($\theta_{R}$). In the slightly simpler model, the probability of interaction was assumed to be the same for any pair of genes ($\theta_{P}=\theta_{R}$).

In our model, we start with two disjoint sets of genes: $S_{P}$ with $n$ genes linked to a given phenotype, *P;* and $S_{\bar{P}}$ with $m$ remaining genes within our network. We form two new sets of unordered *pairs* of genes. The first such set, $M_{P}$, contains all unordered pairs of genes from $S_{P}$, excluding pairs with repeated entries ($M_{P}=\{(\nu,\upsilon):\nu\in S_{P},\upsilon\in S_{P},\nu\neq\upsilon\}$). The second set,$M_{R}$, contains the rest of all possible unordered heterogeneous pairs of genes ($M_{R}=\{(\nu,\upsilon):\nu\in S_{\bar{P}},\upsilon\in S_{\bar{P}},\nu\neq\upsilon\}\cup\{(\nu,\upsilon):\nu\in S_{P},\upsilon\in S_{\bar{P}}\}$). The sizes of the$M_{P}$ and $M_{R}$sets are given by $|M_{P}|=n(n-1)/2$ and$|M_{R}|=m(m-1)/2+nm$. We then apply the stochastic network generation models proposed by Hofman and Wiggins [14]. In these models, the decision to form an edge is made for every unordered pair of genes with probability $\theta_{P}$ for the pairs in set$M_{P}$ and probability $\theta_{R}$ for the pairs in$M_{R}$. These assumptions lead to the following probability of observing $p$and *r* interactions within sets$M_{P}$and$M_{R}$, respectively:

$L(p,r|\theta_{P},\theta_{R},S_{P},S_{\bar{P}})=\left(\begin{matrix} |M_{P}| \\ p \end{matrix}\right)\theta_{P}^{p}(1-\theta_{P}^{p})^{|M_{P}|-p}\left(\begin{matrix} |M_{R}| \\ r \end{matrix}\right)\theta_{R}^{r}(1-\theta_{R}^{r})^{|M_{R}|-r}$.

In our analysis we observe $p$ and $r$ directly, given a phenotype and a molecular network. The maximum likelihood estimates of $\theta_{P}$and $\theta_{R}$are then given by $\hat{\theta}_{P}=\frac{p}{|M_{P}|}$and $\hat{\theta}_{R}=\frac{r}{|M_{R}|}$. We also consider a simpler nested model in which the probability of forming an edge is the same for the pairs in $M_{P}$and in $M_{R}$: $\theta_{P}=\theta_{R}\mathop{=}\limits^{def} \theta_{null}$. The maximum likelihood estimate for $\theta_{null}$is given by $\hat{\theta}_{null}=\frac{p+r}{|M_{P}|+|M_{R}|}.$ Because the models are nested and the difference in the number of free parameters between the two models is one ($\theta_{R}$ and $\theta_{P}$ versus $\theta_{null}$), the doubled absolute difference between their maximized log-likelihood values asymptotically follows a *χ*^2^ distribution with one degree of freedom *when the simpler model is used to generate data* [15]*.*

## Test for a connectivity bias in a gene list

To test the hypothesis that highly connected genes are overrepresented in the lists of genes capable of affecting cerebellar phenotypes, we implemented a parametric test based on a simple generative model. In this model, the probability of a node being sampled depends explicitly on the node’s connectivity: The probability is proportional to$i^{\alpha}$, where $i$ is the graph-theoretic degree of the node and *α* is a real-valued parameter. When $\alpha$ is set to zero, every node is sampled with the same probability; in contrast, positive or negative$\alpha$ encourages preferential sampling of more or less connected nodes, respectively. As long as the degree-blind model (with $\alpha=0$) is a special case of a model with unconstrained values of *α*, we can use a likelihood ratio statistic to test the non-randomness of network node selection.

Given a network having a maximum node degree of$D$and containing $n_{i}$ nodes with degree$i$ for$i=1,\ldots,D$, we postulate that the probability of selecting a set of nodes with $k_{i}$ nodes with degree $i$ for $i=1,\ldots,D$to be:

$L(k_{1},\ldots,k_{D}|n_{1},\ldots,n_{D},\alpha)=\left(\frac{(k_{1}+\cdots+k_{D})!}{k_{1}!\cdots k_{D}!}\right)\delta_{1}^{k_{1}}\cdots\delta_{D}^{k_{D}}$, where $\delta_{i}=\frac{n_{i}i^{\alpha}}{\sum_{j=1}^{D} n_{j}j^{\alpha}}$.

While it is difficult to find an analytical solution for *α* that maximizes the above likelihood ($\hat{\alpha}$), it is quite easy to obtain$\hat{\alpha}$numerically. If the subset sampling were blind to the degrees of the nodes, the following statistic would approximately follow a$\chi^{2}$distribution with one degree of freedom [15]:

$S=2\log\frac{L(k_{1},\ldots,k_{D}|n_{1},\ldots,n_{D},\alpha=\hat{\alpha})}{L(k_{1},\ldots,k_{D}|n_{1},\ldots,n_{D},\alpha=0)}$.

## Molecular triangulation for genes and gene sets

We extended the original triangulation technique [16] in three major directions. First, we incorporated individual scoring of gene sets; second, we implemented estimation of the background score distributions using network rewiring; and third, we introduced an additional clustering scoring function based on simple counting of network edges.

Both the original and the modified triangulation approaches begin with a set of so-called *initial* genes that are known to be associated with the phenotype of interest. Using knowledge embodied by a molecular network, the initial genes then are used to suggest additional genes that are putatively associated with the same phenotype. The newly implicated genes are typically network neighbors of multiple initial genes.

In our triangulation analyses we assume that we have a known undirected molecular network, where *N* is a set of genes and *E* is a set of their interactions. Further, we have a pre-defined set of sets of the network genes, ; a set of initial genes, *I*; and a real-valued function $e(\nu)$for all $\nu\in I$, representing our confidence in the decision to include a given gene in the initial set. We then define a score function, which represents the degree to which the set $T_{i}$is implicated by the initial evidence in the context of the network , and we aim to identify subsets in that appear closer to the initial genes (or, equivalently, to have a higher score) than expected under a specified null model.

Here we define in two different ways. In the first approach (used in gene prediction efforts), we use all single-gene sets $T_{i}=\{g_{i}\}$ covering every gene$g_{i}\in N$. In the second approach (used in gene set enrichment analysis), we use gene sets that are defined in the MSigDB [3].

We define two different scores (*trn* and *cnt*) and two different background models (*ini* and *rwr*). The first score is defined as in the initial study [16]:

where $d_{uv}$is the length of the shortest path between the genes $u$and $\nu$within network . The second score,, is the number of edges in $E$ that connect a node from$T_{i}$with a node from $I$. (Note that the $S^{cnt}$score ignores the strength of the evidence, the $e$-function.) The first background model (*ini*) assumes that the set of initial nodes $I$ is sampled uniformly from the network nodes in$N$. The second background model (*rwr*) assumes instead that the network edges $E$are attached to nodes using a random rewiring process so that every gene preserves its observed degree [17].

Once we choose a score function $S$and a background model, we can generate *B replicates* according to the background model (*B* random initial sets$I^{j}$in the case of the *ini* background model or *B* random networks $E^{j}$in the case of the *rwr* model). We compute the score for the given initial set and the given network and the background score $s_{i}^{j}$for each of the background replicates ( for *ini* and for *rwr*) for every test set $T_{i}$. We can then assign a *p*-value for each $T_{i}$ based on how many of the background scores are higher or equal to the real score:

$\hat{p}_{i}=\frac{|\{s_{i}^{j}:s_{i}^{j}\geq s_{i}^{real}\}|}{B}$.

If all of the background scores are lower than the real score, the above definition will assign a *p*-value of 0. To avoid the reporting of such zero *p*-values, we use two different approaches. We either replace zero *p*-values with $1/[2\times B]$ or we use “smoothed” *p*-values by fitting the background scores $\{s_{i}^{j}\}_{j=1,\ldots,B}$to a normal distribution and computing the *p*-values based on the normal distribution. The smoothed *p*-values approach is better in assigning small *p*-values to cases where the real score is much higher than all of the background scores. Unfortunately, however, the normal distribution assumption is not automatically warranted and has to be carefully tested with respect to the data. In our experiments we found it safe to assume a normal distribution of the background scores when the $S^{trn}$score function is used.

**References**

1. Mishra GR, Suresh M, Kumaran K, Kannabiran N, Suresh S, et al. (2006) Human protein reference database--2006 update. Nucleic Acids Res 34: D411-414.

2. Peri S, Navarro JD, Kristiansen TZ, Amanchy R, Surendranath V, et al. (2004) Human protein reference database as a discovery resource for proteomics. Nucleic Acids Res 32: D497-501.

3. Subramanian A, Tamayo P, Mootha VK, Mukherjee S, Ebert BL, et al. (2005) Gene set enrichment analysis: a knowledge-based approach for interpreting genome-wide expression profiles. Proc Natl Acad Sci U S A 102: 15545-15550.

4. Ashburner M, Ball CA, Blake JA, Botstein D, Butler H, et al. (2000) Gene ontology: tool for the unification of biology. The Gene Ontology Consortium. Nat Genet 25: 25-29.

5. Benjamini Y, Yekutieli D (2001) The control of the false discovery rate in multiple testing under dependency. Annals of Statistics 29: 1165-1188.

6. Benjamini Y, Hochberg Y (1995) Controlling the False Discovery Rate - a Practical and Powerful Approach to Multiple Testing. Journal of the Royal Statistical Society Series B-Methodological 57: 289-300.

7. Norbury CJ, Zhivotovsky B (2004) DNA damage-induced apoptosis. Oncogene 23: 2797-2808.

8. Roos WP, Kaina B (2006) DNA damage-induced cell death by apoptosis. Trends Mol Med 12: 440-450.

9. Seringhaus MR, Cayting PD, Gerstein MB (2008) Uncovering trends in gene naming. Genome Biol 9: 401.

10. Cohen AM. Unsupervised gene/protein entity normalization using automatically extracted dictionaries.; 2005; Detroit, MI. Association for Computational Linguistics. pp. 17-24.

11. Fang H-r, Murphy K, Jin Y, Kim J, White P. Human gene name normalization using text matching with automatically extracted synonym dictionaries; 2006; New York, NY. Association for Computational Linguistics. pp. 41--48.

12. Burks C, Fickett JW, Goad WB, Kanehisa M, Lewitter FI, et al. (1985) The GenBank nucleic acid sequence database. Comput Appl Biosci 1: 225-233.

13. Rodriguez-Esteban R, Iossifov I, Rzhetsky A (2006) Imitating manual curation of text-mined facts in biomedicine. PLoS Comput Biol 2: e118.

14. Hofman JM, Wiggins CH (2008) Bayesian approach to network modularity. Phys Rev Lett 100: 258701.

15. Wilks SS (1938) The large-sample distribution of the likelihood ratio for testing com-

posite hypotheses. Ann Math Statist 9: 60-62.

16. Krauthammer M, Kaufmann CA, Gilliam TC, Rzhetsky A (2004) Molecular triangulation: Bridging linkage and molecular-network information for identifying candidate genes in Alzheimer's disease. Proc Natl Acad Sci U S A 101: 15148-15153.

17. Feldman I, Rzhetsky A, Vitkup D (2008) Network properties of genes harboring inherited disease mutations. Proc Natl Acad Sci U S A 105: 4323-4328.
