# Supplementary figures and images for: Looking at Cerebellar Malformations through Text-Mined Interactomes of Mice and Humans
Source: PLoS Comput Biol. 2009 Nov 6;5(11):e1000559. doi: 10.1371/journal.pcbi.1000559 (PMC2767227; doi:10.1371/journal.pcbi.1000559)

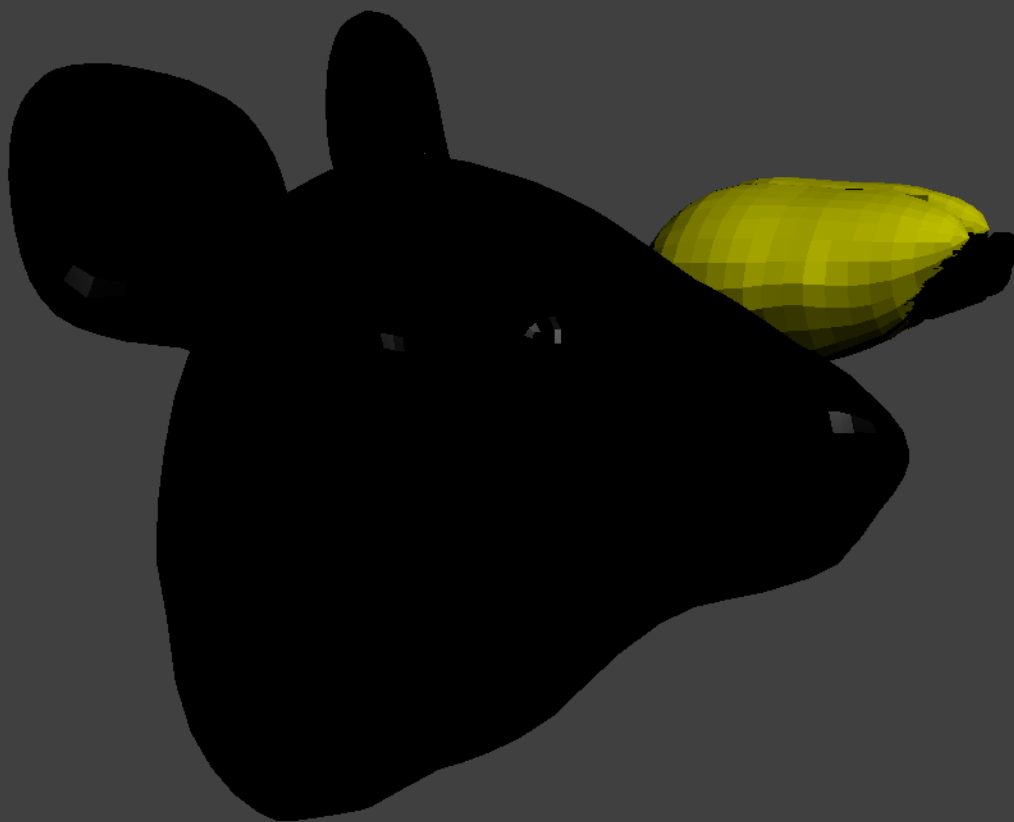

Supplement: Figure S1 — Interactive mouse brain head model. (1.17 MB PDF) [file pcbi.1000559.s002.pdf]
