# Supplementary material for: Looking at Cerebellar Malformations through Text-Mined Interactomes of Mice and Humans
Source: PLoS Comput Biol. 2009 Nov 6;5(11):e1000559. doi: 10.1371/journal.pcbi.1000559 (PMC2767227; doi:10.1371/journal.pcbi.1000559)
Supplement: Table S2 — Pairwise phenotype overlap. The significance is measured based on a whole genome comprised of 25,000 genes. (0.03 MB DOC) [file pcbi.1000559.s004.pdf]

Table S2. Pairwise phenotype overlap. The significance is measures based on a whole genome comprised of 25,000 genes. ↓ the observed overlap is less than expected.

	degeneration	abnormal foliation	abnormal vermis	absent cerebellum	small cerebellum	ataxia
**gene count**	39	56	24	10	70	172
**abnormal foliation**	1					
expected	0.087					
p-value	0.003					
**abnormal vermis**	2	11				
expected	0.037	0.054				
p-value	7×10^{-6}	2×10^{-11}				
**absent cerebellum**	0↓	2	3			
expected	0.016	0.022	0.010			
p-value	0.985	1×10^{-6}	2×10^{-10}			
**small cerebellum**	8	37	13	2		
expected	0.109	0.157	0.067	0.028		
p-value	$<10^{-12}$	2×10^{-12}	4×10^{-11}	2×10^{-6}		
**ataxia**	28	22	12	2	36	
expected	0.268	0.385	0.165	0.069	0.482	
p-value	$<10^{-12}$	2×10^{-11}	5×10^{-11}	4×10^{-5}	4×10^{-11}	
