# Supplementary material for: Looking at Cerebellar Malformations through Text-Mined Interactomes of Mice and Humans
Source: PLoS Comput Biol. 2009 Nov 6;5(11):e1000559. doi: 10.1371/journal.pcbi.1000559 (PMC2767227; doi:10.1371/journal.pcbi.1000559)
Supplement: Table S3 — Pairwise phenotype overlap. The significance is measured based on a ataxia sub-network comprising 172 genes. (0.03 MB DOC) [file pcbi.1000559.s005.pdf]

Table S3. Pairwise phenotype overlap. The significance is measures based on a ataxia sub-network comprising 172 genes. ↓ the observed overlap is less than expected.

	degeneration	abnormal foliation	abnormal vermis	absent cerebellum	small cerebellum
**gene count**	28	22	12	2	36
**abnormal foliation**	1↓				
expected	3.581				
p-value	0.090				
**abnormal vermis**	1↓	8			
expected	1.953	1.535			
p-value	0.387	3×10^{-7}			
**absent cerebellum**	0↓	1	1		
expected	0.326	0.256	0.140		
p-value	0.700	0.016	0.004		
**small cerebellum**	7	18	10	1	
expected	5.860	4.605	2.512	0.419	
p-value	0.200	1×10^{-12}	9×10^{-8}	0.043	
