# Supplementary material for: Looking at Cerebellar Malformations through Text-Mined Interactomes of Mice and Humans
Source: PLoS Comput Biol. 2009 Nov 6;5(11):e1000559. doi: 10.1371/journal.pcbi.1000559 (PMC2767227; doi:10.1371/journal.pcbi.1000559)
Supplement: Table S4 — Results of the tests for high connectivity of the phenotype genes. (0.01 MB DOC) [file pcbi.1000559.s006.pdf]

Table S4. Results of the tests for high connectivity of the phenotype genes.

	p-value			
	**whole**	**hprd**	**physical**	**Y2H**
**all**	1×10^{-25}	1×10^{-10}	5×10^{-17}	0.045
**degeneration**	6×10^{-5}	0.224	0.010	0.613
**abnormal foliation**	1×10^{-08}	0.002	7×10^{-6}	0.267
**abnormal vermis**	6×10^{-07}	3×10^{-4}	5×10^{-6}	0.045
**absent cerebellum**	4×10^{-4}	0.852	0.414	N/A
**small cerebellum**	8×10^{-09}	2×10^{-4}	6×10^{-6}	0.843
**ataxia**	5×10^{-15}	2×10^{-7}	2×10^{-11}	0.061
