# Supplementary material for: Looking at Cerebellar Malformations through Text-Mined Interactomes of Mice and Humans
Source: PLoS Comput Biol. 2009 Nov 6;5(11):e1000559. doi: 10.1371/journal.pcbi.1000559 (PMC2767227; doi:10.1371/journal.pcbi.1000559)
Supplement: Table S5 — Results of the tests for clustering of the phenotype genes. (0.02 MB PDF) [file pcbi.1000559.s007.pdf]

Table S5. Results of the tests for clustering of the phenotype genes. See the main text for a description of the three tests Mod, Ini, and Rwr.

		whole	hprd	physical
**network size [Nodes / Edges]**		[11,530 / 98,634]	[9,303 / 34,998]	[10,475 / 51,974]
**all**		[236 / 399]	[212 / 120]	[225 / 184]
p-values	Mod	$< 1\times10^{-16}$	$< 1\times10^{-16}$	$< 1\times10^{-16}$
	Ini	< 0.001	< 0.001	< 0.001
	Rwr	< 0.001	< 0.001	< 0.001
**degeneration**		[39 / 10]	[34 / 2]	[38 / 4]
p-values	Mod	3×10^{-7}	0.092	0.006
	Ini	0.003	0.066	0.008
	Rwr	0.002	0.199	0.085
**abnormal foliation**		[55 / 47]	[49 / 15]	[52 / 21]
p-values	Mod	$< 1\times10^{-16}$	1×10^{-13}	$< 1\times10^{-16}$
	Ini	< 0.001	< 0.001	< 0.001
	Rwr	< 0.001	< 0.001	< 0.001
**abnormal vermis**		[24 / 17]	[21 / 4]	[22 / 5]
p-values	Mod	$< 1\times10^{-16}$	3×10^{-5}	3×10^{-6}
	Ini	< 0.001	< 0.001	< 0.001
	Rwr	< 0.001	0.184	0.27
**absent cerebellum**		[10 / 13]	[9 / 0]	[9 / 1]
p-values	Mod	$< 1\times10^{-16}$	N/A	0.028
	Ini	< 0.001	1	0.03
	Rwr	< 0.001	1	0.081
**small cerebellum**		[68 / 54]	[60 / 20]	[64 / 27]
p-values	Mod	$< 1\times10^{-16}$	$< 1\times10^{-16}$	$< 1\times10^{-16}$
	Ini	< 0.001	< 0.001	< 0.001
	Rwr	< 0.001	< 0.001	< 0.001
**ataxia**		[165 / 176]	[149 / 49]	[157 / 84]
p-values	Mod	$< 1\times10^{-16}$	1×10^{-16}	$< 1\times10^{-16}$
	Ini	< 0.001	< 0.001	< 0.001
	Rwr	< 0.001	< 0.001	< 0.001
