# Supplementary material for: Looking at Cerebellar Malformations through Text-Mined Interactomes of Mice and Humans
Source: PLoS Comput Biol. 2009 Nov 6;5(11):e1000559. doi: 10.1371/journal.pcbi.1000559 (PMC2767227; doi:10.1371/journal.pcbi.1000559)
Supplement: Dataset S1 — All enrichment results. (0.20 MB ZIP) [file pcbi.1000559.s012.zip › enrichment_results/Table A. enrichment_hprd-abnormal foliation.html]

Complete Clustering results for network hprd and phenotype abnormal foliation (FDR <= 0.001)


# Complete Clustering results for network hprd and phenotype abnormal foliation (FDR <= 0.001)

| Set | p-Value | Gene Count | Interaction Count | Expected Interection Count |
| --- | --- | --- | --- | --- |
| HSA04340\_HEDGEHOG\_SIGNALING\_PATHWAY (c2) Genes involved in Hedgehog signaling pathway | 3.52496e-13 | 43/57 | 23 | 5.984 |
| INTEGRIN\_COMPLEX (c5) Genes annotated by the GO term GO:0008305. Any member of a family of heterodimeric transmembrane receptors for cell-adhesion molecules. The alpha and beta subunits are noncovalently bonded. | 2.19436e-12 | 18/19 | 12 | 2.132 |
| REELINPATHWAY (c2) Reelin is secreted by neurons and recognized by receptors including cadherin related neuronal receptors, which promote phosphorylation of Dab1. | 4.75076e-12 | 6/7 | 13 | 2.631 |
| FOSBPATHWAY (c2) FOSB gene expression and drug abuse | 3.76614e-11 | 4/5 | 7 | 0.898 |
| chr7q36 (c1) Genes in cytogenetic band chr7q36 | 5.25219e-11 | 21/68 | 8 | 1.23 |
| P35ALZHEIMERSPATHWAY (c2) p35, a neuron-specific activator of cyclin-dependent kinase 5, is cleaved to p25 in Alzheimer's disease and promotoes hyperphosphorylated tau formation and apoptosis. | 6.73708e-11 | 10/11 | 17 | 4.176 |
| CELL\_CYCLE\_GO\_0007049 (c5) Genes annotated by the GO term GO:0007049. The progression of biochemical and morphological phases and events that occur in a cell during successive cell replication or nuclear replication events. Canonically, the cell cycle comprises the replication and segregation of genetic material followed by the division of the cell, but in endocycles or syncytial cells nuclear replication or nuclear division may not be followed by cell division. | 6.30979e-10 | 282/311 | 79 | 43.048 |
| EMBRYONIC\_MORPHOGENESIS (c5) Genes annotated by the GO term GO:0048598. The process by which anatomical structures are generated and organized during the embryonic phase. Morphogenesis pertains to the creation of form. The embryonic phase begins with zygote formation. The end of the embryonic phase is organism-specific. For example, it would be at birth for mammals, larval hatching for insects and seed dormancy in plants. | 7.22872e-10 | 11/17 | 6 | 0.773 |
| CELL\_SOMA (c5) Genes annotated by the GO term GO:0043025. The portion of a cell bearing surface projections such as axons, dendrites, cilia, or flagella that includes the nucleus, but excludes all cell projections. | 3.1451e-09 | 9/10 | 7 | 1.044 |
| HSA04115\_P53\_SIGNALING\_PATHWAY (c2) Genes involved in p53 signaling pathway | 1.76624e-08 | 58/66 | 37 | 16.211 |
| DNA\_RECOMBINATION (c5) Genes annotated by the GO term GO:0006310. The processes by which a new genotype is formed by reassortment of genes resulting in gene combinations different from those that were present in the parents. In eukaryotes genetic recombination can occur by chromosome assortment, intrachromosomal recombination, or nonreciprocal interchromosomal recombination. Intrachromosomal recombination occurs by crossing over. In bacteria it may occur by genetic transformation, conjugation, transduction, or F-duction. | 2.55103e-08 | 41/47 | 20 | 6.401 |
| POSITIVE\_REGULATION\_OF\_DEVELOPMENTAL\_PROCESS (c5) Genes annotated by the GO term GO:0051094. Any process that activates or increases the rate or extent of development, the biological process whose specific outcome is the progression of an organism over time from an initial condition (e.g. a zygote, or a young adult) to a later condition (e.g. a multicellular animal or an aged adult). | 2.95129e-08 | 194/215 | 69 | 38.657 |
| SHHPATHWAY (c2) Sonic hedgehog (Shh) signaling in the developing CNS induces neuronal proliferation via interaction with the patched (Ptc-1) and smoothened receptors. | 2.99009e-08 | 12/14 | 10 | 2.187 |
| GROWTH\_CONE (c5) Genes annotated by the GO term GO:0030426. The migrating motile tip of a growing nerve cell axon or dendrite. | 5.25351e-08 | 9/10 | 8 | 1.55 |
| DNA\_DAMAGE\_SIGNALING (c2) Genes involved in DNA damage signaling | 5.50843e-08 | 84/89 | 37 | 16.573 |
| ACETAMINOPHENPATHWAY (c2) Acetaminophen selectively inhibits Cox-3, which is localized to the brain, and yields the toxic metabolite NAPQI when processed by CAR in the liver. | 5.54132e-08 | 4/6 | 2 | 0.131 |
| HSA04512\_ECM\_RECEPTOR\_INTERACTION (c2) Genes involved in ECM-receptor interaction | 6.09852e-08 | 74/86 | 26 | 9.986 |
| CELL\_CYCLE\_REGULATOR (c2) Obsolete by GO - was not defined before being made obsolete | 6.20208e-08 | 19/21 | 17 | 5.39 |
| BRAIN\_DEVELOPMENT (c5) Genes annotated by the GO term GO:0007420. The process whose specific outcome is the progression of the brain over time, from its formation to the mature structure. The brain is one of the two components of the central nervous system and is the center of thought and emotion. It is responsible for the coordination and control of bodily activities and the interpretation of information from the senses (sight, hearing, smell, etc.). | 6.66081e-08 | 34/51 | 10 | 2.303 |
| RESPONSE\_TO\_DNA\_DAMAGE\_STIMULUS (c5) Genes annotated by the GO term GO:0006974. A change in state or activity of a cell or an organism (in terms of movement, secretion, enzyme production, gene expression, etc.) as a result of a stimulus indicating damage to its DNA from environmental insults or errors during metabolism. | 8.19815e-08 | 148/161 | 47 | 23.41 |
| P53\_SIGNALING (c2) Genes involved in p53 signaling | 9.35505e-08 | 85/91 | 72 | 41.331 |
| CELL\_ADHESION\_RECEPTOR\_ACTIVITY (c2) Obsolete by GO - combining with cell adhesion molecules to initiate a change in cell activity. | 1.02754e-07 | 30/33 | 15 | 4.41 |
| module\_275 (c4) Genes in module\_275 | 1.30851e-07 | 15/16 | 11 | 2.74 |
| HSA04110\_CELL\_CYCLE (c2) Genes involved in cell cycle | 1.38828e-07 | 106/112 | 74 | 43.954 |
| XENOBIOTIC\_METABOLIC\_PROCESS (c5) Genes annotated by the GO term GO:0006805. The chemical reactions and pathways involving a xenobiotic compound, a compound foreign to living organisms. Used of chemical compounds, e.g. a xenobiotic chemical, such as a pesticide. | 1.52475e-07 | 3/11 | 2 | 0.137 |
| CELLCYCLEPATHWAY (c2) Cyclins interact with cyclin-dependent kinases to form active kinase complexes that regulate progression through the cell cycle. | 1.54396e-07 | 22/23 | 23 | 8.764 |
| NUCLEUS (c5) Genes annotated by the GO term GO:0005634. A membrane-bounded organelle of eukaryotic cells in which chromosomes are housed and replicated. In most cells, the nucleus contains all of the cell's chromosomes except the organellar chromosomes, and is the site of RNA synthesis and processing. In some species, or in specialized cell types, RNA metabolism or DNA replication may be absent. | 1.77634e-07 | 1201/1417 | 224 | 170.674 |
| V$E2F1\_Q3\_01 (c3) Genes with promoter regions [-2kb,2kb] around transcription start site containing the motif TTGGCGCGRAANNGNM which matches annotation for E2F1: E2F transcription factor 1 | 1.93342e-07 | 149/193 | 37 | 17.132 |
| BRENTANI\_CELL\_CYCLE (c2) Cancer related genes involved in the cell cycle | 3.45609e-07 | 78/79 | 40 | 18.845 |
| DNA\_METABOLIC\_PROCESS (c5) Genes annotated by the GO term GO:0006259. The chemical reactions and pathways involving DNA, deoxyribonucleic acid, one of the two main types of nucleic acid, consisting of a long, unbranched macromolecule formed from one, or more commonly, two, strands of linked deoxyribonucleotides. | 4.10594e-07 | 228/256 | 60 | 33.5 |
| DNA\_REPAIR (c5) Genes annotated by the GO term GO:0006281. The process of restoring DNA after damage. Genomes are subject to damage by chemical and physical agents in the environment (e.g. UV and ionizing radiations, chemical mutagens, fungal and bacterial toxins, etc.) and by free radicals or alkylating agents endogenously generated in metabolism. DNA is also damaged because of errors during its replication. A variety of different DNA repair pathways have been reported that include direct reversal, base excision repair, nucleotide excision repair, photoreactivation, bypass, double-strand break repair pathway, and mismatch repair pathway. | 4.45667e-07 | 116/125 | 39 | 18.894 |
| CACTTTG,MIR-520G,MIR-520H (c3) Targets of MicroRNA CACTTTG,MIR-520G,MIR-520H | 7.36517e-07 | 150/216 | 39 | 18.967 |
| RORIE\_ES\_PNET\_DN (c2) The 30 genes showing the greatest decrease in expression in NBa Ews/Fli-1 infectants | 9.08035e-07 | 22/26 | 8 | 1.739 |
| RESPONSE\_TO\_ENDOGENOUS\_STIMULUS (c5) Genes annotated by the GO term GO:0009719. A change in state or activity of a cell or an organism (in terms of movement, secretion, enzyme production, gene expression, etc.) as a result of an endogenous stimulus. | 9.25723e-07 | 177/198 | 49 | 26.271 |
| HSA04510\_FOCAL\_ADHESION (c2) Genes involved in focal adhesion | 1.25604e-06 | 179/192 | 88 | 57.168 |
| G1PATHWAY (c2) CDK4/6-cyclin D and CDK2-cyclin E phosphorylate Rb, which allows the transcription of genes needed for the G1/S cell cycle transition. | 1.34878e-06 | 25/26 | 30 | 13.488 |
| SITE\_OF\_POLARIZED\_GROWTH (c5) Genes annotated by the GO term GO:0030427. Any part of a cell where non-isotropic growth takes place. | 1.47827e-06 | 10/11 | 8 | 1.833 |
| chr3q24 (c1) Genes in cytogenetic band chr3q24 | 1.72488e-06 | 7/20 | 2 | 0.163 |
| TAAYNRNNTCC\_UNKNOWN (c3) Genes with promoter regions [-2kb,2kb] around transcription start site containing motif TAAYNRNNTCC. Motif does not match any known transcription factor | 2.19337e-06 | 99/129 | 20 | 7.79 |
| G2PATHWAY (c2) Activated Cdc2-cyclin B kinase regulates the G2/M transition; DNA damage stimulates the DNA-PK/ATM/ATR kinases, which inactivate Cdc2. | 2.25438e-06 | 22/23 | 28 | 12.544 |
| CELL\_DEVELOPMENT (c5) Genes annotated by the GO term GO:0048468. The process whose specific outcome is the progression of the cell over time, from its formation to the mature structure. Cell development does not include the steps involved in committing a cell to a specific fate. | 2.29265e-06 | 510/571 | 135 | 95.015 |
| ATRBRCAPATHWAY (c2) BRCA1 and 2 block cell cycle progression in response to DNA damage and promote double-stranded break repair; mutations induce breast cancer susceptibility. | 2.36451e-06 | 20/21 | 17 | 6.082 |
| REGULATION\_OF\_CELL\_CYCLE (c5) Genes annotated by the GO term GO:0051726. Any process that modulates the rate or extent of progression through the cell cycle. | 2.41507e-06 | 165/180 | 49 | 27.09 |
| DNA\_DAMAGE\_RESPONSE\_\_SIGNAL\_TRANSDUCTION (c5) Genes annotated by the GO term GO:0042770. A cascade of processes induced by the detection of DNA damage within a cell. | 3.15776e-06 | 29/34 | 20 | 7.965 |
| HSA05217\_BASAL\_CELL\_CARCINOMA (c2) Genes involved in basal cell carcinoma | 4.02483e-06 | 43/55 | 20 | 8.178 |
| REGULATION\_OF\_CYCLIN\_DEPENDENT\_PROTEIN\_KINASE\_ACTIVITY (c5) Genes annotated by the GO term GO:0000079. Any process that modulates the frequency, rate or extent of CDK activity. | 4.5259e-06 | 41/43 | 16 | 5.541 |
| CELL\_CYCLE\_PROCESS (c5) Genes annotated by the GO term GO:0022402. A cellular process that is involved in the progression of biochemical and morphological phases and events that occur in a cell during successive cell replication or nuclear replication events. | 4.96299e-06 | 173/191 | 46 | 25.421 |
| GRAEBER\_BETA2\_INTEGRINS (c2) Genes in the beta2 integrins family | 6.46521e-06 | 10/11 | 8 | 1.987 |
| REGULATION\_OF\_DEVELOPMENTAL\_PROCESS (c5) Genes annotated by the GO term GO:0050793. Any process that modulates the frequency, rate or extent of development, the biological process whose specific outcome is the progression of a multicellular organism over time from an initial condition (e.g. a zygote, or a young adult) to a later condition (e.g. a multicellular animal or an aged adult). | 7.72072e-06 | 393/436 | 111 | 76.796 |
| INTEGRIN\_MEDIATED\_CELL\_ADHESION\_KEGG (c2) | 8.03741e-06 | 80/90 | 45 | 25.624 |
| UVB\_NHEK3\_C4 (c2) Regulated by UV-B light in normal human epidermal keratinocytes, cluster 4 | 8.35861e-06 | 9/12 | 7 | 1.641 |
| ACATATC,MIR-190 (c3) Targets of MicroRNA ACATATC,MIR-190 | 9.06788e-06 | 33/56 | 11 | 3.324 |
| V$PITX2\_Q2 (c3) Genes with promoter regions [-2kb,2kb] around transcription start site containing the motif WNTAATCCCAR which matches annotation for PITX2: paired-like homeodomain transcription factor 2 | 9.52665e-06 | 140/206 | 33 | 16.573 |
| NDKDYNAMINPATHWAY (c2) Endocytotic role of NDK, Phosphins and Dynamin | 9.62779e-06 | 18/19 | 13 | 4.465 |
