# Supplementary material for: Looking at Cerebellar Malformations through Text-Mined Interactomes of Mice and Humans
Source: PLoS Comput Biol. 2009 Nov 6;5(11):e1000559. doi: 10.1371/journal.pcbi.1000559 (PMC2767227; doi:10.1371/journal.pcbi.1000559)
Supplement: Dataset S1 — All enrichment results. (0.20 MB ZIP) [file pcbi.1000559.s012.zip › enrichment_results/Table B. enrichment_hprd-abnormal vermis.html]

Complete Clustering results for network hprd and phenotype abnormal vermis (FDR <= 0.001)


# Complete Clustering results for network hprd and phenotype abnormal vermis (FDR <= 0.001)

| Set | p-Value | Gene Count | Interaction Count | Expected Interection Count |
| --- | --- | --- | --- | --- |
| RNA\_BIOSYNTHETIC\_PROCESS (c5) Genes annotated by the GO term GO:0032774. The chemical reactions and pathways resulting in the formation of RNA, ribonucleic acid, one of the two main type of nucleic acid, consisting of a long, unbranched macromolecule formed from ribonucleotides joined in 3',5'-phosphodiester linkage. Includes polymerization of ribonucleotide monomers. | 1e-20 | 562/636 | 130 | 70.041 |
| TRANSCRIPTION\_\_DNA\_DEPENDENT (c5) Genes annotated by the GO term GO:0006351. The synthesis of RNA on a template of DNA. | 1.11022e-16 | 561/634 | 129 | 70.034 |
| TRANSCRIPTION (c5) Genes annotated by the GO term GO:0006350. The synthesis of either RNA on a template of DNA or DNA on a template of RNA. | 1.44329e-15 | 665/750 | 150 | 88.315 |
| SA\_REG\_CASCADE\_OF\_CYCLIN\_EXPR (c2) Expression of cyclins regulates progression through the cell cycle by activating cyclin-dependent kinases. | 1.44329e-15 | 11/13 | 17 | 3.247 |
| NUCLEOBASE\_\_NUCLEOSIDE\_\_NUCLEOTIDE\_AND\_NUCLEIC\_ACID\_METABOLIC\_PROCESS (c5) Genes annotated by the GO term GO:0006139. The chemical reactions and pathways involving nucleobases, nucleosides, nucleotides and nucleic acids. | 1.9984e-15 | 1055/1234 | 187 | 116.482 |
| module\_198 (c4) Genes in module\_198 | 3.88578e-15 | 279/301 | 85 | 40.659 |
| module\_98 (c4) Genes in module\_98 | 5.66214e-15 | 361/391 | 97 | 48.588 |
| NUCLEUS (c5) Genes annotated by the GO term GO:0005634. A membrane-bounded organelle of eukaryotic cells in which chromosomes are housed and replicated. In most cells, the nucleus contains all of the cell's chromosomes except the organellar chromosomes, and is the site of RNA synthesis and processing. In some species, or in specialized cell types, RNA metabolism or DNA replication may be absent. | 1.44329e-14 | 1201/1417 | 210 | 138.285 |
| DNA\_METABOLIC\_PROCESS (c5) Genes annotated by the GO term GO:0006259. The chemical reactions and pathways involving DNA, deoxyribonucleic acid, one of the two main types of nucleic acid, consisting of a long, unbranched macromolecule formed from one, or more commonly, two, strands of linked deoxyribonucleotides. | 1.40554e-13 | 228/256 | 61 | 26.84 |
| TRANSCRIPTION\_FROM\_RNA\_POLYMERASE\_II\_PROMOTER (c5) Genes annotated by the GO term GO:0006366. The synthesis of RNA from a DNA template by RNA polymerase II (Pol II), originating at a Pol II-specific promoter. Includes transcription of messenger RNA (mRNA) and certain small nuclear RNAs (snRNAs). | 3.60934e-13 | 407/456 | 98 | 52.064 |
| module\_197 (c4) Genes in module\_197 | 6.22724e-13 | 148/173 | 52 | 20.831 |
| CELL\_CYCLE\_GO\_0007049 (c5) Genes annotated by the GO term GO:0007049. The progression of biochemical and morphological phases and events that occur in a cell during successive cell replication or nuclear replication events. Canonically, the cell cycle comprises the replication and segregation of genetic material followed by the division of the cell, but in endocycles or syncytial cells nuclear replication or nuclear division may not be followed by cell division. | 1.73328e-12 | 282/311 | 71 | 34.005 |
| REGULATION\_OF\_NUCLEOBASE\_\_NUCLEOSIDE\_\_NUCLEOTIDE\_AND\_NUCLEIC\_ACID\_METABOLIC\_PROCESS (c5) Genes annotated by the GO term GO:0019219. Any process that modulates the frequency, rate or extent of the chemical reactions and pathways involving nucleobases, nucleosides, nucleotides and nucleic acids. | 9.02456e-12 | 539/614 | 123 | 74.294 |
| module\_124 (c4) Genes in module\_124 | 1.86788e-11 | 90/96 | 31 | 10.636 |
| RESPONSE\_TO\_DNA\_DAMAGE\_STIMULUS (c5) Genes annotated by the GO term GO:0006974. A change in state or activity of a cell or an organism (in terms of movement, secretion, enzyme production, gene expression, etc.) as a result of a stimulus indicating damage to its DNA from environmental insults or errors during metabolism. | 2.09603e-11 | 148/161 | 45 | 18.553 |
| HSA00100\_BIOSYNTHESIS\_OF\_STEROIDS (c2) Genes involved in biosynthesis of steroids | 2.13886e-11 | 10/24 | 3 | 0.184 |
| RNA\_METABOLIC\_PROCESS (c5) Genes annotated by the GO term GO:0016070. The chemical reactions and pathways involving RNA, ribonucleic acid, one of the two main type of nucleic acid, consisting of a long, unbranched macromolecule formed from ribonucleotides joined in 3',5'-phosphodiester linkage. | 4.21008e-11 | 724/835 | 131 | 80.72 |
| NUCLEAR\_PART (c5) Genes annotated by the GO term GO:0044428. Any constituent part of the nucleus, a membrane-bounded organelle of eukaryotic cells in which chromosomes are housed and replicated. | 6.62668e-11 | 492/572 | 102 | 58.607 |
| BRENTANI\_CELL\_CYCLE (c2) Cancer related genes involved in the cell cycle | 8.43392e-11 | 78/79 | 38 | 14.29 |
| REGULATION\_OF\_TRANSCRIPTION (c5) Genes annotated by the GO term GO:0045449. Any process that modulates the frequency, rate or extent of the synthesis of either RNA on a template of DNA or DNA on a template of RNA. | 1.21479e-10 | 493/563 | 113 | 69.078 |
| HSA04130\_SNARE\_INTERACTIONS\_IN\_VESICULAR\_TRANSPORT (c2) Genes involved in SNARE interactions in vesicular transport | 1.39786e-10 | 30/36 | 11 | 2.056 |
| module\_403 (c4) Genes in module\_403 | 1.7234e-10 | 39/46 | 23 | 7.277 |
| G1\_TO\_S\_CELL\_CYCLE\_REACTOME (c2) | 3.02871e-10 | 63/66 | 34 | 12.898 |
| REGULATION\_OF\_TRANSCRIPTION\_\_DNA\_DEPENDENT (c5) Genes annotated by the GO term GO:0006355. Any process that modulates the frequency, rate or extent of DNA-dependent transcription. | 3.66643e-10 | 400/459 | 90 | 51.641 |
| REGULATION\_OF\_RNA\_METABOLIC\_PROCESS (c5) Genes annotated by the GO term GO:0051252. Any process that modulates the frequency, rate or extent of the chemical reactions and pathways involving RNA. | 4.08273e-10 | 408/468 | 91 | 52.56 |
| REGULATION\_OF\_CELLULAR\_METABOLIC\_PROCESS (c5) Genes annotated by the GO term GO:0031323. Any process that modulates the frequency, rate or extent of the chemical reactions and pathways by which individual cells transform chemical substances. | 4.85988e-10 | 688/782 | 137 | 89.687 |
| REGULATION\_OF\_METABOLIC\_PROCESS (c5) Genes annotated by the GO term GO:0019222. Any process that modulates the frequency, rate or extent of the chemical reactions and pathways within a cell or an organism. | 5.5692e-10 | 699/794 | 139 | 91.342 |
| TRANSCRIPTION\_INITIATION (c5) Genes annotated by the GO term GO:0006352. Processes involved in the assembly of the RNA polymerase complex at the promoter region of a DNA template resulting in the subsequent synthesis of RNA from that promoter. | 1.08426e-09 | 34/35 | 15 | 3.971 |
| CELL\_CYCLE\_KEGG (c2) | 1.10095e-09 | 79/84 | 48 | 21.99 |
| CELLCYCLEPATHWAY (c2) Cyclins interact with cyclin-dependent kinases to form active kinase complexes that regulate progression through the cell cycle. | 1.10841e-09 | 22/23 | 21 | 6.571 |
| RESPONSE\_TO\_ENDOGENOUS\_STIMULUS (c5) Genes annotated by the GO term GO:0009719. A change in state or activity of a cell or an organism (in terms of movement, secretion, enzyme production, gene expression, etc.) as a result of an endogenous stimulus. | 1.58775e-09 | 177/198 | 46 | 20.852 |
| BIOPOLYMER\_METABOLIC\_PROCESS (c5) Genes annotated by the GO term GO:0043283. The chemical reactions and pathways involving biopolymers, long, repeating chains of monomers found in nature e.g. polysaccharides and proteins. | 2.28695e-09 | 1426/1667 | 222 | 160.965 |
| CELL\_PROLIFERATION\_GO\_0008283 (c5) Genes annotated by the GO term GO:0008283. The multiplication or reproduction of cells, resulting in the expansion of a cell population. | 2.93496e-09 | 443/513 | 84 | 47.172 |
| REGULATION\_OF\_GENE\_EXPRESSION (c5) Genes annotated by the GO term GO:0010468. Any process that modulates the frequency, rate or extent of gene expression. Gene expression is the process in which a gene's coding sequence is converted into a mature gene product or products (proteins or RNA). This includes the production of an RNA transcript as well as any processing to produce a mature RNA product or an mRNA (for protein-coding genes) and the translation of that mRNA into protein. Some protein processing events may be included when they are required to form an active form of a product from an inactive precursor form. | 3.80944e-09 | 582/670 | 118 | 75.667 |
| module\_252 (c4) Genes in module\_252 | 4.04501e-09 | 219/235 | 63 | 33.123 |
| CELL\_CYCLE (c2) The progression of biochemical and morphological events that occur during nuclear or cellular replication. | 5.42998e-09 | 72/76 | 44 | 20.251 |
| REGULATION\_OF\_TRANSCRIPTION\_FROM\_RNA\_POLYMERASE\_II\_PROMOTER (c5) Genes annotated by the GO term GO:0006357. Any process that modulates the frequency, rate or extent of transcription from an RNA polymerase II promoter. | 6.17148e-09 | 252/288 | 63 | 33.478 |
| HSA04115\_P53\_SIGNALING\_PATHWAY (c2) Genes involved in p53 signaling pathway | 9.12547e-09 | 58/66 | 32 | 13.125 |
| NEGATIVE\_REGULATION\_OF\_TRANSCRIPTION (c5) Genes annotated by the GO term GO:0016481. Any process that stops, prevents or reduces the frequency, rate or extent of transcription. | 1.08014e-08 | 163/187 | 53 | 27.374 |
| DNA\_REPAIR (c5) Genes annotated by the GO term GO:0006281. The process of restoring DNA after damage. Genomes are subject to damage by chemical and physical agents in the environment (e.g. UV and ionizing radiations, chemical mutagens, fungal and bacterial toxins, etc.) and by free radicals or alkylating agents endogenously generated in metabolism. DNA is also damaged because of errors during its replication. A variety of different DNA repair pathways have been reported that include direct reversal, base excision repair, nucleotide excision repair, photoreactivation, bypass, double-strand break repair pathway, and mismatch repair pathway. | 1.10432e-08 | 116/125 | 35 | 14.926 |
| PARP\_KO\_DN (c2) Downregulated in MEF cells from PARP knockout mice | 1.23615e-08 | 11/14 | 7 | 1.116 |
| DNA\_BINDING (c5) Genes annotated by the GO term GO:0003677. Interacting selectively with DNA (deoxyribonucleic acid). | 1.32317e-08 | 482/600 | 100 | 62.376 |
| HOFMANN\_MANTEL\_LYMPHOMA\_VS\_LYMPH\_NODES\_UP (c2) Genes whose expression is up-regulated in mantle cell lymphoma compared to hyperplastic lymph nodes as analyzed by oligonucleotide microarray | 1.51154e-08 | 43/47 | 23 | 8.217 |
| NEGATIVE\_REGULATION\_OF\_NUCLEOBASE\_\_NUCLEOSIDE\_\_NUCLEOTIDE\_AND\_NUCLEIC\_ACID\_METABOLIC\_PROCESS (c5) Genes annotated by the GO term GO:0045934. Any process that stops, prevents or reduces the frequency, rate or extent of the chemical reactions and pathways involving nucleobases, nucleosides, nucleotides and nucleic acids. | 1.92075e-08 | 184/209 | 55 | 28.824 |
| NUCLEAR\_LUMEN (c5) Genes annotated by the GO term GO:0031981. The volume enclosed by the nuclear inner membrane. | 2.72745e-08 | 328/381 | 74 | 42.759 |
| G1PATHWAY (c2) CDK4/6-cyclin D and CDK2-cyclin E phosphorylate Rb, which allows the transcription of genes needed for the G1/S cell cycle transition. | 3.13584e-08 | 25/26 | 26 | 10.03 |
| chr14q (c1) Genes in cytogenetic band chr14q | 3.56649e-08 | 4/11 | 3 | 0.269 |
| module\_158 (c4) Genes in module\_158 | 6.07706e-08 | 39/43 | 16 | 4.907 |
| NEGATIVE\_REGULATION\_OF\_CELLULAR\_PROCESS (c5) Genes annotated by the GO term GO:0048523. Any process that stops, prevents or reduces the frequency, rate or extent of cellular processes, those that are carried out at the cellular level, but are not necessarily restricted to a single cell. For example, cell communication occurs among more than one cell, but occurs at the cellular level. | 8.85028e-08 | 556/640 | 114 | 74.86 |
| HSA05218\_MELANOMA (c2) Genes involved in melanoma | 9.2556e-08 | 64/71 | 37 | 17.856 |
| INTEGRIN\_COMPLEX (c5) Genes annotated by the GO term GO:0008305. Any member of a family of heterodimeric transmembrane receptors for cell-adhesion molecules. The alpha and beta subunits are noncovalently bonded. | 1.04294e-07 | 18/19 | 8 | 1.687 |
| LAMB\_CYCLIN\_D3\_GLOCUS (c2) E2F target genes highly correlated with cyclin D3 expression (p = 0.002) | 1.33115e-07 | 13/15 | 9 | 2.157 |
| GNF2\_PCNA (c4) Neighborhood of PCNA | 1.7329e-07 | 55/65 | 17 | 5.622 |
| SKP2E2FPATHWAY (c2) E2F-1, a transcription factor that promotes the G1/S transition, is repressed by Rb and activated by cdk2/cyclin E. | 1.75472e-07 | 8/9 | 11 | 2.93 |
| module\_57 (c4) Genes in module\_57 | 1.75633e-07 | 53/56 | 34 | 15.402 |
| module\_125 (c4) Genes in module\_125 | 1.93201e-07 | 40/44 | 16 | 5.059 |
| ORGANELLE\_LUMEN (c5) Genes annotated by the GO term GO:0043233. The volume enclosed by the membranes of a particular organelle, e.g. endoplasmic reticulum lumen, or the space between the two lipid bilayers of a double membrane surrounding an organelle, e.g. nuclear membrane lumen. | 2.09537e-07 | 361/451 | 75 | 44.946 |
| MEMBRANE\_ENCLOSED\_LUMEN (c5) Genes annotated by the GO term GO:0031974. The enclosed volume within a sealed membrane or between two sealed membranes. | 2.09537e-07 | 361/451 | 75 | 44.946 |
| NUCLEOLUS (c5) Genes annotated by the GO term GO:0005730. A small, dense body one or more of which are present in the nucleus of eukaryotic cells. It is rich in RNA and protein, is not bounded by a limiting membrane, and is not seen during mitosis. Its prime function is the transcription of the nucleolar DNA into 45S ribosomal-precursor RNA, the processing of this RNA into 5.8S, 18S, and 28S components of ribosomal RNA, and the association of these components with 5S RNA and proteins synthesized outside the nucleolus. This association results in the formation of ribonucleoprotein precursors; these pass into the cytoplasm and mature into the 40S and 60S subunits of the ribosome. | 2.17514e-07 | 98/123 | 26 | 10.631 |
| PROTEIN\_DNA\_COMPLEX\_ASSEMBLY (c5) Genes annotated by the GO term GO:0065004. The aggregation, arrangement and bonding together of proteins and DNA molecules to form a protein-DNA complex. | 2.17577e-07 | 44/49 | 17 | 5.852 |
| SHEPARD\_CRASH\_AND\_BURN\_MUT\_VS\_WT\_UP (c2) Genes upregulated in the crash and burn zebra fish mutant | 2.80905e-07 | 97/143 | 25 | 10.317 |
| DNA\_DEPENDENT\_DNA\_REPLICATION (c5) Genes annotated by the GO term GO:0006261. The process whereby new strands of DNA are synthesized, using parental DNA as a template for the DNA-dependent DNA polymerases that synthesize the new strands. | 3.00031e-07 | 50/56 | 17 | 5.64 |
| NEGATIVE\_REGULATION\_OF\_BIOLOGICAL\_PROCESS (c5) Genes annotated by the GO term GO:0048519. Any process that stops, prevents or reduces the frequency, rate or extent of a biological process. Biological processes are regulated by many means; examples include the control of gene expression, protein modification or interaction with a protein or substrate molecule. | 3.02176e-07 | 584/670 | 115 | 77.168 |
| GNF2\_CENPF (c4) Neighborhood of CENPF | 3.57547e-07 | 47/58 | 15 | 4.766 |
| CELL\_CYCLE\_PROCESS (c5) Genes annotated by the GO term GO:0022402. A cellular process that is involved in the progression of biochemical and morphological phases and events that occur in a cell during successive cell replication or nuclear replication events. | 3.7175e-07 | 173/191 | 41 | 19.956 |
| SCHUMACHER\_MYC\_DN (c2) Genes down-regulated by MYC in P493-6 (B-cell) | 4.67848e-07 | 6/7 | 4 | 0.554 |
| BIOSYNTHESIS\_OF\_STEROIDS (c2) | 6.88151e-07 | 7/14 | 2 | 0.149 |
| SARCOMAS\_SYNOVIAL\_UP (c2) Top 20 positive significant genes associated with synovial sarcomas, versus other soft-tissue tumors. | 7.38514e-07 | 8/12 | 2 | 0.15 |
| PROLIFERATION\_GENES (c2) Proliferation related genes | 8.55611e-07 | 322/359 | 63 | 36.241 |
| TRANSCRIPTION\_INITIATION\_FROM\_RNA\_POLYMERASE\_II\_PROMOTER (c5) Genes annotated by the GO term GO:0006367. Processes involved in starting transcription from the RNA polymerase II promoter. | 9.66172e-07 | 28/29 | 12 | 3.659 |
| VERNELL\_PRB\_CLSTR1 (c2) pRB pathway target genes CLUSTER 1 The listed genes were found regulated by pRB and p16 and one of the E2Fs (E2F1, E2F2, or E2F3) Cluster 1 genes are up-regulated by E2F and down-regulated by pRB and p16 | 1.00261e-06 | 48/61 | 15 | 4.873 |
| NEGATIVE\_REGULATION\_OF\_METABOLIC\_PROCESS (c5) Genes annotated by the GO term GO:0009892. Any process that stops, prevents or reduces the frequency, rate or extent of the chemical reactions and pathways within a cell or an organism. | 1.06279e-06 | 232/260 | 59 | 34.236 |
| REGULATION\_OF\_CELL\_CYCLE (c5) Genes annotated by the GO term GO:0051726. Any process that modulates the rate or extent of progression through the cell cycle. | 1.13627e-06 | 165/180 | 41 | 21.275 |
| V$IK3\_01 (c3) Genes with promoter regions [-2kb,2kb] around transcription start site containing motif TNYTGGGAATACC. Motif does not match any known transcription factor | 1.15538e-06 | 124/169 | 20 | 7.426 |
| HSA04110\_CELL\_CYCLE (c2) Genes involved in cell cycle | 1.28132e-06 | 106/112 | 59 | 34.715 |
| TRANSCRIPTION\_REPRESSOR\_ACTIVITY (c5) Genes annotated by the GO term GO:0016564. Any transcription regulator activity that prevents or downregulates transcription. | 1.28793e-06 | 139/150 | 35 | 16.588 |
| PEART\_HISTONE\_UP (c2) Cell-proliferation-related genes upregulated by SAHA and depsipeptide (histone deacetylase inhibitors) | 1.3134e-06 | 47/51 | 16 | 5.446 |
| MYC\_ONCOGENIC\_SIGNATURE (c2) Genes selected in supervised analyses to discriminate cells expressing c-Myc oncogene from control cells expressing GFP. | 1.51409e-06 | 113/176 | 24 | 9.956 |
| SASAKI\_ATL\_UP (c2) Highly expressed genes in ATL cells compared with normal CD4 and CD4 CD45RO T cells | 1.56588e-06 | 132/161 | 35 | 17.063 |
| SASAKI\_TCELL\_LYMPHOMA\_VS\_CD4\_UP (c2) Genes overexpressed at least twofold in adult T-cell lymphoma (ATL) cells versus normal CD4+ and CD4+/CD45RO+ T cells. | 1.56588e-06 | 132/161 | 35 | 17.063 |
| HSA00900\_TERPENOID\_BIOSYNTHESIS (c2) Genes involved in terpenoid biosynthesis | 1.58933e-06 | 2/6 | 1 | 0.042 |
| TERPENOID\_BIOSYNTHESIS (c2) | 1.58933e-06 | 2/4 | 1 | 0.042 |
| NEGATIVE\_REGULATION\_OF\_CELL\_PROLIFERATION (c5) Genes annotated by the GO term GO:0008285. Any process that stops, prevents or reduces the rate or extent of cell proliferation. | 2.0097e-06 | 131/156 | 28 | 12.624 |
| NEGATIVE\_REGULATION\_OF\_CELLULAR\_METABOLIC\_PROCESS (c5) Genes annotated by the GO term GO:0031324. Any process that stops, prevents or reduces the frequency, rate or extent of the chemical reactions and pathways by which individual cells transform chemical substances. | 2.53587e-06 | 229/257 | 57 | 33.315 |
| DNA\_RECOMBINATION (c5) Genes annotated by the GO term GO:0006310. The processes by which a new genotype is formed by reassortment of genes resulting in gene combinations different from those that were present in the parents. In eukaryotes genetic recombination can occur by chromosome assortment, intrachromosomal recombination, or nonreciprocal interchromosomal recombination. Intrachromosomal recombination occurs by crossing over. In bacteria it may occur by genetic transformation, conjugation, transduction, or F-duction. | 2.8638e-06 | 41/47 | 15 | 5.072 |
| RNA\_ELONGATION (c5) Genes annotated by the GO term GO:0006354. The extension of an RNA molecule after transcription initiation by the addition of ribonucleotides catalyzed by an RNA polymerase. | 3.12519e-06 | 9/10 | 5 | 0.922 |
| CELL\_CYCLE\_PHASE (c5) Genes annotated by the GO term GO:0022403. A cell cycle process comprising the steps by which a cell progresses through one of the biochemical and morphological phases and events that occur during successive cell replication or nuclear replication events. | 3.55525e-06 | 156/169 | 37 | 18.503 |
| NEGATIVE\_REGULATION\_OF\_TRANSCRIPTION\_\_DNA\_DEPENDENT (c5) Genes annotated by the GO term GO:0045892. Any process that stops, prevents or reduces the frequency, rate or extent of DNA-dependent transcription. | 3.55922e-06 | 113/129 | 35 | 18.406 |
| NUCLEOPLASM (c5) Genes annotated by the GO term GO:0005654. That part of the nuclear content other than the chromosomes or the nucleolus. | 3.58087e-06 | 248/276 | 62 | 37.513 |
| REGULATION\_OF\_CELL\_PROLIFERATION (c5) Genes annotated by the GO term GO:0042127. Any process that modulates the frequency, rate or extent of cell proliferation. | 3.86522e-06 | 269/308 | 52 | 29.704 |
| BREASTCA\_TWO\_CLASSES (c2) Gene set that can be used to differentiate BRCA1-linked and BRCA2-linked breast cancers | 3.87062e-06 | 111/132 | 39 | 20.793 |
| P53\_SIGNALING (c2) Genes involved in p53 signaling | 3.92171e-06 | 85/91 | 56 | 33.382 |
| TRANSFERASE\_ACTIVITY\_\_TRANSFERRING\_PENTOSYL\_GROUPS (c5) Genes annotated by the GO term GO:0016763. Catalysis of the transfer of a pentosyl group from one compound (donor) to another (acceptor). | 3.92615e-06 | 12/20 | 4 | 0.601 |
| NEGATIVE\_REGULATION\_OF\_RNA\_METABOLIC\_PROCESS (c5) Genes annotated by the GO term GO:0051253. Any process that stops, prevents or reduces the frequency, rate or extent of the chemical reactions and pathways involving RNA. | 4.04456e-06 | 114/130 | 35 | 18.45 |
| CHANG\_SERUM\_RESPONSE\_UP (c2) CSR (Serum Response) signature for activated genes (Stanford) | 4.10746e-06 | 108/135 | 25 | 11.104 |
| TARTE\_PLASMA\_BLASTIC (c2) Genes overexpressed in mature plasma cells isolated from tonsils (TPCs) and mature plasma cells isolated from bone marrow (BMPCs) as compared to polyclonal plasmablastic cells (PPCs). | 4.39059e-06 | 235/286 | 47 | 26.177 |
| module\_54 (c4) Genes in module\_54 | 5.14693e-06 | 188/255 | 33 | 16.008 |
| GNF2\_CDC2 (c4) Neighborhood of CDC2 | 5.18105e-06 | 49/58 | 13 | 4.387 |
| DNAFRAGMENTPATHWAY (c2) DNA fragmentation during apoptosis is effected by DFF, a caspase-activated DNAse, and by endonuclease G. | 5.45018e-06 | 8/10 | 9 | 2.46 |
| ZHAN\_MULTIPLE\_MYELOMA\_VS\_NORMAL\_UP (c2) The 70 most significantly up-regulated genes in MM in comparison with normal bone marrow PCs | 5.57467e-06 | 47/60 | 15 | 5.281 |
| LEI\_HOXC8\_DN (c2) Downregulated target genes of murine transcription factor Hoxc8. | 6.09342e-06 | 14/15 | 4 | 0.628 |
| NUCLEAR\_REPLICATION\_FORK (c5) Genes annotated by the GO term GO:0043596. The Y-shaped region of a nuclear replicating DNA molecule, resulting from the separation of the DNA strands and in which the synthesis of new strands takes place. Also includes associated protein complexes. | 6.19542e-06 | 9/10 | 6 | 1.339 |
| V$COMP1\_01 (c3) Genes with promoter regions [-2kb,2kb] around transcription start site containing the motif NVTNWTGATTGACNACAAVARRBN which matches annotation for MYOG: myogenin (myogenic factor 4) | 6.56864e-06 | 69/94 | 14 | 4.806 |
| LE\_MYELIN\_UP (c2) Genes upregulated in Egr2Lo/Lo mice (who bear mutations in the transcription factor Egr2 and in which peripheral nerve myelination is disrupted) whose expression is significantly altered after sciatic nerve injury. | 7.77851e-06 | 68/87 | 18 | 6.74 |
| module\_308 (c4) Genes in module\_308 | 8.71687e-06 | 52/70 | 12 | 3.833 |
| DNA\_DEPENDENT\_ATPASE\_ACTIVITY (c5) Genes annotated by the GO term GO:0008094. Catalysis of the reaction: ATP + H2O = ADP + phosphate in the presence of single- or double-stranded DNA; drives another reaction. | 9.66333e-06 | 18/22 | 10 | 3.097 |
| GREENBAUM\_E2A\_UP (c2) Table includes transcripts up-regulated 3-fold or greater in the E2A-deficient cell lines | 9.69704e-06 | 30/33 | 8 | 2.02 |
| chr20p11 (c1) Genes in cytogenetic band chr20p11 | 1.03214e-05 | 21/68 | 4 | 0.667 |
| MITOTIC\_CELL\_CYCLE (c5) Genes annotated by the GO term GO:0000278. Progression through the phases of the mitotic cell cycle, the most common eukaryotic cell cycle, which canonically comprises four successive phases called G1, S, G2, and M and includes replication of the genome and the subsequent segregation of chromosomes into daughter cells. In some variant cell cycles nuclear replication or nuclear division may not be followed by cell division, or G1 and G2 phases may be absent. | 1.11885e-05 | 141/153 | 35 | 17.972 |
| MORF\_BUB1B (c4) Neighborhood of BUB1B | 1.42725e-05 | 54/65 | 16 | 6.132 |
| module\_318 (c4) Genes in module\_318 | 1.46532e-05 | 25/28 | 12 | 3.983 |
| TRANSCRIPTION\_ACTIVATOR\_ACTIVITY (c5) Genes annotated by the GO term GO:0016563. Any transcription regulator activity required for initiation or upregulation of transcription. | 1.48237e-05 | 163/171 | 43 | 24.31 |
| GROWTH\_FACTOR\_ACTIVITY (c5) Genes annotated by the GO term GO:0008083. The function that stimulates a cell to grow or proliferate. Most growth factors have other actions besides the induction of cell growth or proliferation. | 1.61157e-05 | 46/55 | 9 | 2.528 |
| module\_18 (c4) Genes in module\_18 | 1.84457e-05 | 345/447 | 57 | 34.544 |
| GNF2\_RRM1 (c4) Neighborhood of RRM1 | 1.93171e-05 | 71/85 | 17 | 6.868 |
