# Supplementary material for: Looking at Cerebellar Malformations through Text-Mined Interactomes of Mice and Humans
Source: PLoS Comput Biol. 2009 Nov 6;5(11):e1000559. doi: 10.1371/journal.pcbi.1000559 (PMC2767227; doi:10.1371/journal.pcbi.1000559)
Supplement: Dataset S1 — All enrichment results. (0.20 MB ZIP) [file pcbi.1000559.s012.zip › enrichment_results/Table C. enrichment_hprd-absent cerebellum.html]

Complete Clustering results for network hprd and phenotype absent cerebellum (FDR <= 0.001)


# Complete Clustering results for network hprd and phenotype absent cerebellum (FDR <= 0.001)

| Set | p-Value | Gene Count | Interaction Count | Expected Interection Count |
| --- | --- | --- | --- | --- |
| HSA05217\_BASAL\_CELL\_CARCINOMA (c2) Genes involved in basal cell carcinoma | 1e-20 | 43/55 | 14 | 1.502 |
| HSA04310\_WNT\_SIGNALING\_PATHWAY (c2) Genes involved in Wnt signaling pathway | 1e-20 | 123/147 | 37 | 8.132 |
| TRANSMEMBRANE\_RECEPTOR\_PROTEIN\_PHOSPHATASE\_ACTIVITY (c5) Genes annotated by the GO term GO:0019198. The catalysis of phosphate removal from a phosphotyrosine using aspartic acid as a nucleophile in a metal-dependent manner. | 1e-20 | 18/19 | 6 | 0.41 |
| HSA00534\_HEPARAN\_SULFATE\_BIOSYNTHESIS (c2) Genes involved in heparan sulfate biosynthesis | 1e-20 | 2/19 | 1 | 0.002 |
| HSA04520\_ADHERENS\_JUNCTION (c2) Genes involved in adherens junction | 1.11022e-16 | 71/75 | 29 | 8.291 |
| ST\_WNT\_BETA\_CATENIN\_PATHWAY (c2) Beta-catenin is degraded in the absence of Wnt signaling; when extracellular Wnt binds Frizzled receptors, beta-catenin accumulates in the nucleus and may promote cell survival. | 3.51941e-14 | 25/31 | 9 | 1.182 |
| PROTEIN\_TYROSINE\_PHOSPHATASE\_ACTIVITY (c5) Genes annotated by the GO term GO:0004725. Catalysis of the reaction: protein tyrosine phosphate + H2O = protein tyrosine + phosphate. | 6.63114e-12 | 48/53 | 9 | 1.416 |
| PS1PATHWAY (c2) Presenilin is required for gamma-secretase activity to activate Notch signaling; presenilin also inhibits beta-catenin in the Wnt/Frizzled pathway. | 2.0951e-11 | 11/12 | 6 | 0.696 |
| TSADAC\_RKOSILENT\_UP (c2) Genes basally silent, with hypermethylated promoters, upregulated by the combination of TSA and DAC in RKO cells | 4.18066e-11 | 12/18 | 2 | 0.084 |
| UVC\_LOW\_C3\_DN (c2) Down-regulated at 12 hours following treatment of WS1 human skin fibroblasts with UVC at a low dose (10 J/m^2) (cluster c3) | 1.11402e-10 | 16/19 | 3 | 0.198 |
| TRANSMEMBRANE\_RECEPTOR\_ACTIVITY (c5) Genes annotated by the GO term GO:0004888. Combining with an extracellular or intracellular messenger to initiate a change in cell activity, and spanning to the membrane of either the cell or an organelle. | 2.98534e-10 | 331/418 | 20 | 5.893 |
| HSA05210\_COLORECTAL\_CANCER (c2) Genes involved in colorectal cancer | 1.07447e-09 | 81/84 | 24 | 8.527 |
| chr5q21 (c1) Genes in cytogenetic band chr5q21 | 3.2298e-09 | 9/26 | 2 | 0.11 |
| PHOSPHORIC\_MONOESTER\_HYDROLASE\_ACTIVITY (c5) Genes annotated by the GO term GO:0016791. Catalysis of the hydrolysis of phosphoric monoesters, releasing inorganic phosphate. | 3.48555e-09 | 92/111 | 11 | 2.384 |
| DISTECHE\_XINACTIVATED\_GENES (c2) Genes that escape X inactivation | 5.28904e-09 | 14/19 | 2 | 0.109 |
| BRENTANI\_CELL\_ADHESION (c2) Cancer related genes involved in cell adhesion and metalloproteinases | 6.35375e-09 | 87/93 | 11 | 2.487 |
| PHOSPHOPROTEIN\_PHOSPHATASE\_ACTIVITY (c5) Genes annotated by the GO term GO:0004721. Catalysis of the reaction: a phosphoprotein + H2O = a protein + phosphate. Together with protein kinases, these enzymes control the state of phosphorylation of cell proteins and thereby provide an important mechanism for regulating cellular activity. | 1.73027e-08 | 72/81 | 10 | 2.166 |
| CELL\_ADHESION (c2) The attachment of a cell, either to another cell or to the extracellular matrix, via cell adhesion molecules. | 2.72369e-08 | 142/174 | 12 | 3.015 |
| MEMBRANE\_PART (c5) Genes annotated by the GO term GO:0044425. Any constituent part of a membrane, a double layer of lipid molecules that encloses all cells, and, in eukaryotes, many organelles; may be a single or double lipid bilayer; also includes associated proteins. | 6.34606e-08 | 1170/1661 | 38 | 17.974 |
| HSA04514\_CELL\_ADHESION\_MOLECULES (c2) Genes involved in cell adhesion molecules (CAMs) | 1.20692e-07 | 116/133 | 9 | 1.933 |
| HSA05213\_ENDOMETRIAL\_CANCER (c2) Genes involved in endometrial cancer | 1.34252e-07 | 50/52 | 15 | 4.877 |
| WNTPATHWAY (c2) The Wnt glycoprotein binds to membrane-bound receptors such as Frizzled to activate a number of signaling pathways, including that of beta-catenin. | 1.35876e-07 | 22/24 | 10 | 2.64 |
| PHOSPHORIC\_ESTER\_HYDROLASE\_ACTIVITY (c5) Genes annotated by the GO term GO:0042578. Catalysis of the reaction: RPO-R' + H2O = RPOOH + R'H. This reaction is the hydrolysis of any phosphoric ester bond, any ester formed from orthophosphoric acid, O=P(OH)3. | 1.37444e-07 | 119/151 | 11 | 2.723 |
| ZHAN\_PCS\_MULTIPLE\_MYELOMA\_SPKD (c2) Genes with spiked expression in subsets of MM PCs from newly diagnosed patients | 1.83808e-07 | 18/22 | 3 | 0.306 |
| PITX2PATHWAY (c2) The bicoid-related transcription factor Pitx2 is activated by Wnt binding to the Frizzled receptor and induces tissue-specific cell proliferation. | 2.30158e-07 | 14/15 | 8 | 2.054 |
| CARBONATE\_DEHYDRATASE\_ACTIVITY (c5) Genes annotated by the GO term GO:0004089. Catalysis of the reaction: H2CO3 = CO2 + H2O. | 2.46604e-07 | 5/13 | 1 | 0.036 |
| RIBOFLAVIN\_METABOLISM (c2) | 2.4697e-07 | 7/10 | 1 | 0.038 |
| LIZUKA\_G2\_GR\_G3 (c2) Genes highly expressed in moderately differentiated vs. poorly differentiated hepatocellular carcinoma | 2.68408e-07 | 15/24 | 2 | 0.139 |
| ACTIN\_FILAMENT\_BUNDLE\_FORMATION (c5) Genes annotated by the GO term GO:0051017. The assembly of actin filament bundles; actin filaments are on the same axis but may be oriented with the same or opposite polarities and may be packed with different levels of tightness. | 3.38703e-07 | 10/13 | 3 | 0.289 |
| ABBUD\_LIF\_GH3\_DN (c2) Genes that decreased after LIF treatment of GH3 cells | 3.4981e-07 | 4/5 | 1 | 0.037 |
| CELL\_RECOGNITION (c5) Genes annotated by the GO term GO:0008037. The process by which a cell in a multicellular organism interprets its surroundings. | 3.99948e-07 | 11/18 | 2 | 0.151 |
| INTEGRAL\_TO\_MEMBRANE (c5) Genes annotated by the GO term GO:0016021. Penetrating at least one phospholipid bilayer of a membrane. May also refer to the state of being buried in the bilayer with no exposure outside the bilayer. When used to describe a protein, indicates that all or part of the peptide sequence is embedded in the membrane. | 7.90521e-07 | 923/1325 | 28 | 12.356 |
| PLASMA\_MEMBRANE (c5) Genes annotated by the GO term GO:0005886. The membrane surrounding a cell that separates the cell from its external environment. It consists of a phospholipid bilayer and associated proteins. | 8.44674e-07 | 1068/1421 | 39 | 20.105 |
| INTRINSIC\_TO\_MEMBRANE (c5) Genes annotated by the GO term GO:0031224. Located in a membrane such that some covalently attached portion of the gene product, for example part of a peptide sequence or some other covalently attached moiety such as a GPI anchor, spans or is embedded in one or both leaflets of the membrane. | 8.66781e-07 | 936/1343 | 28 | 12.454 |
| BRENTANI\_ANGIOGENESIS (c2) Cancer related genes involved in angiogenesis | 9.4439e-07 | 7/9 | 3 | 0.338 |
| WNT\_TARGETS (c2) WNT target genes from literatures | 1.06041e-06 | 20/22 | 5 | 0.868 |
| MEMBRANE (c5) Genes annotated by the GO term GO:0016020. Double layer of lipid molecules that encloses all cells, and, in eukaryotes, many organelles; may be a single or double lipid bilayer; also includes associated proteins. | 1.20384e-06 | 1426/1981 | 47 | 25.682 |
| chr12p11 (c1) Genes in cytogenetic band chr12p11 | 1.51946e-06 | 17/30 | 2 | 0.147 |
| HSA04340\_HEDGEHOG\_SIGNALING\_PATHWAY (c2) Genes involved in Hedgehog signaling pathway | 1.90432e-06 | 43/57 | 6 | 1.217 |
| chr11p1 (c1) Genes in cytogenetic band chr11p1 | 2.0699e-06 | 0/1 | 1 | 0.045 |
| chr16q22 (c1) Genes in cytogenetic band chr16q22 | 2.35906e-06 | 62/120 | 5 | 0.875 |
| CELL\_ADHESION\_MOLECULE\_ACTIVITY (c2) Obsolete by GO - mediates the adhesion of the cell to other cells or to the extracellular matrix. | 2.71703e-06 | 83/108 | 7 | 1.467 |
| GLIOGENESIS (c5) Genes annotated by the GO term GO:0042063. The process by which glial cells are generated. This includes the production of glial progenitors and their differentiation into mature glia. | 3.95928e-06 | 10/11 | 2 | 0.173 |
| PLASMA\_MEMBRANE\_PART (c5) Genes annotated by the GO term GO:0044459. Any constituent part of the plasma membrane, the membrane surrounding a cell that separates the cell from its external environment. It consists of a phospholipid bilayer and associated proteins. | 4.3863e-06 | 844/1157 | 29 | 13.931 |
| TRANSMEMBRANE\_RECEPTOR\_PROTEIN\_KINASE\_ACTIVITY (c5) Genes annotated by the GO term GO:0019199. | 5.78906e-06 | 46/51 | 9 | 2.516 |
| DFOSB\_BRAIN\_8WKS\_UP (c2) Up-regulated in the nucleus accumbens of mice after 8 weeks of induction of transgenic deltaFosB | 6.2823e-06 | 32/40 | 4 | 0.596 |
| HSA00740\_RIBOFLAVIN\_METABOLISM (c2) Genes involved in riboflavin metabolism | 6.60642e-06 | 11/15 | 1 | 0.05 |
| HEART\_DEVELOPMENT (c5) Genes annotated by the GO term GO:0007507. The process whose specific outcome is the progression of the heart over time, from its formation to the mature structure. The heart is a hollow, muscular organ, which, by contracting rhythmically, keeps up the circulation of the blood. | 6.62973e-06 | 29/37 | 4 | 0.64 |
| BECKER\_CANCER\_ASSOCIATED\_SUBSET\_2 (c2) Genes which are known to be associated with cancer and which are downregulated in MaCa 3366/TAM compared to MaCa 3366 (fold change > 2) | 6.9551e-06 | 10/12 | 3 | 0.391 |
| GSK3PATHWAY (c2) Bacterial lipopolysaccharide activates AKT to promote the survival and activation of macrophages and inhibits Gsk3-beta to promote beta-catenin accumulation in the nucleus. | 7.09774e-06 | 25/26 | 8 | 2.223 |
| RECEPTOR\_ACTIVITY (c5) Genes annotated by the GO term GO:0004872. Combining with an extracellular or intracellular messenger to initiate a change in cell activity. | 7.29726e-06 | 474/583 | 22 | 9.474 |
