# Supplementary material for: Looking at Cerebellar Malformations through Text-Mined Interactomes of Mice and Humans
Source: PLoS Comput Biol. 2009 Nov 6;5(11):e1000559. doi: 10.1371/journal.pcbi.1000559 (PMC2767227; doi:10.1371/journal.pcbi.1000559)
Supplement: Dataset S1 — All enrichment results. (0.20 MB ZIP) [file pcbi.1000559.s012.zip › enrichment_results/Table D. enrichment_hprd-all.html]

Complete Clustering results for network hprd and phenotype all (FDR <= 0.001)


# Complete Clustering results for network hprd and phenotype all (FDR <= 0.001)

| Set | p-Value | Gene Count | Interaction Count | Expected Interection Count |
| --- | --- | --- | --- | --- |
| KINASE\_ACTIVITY (c5) Genes annotated by the GO term GO:0016301. Catalysis of the transfer of a phosphate group, usually from ATP, to a substrate molecule. | 7.13063e-12 | 318/363 | 319 | 225.831 |
| HSA00600\_SPHINGOLIPID\_METABOLISM (c2) Genes involved in sphingolipid metabolism | 1.07825e-11 | 21/38 | 11 | 1.907 |
| TRANSFERASE\_ACTIVITY\_\_TRANSFERRING\_PHOSPHORUS\_CONTAINING\_GROUPS (c5) Genes annotated by the GO term GO:0016772. Catalysis of the transfer of a phosphorus-containing group from one compound (donor) to another (acceptor). | 3.74364e-11 | 358/418 | 326 | 234.72 |
| HSA04012\_ERBB\_SIGNALING\_PATHWAY (c2) Genes involved in ErbB signaling pathway | 4.94303e-11 | 85/87 | 226 | 152.248 |
| HSA05010\_ALZHEIMERS\_DISEASE (c2) Genes involved in Alzheimer's disease | 7.03676e-11 | 27/28 | 67 | 32.078 |
| TRANSMEMBRANE\_RECEPTOR\_PROTEIN\_TYROSINE\_KINASE\_SIGNALING\_PATHWAY (c5) Genes annotated by the GO term GO:0007169. The series of molecular signals generated as a consequence of a transmembrane receptor tyrosine kinase binding to its physiological ligand. | 8.00208e-11 | 78/83 | 156 | 98.791 |
| ST\_INTEGRIN\_SIGNALING\_PATHWAY (c2) Integrins are transmembrane receptors that mediate cell growth, survival, and migration by binding to ligands in the extracellular matrix. | 8.71639e-11 | 76/79 | 171 | 110.294 |
| PHOSPHOTRANSFERASE\_ACTIVITY\_\_ALCOHOL\_GROUP\_AS\_ACCEPTOR (c5) Genes annotated by the GO term GO:0016773. Catalysis of the transfer of a phosphorus-containing group from one compound (donor) to an alcohol group (acceptor). | 1.15573e-10 | 293/329 | 299 | 214.178 |
| PROTEIN\_KINASE\_ACTIVITY (c5) Genes annotated by the GO term GO:0004672. Catalysis of the phosphorylation of an amino acid residue in a protein, usually according to the reaction: a protein + ATP = a phosphoprotein + ADP. | 4.03647e-10 | 259/280 | 286 | 205.479 |
| HSA04115\_P53\_SIGNALING\_PATHWAY (c2) Genes involved in p53 signaling pathway | 5.63798e-10 | 58/66 | 116 | 67.664 |
| chr7q36 (c1) Genes in cytogenetic band chr7q36 | 1.6874e-09 | 21/68 | 18 | 5.145 |
| REELINPATHWAY (c2) Reelin is secreted by neurons and recognized by receptors including cadherin related neuronal receptors, which promote phosphorylation of Dab1. | 5.3049e-09 | 6/7 | 28 | 10.707 |
| SYNAPSE (c5) Genes annotated by the GO term GO:0045202. The junction between a nerve fiber of one neuron and another neuron or muscle fiber or glial cell; the site of interneuronal communication. As the nerve fiber approaches the synapse it enlarges into a specialized structure, the presynaptic nerve ending, which contains mitochondria and synaptic vesicles. At the tip of the nerve ending is the presynaptic membrane; facing it, and separated from it by a minute cleft (the synaptic cleft) is a specialized area of membrane on the receiving cell, known as the postsynaptic membrane. In response to the arrival of nerve impulses, the presynaptic nerve ending secretes molecules of neurotransmitters into the synaptic cleft. These diffuse across the cleft and transmit the signal to the postsynaptic membrane. | 6.93869e-09 | 24/27 | 39 | 16.993 |
| MAPKKK\_CASCADE\_GO\_0000165 (c5) Genes annotated by the GO term GO:0000165. Cascade of at least three protein kinase activities culminating in the phosphorylation and activation of a MAP kinase. MAPKKK cascades lie downstream of numerous signaling pathways. | 1.00109e-08 | 95/102 | 99 | 58.681 |
| NERVOUS\_SYSTEM\_DEVELOPMENT (c5) Genes annotated by the GO term GO:0007399. The process whose specific outcome is the progression of nervous tissue over time, from its formation to its mature state. | 1.48003e-08 | 281/382 | 194 | 135.304 |
| module\_274 (c4) Genes in module\_274 | 1.56327e-08 | 73/82 | 67 | 36.485 |
| module\_66 (c4) Genes in module\_66 | 1.77023e-08 | 420/543 | 231 | 165.005 |
| GROWTH\_CONE (c5) Genes annotated by the GO term GO:0030426. The migrating motile tip of a growing nerve cell axon or dendrite. | 1.89538e-08 | 9/10 | 20 | 6.299 |
| ASTON\_DEPRESSION\_DN (c2) Genes downregulated in major depressive disorder (p < 0.05, fold change > 1.4, mean average difference > 150 in at least one of the groups, called present in greater than 20% of all samples) | 2.0935e-08 | 104/140 | 100 | 59.482 |
| GLYCOSPHINGOLIPID\_METABOLISM (c2) | 3.10353e-08 | 16/22 | 8 | 1.525 |
| module\_100 (c4) Genes in module\_100 | 7.16339e-08 | 413/536 | 225 | 162.291 |
| module\_137 (c4) Genes in module\_137 | 7.96573e-08 | 413/539 | 226 | 163.225 |
| SA\_REG\_CASCADE\_OF\_CYCLIN\_EXPR (c2) Expression of cyclins regulates progression through the cell cycle by activating cyclin-dependent kinases. | 8.33334e-08 | 11/13 | 36 | 16.187 |
| FOSBPATHWAY (c2) FOSB gene expression and drug abuse | 9.96433e-08 | 4/5 | 13 | 3.635 |
| HSA05214\_GLIOMA (c2) Genes involved in glioma | 1.42249e-07 | 61/64 | 174 | 123.128 |
| HSA04340\_HEDGEHOG\_SIGNALING\_PATHWAY (c2) Genes involved in Hedgehog signaling pathway | 1.70683e-07 | 43/57 | 47 | 23.619 |
| ABRAHAM\_AL\_VS\_MM\_DN (c2) Genes with significantly lower average gene expression in AL plasma cells than in MM cells | 2.0148e-07 | 17/18 | 57 | 30.29 |
| CELL\_CYCLE\_GO\_0007049 (c5) Genes annotated by the GO term GO:0007049. The progression of biochemical and morphological phases and events that occur in a cell during successive cell replication or nuclear replication events. Canonically, the cell cycle comprises the replication and segregation of genetic material followed by the division of the cell, but in endocycles or syncytial cells nuclear replication or nuclear division may not be followed by cell division. | 2.26446e-07 | 282/311 | 231 | 170.451 |
| GNF2\_SPRR1B (c4) Neighborhood of SPRR1B | 2.4802e-07 | 17/24 | 12 | 3.141 |
| SITE\_OF\_POLARIZED\_GROWTH (c5) Genes annotated by the GO term GO:0030427. Any part of a cell where non-isotropic growth takes place. | 2.95604e-07 | 10/11 | 21 | 7.56 |
| CELLCYCLEPATHWAY (c2) Cyclins interact with cyclin-dependent kinases to form active kinase complexes that regulate progression through the cell cycle. | 3.41344e-07 | 22/23 | 63 | 34.631 |
| G1PATHWAY (c2) CDK4/6-cyclin D and CDK2-cyclin E phosphorylate Rb, which allows the transcription of genes needed for the G1/S cell cycle transition. | 3.82142e-07 | 25/26 | 89 | 54.768 |
| EPIDERMAL\_GROWTH\_FACTOR\_RECEPTOR\_SIGNALING\_PATHWAY (c5) Genes annotated by the GO term GO:0007173. The series of molecular signals generated as a consequence of an epidermal growth factor receptor binding to one of its physiological ligands. | 3.97721e-07 | 20/22 | 61 | 34.68 |
| module\_11 (c4) Genes in module\_11 | 4.43462e-07 | 407/533 | 221 | 161.654 |
| module\_12 (c4) Genes in module\_12 | 4.59588e-07 | 280/354 | 182 | 129.487 |
| P35ALZHEIMERSPATHWAY (c2) p35, a neuron-specific activator of cyclin-dependent kinase 5, is cleaved to p25 in Alzheimer's disease and promotoes hyperphosphorylated tau formation and apoptosis. | 4.77874e-07 | 10/11 | 35 | 16.603 |
| RESPONSE\_TO\_DNA\_DAMAGE\_STIMULUS (c5) Genes annotated by the GO term GO:0006974. A change in state or activity of a cell or an organism (in terms of movement, secretion, enzyme production, gene expression, etc.) as a result of a stimulus indicating damage to its DNA from environmental insults or errors during metabolism. | 6.20744e-07 | 148/161 | 136 | 93.588 |
| EGF\_RECEPTOR\_SIGNALING\_PATHWAY (c2) EDF receptor signaling pathway | 6.45732e-07 | 12/13 | 48 | 25.855 |
| LEARNING\_AND\_OR\_MEMORY (c5) Genes annotated by the GO term GO:0007611. The acquisition and processing of information and/or the storage and retrieval of this information over time. | 8.00487e-07 | 10/14 | 29 | 12.658 |
| HSA05223\_NON\_SMALL\_CELL\_LUNG\_CANCER (c2) Genes involved in non-small cell lung cancer | 8.4066e-07 | 53/54 | 154 | 109.224 |
| CELL\_SOMA (c5) Genes annotated by the GO term GO:0043025. The portion of a cell bearing surface projections such as axons, dendrites, cilia, or flagella that includes the nucleus, but excludes all cell projections. | 8.51506e-07 | 9/10 | 14 | 4.24 |
| CAGGTG\_V$E12\_Q6 (c3) Genes with promoter regions [-2kb,2kb] around transcription start site containing the motif CAGGTG which matches annotation for TCF3: transcription factor 3 (E2A immunoglobulin enhancer binding factors E12/E47) | 9.01714e-07 | 1278/1832 | 626 | 532.844 |
| PROTEIN\_SERINE\_THREONINE\_KINASE\_ACTIVITY (c5) Genes annotated by the GO term GO:0004674. Catalysis of the reaction: ATP + a protein serine/threonine = ADP + protein serine/threonine phosphate. | 9.53306e-07 | 187/201 | 197 | 144.369 |
| module\_2 (c4) Genes in module\_2 | 1.02917e-06 | 301/381 | 191 | 137.351 |
| RESPONSE\_TO\_ENDOGENOUS\_STIMULUS (c5) Genes annotated by the GO term GO:0009719. A change in state or activity of a cell or an organism (in terms of movement, secretion, enzyme production, gene expression, etc.) as a result of an endogenous stimulus. | 1.14449e-06 | 177/198 | 149 | 105.171 |
| ASTON\_OLIGODENDROGLIA\_MYELINATION\_SUBSET (c2) Oligodendroglia/myelination related genes which are downregulated in major depressive disorder (p < 0.05, fold change > 1.4, mean average difference > 150 in at least one of the groups, called present in greater than 20% of all samples) | 1.22173e-06 | 11/17 | 13 | 4.148 |
| V$E47\_01 (c3) Genes with promoter regions [-2kb,2kb] around transcription start site containing the motif VSNGCAGGTGKNCNN which matches annotation for TCF3: transcription factor 3 (E2A immunoglobulin enhancer binding factors E12/E47) | 1.30111e-06 | 148/200 | 107 | 69.639 |
| HSA01510\_NEURODEGENERATIVE\_DISEASES (c2) Genes involved in neurodegenerative diseases | 1.32165e-06 | 34/38 | 111 | 74.3 |
| PROTEIN\_MODIFICATION\_PROCESS (c5) Genes annotated by the GO term GO:0006464. The covalent alteration of one or more amino acids occurring in proteins, peptides and nascent polypeptides (co-translational, post-translational modifications). Includes the modification of charged tRNAs that are destined to occur in a protein (pre-translation modification). | 1.34396e-06 | 521/623 | 409 | 332.306 |
| DENDRITE (c5) Genes annotated by the GO term GO:0030425. A branching protoplasmic process of a neuron that receive and integrate signals coming from axons of other neurons, and convey the resulting signal to the body of the cell. | 1.41288e-06 | 14/16 | 22 | 8.403 |
| V$IK3\_01 (c3) Genes with promoter regions [-2kb,2kb] around transcription start site containing motif TNYTGGGAATACC. Motif does not match any known transcription factor | 1.51938e-06 | 124/169 | 68 | 39.475 |
| PARP\_KO\_DN (c2) Downregulated in MEF cells from PARP knockout mice | 1.55695e-06 | 11/14 | 19 | 6.935 |
| INTEGRIN\_MEDIATED\_CELL\_ADHESION\_KEGG (c2) | 1.56803e-06 | 80/90 | 142 | 99.434 |
| AXON (c5) Genes annotated by the GO term GO:0030424. The long process of a neuron that conducts nerve impulses, usually away from the cell body to the terminals and varicosities, which are sites of storage and release of neurotransmitter. | 1.83665e-06 | 10/12 | 19 | 7.081 |
| CELL\_PROLIFERATION\_GO\_0008283 (c5) Genes annotated by the GO term GO:0008283. The multiplication or reproduction of cells, resulting in the expansion of a cell population. | 1.85259e-06 | 443/513 | 301 | 234.952 |
| CYSTEINE\_TYPE\_PEPTIDASE\_ACTIVITY (c5) Genes annotated by the GO term GO:0008234. Catalysis of the hydrolysis of peptide linkages in oligopeptides or polypeptides; a cysteine residue is at the active center. | 1.98507e-06 | 40/54 | 46 | 24.355 |
| HSA04510\_FOCAL\_ADHESION (c2) Genes involved in focal adhesion | 2.20313e-06 | 179/192 | 283 | 222.596 |
| MORF\_MYL3 (c4) Neighborhood of MYL3 | 2.74314e-06 | 44/69 | 27 | 11.593 |
| BIOPOLYMER\_MODIFICATION (c5) Genes annotated by the GO term GO:0043412. The covalent alteration of one or more monomeric units in a polypeptide, polynucleotide, polysaccharide, or other biological polymer, resulting in a change in its properties. | 3.28637e-06 | 538/642 | 415 | 340.625 |
| POST\_TRANSLATIONAL\_PROTEIN\_MODIFICATION (c5) Genes annotated by the GO term GO:0043687. The covalent alteration of one or more amino acids occurring in a protein after the protein has been completely translated and released from the ribosome. | 3.67906e-06 | 419/470 | 368 | 297.876 |
| NEURON\_DEVELOPMENT (c5) Genes annotated by the GO term GO:0048666. The process whose specific outcome is the progression of a neuron over time, from initial commitment of the cell to a specific fate, to the fully functional differentiated cell. | 4.83843e-06 | 55/61 | 51 | 28.315 |
| FASPATHWAY (c2) Binding of the Fas ligand to the Fas receptor induces caspase activation and consequent apoptosis in the Fas-expressing cell. | 5.63765e-06 | 25/27 | 72 | 44.524 |
| INTEGRIN\_COMPLEX (c5) Genes annotated by the GO term GO:0008305. Any member of a family of heterodimeric transmembrane receptors for cell-adhesion molecules. The alpha and beta subunits are noncovalently bonded. | 6.60066e-06 | 18/19 | 20 | 8.009 |
| REGULATION\_OF\_NEURON\_APOPTOSIS (c5) Genes annotated by the GO term GO:0043523. Any process that modulates the occurrence or rate of cell death by apoptosis in neurons. | 6.68088e-06 | 9/12 | 20 | 7.984 |
| RTTTNNNYTGGM\_UNKNOWN (c3) Genes with promoter regions [-2kb,2kb] around transcription start site containing motif RTTTNNNYTGGM. Motif does not match any known transcription factor | 7.0353e-06 | 86/120 | 65 | 39.889 |
| CELLULAR\_PROTEIN\_METABOLIC\_PROCESS (c5) Genes annotated by the GO term GO:0044267. The chemical reactions and pathways involving a specific protein, rather than of proteins in general, occurring at the level of an individual cell. Includes protein modification. | 7.64987e-06 | 887/1100 | 580 | 494.149 |
| S\_PHASE\_OF\_MITOTIC\_CELL\_CYCLE (c5) Genes annotated by the GO term GO:0000084. Progression through S phase, the part of the mitotic cell cycle during which DNA synthesis takes place. | 8.07401e-06 | 9/10 | 17 | 6.74 |
| NEURITE\_DEVELOPMENT (c5) Genes annotated by the GO term GO:0031175. The process whose specific outcome is the progression of the neurite over time, from its formation to the mature structure. The neurite is any process extending from a neural cell, such as axons or dendrites. | 8.41123e-06 | 48/53 | 46 | 25.339 |
| module\_220 (c4) Genes in module\_220 | 8.83846e-06 | 283/329 | 163 | 121.178 |
| CELLULAR\_MACROMOLECULE\_METABOLIC\_PROCESS (c5) Genes annotated by the GO term GO:0044260. The chemical reactions and pathways involving macromolecules, large molecules including proteins, nucleic acids and carbohydrates, as carried out by individual cells. | 9.10517e-06 | 895/1114 | 582 | 496.637 |
| POSITIVE\_REGULATION\_OF\_ANGIOGENESIS (c5) Genes annotated by the GO term GO:0045766. Any process that activates or increases angiogenesis. | 1.03998e-05 | 8/10 | 9 | 2.448 |
| TRANSMEMBRANE\_RECEPTOR\_PROTEIN\_TYROSINE\_KINASE\_ACTIVITY (c5) Genes annotated by the GO term GO:0004714. Catalysis of the reaction: ATP + a protein-L-tyrosine = ADP + a protein-L-tyrosine phosphate, to initiate a change in cell activity. | 1.05101e-05 | 38/43 | 61 | 36.158 |
| HSA01032\_GLYCAN\_STRUCTURES\_DEGRADATION (c2) Genes involved in degradation of glycan structures | 1.07357e-05 | 13/29 | 5 | 0.971 |
| SIGNAL\_TRANSDUCTION (c5) Genes annotated by the GO term GO:0007165. The cascade of processes by which a signal interacts with a receptor, causing a change in the level or activity of a second messenger or other downstream target, and ultimately effecting a change in the functioning of the cell. | 1.16156e-05 | 1416/1625 | 906 | 808.541 |
| NEURON\_PROJECTION (c5) Genes annotated by the GO term GO:0043005. A prolongation or process extending from a nerve cell, e.g. an axon or dendrite. | 1.31672e-05 | 17/20 | 22 | 9.565 |
