# Supplementary material for: Looking at Cerebellar Malformations through Text-Mined Interactomes of Mice and Humans
Source: PLoS Comput Biol. 2009 Nov 6;5(11):e1000559. doi: 10.1371/journal.pcbi.1000559 (PMC2767227; doi:10.1371/journal.pcbi.1000559)
Supplement: Dataset S1 — All enrichment results. (0.20 MB ZIP) [file pcbi.1000559.s012.zip › enrichment_results/Table E. enrichment_hprd-ataxia.html]

Complete Clustering results for network hprd and phenotype ataxia (FDR <= 0.001)


# Complete Clustering results for network hprd and phenotype ataxia (FDR <= 0.001)

| Set | p-Value | Gene Count | Interaction Count | Expected Interection Count |
| --- | --- | --- | --- | --- |
| KINASE\_ACTIVITY (c5) Genes annotated by the GO term GO:0016301. Catalysis of the transfer of a phosphate group, usually from ATP, to a substrate molecule. | 2.22045e-16 | 318/363 | 242 | 149.994 |
| TRANSFERASE\_ACTIVITY\_\_TRANSFERRING\_PHOSPHORUS\_CONTAINING\_GROUPS (c5) Genes annotated by the GO term GO:0016772. Catalysis of the transfer of a phosphorus-containing group from one compound (donor) to another (acceptor). | 9.99201e-16 | 358/418 | 247 | 155.914 |
| PHOSPHOTRANSFERASE\_ACTIVITY\_\_ALCOHOL\_GROUP\_AS\_ACCEPTOR (c5) Genes annotated by the GO term GO:0016773. Catalysis of the transfer of a phosphorus-containing group from one compound (donor) to an alcohol group (acceptor). | 4.94049e-14 | 293/329 | 224 | 142.474 |
| PROTEIN\_KINASE\_ACTIVITY (c5) Genes annotated by the GO term GO:0004672. Catalysis of the phosphorylation of an amino acid residue in a protein, usually according to the reaction: a protein + ATP = a phosphoprotein + ADP. | 1.15019e-13 | 259/280 | 215 | 136.511 |
| HSA05010\_ALZHEIMERS\_DISEASE (c2) Genes involved in Alzheimer's disease | 7.47669e-12 | 27/28 | 51 | 21.17 |
| LEARNING\_AND\_OR\_MEMORY (c5) Genes annotated by the GO term GO:0007611. The acquisition and processing of information and/or the storage and retrieval of this information over time. | 4.5745e-11 | 10/14 | 26 | 8.346 |
| module\_274 (c4) Genes in module\_274 | 1.81719e-10 | 73/82 | 54 | 24.063 |
| PROTEIN\_SERINE\_THREONINE\_KINASE\_ACTIVITY (c5) Genes annotated by the GO term GO:0004674. Catalysis of the reaction: ATP + a protein serine/threonine = ADP + protein serine/threonine phosphate. | 3.61613e-10 | 187/201 | 151 | 95.039 |
| SYNAPSE (c5) Genes annotated by the GO term GO:0045202. The junction between a nerve fiber of one neuron and another neuron or muscle fiber or glial cell; the site of interneuronal communication. As the nerve fiber approaches the synapse it enlarges into a specialized structure, the presynaptic nerve ending, which contains mitochondria and synaptic vesicles. At the tip of the nerve ending is the presynaptic membrane; facing it, and separated from it by a minute cleft (the synaptic cleft) is a specialized area of membrane on the receiving cell, known as the postsynaptic membrane. In response to the arrival of nerve impulses, the presynaptic nerve ending secretes molecules of neurotransmitters into the synaptic cleft. These diffuse across the cleft and transmit the signal to the postsynaptic membrane. | 3.73212e-10 | 24/27 | 31 | 11.173 |
| HSA00600\_SPHINGOLIPID\_METABOLISM (c2) Genes involved in sphingolipid metabolism | 5.91038e-10 | 21/38 | 8 | 1.228 |
| GLYCOSPHINGOLIPID\_METABOLISM (c2) | 1.83353e-09 | 16/22 | 7 | 0.982 |
| NERVOUS\_SYSTEM\_DEVELOPMENT (c5) Genes annotated by the GO term GO:0007399. The process whose specific outcome is the progression of nervous tissue over time, from its formation to its mature state. | 1.87855e-09 | 281/382 | 140 | 89.361 |
| ST\_INTEGRIN\_SIGNALING\_PATHWAY (c2) Integrins are transmembrane receptors that mediate cell growth, survival, and migration by binding to ligands in the extracellular matrix. | 2.13615e-09 | 76/79 | 119 | 72.363 |
| MAP\_KINASE\_ACTIVITY (c5) Genes annotated by the GO term GO:0004707. Catalysis of the phosphorylation of proteins. Mitogen-activated protein kinase; a family of protein kinases that perform a crucial step in relaying signals from the plasma membrane to the nucleus. They are activated by a wide range of proliferation- or differentiation-inducing signals; activation is strong with agonists such as polypeptide growth factors and tumor-promoting phorbol esters, but weak (in most cell backgrounds) by stress stimuli. | 4.24714e-09 | 11/12 | 31 | 12.002 |
| SA\_REG\_CASCADE\_OF\_CYCLIN\_EXPR (c2) Expression of cyclins regulates progression through the cell cycle by activating cyclin-dependent kinases. | 6.78764e-09 | 11/13 | 28 | 10.429 |
| HSA01032\_GLYCAN\_STRUCTURES\_DEGRADATION (c2) Genes involved in degradation of glycan structures | 7.45468e-09 | 13/29 | 5 | 0.652 |
| MAPKKK\_CASCADE\_GO\_0000165 (c5) Genes annotated by the GO term GO:0000165. Cascade of at least three protein kinase activities culminating in the phosphorylation and activation of a MAP kinase. MAPKKK cascades lie downstream of numerous signaling pathways. | 1.1665e-08 | 95/102 | 71 | 38.535 |
| N\_GLYCAN\_DEGRADATION (c2) | 1.69624e-08 | 9/13 | 4 | 0.455 |
| ASTON\_DEPRESSION\_DN (c2) Genes downregulated in major depressive disorder (p < 0.05, fold change > 1.4, mean average difference > 150 in at least one of the groups, called present in greater than 20% of all samples) | 2.04217e-08 | 104/140 | 71 | 39.177 |
| module\_235 (c4) Genes in module\_235 | 4.11217e-08 | 51/81 | 26 | 10.302 |
| STRESS\_ACTIVATED\_PROTEIN\_KINASE\_SIGNALING\_PATHWAY (c5) Genes annotated by the GO term GO:0031098. A series of molecular signals in which a stress-activated protein kinase (SAPK) cascade relays one or more of the signals. | 6.58392e-08 | 44/47 | 42 | 20.05 |
| HSA04115\_P53\_SIGNALING\_PATHWAY (c2) Genes involved in p53 signaling pathway | 8.23903e-08 | 58/66 | 78 | 44.54 |
| HSA00511\_N\_GLYCAN\_DEGRADATION (c2) Genes involved in N-glycan degradation | 8.84444e-08 | 10/15 | 4 | 0.495 |
| INTEGRIN\_COMPLEX (c5) Genes annotated by the GO term GO:0008305. Any member of a family of heterodimeric transmembrane receptors for cell-adhesion molecules. The alpha and beta subunits are noncovalently bonded. | 1.09743e-07 | 18/19 | 17 | 5.356 |
| module\_12 (c4) Genes in module\_12 | 1.33992e-07 | 280/354 | 129 | 85.97 |
| INTRINSIC\_TO\_PLASMA\_MEMBRANE (c5) Genes annotated by the GO term GO:0031226. Located in the plasma membrane such that some covalently attached portion of the gene product, for example part of a peptide sequence or some other covalently attached moiety such as a GPI anchor, spans or is embedded in one or both leaflets of the membrane. | 2.32533e-07 | 704/991 | 209 | 151.325 |
| INTEGRAL\_TO\_PLASMA\_MEMBRANE (c5) Genes annotated by the GO term GO:0005887. Penetrating at least one phospholipid bilayer of a plasma membrane. May also refer to the state of being buried in the bilayer with no exposure outside the bilayer. | 2.75462e-07 | 695/977 | 208 | 150.672 |
| JNK\_CASCADE (c5) Genes annotated by the GO term GO:0007254. A cascade of protein kinase activities, culminating in the phosphorylation and activation of a member of the JUN kinase subfamily of stress-activated protein kinases, which in turn are a subfamily of mitogen-activated protein (MAP) kinases that is activated primarily by cytokines and exposure to environmental stress. | 4.14705e-07 | 43/45 | 40 | 19.613 |
| ASTON\_OLIGODENDROGLIA\_MYELINATION\_SUBSET (c2) Oligodendroglia/myelination related genes which are downregulated in major depressive disorder (p < 0.05, fold change > 1.4, mean average difference > 150 in at least one of the groups, called present in greater than 20% of all samples) | 5.06578e-07 | 11/17 | 11 | 2.883 |
| INNEREAR\_UP (c2) Genes prefentially expressed in human inner ear tissue (cochlea and vestibule), at least 10-fold higher from a mixture of 29 other tissues | 5.11945e-07 | 24/38 | 15 | 4.846 |
| PROTEIN\_MODIFICATION\_PROCESS (c5) Genes annotated by the GO term GO:0006464. The covalent alteration of one or more amino acids occurring in proteins, peptides and nascent polypeptides (co-translational, post-translational modifications). Includes the modification of charged tRNAs that are destined to occur in a protein (pre-translation modification). | 5.41449e-07 | 521/623 | 285 | 219.599 |
| V$IK3\_01 (c3) Genes with promoter regions [-2kb,2kb] around transcription start site containing motif TNYTGGGAATACC. Motif does not match any known transcription factor | 6.39063e-07 | 124/169 | 51 | 26.189 |
| CELLULAR\_PROTEIN\_METABOLIC\_PROCESS (c5) Genes annotated by the GO term GO:0044267. The chemical reactions and pathways involving a specific protein, rather than of proteins in general, occurring at the level of an individual cell. Includes protein modification. | 7.61625e-07 | 887/1100 | 402 | 327.618 |
| CELLULAR\_MACROMOLECULE\_METABOLIC\_PROCESS (c5) Genes annotated by the GO term GO:0044260. The chemical reactions and pathways involving macromolecules, large molecules including proteins, nucleic acids and carbohydrates, as carried out by individual cells. | 7.69135e-07 | 895/1114 | 404 | 329.304 |
| REELINPATHWAY (c2) Reelin is secreted by neurons and recognized by receptors including cadherin related neuronal receptors, which promote phosphorylation of Dab1. | 9.01265e-07 | 6/7 | 19 | 7.231 |
| BIOPOLYMER\_MODIFICATION (c5) Genes annotated by the GO term GO:0043412. The covalent alteration of one or more monomeric units in a polypeptide, polynucleotide, polysaccharide, or other biological polymer, resulting in a change in its properties. | 9.2289e-07 | 538/642 | 290 | 225.287 |
| ENDOTHELIAL\_CELL\_PROLIFERATION (c5) Genes annotated by the GO term GO:0001935. The multiplication or reproduction of endothelial cells, resulting in the expansion of a cell population. Endothelial cells are thin flattened cells which line the inside surfaces of body cavities, blood vessels, and lymph vessels, making up the endothelium. | 9.85548e-07 | 11/12 | 11 | 2.94 |
| PROTEIN\_METABOLIC\_PROCESS (c5) Genes annotated by the GO term GO:0019538. The chemical reactions and pathways involving a specific protein, rather than of proteins in general. Includes protein modification. | 1.06492e-06 | 992/1214 | 455 | 377.392 |
| INTRINSIC\_TO\_MEMBRANE (c5) Genes annotated by the GO term GO:0031224. Located in a membrane such that some covalently attached portion of the gene product, for example part of a peptide sequence or some other covalently attached moiety such as a GPI anchor, spans or is embedded in one or both leaflets of the membrane. | 1.1486e-06 | 936/1343 | 244 | 186.189 |
| module\_236 (c4) Genes in module\_236 | 1.25917e-06 | 14/18 | 13 | 3.972 |
| PHOSPHOTRANSFERASE\_ACTIVITY\_\_PHOSPHATE\_GROUP\_AS\_ACCEPTOR (c5) Genes annotated by the GO term GO:0016776. Catalysis of the transfer of a phosphorus-containing group from one compound (donor) to a phosphate group (acceptor). | 1.37675e-06 | 13/18 | 17 | 5.887 |
| INTEGRAL\_TO\_MEMBRANE (c5) Genes annotated by the GO term GO:0016021. Penetrating at least one phospholipid bilayer of a membrane. May also refer to the state of being buried in the bilayer with no exposure outside the bilayer. When used to describe a protein, indicates that all or part of the peptide sequence is embedded in the membrane. | 1.5114e-06 | 923/1325 | 242 | 184.662 |
| TRANSMEMBRANE\_RECEPTOR\_PROTEIN\_TYROSINE\_KINASE\_ACTIVITY (c5) Genes annotated by the GO term GO:0004714. Catalysis of the reaction: ATP + a protein-L-tyrosine = ADP + a protein-L-tyrosine phosphate, to initiate a change in cell activity. | 2.79673e-06 | 38/43 | 46 | 24.484 |
| module\_51 (c4) Genes in module\_51 | 3.06639e-06 | 33/36 | 37 | 18.755 |
| SYNAPSE\_PART (c5) Genes annotated by the GO term GO:0044456. The junction between a nerve fiber of one neuron and another neuron or muscle fiber or glial cell. | 3.11639e-06 | 11/13 | 17 | 6.105 |
| TRANSMEMBRANE\_RECEPTOR\_PROTEIN\_TYROSINE\_KINASE\_SIGNALING\_PATHWAY (c5) Genes annotated by the GO term GO:0007169. The series of molecular signals generated as a consequence of a transmembrane receptor tyrosine kinase binding to its physiological ligand. | 3.61547e-06 | 78/83 | 99 | 65.808 |
| ST\_GRANULE\_CELL\_SURVIVAL\_PATHWAY (c2) The survival and differentiation of granule cells in the brain is controlled by pro-growth PACAP and pro-apoptotic ceramides. | 4.15052e-06 | 24/26 | 44 | 23.677 |
| HSA04510\_FOCAL\_ADHESION (c2) Genes involved in focal adhesion | 4.21211e-06 | 179/192 | 199 | 148.924 |
| APOPTOSIS\_GO (c5) Genes annotated by the GO term GO:0006915. A form of programmed cell death induced by external or internal signals that trigger the activity of proteolytic caspases, whose actions dismantle the cell and result in cell death. Apoptosis begins internally with condensation and subsequent fragmentation of the cell nucleus (blebbing) while the plasma membrane remains intact. Other characteristics of apoptosis include DNA fragmentation and the exposure of phosphatidyl serine on the cell surface. | 4.29771e-06 | 386/425 | 260 | 202.114 |
| PROGRAMMED\_CELL\_DEATH (c5) Genes annotated by the GO term GO:0012501. Cell death resulting from activation of endogenous cellular processes. | 4.34022e-06 | 387/426 | 260 | 202.165 |
| BIOPOLYMER\_METABOLIC\_PROCESS (c5) Genes annotated by the GO term GO:0043283. The chemical reactions and pathways involving biopolymers, long, repeating chains of monomers found in nature e.g. polysaccharides and proteins. | 4.58171e-06 | 1426/1667 | 593 | 511.642 |
| STATIN\_PATHWAY\_PHARMGKB (c2) | 4.73062e-06 | 13/18 | 14 | 4.51 |
| module\_113 (c4) Genes in module\_113 | 5.40117e-06 | 81/107 | 50 | 28.358 |
| AGED\_MOUSE\_HYPOTH\_DN (c2) Down-regulated in the hypothalamus of aged (22 months) BALB/c mice, compared to young (2 months) controls | 5.58542e-06 | 31/38 | 32 | 15.455 |
| POST\_TRANSLATIONAL\_PROTEIN\_MODIFICATION (c5) Genes annotated by the GO term GO:0043687. The covalent alteration of one or more amino acids occurring in a protein after the protein has been completely translated and released from the ribosome. | 6.15446e-06 | 419/470 | 252 | 197.158 |
| CELL\_DEVELOPMENT (c5) Genes annotated by the GO term GO:0048468. The process whose specific outcome is the progression of the cell over time, from its formation to the mature structure. Cell development does not include the steps involved in committing a cell to a specific fate. | 6.15666e-06 | 510/571 | 309 | 246.804 |
| NUCLEOBASE\_\_NUCLEOSIDE\_\_NUCLEOTIDE\_KINASE\_ACTIVITY (c5) Genes annotated by the GO term GO:0019205. Catalysis of the transfer of a phosphate group, usually from ATP or GTP, to a nucleobase, nucleoside, nucleotide or polynucleotide substrate. | 6.19977e-06 | 17/24 | 18 | 6.838 |
| LIPOPROTEIN\_BINDING (c5) Genes annotated by the GO term GO:0008034. Interacting selectively with any conjugated, water-soluble protein in which the nonprotein moiety consists of a lipid or lipids. | 7.43093e-06 | 14/17 | 13 | 4.298 |
| GROWTH\_CONE (c5) Genes annotated by the GO term GO:0030426. The migrating motile tip of a growing nerve cell axon or dendrite. | 8.3544e-06 | 9/10 | 13 | 4.24 |
| CELLCYCLEPATHWAY (c2) Cyclins interact with cyclin-dependent kinases to form active kinase complexes that regulate progression through the cell cycle. | 9.06161e-06 | 22/23 | 43 | 22.688 |
| GLYCOPROTEIN\_METABOLIC\_PROCESS (c5) Genes annotated by the GO term GO:0009100. The chemical reactions and pathways involving glycoproteins, any protein that contains covalently bound glycose (i.e. monosaccharide) residues other than as a moiety of nucleic acid; the glycose occurs most commonly as oligosaccharide or fairly small polysaccharide but occasionally as monosaccharide. | 9.62859e-06 | 54/89 | 21 | 8.442 |
| module\_66 (c4) Genes in module\_66 | 9.75755e-06 | 420/543 | 153 | 109.969 |
| REGULATION\_OF\_APOPTOSIS (c5) Genes annotated by the GO term GO:0042981. Any process that modulates the occurrence or rate of cell death by apoptosis. | 1.07419e-05 | 310/337 | 214 | 163.278 |
| REGULATION\_OF\_PROGRAMMED\_CELL\_DEATH (c5) Genes annotated by the GO term GO:0043067. Any process that modulates the frequency, rate or extent of programmed cell death, cell death resulting from activation of endogenous cellular processes. | 1.08775e-05 | 311/338 | 214 | 163.329 |
| PHOSPHORYLATION (c5) Genes annotated by the GO term GO:0016310. The process of introducing a phosphate group into a molecule, usually with the formation of a phosphoric ester, a phosphoric anhydride or a phosphoric amide. | 1.20243e-05 | 280/307 | 193 | 146.801 |
