# Supplementary material for: Looking at Cerebellar Malformations through Text-Mined Interactomes of Mice and Humans
Source: PLoS Comput Biol. 2009 Nov 6;5(11):e1000559. doi: 10.1371/journal.pcbi.1000559 (PMC2767227; doi:10.1371/journal.pcbi.1000559)
Supplement: Dataset S1 — All enrichment results. (0.20 MB ZIP) [file pcbi.1000559.s012.zip › enrichment_results/Table F. enrichment_hprd-degeneration.html]

Complete Clustering results for network hprd and phenotype degeneration (FDR <= 0.001)


# Complete Clustering results for network hprd and phenotype degeneration (FDR <= 0.001)

| Set | p-Value | Gene Count | Interaction Count | Expected Interection Count |
| --- | --- | --- | --- | --- |
| GNF2\_SPRR1B (c4) Neighborhood of SPRR1B | 1e-20 | 17/24 | 8 | 0.373 |
| GLYCOSPHINGOLIPID\_METABOLISM (c2) | 1e-20 | 16/22 | 4 | 0.164 |
| HSA00600\_SPHINGOLIPID\_METABOLISM (c2) Genes involved in sphingolipid metabolism | 1e-20 | 21/38 | 6 | 0.211 |
| KERATINOCYTE\_DIFFERENTIATION (c5) Genes annotated by the GO term GO:0030216. The process whereby a relatively unspecialized cell acquires specialized features of a keratinocyte. | 1e-20 | 13/15 | 6 | 0.42 |
| STRUCTURE\_SPECIFIC\_DNA\_BINDING (c5) Genes annotated by the GO term GO:0043566. Interacting selectively with DNA of a specific structure or configuration e.g. triplex DNA binding or bent DNA binding. | 5.74651e-13 | 51/56 | 20 | 4.895 |
| module\_164 (c4) Genes in module\_164 | 8.33666e-13 | 54/61 | 15 | 2.997 |
| BRENTANI\_REPAIR (c2) Cancer related genes involved in DNA repair | 2.33835e-12 | 35/36 | 14 | 2.752 |
| SINGLE\_STRANDED\_DNA\_BINDING (c5) Genes annotated by the GO term GO:0003697. Interacting selectively with single-stranded DNA. | 2.55762e-12 | 33/35 | 14 | 2.909 |
| MEIOTIC\_RECOMBINATION (c5) Genes annotated by the GO term GO:0007131. The cell cycle process whereby double strand breaks are formed and repaired through a double Holliday junction intermediate. This results in the equal exchange of genetic material between non-sister chromatids in a pair of homologous chromosomes. These reciprocal recombinant products ensure the proper segregation of homologous chromosomes during meiosis I and create genetic diversity. | 8.68627e-12 | 14/17 | 7 | 0.832 |
| chr20p11 (c1) Genes in cytogenetic band chr20p11 | 1.34012e-11 | 21/68 | 5 | 0.48 |
| ATRBRCAPATHWAY (c2) BRCA1 and 2 block cell cycle progression in response to DNA damage and promote double-stranded break repair; mutations induce breast cancer susceptibility. | 2.13382e-11 | 20/21 | 15 | 3.238 |
| SPHINGOID\_METABOLIC\_PROCESS (c5) Genes annotated by the GO term GO:0046519. The chemical reactions and pathways involving sphingoids, any of a class of compounds comprising sphinganine and its homologues and stereoisomers, and derivatives of these compounds. | 3.31749e-11 | 5/12 | 2 | 0.083 |
| CORNIFIED\_ENVELOPE (c5) Genes annotated by the GO term GO:0001533. An insoluble protein structure formed under the plasma membrane of cornifying epithelial cells. | 4.96109e-11 | 10/13 | 4 | 0.327 |
| MEIOSIS\_I (c5) Genes annotated by the GO term GO:0007127. Progression through the first phase of meiosis, in which cells divide and homologous chromosomes are paired and segregated from each other, producing two daughter cells. | 7.67932e-11 | 15/19 | 7 | 0.886 |
| DNA\_REPAIR (c5) Genes annotated by the GO term GO:0006281. The process of restoring DNA after damage. Genomes are subject to damage by chemical and physical agents in the environment (e.g. UV and ionizing radiations, chemical mutagens, fungal and bacterial toxins, etc.) and by free radicals or alkylating agents endogenously generated in metabolism. DNA is also damaged because of errors during its replication. A variety of different DNA repair pathways have been reported that include direct reversal, base excision repair, nucleotide excision repair, photoreactivation, bypass, double-strand break repair pathway, and mismatch repair pathway. | 8.24944e-10 | 116/125 | 27 | 9.514 |
| DOUBLE\_STRAND\_BREAK\_REPAIR (c5) Genes annotated by the GO term GO:0006302. The repair of double-strand breaks in DNA via homologous and nonhomologous mechanisms to reform a continuous DNA helix. | 1.13657e-09 | 21/23 | 11 | 2.338 |
| RESPONSE\_TO\_DNA\_DAMAGE\_STIMULUS (c5) Genes annotated by the GO term GO:0006974. A change in state or activity of a cell or an organism (in terms of movement, secretion, enzyme production, gene expression, etc.) as a result of a stimulus indicating damage to its DNA from environmental insults or errors during metabolism. | 1.56975e-09 | 148/161 | 31 | 11.547 |
| HSA01032\_GLYCAN\_STRUCTURES\_DEGRADATION (c2) Genes involved in degradation of glycan structures | 1.91233e-09 | 13/29 | 2 | 0.111 |
| RESPONSE\_TO\_ENDOGENOUS\_STIMULUS (c5) Genes annotated by the GO term GO:0009719. A change in state or activity of a cell or an organism (in terms of movement, secretion, enzyme production, gene expression, etc.) as a result of an endogenous stimulus. | 4.93918e-09 | 177/198 | 33 | 12.944 |
| MEIOTIC\_CELL\_CYCLE (c5) Genes annotated by the GO term GO:0051321. Progression through the phases of the meiotic cell cycle, in which canonically a cell replicates to produce four offspring with half the chromosomal content of the progenitor cell. | 5.96165e-09 | 29/34 | 8 | 1.382 |
| SULFURIC\_ESTER\_HYDROLASE\_ACTIVITY (c5) Genes annotated by the GO term GO:0008484. Catalysis of the reaction: RSO-R' + H2O = RSOOH + R'H. This reaction is the hydrolysis of any sulfuric ester bond, any ester formed from sulfuric acid, O=SO(OH)2. | 1.15091e-08 | 3/16 | 1 | 0.031 |
| ARYLSULFATASE\_ACTIVITY (c5) Genes annotated by the GO term GO:0004065. Catalysis of the reaction: a phenol sulfate + H2O = a phenol + SO4(2-) (sulfate). | 1.15091e-08 | 3/12 | 1 | 0.031 |
| CELL\_CYCLE\_CHECKPOINT (c2) A point in the eukaryotic cell cycle where progress through the cycle can be halted until conditions are suitable for the cell to proceed to the next stage. | 2.24362e-08 | 23/24 | 15 | 4.079 |
| DNA\_RECOMBINATION (c5) Genes annotated by the GO term GO:0006310. The processes by which a new genotype is formed by reassortment of genes resulting in gene combinations different from those that were present in the parents. In eukaryotes genetic recombination can occur by chromosome assortment, intrachromosomal recombination, or nonreciprocal interchromosomal recombination. Intrachromosomal recombination occurs by crossing over. In bacteria it may occur by genetic transformation, conjugation, transduction, or F-duction. | 2.91928e-08 | 41/47 | 12 | 2.971 |
| APPEL\_IMATINIB\_UP (c2) Genes up-regulated by imatinib during dendritic cell differentiation | 3.64896e-08 | 24/31 | 7 | 1.174 |
| HSA00940\_PHENYLPROPANOID\_BIOSYNTHESIS (c2) Genes involved in phenylpropanoid biosynthesis | 4.9865e-08 | 3/7 | 1 | 0.034 |
| H2O2\_CSBRESCUED\_C2\_UP (c2) Upregulated by H2O2 in CSB-rescued fibroblasts (Table 1, cluster 2) | 5.98722e-08 | 7/8 | 3 | 0.277 |
| DNA\_DAMAGE\_CHECKPOINT (c5) Genes annotated by the GO term GO:0000077. A signal transduction pathway, induced by DNA damage, that blocks cell cycle progression (in G1, G2 or metaphase) or slows the rate at which S phase proceeds. | 5.99505e-08 | 15/19 | 7 | 1.163 |
| DNA\_DAMAGE\_SIGNALING (c2) Genes involved in DNA damage signaling | 1.27415e-07 | 84/89 | 23 | 8.418 |
| CASPASEPATHWAY (c2) Caspases are cysteine proteases active in apoptosis; caspase-8 and 9 cleave and activate other caspases, while 3, 6, and 7 cleave cellular targets. | 3.77551e-07 | 20/21 | 11 | 2.817 |
| DEOXYRIBONUCLEASE\_ACTIVITY (c5) Genes annotated by the GO term GO:0004536. Catalysis of the hydrolysis of ester linkages within deoxyribonucleic acid. | 8.00789e-07 | 16/22 | 5 | 0.73 |
| DNA\_METABOLIC\_PROCESS (c5) Genes annotated by the GO term GO:0006259. The chemical reactions and pathways involving DNA, deoxyribonucleic acid, one of the two main types of nucleic acid, consisting of a long, unbranched macromolecule formed from one, or more commonly, two, strands of linked deoxyribonucleotides. | 9.47977e-07 | 228/256 | 34 | 16.575 |
| LIZUKA\_L0\_SM\_L1 (c2) Genes highly expressed in non-tumor liver with hepatitis virus infection vs. liver without hepatitis virus infection. | 1.30183e-06 | 16/19 | 5 | 0.852 |
| DNA\_INTEGRITY\_CHECKPOINT (c5) Genes annotated by the GO term GO:0031570. Any cell cycle checkpoint that delays or arrests cell cycle progression in response to changes in DNA structure. | 1.36313e-06 | 19/23 | 7 | 1.386 |
| CASPASE\_ACTIVITY (c2) Genes related to caspase activity | 1.59757e-06 | 7/8 | 7 | 1.417 |
| RESISTANCE\_XENOGRAFTS\_UP (c2) Up-regulation is correlated with resistance of human cancer xenografts to at least six of seven anticancer drugs tested | 2.39314e-06 | 19/27 | 4 | 0.563 |
| module\_102 (c4) Genes in module\_102 | 2.68973e-06 | 11/18 | 3 | 0.335 |
| chr15q15 (c1) Genes in cytogenetic band chr15q15 | 2.94968e-06 | 33/70 | 6 | 1.183 |
| HSA00460\_CYANOAMINO\_ACID\_METABOLISM (c2) Genes involved in cyanoamino acid metabolism | 4.25646e-06 | 4/6 | 1 | 0.044 |
| LEE\_MYC\_E2F1\_UP (c2) Genes up-regulated in hepatoma tissue of Myc+E2f1 transgenic mice | 5.17823e-06 | 44/54 | 11 | 3.217 |
| PEPIPATHWAY (c2) Proepithelin (PEPI) induces epithelial cells to secrete IL-8, which promotes elastase secretion by neutrophils. | 6.06526e-06 | 4/6 | 3 | 0.366 |
| SODIUM\_CHANNEL\_ACTIVITY (c5) Genes annotated by the GO term GO:0005272. Catalysis of facilitated diffusion of a sodium ion (by an energy-independent process) involving passage through a transmembrane aqueous pore or channel without evidence for a carrier-mediated mechanism. | 6.29058e-06 | 12/17 | 3 | 0.354 |
