# Supplementary material for: Looking at Cerebellar Malformations through Text-Mined Interactomes of Mice and Humans
Source: PLoS Comput Biol. 2009 Nov 6;5(11):e1000559. doi: 10.1371/journal.pcbi.1000559 (PMC2767227; doi:10.1371/journal.pcbi.1000559)
Supplement: Dataset S1 — All enrichment results. (0.20 MB ZIP) [file pcbi.1000559.s012.zip › enrichment_results/Table G. enrichment_hprd-development.html]

Complete Clustering results for network hprd and phenotype development (FDR <= 0.001)


# Complete Clustering results for network hprd and phenotype development (FDR <= 0.001)

| Set | p-Value | Gene Count | Interaction Count | Expected Interection Count |
| --- | --- | --- | --- | --- |
| SA\_REG\_CASCADE\_OF\_CYCLIN\_EXPR (c2) Expression of cyclins regulates progression through the cell cycle by activating cyclin-dependent kinases. | 8.54872e-15 | 11/13 | 23 | 5.592 |
| CELL\_CYCLE\_GO\_0007049 (c5) Genes annotated by the GO term GO:0007049. The progression of biochemical and morphological phases and events that occur in a cell during successive cell replication or nuclear replication events. Canonically, the cell cycle comprises the replication and segregation of genetic material followed by the division of the cell, but in endocycles or syncytial cells nuclear replication or nuclear division may not be followed by cell division. | 4.85167e-14 | 282/311 | 111 | 57.571 |
| NUCLEUS (c5) Genes annotated by the GO term GO:0005634. A membrane-bounded organelle of eukaryotic cells in which chromosomes are housed and replicated. In most cells, the nucleus contains all of the cell's chromosomes except the organellar chromosomes, and is the site of RNA synthesis and processing. In some species, or in specialized cell types, RNA metabolism or DNA replication may be absent. | 4.07785e-13 | 1201/1417 | 320 | 232.044 |
| G1PATHWAY (c2) CDK4/6-cyclin D and CDK2-cyclin E phosphorylate Rb, which allows the transcription of genes needed for the G1/S cell cycle transition. | 6.3094e-13 | 25/26 | 46 | 18.018 |
| BRENTANI\_CELL\_CYCLE (c2) Cancer related genes involved in the cell cycle | 1.72862e-12 | 78/79 | 59 | 25.008 |
| HSA04115\_P53\_SIGNALING\_PATHWAY (c2) Genes involved in p53 signaling pathway | 3.16513e-12 | 58/66 | 54 | 22.658 |
| CELLCYCLEPATHWAY (c2) Cyclins interact with cyclin-dependent kinases to form active kinase complexes that regulate progression through the cell cycle. | 1.06335e-11 | 22/23 | 34 | 11.662 |
| CELL\_CYCLE (c2) The progression of biochemical and morphological events that occur during nuclear or cellular replication. | 4.59438e-11 | 72/76 | 72 | 35.446 |
| RNA\_BIOSYNTHETIC\_PROCESS (c5) Genes annotated by the GO term GO:0032774. The chemical reactions and pathways resulting in the formation of RNA, ribonucleic acid, one of the two main type of nucleic acid, consisting of a long, unbranched macromolecule formed from ribonucleotides joined in 3',5'-phosphodiester linkage. Includes polymerization of ribonucleotide monomers. | 1.21012e-10 | 562/636 | 177 | 117.212 |
| TRANSCRIPTION\_\_DNA\_DEPENDENT (c5) Genes annotated by the GO term GO:0006351. The synthesis of RNA on a template of DNA. | 2.32807e-10 | 561/634 | 176 | 117.183 |
| FOSBPATHWAY (c2) FOSB gene expression and drug abuse | 2.86048e-10 | 4/5 | 8 | 1.249 |
| CELL\_CYCLE\_KEGG (c2) | 3.16284e-10 | 79/84 | 75 | 38.27 |
| RESPONSE\_TO\_DNA\_DAMAGE\_STIMULUS (c5) Genes annotated by the GO term GO:0006974. A change in state or activity of a cell or an organism (in terms of movement, secretion, enzyme production, gene expression, etc.) as a result of a stimulus indicating damage to its DNA from environmental insults or errors during metabolism. | 3.52542e-10 | 148/161 | 63 | 31.922 |
| DNA\_METABOLIC\_PROCESS (c5) Genes annotated by the GO term GO:0006259. The chemical reactions and pathways involving DNA, deoxyribonucleic acid, one of the two main types of nucleic acid, consisting of a long, unbranched macromolecule formed from one, or more commonly, two, strands of linked deoxyribonucleotides. | 5.41267e-10 | 228/256 | 84 | 45.979 |
| G1\_TO\_S\_CELL\_CYCLE\_REACTOME (c2) | 5.94497e-10 | 63/66 | 51 | 22.701 |
| HSA04110\_CELL\_CYCLE (c2) Genes involved in cell cycle | 1.14759e-09 | 106/112 | 101 | 59.717 |
| module\_198 (c4) Genes in module\_198 | 1.39351e-09 | 279/301 | 114 | 68.641 |
| TRANSCRIPTION (c5) Genes annotated by the GO term GO:0006350. The synthesis of either RNA on a template of DNA or DNA on a template of RNA. | 1.45594e-09 | 665/750 | 210 | 147.854 |
| module\_403 (c4) Genes in module\_403 | 2.90283e-09 | 39/46 | 32 | 12.63 |
| RESPONSE\_TO\_ENDOGENOUS\_STIMULUS (c5) Genes annotated by the GO term GO:0009719. A change in state or activity of a cell or an organism (in terms of movement, secretion, enzyme production, gene expression, etc.) as a result of an endogenous stimulus. | 5.12107e-09 | 177/198 | 66 | 35.828 |
| chr7q36 (c1) Genes in cytogenetic band chr7q36 | 6.13335e-09 | 21/68 | 9 | 1.722 |
| module\_197 (c4) Genes in module\_197 | 6.24875e-09 | 148/173 | 67 | 35.099 |
| CELL\_CYCLE\_PROCESS (c5) Genes annotated by the GO term GO:0022402. A cellular process that is involved in the progression of biochemical and morphological phases and events that occur in a cell during successive cell replication or nuclear replication events. | 6.7688e-09 | 173/191 | 65 | 33.942 |
| CELL\_SOMA (c5) Genes annotated by the GO term GO:0043025. The portion of a cell bearing surface projections such as axons, dendrites, cilia, or flagella that includes the nucleus, but excludes all cell projections. | 7.19593e-09 | 9/10 | 8 | 1.4 |
| NUCLEOBASE\_\_NUCLEOSIDE\_\_NUCLEOTIDE\_AND\_NUCLEIC\_ACID\_METABOLIC\_PROCESS (c5) Genes annotated by the GO term GO:0006139. The chemical reactions and pathways involving nucleobases, nucleosides, nucleotides and nucleic acids. | 8.17549e-09 | 1055/1234 | 262 | 196.634 |
| PROLIFERATION\_GENES (c2) Proliferation related genes | 8.97788e-09 | 322/359 | 103 | 61.81 |
| INTEGRIN\_COMPLEX (c5) Genes annotated by the GO term GO:0008305. Any member of a family of heterodimeric transmembrane receptors for cell-adhesion molecules. The alpha and beta subunits are noncovalently bonded. | 1.4904e-08 | 18/19 | 12 | 2.737 |
| V$E2F1\_Q3\_01 (c3) Genes with promoter regions [-2kb,2kb] around transcription start site containing the motif TTGGCGCGRAANNGNM which matches annotation for E2F1: E2F transcription factor 1 | 1.60538e-08 | 149/193 | 48 | 23.191 |
| module\_98 (c4) Genes in module\_98 | 1.77478e-08 | 361/391 | 128 | 82.284 |
| REELINPATHWAY (c2) Reelin is secreted by neurons and recognized by receptors including cadherin related neuronal receptors, which promote phosphorylation of Dab1. | 2.04496e-08 | 6/7 | 14 | 3.714 |
| HSA04340\_HEDGEHOG\_SIGNALING\_PATHWAY (c2) Genes involved in Hedgehog signaling pathway | 2.13006e-08 | 43/57 | 23 | 8.179 |
| CELL\_PROLIFERATION\_GO\_0008283 (c5) Genes annotated by the GO term GO:0008283. The multiplication or reproduction of cells, resulting in the expansion of a cell population. | 2.15098e-08 | 443/513 | 128 | 81.048 |
| PARP\_KO\_DN (c2) Downregulated in MEF cells from PARP knockout mice | 2.35646e-08 | 11/14 | 10 | 2.129 |
| REGULATION\_OF\_METABOLIC\_PROCESS (c5) Genes annotated by the GO term GO:0019222. Any process that modulates the frequency, rate or extent of the chemical reactions and pathways within a cell or an organism. | 2.46088e-08 | 699/794 | 214 | 153.823 |
| P35ALZHEIMERSPATHWAY (c2) p35, a neuron-specific activator of cyclin-dependent kinase 5, is cleaved to p25 in Alzheimer's disease and promotoes hyperphosphorylated tau formation and apoptosis. | 2.469e-08 | 10/11 | 18 | 5.771 |
| BIOPOLYMER\_METABOLIC\_PROCESS (c5) Genes annotated by the GO term GO:0043283. The chemical reactions and pathways involving biopolymers, long, repeating chains of monomers found in nature e.g. polysaccharides and proteins. | 2.92559e-08 | 1426/1667 | 342 | 270.27 |
| DNA\_BINDING (c5) Genes annotated by the GO term GO:0003677. Interacting selectively with DNA (deoxyribonucleic acid). | 2.9675e-08 | 482/600 | 155 | 105.234 |
| REGULATION\_OF\_CELLULAR\_METABOLIC\_PROCESS (c5) Genes annotated by the GO term GO:0031323. Any process that modulates the frequency, rate or extent of the chemical reactions and pathways by which individual cells transform chemical substances. | 3.00672e-08 | 688/782 | 210 | 150.973 |
| DNA\_REPAIR (c5) Genes annotated by the GO term GO:0006281. The process of restoring DNA after damage. Genomes are subject to damage by chemical and physical agents in the environment (e.g. UV and ionizing radiations, chemical mutagens, fungal and bacterial toxins, etc.) and by free radicals or alkylating agents endogenously generated in metabolism. DNA is also damaged because of errors during its replication. A variety of different DNA repair pathways have been reported that include direct reversal, base excision repair, nucleotide excision repair, photoreactivation, bypass, double-strand break repair pathway, and mismatch repair pathway. | 3.12791e-08 | 116/125 | 51 | 25.909 |
| TRANSCRIPTION\_FROM\_RNA\_POLYMERASE\_II\_PROMOTER (c5) Genes annotated by the GO term GO:0006366. The synthesis of RNA from a DNA template by RNA polymerase II (Pol II), originating at a Pol II-specific promoter. Includes transcription of messenger RNA (mRNA) and certain small nuclear RNAs (snRNAs). | 3.45706e-08 | 407/456 | 133 | 87.83 |
| REGULATION\_OF\_NUCLEOBASE\_\_NUCLEOSIDE\_\_NUCLEOTIDE\_AND\_NUCLEIC\_ACID\_METABOLIC\_PROCESS (c5) Genes annotated by the GO term GO:0019219. Any process that modulates the frequency, rate or extent of the chemical reactions and pathways involving nucleobases, nucleosides, nucleotides and nucleic acids. | 4.04457e-08 | 539/614 | 178 | 124.832 |
| BRAIN\_DEVELOPMENT (c5) Genes annotated by the GO term GO:0007420. The process whose specific outcome is the progression of the brain over time, from its formation to the mature structure. The brain is one of the two components of the central nervous system and is the center of thought and emotion. It is responsible for the coordination and control of bodily activities and the interpretation of information from the senses (sight, hearing, smell, etc.). | 4.43619e-08 | 34/51 | 12 | 3.051 |
| CELL\_CYCLE\_PHASE (c5) Genes annotated by the GO term GO:0022403. A cell cycle process comprising the steps by which a cell progresses through one of the biochemical and morphological phases and events that occur during successive cell replication or nuclear replication events. | 5.16028e-08 | 156/169 | 59 | 31.44 |
| REGULATION\_OF\_CELL\_CYCLE (c5) Genes annotated by the GO term GO:0051726. Any process that modulates the rate or extent of progression through the cell cycle. | 5.46859e-08 | 165/180 | 66 | 36.068 |
| module\_57 (c4) Genes in module\_57 | 5.79765e-08 | 53/56 | 52 | 26.486 |
| DNA\_DAMAGE\_SIGNALING (c2) Genes involved in DNA damage signaling | 5.82366e-08 | 84/89 | 47 | 22.598 |
| NEGATIVE\_REGULATION\_OF\_CELLULAR\_PROCESS (c5) Genes annotated by the GO term GO:0048523. Any process that stops, prevents or reduces the frequency, rate or extent of cellular processes, those that are carried out at the cellular level, but are not necessarily restricted to a single cell. For example, cell communication occurs among more than one cell, but occurs at the cellular level. | 7.42507e-08 | 556/640 | 178 | 126.019 |
| DNA\_RECOMBINATION (c5) Genes annotated by the GO term GO:0006310. The processes by which a new genotype is formed by reassortment of genes resulting in gene combinations different from those that were present in the parents. In eukaryotes genetic recombination can occur by chromosome assortment, intrachromosomal recombination, or nonreciprocal interchromosomal recombination. Intrachromosomal recombination occurs by crossing over. In bacteria it may occur by genetic transformation, conjugation, transduction, or F-duction. | 7.57462e-08 | 41/47 | 23 | 8.477 |
| module\_124 (c4) Genes in module\_124 | 1.25274e-07 | 90/96 | 39 | 18.329 |
| module\_308 (c4) Genes in module\_308 | 1.31088e-07 | 52/70 | 19 | 6.53 |
| P53PATHWAY (c2) p53 induces cell cycle arrest or apoptosis under conditions of DNA damage. | 1.4488e-07 | 15/16 | 29 | 12.223 |
| GCAAAAA,MIR-129 (c3) Targets of MicroRNA GCAAAAA,MIR-129 | 1.49417e-07 | 113/166 | 45 | 23.074 |
| ABRAHAM\_AL\_VS\_MM\_DN (c2) Genes with significantly lower average gene expression in AL plasma cells than in MM cells | 1.50019e-07 | 17/18 | 27 | 10.684 |
| REGULATION\_OF\_CYCLIN\_DEPENDENT\_PROTEIN\_KINASE\_ACTIVITY (c5) Genes annotated by the GO term GO:0000079. Any process that modulates the frequency, rate or extent of CDK activity. | 2.25273e-07 | 41/43 | 21 | 7.631 |
| NEGATIVE\_REGULATION\_OF\_BIOLOGICAL\_PROCESS (c5) Genes annotated by the GO term GO:0048519. Any process that stops, prevents or reduces the frequency, rate or extent of a biological process. Biological processes are regulated by many means; examples include the control of gene expression, protein modification or interaction with a protein or substrate molecule. | 2.40152e-07 | 584/670 | 180 | 129.821 |
| P53\_SIGNALING (c2) Genes involved in p53 signaling | 2.5735e-07 | 85/91 | 91 | 56.469 |
| ZHAN\_MULTIPLE\_MYELOMA\_VS\_NORMAL\_UP (c2) The 70 most significantly up-regulated genes in MM in comparison with normal bone marrow PCs | 3.44794e-07 | 47/60 | 24 | 9.373 |
| module\_244 (c4) Genes in module\_244 | 3.769e-07 | 166/186 | 63 | 35.174 |
| P27PATHWAY (c2) p27 blocks the G1/S transition by inhibiting the checkpoint kinase cdk2/cyclin E and is inhibited by cdk2-mediated ubiquitination. | 4.11258e-07 | 11/12 | 17 | 5.77 |
| V$IK3\_01 (c3) Genes with promoter regions [-2kb,2kb] around transcription start site containing motif TNYTGGGAATACC. Motif does not match any known transcription factor | 5.0261e-07 | 124/169 | 30 | 13.27 |
| V$COMP1\_01 (c3) Genes with promoter regions [-2kb,2kb] around transcription start site containing the motif NVTNWTGATTGACNACAAVARRBN which matches annotation for MYOG: myogenin (myogenic factor 4) | 6.37691e-07 | 69/94 | 22 | 8.425 |
| REGULATION\_OF\_TRANSCRIPTION (c5) Genes annotated by the GO term GO:0045449. Any process that modulates the frequency, rate or extent of the synthesis of either RNA on a template of DNA or DNA on a template of RNA. | 8.36955e-07 | 493/563 | 162 | 115.869 |
| SARCOMAS\_SYNOVIAL\_UP (c2) Top 20 positive significant genes associated with synovial sarcomas, versus other soft-tissue tumors. | 8.38229e-07 | 8/12 | 3 | 0.336 |
| GROWTH\_CONE (c5) Genes annotated by the GO term GO:0030426. The migrating motile tip of a growing nerve cell axon or dendrite. | 9.51881e-07 | 9/10 | 9 | 2.079 |
| EMBRYONIC\_MORPHOGENESIS (c5) Genes annotated by the GO term GO:0048598. The process by which anatomical structures are generated and organized during the embryonic phase. Morphogenesis pertains to the creation of form. The embryonic phase begins with zygote formation. The end of the embryonic phase is organism-specific. For example, it would be at birth for mammals, larval hatching for insects and seed dormancy in plants. | 1.0077e-06 | 11/17 | 6 | 1.105 |
| PROTEIN\_DNA\_COMPLEX\_ASSEMBLY (c5) Genes annotated by the GO term GO:0065004. The aggregation, arrangement and bonding together of proteins and DNA molecules to form a protein-DNA complex. | 1.11944e-06 | 44/49 | 24 | 9.733 |
| module\_252 (c4) Genes in module\_252 | 1.13553e-06 | 219/235 | 88 | 55.768 |
| REGULATION\_OF\_TRANSCRIPTION\_\_DNA\_DEPENDENT (c5) Genes annotated by the GO term GO:0006355. Any process that modulates the frequency, rate or extent of DNA-dependent transcription. | 1.24334e-06 | 400/459 | 126 | 86.443 |
| TRANSCRIPTION\_INITIATION (c5) Genes annotated by the GO term GO:0006352. Processes involved in the assembly of the RNA polymerase complex at the promoter region of a DNA template resulting in the subsequent synthesis of RNA from that promoter. | 1.34126e-06 | 34/35 | 18 | 6.591 |
| CELL\_CYCLE\_REGULATOR (c2) Obsolete by GO - was not defined before being made obsolete | 1.49398e-06 | 19/21 | 20 | 7.393 |
| SA\_G1\_AND\_S\_PHASES (c2) Cdk2, 4, and 6 bind cyclin D in G1, while cdk2/cyclin E promotes the G1/S transition. | 1.68762e-06 | 13/15 | 23 | 9.648 |
| MITOTIC\_CELL\_CYCLE (c5) Genes annotated by the GO term GO:0000278. Progression through the phases of the mitotic cell cycle, the most common eukaryotic cell cycle, which canonically comprises four successive phases called G1, S, G2, and M and includes replication of the genome and the subsequent segregation of chromosomes into daughter cells. In some variant cell cycles nuclear replication or nuclear division may not be followed by cell division, or G1 and G2 phases may be absent. | 1.85025e-06 | 141/153 | 55 | 30.875 |
| APOPTOSIS\_GO (c5) Genes annotated by the GO term GO:0006915. A form of programmed cell death induced by external or internal signals that trigger the activity of proteolytic caspases, whose actions dismantle the cell and result in cell death. Apoptosis begins internally with condensation and subsequent fragmentation of the cell nucleus (blebbing) while the plasma membrane remains intact. Other characteristics of apoptosis include DNA fragmentation and the exposure of phosphatidyl serine on the cell surface. | 1.93586e-06 | 386/425 | 150 | 106.746 |
| PEART\_HISTONE\_UP (c2) Cell-proliferation-related genes upregulated by SAHA and depsipeptide (histone deacetylase inhibitors) | 1.95223e-06 | 47/51 | 23 | 9.355 |
| INTERPHASE (c5) Genes annotated by the GO term GO:0051325. Progression through interphase, the stage of cell cycle between successive rounds of chromosome segregation. Canonically, interphase is the stage of the cell cycle during which the biochemical and physiologic functions of the cell are performed and replication of chromatin occurs. | 1.97796e-06 | 66/68 | 37 | 17.832 |
| PROGRAMMED\_CELL\_DEATH (c5) Genes annotated by the GO term GO:0012501. Cell death resulting from activation of endogenous cellular processes. | 1.99109e-06 | 387/426 | 150 | 106.776 |
| REGULATION\_OF\_RNA\_METABOLIC\_PROCESS (c5) Genes annotated by the GO term GO:0051252. Any process that modulates the frequency, rate or extent of the chemical reactions and pathways involving RNA. | 2.03639e-06 | 408/468 | 127 | 87.9 |
| REGULATION\_OF\_GENE\_EXPRESSION (c5) Genes annotated by the GO term GO:0010468. Any process that modulates the frequency, rate or extent of gene expression. Gene expression is the process in which a gene's coding sequence is converted into a mature gene product or products (proteins or RNA). This includes the production of an RNA transcript as well as any processing to produce a mature RNA product or an mRNA (for protein-coding genes) and the translation of that mRNA into protein. Some protein processing events may be included when they are required to form an active form of a product from an inactive precursor form. | 2.24374e-06 | 582/670 | 174 | 127.201 |
| INTERPHASE\_OF\_MITOTIC\_CELL\_CYCLE (c5) Genes annotated by the GO term GO:0051329. Progression through interphase, the stage of cell cycle between successive rounds of mitosis. Canonically, interphase is the stage of the cell cycle during which the biochemical and physiologic functions of the cell are performed and replication of chromatin occurs. | 2.4434e-06 | 60/62 | 33 | 15.314 |
| CELL\_DEVELOPMENT (c5) Genes annotated by the GO term GO:0048468. The process whose specific outcome is the progression of the cell over time, from its formation to the mature structure. Cell development does not include the steps involved in committing a cell to a specific fate. | 2.52597e-06 | 510/571 | 177 | 130.321 |
| GNF2\_CENPF (c4) Neighborhood of CENPF | 2.66697e-06 | 47/58 | 21 | 8.242 |
| CACTTTG,MIR-520G,MIR-520H (c3) Targets of MicroRNA CACTTTG,MIR-520G,MIR-520H | 3.22627e-06 | 150/216 | 48 | 25.969 |
| TRANSCRIPTION\_FACTOR\_ACTIVITY (c5) Genes annotated by the GO term GO:0003700. The function of binding to a specific DNA sequence in order to modulate transcription. The transcription factor may or may not also interact selectively with a protein or macromolecular complex. | 3.34996e-06 | 278/353 | 101 | 66.67 |
| RNA\_ELONGATION (c5) Genes annotated by the GO term GO:0006354. The extension of an RNA molecule after transcription initiation by the addition of ribonucleotides catalyzed by an RNA polymerase. | 4.67321e-06 | 9/10 | 7 | 1.643 |
| HSA04510\_FOCAL\_ADHESION (c2) Genes involved in focal adhesion | 4.89202e-06 | 179/192 | 113 | 78.887 |
| DNA\_DEPENDENT\_DNA\_REPLICATION (c5) Genes annotated by the GO term GO:0006261. The process whereby new strands of DNA are synthesized, using parental DNA as a template for the DNA-dependent DNA polymerases that synthesize the new strands. | 5.46318e-06 | 50/56 | 23 | 10.164 |
| BREAST\_CANCER\_ESTROGEN\_SIGNALING (c2) Genes preferentially expressed in breast cancers, especially those involved in estrogen-receptor-dependent signal transduction. | 5.47468e-06 | 82/92 | 60 | 35.671 |
| module\_254 (c4) Genes in module\_254 | 5.66772e-06 | 55/58 | 27 | 12.316 |
| MENSSEN\_MYC\_UP (c2) Genes up-regulated by MYC in HUVEC (umbilical vein endothelial cell) | 6.50424e-06 | 25/28 | 21 | 8.728 |
| YTAATTAA\_V$LHX3\_01 (c3) Genes with promoter regions [-2kb,2kb] around transcription start site containing the motif YTAATTAA which matches annotation for LHX3: LIM homeobox 3 | 7.06165e-06 | 97/145 | 22 | 9.097 |
| G2PATHWAY (c2) Activated Cdc2-cyclin B kinase regulates the G2/M transition; DNA damage stimulates the DNA-PK/ATM/ATR kinases, which inactivate Cdc2. | 7.16585e-06 | 22/23 | 35 | 17.393 |
| HSA05218\_MELANOMA (c2) Genes involved in melanoma | 7.26059e-06 | 64/71 | 53 | 30.587 |
| REGULATION\_OF\_TRANSCRIPTION\_FROM\_RNA\_POLYMERASE\_II\_PROMOTER (c5) Genes annotated by the GO term GO:0006357. Any process that modulates the frequency, rate or extent of transcription from an RNA polymerase II promoter. | 7.3194e-06 | 252/288 | 87 | 56.79 |
| V$LMO2COM\_02 (c3) Genes with promoter regions [-2kb,2kb] around transcription start site containing the motif NMGATANSG which matches annotation for LMO2: LIM domain only 2 (rhombotin-like 1) | 7.55005e-06 | 147/193 | 38 | 19.749 |
| V$OCT1\_04 (c3) Genes with promoter regions [-2kb,2kb] around transcription start site containing the motif NNNNNNNWATGCAAATNNNWNNA which matches annotation for POU2F1: POU domain, class 2, transcription factor 1 | 8.68442e-06 | 134/191 | 35 | 17.501 |
| RNA\_METABOLIC\_PROCESS (c5) Genes annotated by the GO term GO:0016070. The chemical reactions and pathways involving RNA, ribonucleic acid, one of the two main type of nucleic acid, consisting of a long, unbranched macromolecule formed from ribonucleotides joined in 3',5'-phosphodiester linkage. | 9.07962e-06 | 724/835 | 179 | 135.763 |
| DNA\_DAMAGE\_RESPONSE\_\_SIGNAL\_TRANSDUCTION (c5) Genes annotated by the GO term GO:0042770. A cascade of processes induced by the detection of DNA damage within a cell. | 9.12726e-06 | 29/34 | 24 | 10.98 |
| HEMATOPOESIS\_RELATED\_TRANSCRIPTION\_FACTORS (c2) Transcription factors involved in hematopoiesis | 9.55759e-06 | 77/83 | 51 | 29.693 |
| BREASTCA\_TWO\_CLASSES (c2) Gene set that can be used to differentiate BRCA1-linked and BRCA2-linked breast cancers | 1.01903e-05 | 111/132 | 57 | 34.574 |
| ACETAMINOPHENPATHWAY (c2) Acetaminophen selectively inhibits Cox-3, which is localized to the brain, and yields the toxic metabolite NAPQI when processed by CAR in the liver. | 1.15036e-05 | 4/6 | 2 | 0.186 |
| TSA\_HEPATOMA\_UP (c2) Up-regulated in more than one of several human hepatoma cell lines by 24-hour treatment with trichostatin A | 1.15424e-05 | 27/35 | 16 | 6.016 |
| TAAYNRNNTCC\_UNKNOWN (c3) Genes with promoter regions [-2kb,2kb] around transcription start site containing motif TAAYNRNNTCC. Motif does not match any known transcription factor | 1.30795e-05 | 99/129 | 24 | 10.646 |
| JNK\_UP (c2) Upregulated by expression of constitutively active JNK in 3T3 cells | 1.45077e-05 | 21/29 | 13 | 4.67 |
| VERNELL\_PRB\_CLSTR1 (c2) pRB pathway target genes CLUSTER 1 The listed genes were found regulated by pRB and p16 and one of the E2Fs (E2F1, E2F2, or E2F3) Cluster 1 genes are up-regulated by E2F and down-regulated by pRB and p16 | 1.52146e-05 | 48/61 | 20 | 8.296 |
| REGULATION\_OF\_APOPTOSIS (c5) Genes annotated by the GO term GO:0042981. Any process that modulates the occurrence or rate of cell death by apoptosis. | 1.52741e-05 | 310/337 | 121 | 86.292 |
| V$OCT1\_07 (c3) Genes with promoter regions [-2kb,2kb] around transcription start site containing the motif TNTATGBTAATT which matches annotation for POU2F1: POU domain, class 2, transcription factor 1 | 1.54047e-05 | 80/121 | 23 | 10.271 |
| REGULATION\_OF\_PROGRAMMED\_CELL\_DEATH (c5) Genes annotated by the GO term GO:0043067. Any process that modulates the frequency, rate or extent of programmed cell death, cell death resulting from activation of endogenous cellular processes. | 1.56809e-05 | 311/338 | 121 | 86.322 |
| ROTH\_HTERT\_DIFF (c2) Expression of selected genes involved in DNA repair and cell-cycle control in hTERT-transduced T cells | 1.76727e-05 | 27/29 | 27 | 13.473 |
| ABRAHAM\_MM\_VS\_AL\_UP (c2) Genes highly expressed in multiple myeloma (MM) versus immunoglobulin light chain amyloidosis (AL) in plasma cells. | 1.91351e-05 | 18/19 | 28 | 13.332 |
| POSITIVE\_REGULATION\_OF\_DEVELOPMENTAL\_PROCESS (c5) Genes annotated by the GO term GO:0051094. Any process that activates or increases the rate or extent of development, the biological process whose specific outcome is the progression of an organism over time from an initial condition (e.g. a zygote, or a young adult) to a later condition (e.g. a multicellular animal or an aged adult). | 1.93212e-05 | 194/215 | 81 | 53.106 |
| GNF2\_PCNA (c4) Neighborhood of PCNA | 1.93228e-05 | 55/65 | 22 | 9.703 |
| HSA04012\_ERBB\_SIGNALING\_PATHWAY (c2) Genes involved in ErbB signaling pathway | 2.01396e-05 | 85/87 | 82 | 54.306 |
| XENOBIOTIC\_METABOLIC\_PROCESS (c5) Genes annotated by the GO term GO:0006805. The chemical reactions and pathways involving a xenobiotic compound, a compound foreign to living organisms. Used of chemical compounds, e.g. a xenobiotic chemical, such as a pesticide. | 2.01513e-05 | 3/11 | 2 | 0.2 |
