# Supplementary material for: Looking at Cerebellar Malformations through Text-Mined Interactomes of Mice and Humans
Source: PLoS Comput Biol. 2009 Nov 6;5(11):e1000559. doi: 10.1371/journal.pcbi.1000559 (PMC2767227; doi:10.1371/journal.pcbi.1000559)
Supplement: Dataset S1 — All enrichment results. (0.20 MB ZIP) [file pcbi.1000559.s012.zip › enrichment_results/Table H. enrichment_hprd-small cerebellum.html]

Complete Clustering results for network hprd and phenotype small cerebellum (FDR <= 0.001)


# Complete Clustering results for network hprd and phenotype small cerebellum (FDR <= 0.001)

| Set | p-Value | Gene Count | Interaction Count | Expected Interection Count |
| --- | --- | --- | --- | --- |
| SA\_REG\_CASCADE\_OF\_CYCLIN\_EXPR (c2) Expression of cyclins regulates progression through the cell cycle by activating cyclin-dependent kinases. | 6.43929e-15 | 11/13 | 21 | 4.464 |
| INTEGRIN\_COMPLEX (c5) Genes annotated by the GO term GO:0008305. Any member of a family of heterodimeric transmembrane receptors for cell-adhesion molecules. The alpha and beta subunits are noncovalently bonded. | 2.90101e-13 | 18/19 | 13 | 2.337 |
| V$E47\_01 (c3) Genes with promoter regions [-2kb,2kb] around transcription start site containing the motif VSNGCAGGTGKNCNN which matches annotation for TCF3: transcription factor 3 (E2A immunoglobulin enhancer binding factors E12/E47) | 6.65268e-12 | 148/200 | 49 | 19.934 |
| EMBRYONIC\_MORPHOGENESIS (c5) Genes annotated by the GO term GO:0048598. The process by which anatomical structures are generated and organized during the embryonic phase. Morphogenesis pertains to the creation of form. The embryonic phase begins with zygote formation. The end of the embryonic phase is organism-specific. For example, it would be at birth for mammals, larval hatching for insects and seed dormancy in plants. | 3.44145e-11 | 11/17 | 7 | 0.927 |
| BREAST\_CANCER\_ESTROGEN\_SIGNALING (c2) Genes preferentially expressed in breast cancers, especially those involved in estrogen-receptor-dependent signal transduction. | 3.90933e-11 | 82/92 | 63 | 29.884 |
| HSA05214\_GLIOMA (c2) Genes involved in glioma | 4.23499e-11 | 61/64 | 71 | 35.382 |
| HSA04012\_ERBB\_SIGNALING\_PATHWAY (c2) Genes involved in ErbB signaling pathway | 6.29043e-11 | 85/87 | 83 | 43.85 |
| REELINPATHWAY (c2) Reelin is secreted by neurons and recognized by receptors including cadherin related neuronal receptors, which promote phosphorylation of Dab1. | 1.11091e-10 | 6/7 | 14 | 3.079 |
| module\_275 (c4) Genes in module\_275 | 1.23914e-10 | 15/16 | 14 | 3.005 |
| HSA04510\_FOCAL\_ADHESION (c2) Genes involved in focal adhesion | 7.05698e-10 | 179/192 | 109 | 64.439 |
| CELL\_ADHESION\_RECEPTOR\_ACTIVITY (c2) Obsolete by GO - combining with cell adhesion molecules to initiate a change in cell activity. | 1.99345e-09 | 30/33 | 18 | 4.907 |
| G1PATHWAY (c2) CDK4/6-cyclin D and CDK2-cyclin E phosphorylate Rb, which allows the transcription of genes needed for the G1/S cell cycle transition. | 2.66088e-09 | 25/26 | 38 | 15.858 |
| FOSBPATHWAY (c2) FOSB gene expression and drug abuse | 3.67007e-09 | 4/5 | 7 | 1.086 |
| CELLCYCLEPATHWAY (c2) Cyclins interact with cyclin-dependent kinases to form active kinase complexes that regulate progression through the cell cycle. | 5.37129e-09 | 22/23 | 27 | 9.404 |
| chr7q36 (c1) Genes in cytogenetic band chr7q36 | 6.2019e-09 | 21/68 | 8 | 1.48 |
| V$E2F1\_Q3\_01 (c3) Genes with promoter regions [-2kb,2kb] around transcription start site containing the motif TTGGCGCGRAANNGNM which matches annotation for E2F1: E2F transcription factor 1 | 1.73081e-08 | 149/193 | 43 | 19.165 |
| BRENTANI\_CELL\_CYCLE (c2) Cancer related genes involved in the cell cycle | 3.10702e-08 | 78/79 | 46 | 22.046 |
| HSA04115\_P53\_SIGNALING\_PATHWAY (c2) Genes involved in p53 signaling pathway | 3.48972e-08 | 58/66 | 42 | 19.584 |
| CELL\_SOMA (c5) Genes annotated by the GO term GO:0043025. The portion of a cell bearing surface projections such as axons, dendrites, cilia, or flagella that includes the nucleus, but excludes all cell projections. | 3.82443e-08 | 9/10 | 7 | 1.263 |
| PROLIFERATION\_GENES (c2) Proliferation related genes | 3.96568e-08 | 322/359 | 87 | 50.848 |
| HSA04512\_ECM\_RECEPTOR\_INTERACTION (c2) Genes involved in ECM-receptor interaction | 4.13999e-08 | 74/86 | 29 | 11.324 |
| CACAMPATHWAY (c2) Calcium functions as a second messenger activating the calcium/calmodulin-dependent kinases, which phosphorylate targets such as CREB. | 4.42908e-08 | 12/14 | 14 | 3.904 |
| CELL\_PROLIFERATION\_GO\_0008283 (c5) Genes annotated by the GO term GO:0008283. The multiplication or reproduction of cells, resulting in the expansion of a cell population. | 5.03362e-08 | 443/513 | 108 | 67.646 |
| EGF\_RECEPTOR\_SIGNALING\_PATHWAY (c2) EDF receptor signaling pathway | 1.03171e-07 | 12/13 | 21 | 7.393 |
| CALCINEURINPATHWAY (c2) Increased intracellular calcium activates the phosphatase calcineurin in differentiating keratinocytes. | 2.36951e-07 | 18/19 | 24 | 9.637 |
| HSA04070\_PHOSPHATIDYLINOSITOL\_SIGNALING\_SYSTEM (c2) Genes involved in phosphatidylinositol signaling system | 2.50842e-07 | 60/76 | 30 | 13.004 |
| SHEPARD\_CELL\_PROLIFERATION (c2) Cell proliferation genes determined in zebra fish | 2.74751e-07 | 175/201 | 55 | 28.669 |
| CELL\_PROLIFERATION (c2) The multiplication or reproduction of cells, resulting in the rapid expansion of a cell population. | 2.74751e-07 | 175/201 | 55 | 28.669 |
| module\_412 (c4) Genes in module\_412 | 2.89249e-07 | 12/13 | 11 | 2.749 |
| DENDRITE (c5) Genes annotated by the GO term GO:0030425. A branching protoplasmic process of a neuron that receive and integrate signals coming from axons of other neurons, and convey the resulting signal to the body of the cell. | 5.4052e-07 | 14/16 | 10 | 2.483 |
| EPIDERMAL\_GROWTH\_FACTOR\_RECEPTOR\_SIGNALING\_PATHWAY (c5) Genes annotated by the GO term GO:0007173. The series of molecular signals generated as a consequence of an epidermal growth factor receptor binding to one of its physiological ligands. | 5.8055e-07 | 20/22 | 25 | 9.989 |
| V$TFIII\_Q6 (c3) Genes with promoter regions [-2kb,2kb] around transcription start site containing the motif RGAGGKAGG which matches annotation for GTF2A1: general transcription factor IIA, 1, 19/37kDa  GTF2A2: general transcription factor IIA, 2, 12kDa | 5.85448e-07 | 125/165 | 38 | 17.366 |
| BREASTCA\_TWO\_CLASSES | 1.03582e-06 | 111/132 | 52 | 28.161 |
| G1\_TO\_S\_CELL\_CYCLE\_REACTOME (c2) | 1.15846e-06 | 63/66 | 40 | 19.547 |
| ABRAHAM\_AL\_VS\_MM\_DN (c2) Genes with significantly lower average gene expression in AL plasma cells than in MM cells | 1.33329e-06 | 17/18 | 22 | 8.676 |
| CELL\_CYCLE\_KEGG (c2) | 1.35736e-06 | 79/84 | 57 | 32.53 |
| MMS\_HUMAN\_LYMPH\_HIGH\_24HRS\_UP (c2) Up-regulated at 24 hours following treatment of human lymphocytes (TK6) with a high dose of methyl methanesulfonate (MMS) | 1.43181e-06 | 17/18 | 9 | 2.243 |
| CELL\_CYCLE\_GO\_0007049 (c5) Genes annotated by the GO term GO:0007049. The progression of biochemical and morphological phases and events that occur in a cell during successive cell replication or nuclear replication events. Canonically, the cell cycle comprises the replication and segregation of genetic material followed by the division of the cell, but in endocycles or syncytial cells nuclear replication or nuclear division may not be followed by cell division. | 1.59797e-06 | 282/311 | 79 | 48.714 |
| UVB\_NHEK3\_C4 (c2) Regulated by UV-B light in normal human epidermal keratinocytes, cluster 4 | 1.63099e-06 | 9/12 | 8 | 1.869 |
| GCAAAAA,MIR-129 (c3) Targets of MicroRNA GCAAAAA,MIR-129 | 1.96757e-06 | 113/166 | 39 | 19.23 |
| module\_308 (c4) Genes in module\_308 | 2.17749e-06 | 52/70 | 16 | 5.489 |
| MORPHOGENESIS\_OF\_AN\_EPITHELIUM (c5) Genes annotated by the GO term GO:0002009. The process by which the anatomical structures of epithelia are generated and organized. Morphogenesis pertains to the creation of form. An epithelium is a sheet of closely packed cells arranged in one or more layers, that covers the outer surfaces of the body or lines any internal cavity or tube. | 2.22844e-06 | 12/16 | 5 | 0.858 |
| TAAYNRNNTCC\_UNKNOWN (c3) Genes with promoter regions [-2kb,2kb] around transcription start site containing motif TAAYNRNNTCC. Motif does not match any known transcription factor | 2.45807e-06 | 99/129 | 23 | 9.226 |
| BLEO\_HUMAN\_LYMPH\_HIGH\_4HRS\_UP (c2) Up-regulated at 4 hours following treatment of human lymphocytes (TK6) with a high dose of bleomycin | 2.57723e-06 | 19/20 | 9 | 2.212 |
| RAC1PATHWAY (c2) Rac-1 is a Rho family G protein that stimulates formation of actin-dependent structures such as filopodia and lamellopodia. | 2.94766e-06 | 20/22 | 19 | 7.548 |
| BIOPEPTIDESPATHWAY (c2) Extracellular signaling peptides exert biological effects via G-protein coupled receptors (GPCRs), which activate intracellular GTPases. | 3.05628e-06 | 37/38 | 51 | 28.799 |
| GRAEBER\_BETA2\_INTEGRINS (c2) Genes in the beta2 integrins family | 3.30047e-06 | 10/11 | 9 | 2.233 |
| RNA\_BIOSYNTHETIC\_PROCESS (c5) Genes annotated by the GO term GO:0032774. The chemical reactions and pathways resulting in the formation of RNA, ribonucleic acid, one of the two main type of nucleic acid, consisting of a long, unbranched macromolecule formed from ribonucleotides joined in 3',5'-phosphodiester linkage. Includes polymerization of ribonucleotide monomers. | 3.70566e-06 | 562/636 | 135 | 96.625 |
| TRANSCRIPTION\_\_DNA\_DEPENDENT (c5) Genes annotated by the GO term GO:0006351. The synthesis of RNA on a template of DNA. | 3.73194e-06 | 561/634 | 135 | 96.602 |
| SKP2E2FPATHWAY (c2) E2F-1, a transcription factor that promotes the G1/S transition, is repressed by Rb and activated by cdk2/cyclin E. | 3.89793e-06 | 8/9 | 13 | 4.162 |
| HSA05223\_NON\_SMALL\_CELL\_LUNG\_CANCER (c2) Genes involved in non-small cell lung cancer | 3.93346e-06 | 53/54 | 54 | 31.018 |
| P27PATHWAY (c2) p27 blocks the G1/S transition by inhibiting the checkpoint kinase cdk2/cyclin E and is inhibited by cdk2-mediated ubiquitination. | 4.76222e-06 | 11/12 | 14 | 4.684 |
| INTEGRIN\_MEDIATED\_CELL\_ADHESION\_KEGG (c2) | 5.28829e-06 | 80/90 | 51 | 28.716 |
| NDKDYNAMINPATHWAY (c2) Endocytotic role of NDK, Phosphins and Dynamin | 5.91966e-06 | 18/19 | 15 | 5.341 |
| CAGGTG\_V$E12\_Q6 (c3) Genes with promoter regions [-2kb,2kb] around transcription start site containing the motif CAGGTG which matches annotation for TCF3: transcription factor 3 (E2A immunoglobulin enhancer binding factors E12/E47) | 6.02817e-06 | 1278/1832 | 203 | 153.606 |
| module\_321 (c4) Genes in module\_321 | 6.52162e-06 | 88/110 | 27 | 12.257 |
| INTERPHASE (c5) Genes annotated by the GO term GO:0051325. Progression through interphase, the stage of cell cycle between successive rounds of chromosome segregation. Canonically, interphase is the stage of the cell cycle during which the biochemical and physiologic functions of the cell are performed and replication of chromatin occurs. | 6.55349e-06 | 66/68 | 31 | 14.595 |
| CELL\_CYCLE (c2) The progression of biochemical and morphological events that occur during nuclear or cellular replication. | 6.72462e-06 | 72/76 | 53 | 30.157 |
| EMBRYONIC\_DEVELOPMENT (c5) Genes annotated by the GO term GO:0009790. The process whose specific outcome is the progression of an embryo from its formation until the end of its embryonic life stage. The end of the embryonic stage is organism-specific. For example, for mammals, the process would begin with zygote formation and end with birth. For insects, the process would begin at zygote formation and end with larval hatching. For plant zygotic embryos, this would be from zygote formation to the end of seed dormancy. For plant vegetative embryos, this would be from the initial determination of the cell or group of cells to form an embryo until the point when the embryo becomes independent of the parent plant. | 6.83634e-06 | 41/57 | 23 | 9.879 |
| POSITIVE\_REGULATION\_OF\_CELL\_ADHESION (c5) Genes annotated by the GO term GO:0045785. Any process that activates or increases the frequency, rate or extent of cell adhesion. | 6.9149e-06 | 11/13 | 6 | 1.213 |
| CGGTGTG,MIR-220 (c3) Targets of MicroRNA CGGTGTG,MIR-220 | 7.33162e-06 | 4/6 | 4 | 0.641 |
| module\_220 (c4) Genes in module\_220 | 7.49049e-06 | 283/329 | 59 | 34.689 |
| CELL\_CYCLE\_PROCESS (c5) Genes annotated by the GO term GO:0022402. A cellular process that is involved in the progression of biochemical and morphological phases and events that occur in a cell during successive cell replication or nuclear replication events. | 7.79666e-06 | 173/191 | 50 | 27.79 |
| POSITIVE\_REGULATION\_OF\_CELLULAR\_PROCESS (c5) Genes annotated by the GO term GO:0048522. Any process that activates or increases the frequency, rate or extent of cellular processes, those that are carried out at the cellular level, but are not necessarily restricted to a single cell. For example, cell communication occurs among more than one cell, but occurs at the cellular level. | 8.18447e-06 | 595/659 | 158 | 117.287 |
| CELL\_CYCLE\_PHASE (c5) Genes annotated by the GO term GO:0022403. A cell cycle process comprising the steps by which a cell progresses through one of the biochemical and morphological phases and events that occur during successive cell replication or nuclear replication events. | 8.89993e-06 | 156/169 | 47 | 25.797 |
| CELL\_CYCLE\_REGULATOR (c2) Obsolete by GO - was not defined before being made obsolete | 9.62662e-06 | 19/21 | 16 | 6.038 |
| UNDERHILL\_PROLIFERATION (c2) Cell cycle- and proliferation-related genes underexpressed in plasma cells. | 9.70748e-06 | 16/18 | 12 | 3.827 |
| ACATATC,MIR-190 (c3) Targets of MicroRNA ACATATC,MIR-190 | 9.72601e-06 | 33/56 | 12 | 3.759 |
| V$ATF3\_Q6 (c3) Genes with promoter regions [-2kb,2kb] around transcription start site containing the motif CBCTGACGTCANCS which matches annotation for ATF3: activating transcription factor 3 | 1.0124e-05 | 148/196 | 35 | 17.661 |
| HOFMANN\_MANTEL\_LYMPHOMA\_VS\_LYMPH\_NODES\_UP (c2) Genes whose expression is up-regulated in mantle cell lymphoma compared to hyperplastic lymph nodes as analyzed by oligonucleotide microarray | 1.02629e-05 | 43/47 | 24 | 10.546 |
| TGGTGCT,MIR-29A,MIR-29B,MIR-29C (c3) Targets of MicroRNA TGGTGCT,MIR-29A,MIR-29B,MIR-29C | 1.06841e-05 | 294/456 | 60 | 35.504 |
| V$OCT1\_04 (c3) Genes with promoter regions [-2kb,2kb] around transcription start site containing the motif NNNNNNNWATGCAAATNNNWNNA which matches annotation for POU2F1: POU domain, class 2, transcription factor 1 | 1.10596e-05 | 134/191 | 30 | 14.408 |
| V$SOX5\_01 (c3) Genes with promoter regions [-2kb,2kb] around transcription start site containing the motif NNAACAATNN which matches annotation for SOX5: SRY (sex determining region Y)-box 5 | 1.13102e-05 | 152/195 | 39 | 20.649 |
| REGULATION\_OF\_PHOSPHORYLATION (c5) Genes annotated by the GO term GO:0042325. Any process that modulates the frequency, rate or extent of addition of phosphate groups into a molecule. | 1.19871e-05 | 47/49 | 24 | 10.658 |
| TRANSCRIPTION\_FROM\_RNA\_POLYMERASE\_II\_PROMOTER (c5) Genes annotated by the GO term GO:0006366. The synthesis of RNA from a DNA template by RNA polymerase II (Pol II), originating at a Pol II-specific promoter. Includes transcription of messenger RNA (mRNA) and certain small nuclear RNAs (snRNAs). | 1.21599e-05 | 407/456 | 105 | 72.376 |
| module\_236 (c4) Genes in module\_236 | 1.23565e-05 | 14/18 | 7 | 1.664 |
| V$ZF5\_B (c3) Genes with promoter regions [-2kb,2kb] around transcription start site containing the motif NRNGNGCGCGCWN which matches annotation for ZFP161: zinc finger protein 161 homolog (mouse) | 1.28704e-05 | 137/188 | 30 | 14.149 |
| HSA00940\_PHENYLPROPANOID\_BIOSYNTHESIS (c2) Genes involved in phenylpropanoid biosynthesis | 1.43666e-05 | 3/7 | 1 | 0.054 |
