# Supplementary material for: Looking at Cerebellar Malformations through Text-Mined Interactomes of Mice and Humans
Source: PLoS Comput Biol. 2009 Nov 6;5(11):e1000559. doi: 10.1371/journal.pcbi.1000559 (PMC2767227; doi:10.1371/journal.pcbi.1000559)
Supplement: Dataset S1 — All enrichment results. (0.20 MB ZIP) [file pcbi.1000559.s012.zip › enrichment_results/Table I. enrichment_physical-abnormal foliation.html]

Complete Clustering results for network physical and phenotype abnormal foliation (FDR <= 0.001)


# Complete Clustering results for network physical and phenotype abnormal foliation (FDR <= 0.001)

| Set | p-Value | Gene Count | Interaction Count | Expected Interection Count |
| --- | --- | --- | --- | --- |
| SHHPATHWAY (c2) Sonic hedgehog (Shh) signaling in the developing CNS induces neuronal proliferation via interaction with the patched (Ptc-1) and smoothened receptors. | 1.35658e-12 | 12/14 | 16 | 3.69 |
| HSA04115\_P53\_SIGNALING\_PATHWAY (c2) Genes involved in p53 signaling pathway | 1.88438e-12 | 59/66 | 63 | 28.843 |
| HSA04340\_HEDGEHOG\_SIGNALING\_PATHWAY (c2) Genes involved in Hedgehog signaling pathway | 7.64939e-11 | 46/57 | 32 | 11.643 |
| REELINPATHWAY (c2) Reelin is secreted by neurons and recognized by receptors including cadherin related neuronal receptors, which promote phosphorylation of Dab1. | 1.79191e-10 | 6/7 | 16 | 4.212 |
| FOSBPATHWAY (c2) FOSB gene expression and drug abuse | 3.36845e-10 | 4/5 | 10 | 1.864 |
| BRAIN\_DEVELOPMENT (c5) Genes annotated by the GO term GO:0007420. The process whose specific outcome is the progression of the brain over time, from its formation to the mature structure. The brain is one of the two components of the central nervous system and is the center of thought and emotion. It is responsible for the coordination and control of bodily activities and the interpretation of information from the senses (sight, hearing, smell, etc.). | 3.63898e-09 | 40/51 | 16 | 4.401 |
| P35ALZHEIMERSPATHWAY (c2) p35, a neuron-specific activator of cyclin-dependent kinase 5, is cleaved to p25 in Alzheimer's disease and promotoes hyperphosphorylated tau formation and apoptosis. | 5.18942e-09 | 10/11 | 22 | 7.567 |
| V$FAC1\_01 (c3) Genes with promoter regions [-2kb,2kb] around transcription start site containing the motif NNNCAMAACACRNA which matches annotation for FALZ: fetal Alzheimer antigen | 5.98264e-09 | 129/158 | 47 | 21.697 |
| EMBRYONIC\_MORPHOGENESIS (c5) Genes annotated by the GO term GO:0048598. The process by which anatomical structures are generated and organized during the embryonic phase. Morphogenesis pertains to the creation of form. The embryonic phase begins with zygote formation. The end of the embryonic phase is organism-specific. For example, it would be at birth for mammals, larval hatching for insects and seed dormancy in plants. | 6.98299e-09 | 13/17 | 9 | 1.691 |
| HSA04110\_CELL\_CYCLE (c2) Genes involved in cell cycle | 1.74347e-08 | 109/112 | 106 | 66.825 |
| INTEGRIN\_COMPLEX (c5) Genes annotated by the GO term GO:0008305. Any member of a family of heterodimeric transmembrane receptors for cell-adhesion molecules. The alpha and beta subunits are noncovalently bonded. | 2.04384e-08 | 18/19 | 12 | 2.802 |
| GROWTH\_CONE (c5) Genes annotated by the GO term GO:0030426. The migrating motile tip of a growing nerve cell axon or dendrite. | 7.63055e-08 | 9/10 | 13 | 3.578 |
| CELLCYCLEPATHWAY (c2) Cyclins interact with cyclin-dependent kinases to form active kinase complexes that regulate progression through the cell cycle. | 9.79143e-08 | 22/23 | 33 | 14.775 |
| POSITIVE\_REGULATION\_OF\_DEVELOPMENTAL\_PROCESS (c5) Genes annotated by the GO term GO:0051094. Any process that activates or increases the rate or extent of development, the biological process whose specific outcome is the progression of an organism over time from an initial condition (e.g. a zygote, or a young adult) to a later condition (e.g. a multicellular animal or an aged adult). | 1.79595e-07 | 201/215 | 101 | 64.272 |
| CELL\_DEVELOPMENT (c5) Genes annotated by the GO term GO:0048468. The process whose specific outcome is the progression of the cell over time, from its formation to the mature structure. Cell development does not include the steps involved in committing a cell to a specific fate. | 2.23723e-07 | 538/571 | 215 | 160.651 |
| module\_275 (c4) Genes in module\_275 | 2.36005e-07 | 15/16 | 13 | 3.569 |
| G1PATHWAY (c2) CDK4/6-cyclin D and CDK2-cyclin E phosphorylate Rb, which allows the transcription of genes needed for the G1/S cell cycle transition. | 2.48921e-07 | 25/26 | 45 | 22.911 |
| P53\_SIGNALING (c2) Genes involved in p53 signaling | 3.0076e-07 | 86/91 | 104 | 67.349 |
| NUCLEUS (c5) Genes annotated by the GO term GO:0005634. A membrane-bounded organelle of eukaryotic cells in which chromosomes are housed and replicated. In most cells, the nucleus contains all of the cell's chromosomes except the organellar chromosomes, and is the site of RNA synthesis and processing. In some species, or in specialized cell types, RNA metabolism or DNA replication may be absent. | 4.36456e-07 | 1255/1417 | 337 | 268.849 |
| DNA\_DAMAGE\_SIGNALING (c2) Genes involved in DNA damage signaling | 4.71041e-07 | 86/89 | 55 | 29.924 |
| HSA04510\_FOCAL\_ADHESION (c2) Genes involved in focal adhesion | 5.01194e-07 | 183/192 | 141 | 97.501 |
| ST\_INTEGRIN\_SIGNALING\_PATHWAY (c2) Integrins are transmembrane receptors that mediate cell growth, survival, and migration by binding to ligands in the extracellular matrix. | 5.09532e-07 | 76/79 | 78 | 47.255 |
| chr7q36 (c1) Genes in cytogenetic band chr7q36 | 5.85332e-07 | 26/68 | 10 | 2.564 |
| SITE\_OF\_POLARIZED\_GROWTH (c5) Genes annotated by the GO term GO:0030427. Any part of a cell where non-isotropic growth takes place. | 5.8611e-07 | 10/11 | 13 | 3.908 |
| HADDAD\_HPCLYMPHO\_ENRICHED (c2) Genes enriched in CD45RAhiLin-CD10+ vs CD45RAintCD7- and CD45RAhiCD7hi HPCs | 6.05512e-07 | 197/253 | 70 | 42.047 |
| REGULATION\_OF\_DEVELOPMENTAL\_PROCESS (c5) Genes annotated by the GO term GO:0050793. Any process that modulates the frequency, rate or extent of development, the biological process whose specific outcome is the progression of a multicellular organism over time from an initial condition (e.g. a zygote, or a young adult) to a later condition (e.g. a multicellular animal or an aged adult). | 1.1949e-06 | 411/436 | 175 | 129.584 |
| CELL\_SOMA (c5) Genes annotated by the GO term GO:0043025. The portion of a cell bearing surface projections such as axons, dendrites, cilia, or flagella that includes the nucleus, but excludes all cell projections. | 1.25729e-06 | 9/10 | 9 | 2.199 |
| V$PITX2\_Q2 (c3) Genes with promoter regions [-2kb,2kb] around transcription start site containing the motif WNTAATCCCAR which matches annotation for PITX2: paired-like homeodomain transcription factor 2 | 1.30302e-06 | 149/206 | 54 | 29.428 |
| REGULATION\_OF\_PROGRAMMED\_CELL\_DEATH (c5) Genes annotated by the GO term GO:0043067. Any process that modulates the frequency, rate or extent of programmed cell death, cell death resulting from activation of endogenous cellular processes. | 1.47244e-06 | 323/338 | 149 | 107.002 |
| REGULATION\_OF\_APOPTOSIS (c5) Genes annotated by the GO term GO:0042981. Any process that modulates the occurrence or rate of cell death by apoptosis. | 1.48012e-06 | 322/337 | 149 | 106.982 |
| CELL\_CYCLE\_GO\_0007049 (c5) Genes annotated by the GO term GO:0007049. The progression of biochemical and morphological phases and events that occur in a cell during successive cell replication or nuclear replication events. Canonically, the cell cycle comprises the replication and segregation of genetic material followed by the division of the cell, but in endocycles or syncytial cells nuclear replication or nuclear division may not be followed by cell division. | 1.79208e-06 | 293/311 | 109 | 72.464 |
| HADDAD\_HSC\_CD10\_UP (c2) Genes upregulated in human hematopoietic stem cells of the line CD45RA(hi) Lin- CD10+, which are biased toward developing into B cells, versus CD45RA(int) CD7- and CD45RA(hi) CD7+. | 1.86182e-06 | 186/242 | 66 | 39.925 |
| CELL\_CYCLE\_KEGG (c2) | 1.90503e-06 | 80/84 | 77 | 48.01 |
| PROGRAMMED\_CELL\_DEATH (c5) Genes annotated by the GO term GO:0012501. Cell death resulting from activation of endogenous cellular processes. | 2.00494e-06 | 405/426 | 180 | 134.356 |
| APOPTOSIS\_GO (c5) Genes annotated by the GO term GO:0006915. A form of programmed cell death induced by external or internal signals that trigger the activity of proteolytic caspases, whose actions dismantle the cell and result in cell death. Apoptosis begins internally with condensation and subsequent fragmentation of the cell nucleus (blebbing) while the plasma membrane remains intact. Other characteristics of apoptosis include DNA fragmentation and the exposure of phosphatidyl serine on the cell surface. | 2.00677e-06 | 404/425 | 180 | 134.336 |
| CELL\_CYCLE (c2) The progression of biochemical and morphological events that occur during nuclear or cellular replication. | 2.12506e-06 | 73/76 | 72 | 43.538 |
| NERVOUS\_SYSTEM\_DEVELOPMENT (c5) Genes annotated by the GO term GO:0007399. The process whose specific outcome is the progression of nervous tissue over time, from its formation to its mature state. | 3.3207e-06 | 306/382 | 86 | 55.441 |
| CELL\_PROJECTION (c5) Genes annotated by the GO term GO:0042995. A prolongation or process extending from a cell, e.g. a flagellum or axon. | 3.44166e-06 | 93/108 | 35 | 17.359 |
| METPATHWAY (c2) The hepatocyte growth factor receptor c-Met stimulates proliferation and alters cell motility and adhesion on binding the ligand HGF. | 3.47858e-06 | 34/35 | 67 | 42.199 |
| ST\_DIFFERENTIATION\_PATHWAY\_IN\_PC12\_CELLS (c2) Rat-derived PC12 cells respond to nerve growth factor (NGF) and PACAP to differentiate into neuronal cells. | 3.9991e-06 | 40/42 | 61 | 37.524 |
| chr13q32 (c1) Genes in cytogenetic band chr13q32 | 5.29671e-06 | 12/30 | 4 | 0.626 |
| PARP\_KO\_DN (c2) Downregulated in MEF cells from PARP knockout mice | 6.5655e-06 | 11/14 | 12 | 3.699 |
| NEGATIVE\_REGULATION\_OF\_PHOSPHATE\_METABOLIC\_PROCESS (c5) Genes annotated by the GO term GO:0045936. Any process that stops, prevents or reduces the frequency, rate or extent of the chemical reactions and pathways involving phosphates. | 6.58669e-06 | 12/13 | 16 | 5.932 |
| BIOPEPTIDESPATHWAY (c2) Extracellular signaling peptides exert biological effects via G-protein coupled receptors (GPCRs), which activate intracellular GTPases. | 7.22462e-06 | 37/38 | 64 | 40.445 |
