# Supplementary material for: Looking at Cerebellar Malformations through Text-Mined Interactomes of Mice and Humans
Source: PLoS Comput Biol. 2009 Nov 6;5(11):e1000559. doi: 10.1371/journal.pcbi.1000559 (PMC2767227; doi:10.1371/journal.pcbi.1000559)
Supplement: Dataset S1 — All enrichment results. (0.20 MB ZIP) [file pcbi.1000559.s012.zip › enrichment_results/Table J. enrichment_physical-abnormal vermis.html]

Complete Clustering results for network physical and phenotype abnormal vermis (FDR <= 0.001)


# Complete Clustering results for network physical and phenotype abnormal vermis (FDR <= 0.001)

| Set | p-Value | Gene Count | Interaction Count | Expected Interection Count |
| --- | --- | --- | --- | --- |
| NUCLEUS (c5) Genes annotated by the GO term GO:0005634. A membrane-bounded organelle of eukaryotic cells in which chromosomes are housed and replicated. In most cells, the nucleus contains all of the cell's chromosomes except the organellar chromosomes, and is the site of RNA synthesis and processing. In some species, or in specialized cell types, RNA metabolism or DNA replication may be absent. | 1e-20 | 1255/1417 | 298 | 199.45 |
| module\_98 (c4) Genes in module\_98 | 3.55271e-15 | 366/391 | 135 | 75.173 |
| G1\_TO\_S\_CELL\_CYCLE\_REACTOME (c2) | 2.77556e-14 | 65/66 | 53 | 21.07 |
| CELL\_CYCLE\_KEGG (c2) | 4.35763e-13 | 80/84 | 71 | 34.017 |
| NUCLEAR\_PART (c5) Genes annotated by the GO term GO:0044428. Any constituent part of the nucleus, a membrane-bounded organelle of eukaryotic cells in which chromosomes are housed and replicated. | 1.18539e-12 | 509/572 | 141 | 81.679 |
| module\_198 (c4) Genes in module\_198 | 1.18894e-12 | 284/301 | 113 | 62.442 |
| CELL\_CYCLE (c2) The progression of biochemical and morphological events that occur during nuclear or cellular replication. | 2.48646e-12 | 73/76 | 66 | 30.947 |
| HSA04115\_P53\_SIGNALING\_PATHWAY (c2) Genes involved in p53 signaling pathway | 2.62435e-12 | 59/66 | 51 | 21.546 |
| RNA\_BIOSYNTHETIC\_PROCESS (c5) Genes annotated by the GO term GO:0032774. The chemical reactions and pathways resulting in the formation of RNA, ribonucleic acid, one of the two main type of nucleic acid, consisting of a long, unbranched macromolecule formed from ribonucleotides joined in 3',5'-phosphodiester linkage. Includes polymerization of ribonucleotide monomers. | 7.68274e-12 | 586/636 | 171 | 108.53 |
| SA\_REG\_CASCADE\_OF\_CYCLIN\_EXPR (c2) Expression of cyclins regulates progression through the cell cycle by activating cyclin-dependent kinases. | 7.72415e-12 | 12/13 | 22 | 6.294 |
| TRANSCRIPTION\_\_DNA\_DEPENDENT (c5) Genes annotated by the GO term GO:0006351. The synthesis of RNA on a template of DNA. | 1.58036e-11 | 584/634 | 170 | 108.516 |
| CELLCYCLEPATHWAY (c2) Cyclins interact with cyclin-dependent kinases to form active kinase complexes that regulate progression through the cell cycle. | 2.58608e-11 | 22/23 | 30 | 10.546 |
| DNA\_METABOLIC\_PROCESS (c5) Genes annotated by the GO term GO:0006259. The chemical reactions and pathways involving DNA, deoxyribonucleic acid, one of the two main types of nucleic acid, consisting of a long, unbranched macromolecule formed from one, or more commonly, two, strands of linked deoxyribonucleotides. | 5.44297e-11 | 236/256 | 81 | 41.632 |
| NUCLEOBASE\_\_NUCLEOSIDE\_\_NUCLEOTIDE\_AND\_NUCLEIC\_ACID\_METABOLIC\_PROCESS (c5) Genes annotated by the GO term GO:0006139. The chemical reactions and pathways involving nucleobases, nucleosides, nucleotides and nucleic acids. | 7.46454e-11 | 1112/1234 | 251 | 175.641 |
| NUCLEAR\_PORE (c5) Genes annotated by the GO term GO:0005643. Any of the numerous similar discrete openings in the nuclear envelope of a eukaryotic cell, where the inner and outer nuclear membranes are joined. | 7.92446e-11 | 30/31 | 17 | 4.219 |
| TRANSCRIPTION (c5) Genes annotated by the GO term GO:0006350. The synthesis of either RNA on a template of DNA or DNA on a template of RNA. | 9.27295e-11 | 692/750 | 198 | 133.788 |
| module\_197 (c4) Genes in module\_197 | 3.02505e-10 | 152/173 | 69 | 35.521 |
| TRANSCRIPTION\_INITIATION (c5) Genes annotated by the GO term GO:0006352. Processes involved in the assembly of the RNA polymerase complex at the promoter region of a DNA template resulting in the subsequent synthesis of RNA from that promoter. | 4.21008e-10 | 34/35 | 19 | 5.417 |
| module\_57 (c4) Genes in module\_57 | 4.50425e-10 | 54/56 | 50 | 23.452 |
| module\_252 (c4) Genes in module\_252 | 4.60753e-10 | 221/235 | 91 | 51.526 |
| TRANSCRIPTION\_FROM\_RNA\_POLYMERASE\_II\_PROMOTER (c5) Genes annotated by the GO term GO:0006366. The synthesis of RNA from a DNA template by RNA polymerase II (Pol II), originating at a Pol II-specific promoter. Includes transcription of messenger RNA (mRNA) and certain small nuclear RNAs (snRNAs). | 4.70026e-10 | 425/456 | 129 | 80.728 |
| HSA05218\_MELANOMA (c2) Genes involved in melanoma | 5.41642e-10 | 64/71 | 61 | 30.75 |
| HSA04130\_SNARE\_INTERACTIONS\_IN\_VESICULAR\_TRANSPORT (c2) Genes involved in SNARE interactions in vesicular transport | 5.62201e-10 | 30/36 | 14 | 3.192 |
| module\_124 (c4) Genes in module\_124 | 9.33538e-10 | 91/96 | 40 | 17.012 |
| PORE\_COMPLEX (c5) Genes annotated by the GO term GO:0046930. Any small opening in a membrane that allows the passage of gases and/or liquids. | 2.23706e-09 | 35/36 | 18 | 4.96 |
| CELL\_PROLIFERATION\_GO\_0008283 (c5) Genes annotated by the GO term GO:0008283. The multiplication or reproduction of cells, resulting in the expansion of a cell population. | 3.02629e-09 | 464/513 | 125 | 78.31 |
| NUCLEAR\_ENVELOPE (c5) Genes annotated by the GO term GO:0005635. The double lipid bilayer enclosing the nucleus and separating its contents from the rest of the cytoplasm; includes the intermembrane space, a gap of width 20-40 nm (also called the perinuclear space). | 4.93463e-09 | 66/73 | 29 | 11.087 |
| CELL\_CYCLE\_GO\_0007049 (c5) Genes annotated by the GO term GO:0007049. The progression of biochemical and morphological phases and events that occur in a cell during successive cell replication or nuclear replication events. Canonically, the cell cycle comprises the replication and segregation of genetic material followed by the division of the cell, but in endocycles or syncytial cells nuclear replication or nuclear division may not be followed by cell division. | 5.0588e-09 | 293/311 | 92 | 52.937 |
| BRENTANI\_CELL\_CYCLE (c2) Cancer related genes involved in the cell cycle | 5.4411e-09 | 78/79 | 50 | 23.556 |
| chr13q32 (c1) Genes in cytogenetic band chr13q32 | 1.37029e-08 | 12/30 | 4 | 0.433 |
| SARCOMAS\_SYNOVIAL\_UP (c2) Top 20 positive significant genes associated with synovial sarcomas, versus other soft-tissue tumors. | 1.50129e-08 | 8/12 | 3 | 0.242 |
| RESPONSE\_TO\_DNA\_DAMAGE\_STIMULUS (c5) Genes annotated by the GO term GO:0006974. A change in state or activity of a cell or an organism (in terms of movement, secretion, enzyme production, gene expression, etc.) as a result of a stimulus indicating damage to its DNA from environmental insults or errors during metabolism. | 3.5691e-08 | 150/161 | 55 | 27.761 |
| BIOPOLYMER\_METABOLIC\_PROCESS (c5) Genes annotated by the GO term GO:0043283. The chemical reactions and pathways involving biopolymers, long, repeating chains of monomers found in nature e.g. polysaccharides and proteins. | 4.02113e-08 | 1493/1667 | 307 | 239.358 |
| ORGANELLE\_ENVELOPE (c5) Genes annotated by the GO term GO:0031967. A double membrane structure enclosing an organelle, including two lipid bilayers and the region between them. In some cases, an organelle envelope may have more than two membranes. | 4.20331e-08 | 126/168 | 37 | 16.705 |
| ENVELOPE (c5) Genes annotated by the GO term GO:0031975. A multilayered structure surrounding all or part of a cell; encompasses one or more lipid bilayers, and may include a cell wall layer, also includes the space between layers. | 4.20331e-08 | 126/168 | 37 | 16.705 |
| PROTEIN\_DNA\_COMPLEX\_ASSEMBLY (c5) Genes annotated by the GO term GO:0065004. The aggregation, arrangement and bonding together of proteins and DNA molecules to form a protein-DNA complex. | 4.35904e-08 | 45/49 | 22 | 7.69 |
| REGULATION\_OF\_NUCLEOBASE\_\_NUCLEOSIDE\_\_NUCLEOTIDE\_AND\_NUCLEIC\_ACID\_METABOLIC\_PROCESS (c5) Genes annotated by the GO term GO:0019219. Any process that modulates the frequency, rate or extent of the chemical reactions and pathways involving nucleobases, nucleosides, nucleotides and nucleic acids. | 6.45584e-08 | 560/614 | 161 | 111.975 |
| TRANSCRIPTION\_INITIATION\_FROM\_RNA\_POLYMERASE\_II\_PROMOTER (c5) Genes annotated by the GO term GO:0006367. Processes involved in starting transcription from the RNA polymerase II promoter. | 1.04757e-07 | 28/29 | 16 | 5.058 |
| HSA04110\_CELL\_CYCLE (c2) Genes involved in cell cycle | 1.12146e-07 | 109/112 | 80 | 48.243 |
| GNF2\_PCNA (c4) Neighborhood of PCNA | 1.3361e-07 | 58/65 | 24 | 9.174 |
| LE\_MYELIN\_UP (c2) Genes upregulated in Egr2Lo/Lo mice (who bear mutations in the transcription factor Egr2 and in which peripheral nerve myelination is disrupted) whose expression is significantly altered after sciatic nerve injury. | 1.40004e-07 | 73/87 | 28 | 11.309 |
| RNA\_METABOLIC\_PROCESS (c5) Genes annotated by the GO term GO:0016070. The chemical reactions and pathways involving RNA, ribonucleic acid, one of the two main type of nucleic acid, consisting of a long, unbranched macromolecule formed from ribonucleotides joined in 3',5'-phosphodiester linkage. | 1.40492e-07 | 760/835 | 174 | 122.808 |
| REGULATION\_OF\_TRANSCRIPTION (c5) Genes annotated by the GO term GO:0045449. Any process that modulates the frequency, rate or extent of the synthesis of either RNA on a template of DNA or DNA on a template of RNA. | 2.44389e-07 | 514/563 | 149 | 103.975 |
| NEGATIVE\_REGULATION\_OF\_CELLULAR\_PROCESS (c5) Genes annotated by the GO term GO:0048523. Any process that stops, prevents or reduces the frequency, rate or extent of cellular processes, those that are carried out at the cellular level, but are not necessarily restricted to a single cell. For example, cell communication occurs among more than one cell, but occurs at the cellular level. | 2.49284e-07 | 581/640 | 164 | 118.155 |
| NUCLEAR\_LUMEN (c5) Genes annotated by the GO term GO:0031981. The volume enclosed by the nuclear inner membrane. | 2.61274e-07 | 338/381 | 95 | 59.103 |
| NUCLEAR\_MEMBRANE\_PART (c5) Genes annotated by the GO term GO:0044453. Any constituent part of the nuclear membrane, the envelope that surrounds the nucleus of eukaryotic cells. | 3.197e-07 | 39/42 | 17 | 5.669 |
| PROLIFERATION\_GENES (c2) Proliferation related genes | 4.06117e-07 | 333/359 | 96 | 61.362 |
| BREASTCA\_TWO\_CLASSES (c2) Gene set that can be used to differentiate BRCA1-linked and BRCA2-linked breast cancers | 4.35044e-07 | 118/132 | 56 | 31.341 |
| NEGATIVE\_REGULATION\_OF\_BIOLOGICAL\_PROCESS (c5) Genes annotated by the GO term GO:0048519. Any process that stops, prevents or reduces the frequency, rate or extent of a biological process. Biological processes are regulated by many means; examples include the control of gene expression, protein modification or interaction with a protein or substrate molecule. | 4.45085e-07 | 609/670 | 168 | 122.392 |
| REGULATION\_OF\_TRANSCRIPTION\_\_DNA\_DEPENDENT (c5) Genes annotated by the GO term GO:0006355. Any process that modulates the frequency, rate or extent of DNA-dependent transcription. | 4.82635e-07 | 417/459 | 119 | 79.899 |
| GNF2\_CENPF (c4) Neighborhood of CENPF | 5.10289e-07 | 52/58 | 21 | 7.913 |
| LAMB\_CYCLIN\_D3\_GLOCUS (c2) E2F target genes highly correlated with cyclin D3 expression (p = 0.002) | 5.27432e-07 | 13/15 | 10 | 2.711 |
| REGULATION\_OF\_RNA\_METABOLIC\_PROCESS (c5) Genes annotated by the GO term GO:0051252. Any process that modulates the frequency, rate or extent of the chemical reactions and pathways involving RNA. | 6.83705e-07 | 425/468 | 120 | 81.048 |
| REGULATION\_OF\_TRANSCRIPTION\_FROM\_RNA\_POLYMERASE\_II\_PROMOTER (c5) Genes annotated by the GO term GO:0006357. Any process that modulates the frequency, rate or extent of transcription from an RNA polymerase II promoter. | 7.40061e-07 | 262/288 | 83 | 52.159 |
| ZHAN\_MULTIPLE\_MYELOMA\_VS\_NORMAL\_UP (c2) The 70 most significantly up-regulated genes in MM in comparison with normal bone marrow PCs | 8.26195e-07 | 49/60 | 22 | 8.839 |
| V$E2F1\_Q4\_01 (c3) Genes with promoter regions [-2kb,2kb] around transcription start site containing the motif TTTSGCGSG which matches annotation for E2F  TFDP1: transcription factor Dp-1 | 9.14868e-07 | 133/172 | 46 | 24.588 |
| V$E2F\_01 | 1.0926e-06 | 46/57 | 22 | 8.924 |
| NUCLEAR\_MEMBRANE (c5) Genes annotated by the GO term GO:0031965. Either of the lipid bilayers that surround the nucleus and form the nuclear envelope; excludes the intermembrane space. | 1.35824e-06 | 45/50 | 19 | 7.187 |
| RESPONSE\_TO\_ENDOGENOUS\_STIMULUS (c5) Genes annotated by the GO term GO:0009719. A change in state or activity of a cell or an organism (in terms of movement, secretion, enzyme production, gene expression, etc.) as a result of an endogenous stimulus. | 1.70901e-06 | 184/198 | 57 | 31.767 |
| V$E2F\_Q3\_01 (c3) Genes with promoter regions [-2kb,2kb] around transcription start site containing the motif TTTSGCGSG which matches annotation for E2F  TFDP1: transcription factor Dp-1 | 1.78212e-06 | 134/176 | 47 | 25.404 |
| DNA\_REPAIR | 1.81622e-06 | 117/125 | 42 | 21.686 |
| PARP\_KO\_DN (c2) Downregulated in MEF cells from PARP knockout mice | 1.83985e-06 | 11/14 | 10 | 2.638 |
| module\_403 (c4) Genes in module\_403 | 1.90367e-06 | 41/46 | 27 | 12.211 |
| GNF2\_CDC2 (c4) Neighborhood of CDC2 | 2.11448e-06 | 52/58 | 19 | 7.487 |
| DNA\_BINDING (c5) Genes annotated by the GO term GO:0003677. Interacting selectively with DNA (deoxyribonucleic acid). | 2.20264e-06 | 513/600 | 137 | 97.176 |
| P53\_SIGNALING (c2) Genes involved in p53 signaling | 2.26119e-06 | 86/91 | 77 | 49.285 |
| VERNELL\_PRB\_CLSTR1 (c2) pRB pathway target genes CLUSTER 1 The listed genes were found regulated by pRB and p16 and one of the E2Fs (E2F1, E2F2, or E2F3) Cluster 1 genes are up-regulated by E2F and down-regulated by pRB and p16 | 2.57682e-06 | 50/61 | 20 | 7.89 |
| MEMBRANE\_ENCLOSED\_LUMEN (c5) Genes annotated by the GO term GO:0031974. The enclosed volume within a sealed membrane or between two sealed membranes. | 2.73949e-06 | 376/451 | 96 | 62.594 |
| ORGANELLE\_LUMEN (c5) Genes annotated by the GO term GO:0043233. The volume enclosed by the membranes of a particular organelle, e.g. endoplasmic reticulum lumen, or the space between the two lipid bilayers of a double membrane surrounding an organelle, e.g. nuclear membrane lumen. | 2.73949e-06 | 376/451 | 96 | 62.594 |
| module\_154 (c4) Genes in module\_154 | 3.07659e-06 | 56/75 | 17 | 5.974 |
| GNF2\_RRM1 (c4) Neighborhood of RRM1 | 3.08506e-06 | 76/85 | 25 | 10.774 |
| LATE\_ENDOSOME (c5) Genes annotated by the GO term GO:0005770. A prelysosomal endocytic organelle differentiated from early endosomes by lower lumenal pH and different protein composition. Late endosomes are more spherical than early endosomes and are mostly juxtanuclear, being concentrated near the microtubule organizing center. | 3.31073e-06 | 11/12 | 6 | 1.213 |
| HSA00130\_UBIQUINONE\_BIOSYNTHESIS (c2) Genes involved in ubiquinone biosynthesis | 3.38959e-06 | 3/8 | 1 | 0.047 |
| NUCLEOLUS (c5) Genes annotated by the GO term GO:0005730. A small, dense body one or more of which are present in the nucleus of eukaryotic cells. It is rich in RNA and protein, is not bounded by a limiting membrane, and is not seen during mitosis. Its prime function is the transcription of the nucleolar DNA into 45S ribosomal-precursor RNA, the processing of this RNA into 5.8S, 18S, and 28S components of ribosomal RNA, and the association of these components with 5S RNA and proteins synthesized outside the nucleolus. This association results in the formation of ribonucleoprotein precursors; these pass into the cytoplasm and mature into the 40S and 60S subunits of the ribosome. | 3.50704e-06 | 101/123 | 33 | 16.463 |
| module\_18 (c4) Genes in module\_18 | 3.6208e-06 | 376/447 | 86 | 55.714 |
| TARTE\_PLASMA\_BLASTIC (c2) Genes overexpressed in mature plasma cells isolated from tonsils (TPCs) and mature plasma cells isolated from bone marrow (BMPCs) as compared to polyclonal plasmablastic cells (PPCs). | 3.9617e-06 | 251/286 | 64 | 38.745 |
| NEGATIVE\_REGULATION\_OF\_NUCLEOBASE\_\_NUCLEOSIDE\_\_NUCLEOTIDE\_AND\_NUCLEIC\_ACID\_METABOLIC\_PROCESS (c5) Genes annotated by the GO term GO:0045934. Any process that stops, prevents or reduces the frequency, rate or extent of the chemical reactions and pathways involving nucleobases, nucleosides, nucleotides and nucleic acids. | 4.09652e-06 | 190/209 | 69 | 43.217 |
| GREENBAUM\_E2A\_UP (c2) Table includes transcripts up-regulated 3-fold or greater in the E2A-deficient cell lines | 4.61725e-06 | 31/33 | 12 | 3.723 |
| GNF2\_H2AFX (c4) Neighborhood of H2AFX | 4.66358e-06 | 25/30 | 15 | 5.475 |
| REGULATION\_OF\_CELL\_PROLIFERATION (c5) Genes annotated by the GO term GO:0042127. Any process that modulates the frequency, rate or extent of cell proliferation. | 5.03648e-06 | 278/308 | 79 | 49.953 |
| REGULATION\_OF\_CELL\_CYCLE (c5) Genes annotated by the GO term GO:0051726. Any process that modulates the rate or extent of progression through the cell cycle. | 5.26046e-06 | 170/180 | 59 | 35.01 |
| NEGATIVE\_REGULATION\_OF\_CELL\_PROLIFERATION (c5) Genes annotated by the GO term GO:0008285. Any process that stops, prevents or reduces the rate or extent of cell proliferation. | 5.36731e-06 | 137/156 | 41 | 21.526 |
| INTEGRIN\_COMPLEX (c5) Genes annotated by the GO term GO:0008305. Any member of a family of heterodimeric transmembrane receptors for cell-adhesion molecules. The alpha and beta subunits are noncovalently bonded. | 5.52352e-06 | 18/19 | 8 | 2.031 |
| module\_54 (c4) Genes in module\_54 | 5.93237e-06 | 204/255 | 46 | 25.336 |
| V$E2F1\_Q6 (c3) Genes with promoter regions [-2kb,2kb] around transcription start site containing the motif TTTSGCGS which matches annotation for E2F1: E2F transcription factor 1 | 6.02673e-06 | 141/174 | 45 | 24.426 |
| REGULATION\_OF\_METABOLIC\_PROCESS (c5) Genes annotated by the GO term GO:0019222. Any process that modulates the frequency, rate or extent of the chemical reactions and pathways within a cell or an organism. | 6.08728e-06 | 724/794 | 188 | 143.235 |
| G1PATHWAY (c2) CDK4/6-cyclin D and CDK2-cyclin E phosphorylate Rb, which allows the transcription of genes needed for the G1/S cell cycle transition. | 6.12187e-06 | 25/26 | 32 | 16.011 |
| module\_349 (c4) Genes in module\_349 | 6.67274e-06 | 10/17 | 3 | 0.384 |
| REGULATION\_OF\_CELLULAR\_METABOLIC\_PROCESS (c5) Genes annotated by the GO term GO:0031323. Any process that modulates the frequency, rate or extent of the chemical reactions and pathways by which individual cells transform chemical substances. | 6.80587e-06 | 712/782 | 185 | 140.648 |
| SGCGSSAAA\_V$E2F1DP2\_01 (c3) Genes with promoter regions [-2kb,2kb] around transcription start site containing the motif SGCGSSAAA which matches annotation for E2F1: E2F transcription factor 1  TFDP1: transcription factor Dp-1  RB1: retinoblastoma 1 (including osteosarcoma) | 7.85974e-06 | 100/127 | 32 | 15.74 |
| HSA00534\_HEPARAN\_SULFATE\_BIOSYNTHESIS | 8.10571e-06 | 3/19 | 1 | 0.049 |
| SKP2E2FPATHWAY | 8.24677e-06 | 8/9 | 13 | 4.689 |
| NUCLEOPLASM (c5) Genes annotated by the GO term GO:0005654. That part of the nuclear content other than the chromosomes or the nucleolus. | 9.25839e-06 | 254/276 | 79 | 50.75 |
| V$E2F\_03 (c3) Genes with promoter regions [-2kb,2kb] around transcription start site containing motif TTTSGCGCGMNR. Motif does not match any known transcription factor | 9.49317e-06 | 136/176 | 39 | 20.501 |
| TRANSCRIPTION\_REPRESSOR\_ACTIVITY (c5) Genes annotated by the GO term GO:0016564. Any transcription regulator activity that prevents or downregulates transcription. | 1.01607e-05 | 142/150 | 43 | 23.64 |
| V$E2F\_Q4\_01 (c3) Genes with promoter regions [-2kb,2kb] around transcription start site containing the motif NCSCGCSAAAN which matches annotation for E2F  TFDP1: transcription factor Dp-1 | 1.12361e-05 | 137/175 | 43 | 23.578 |
| REGULATION\_OF\_GENE\_EXPRESSION | 1.13509e-05 | 606/670 | 155 | 115.587 |
| NEGATIVE\_REGULATION\_OF\_TRANSCRIPTION (c5) Genes annotated by the GO term GO:0016481. Any process that stops, prevents or reduces the frequency, rate or extent of transcription. | 1.23861e-05 | 169/187 | 65 | 40.963 |
| GNF2\_CENPE (c4) Neighborhood of CENPE | 1.54155e-05 | 35/39 | 15 | 5.876 |
| V$COMP1\_01 (c3) Genes with promoter regions [-2kb,2kb] around transcription start site containing the motif NVTNWTGATTGACNACAAVARRBN which matches annotation for MYOG: myogenin (myogenic factor 4) | 1.56246e-05 | 76/94 | 19 | 7.981 |
| V$E2F\_Q6\_01 (c3) Genes with promoter regions [-2kb,2kb] around transcription start site containing the motif NKCGCGCSAAAN which matches annotation for E2F  TFDP1: transcription factor Dp-1 | 1.74357e-05 | 133/171 | 42 | 23.371 |
