# Supplementary material for: Looking at Cerebellar Malformations through Text-Mined Interactomes of Mice and Humans
Source: PLoS Comput Biol. 2009 Nov 6;5(11):e1000559. doi: 10.1371/journal.pcbi.1000559 (PMC2767227; doi:10.1371/journal.pcbi.1000559)
Supplement: Dataset S1 — All enrichment results. (0.20 MB ZIP) [file pcbi.1000559.s012.zip › enrichment_results/Table K. enrichment_physical-absent cerebellum.html]

Complete Clustering results for network physical and phenotype absent cerebellum (FDR <= 0.001)


# Complete Clustering results for network physical and phenotype absent cerebellum (FDR <= 0.001)

| Set | p-Value | Gene Count | Interaction Count | Expected Interection Count |
| --- | --- | --- | --- | --- |
| HSA05217\_BASAL\_CELL\_CARCINOMA (c2) Genes involved in basal cell carcinoma | 1e-20 | 47/55 | 17 | 2.117 |
| TRANSMEMBRANE\_RECEPTOR\_PROTEIN\_PHOSPHATASE\_ACTIVITY (c5) Genes annotated by the GO term GO:0019198. The catalysis of phosphate removal from a phosphotyrosine using aspartic acid as a nucleophile in a metal-dependent manner. | 1e-20 | 18/19 | 6 | 0.393 |
| HSA04310\_WNT\_SIGNALING\_PATHWAY (c2) Genes involved in Wnt signaling pathway | 1e-20 | 128/147 | 38 | 11.107 |
| HSA00534\_HEPARAN\_SULFATE\_BIOSYNTHESIS (c2) Genes involved in heparan sulfate biosynthesis | 1e-20 | 3/19 | 1 | 0.008 |
| GPCRS\_CLASS\_A\_RHODOPSIN\_LIKE\_2 (c2) GPCR class A rhodopsin genes | 1e-20 | 3/10 | 1 | 0.014 |
| GPCRDB\_CLASS\_A\_RHODOPSIN\_LIKE2 (c2) | 1e-20 | 3/13 | 1 | 0.014 |
| HYPERME\_COLONCA\_SW48 (c2) Gene identified by chromatin IP and CpG island microarray as being hypermethylated in SW48 colon cancer cells, versus normal colon murcosa or WI38 fibroblasts | 8.88178e-16 | 9/14 | 4 | 0.238 |
| V$BRN2\_01 (c3) Genes with promoter regions [-2kb,2kb] around transcription start site containing the motif NNCATNSRWAATNMRN which matches annotation for POU3F2: POU domain, class 3, transcription factor 2 | 1.44229e-12 | 150/190 | 15 | 3.061 |
| HSA04520\_ADHERENS\_JUNCTION (c2) Genes involved in adherens junction | 5.11091e-12 | 73/75 | 29 | 9.927 |
| PROTEIN\_TYROSINE\_PHOSPHATASE\_ACTIVITY (c5) Genes annotated by the GO term GO:0004725. Catalysis of the reaction: protein tyrosine phosphate + H2O = protein tyrosine + phosphate. | 1.51599e-11 | 48/53 | 9 | 1.42 |
| ST\_WNT\_BETA\_CATENIN\_PATHWAY (c2) Beta-catenin is degraded in the absence of Wnt signaling; when extracellular Wnt binds Frizzled receptors, beta-catenin accumulates in the nucleus and may promote cell survival. | 3.5512e-11 | 25/31 | 10 | 1.823 |
| DISTECHE\_XINACTIVATED\_GENES (c2) Genes that escape X inactivation | 2.74149e-10 | 15/19 | 2 | 0.093 |
| PS1PATHWAY (c2) Presenilin is required for gamma-secretase activity to activate Notch signaling; presenilin also inhibits beta-catenin in the Wnt/Frizzled pathway. | 3.14449e-10 | 11/12 | 7 | 1.005 |
| UVC\_LOW\_C3\_DN (c2) Down-regulated at 12 hours following treatment of WS1 human skin fibroblasts with UVC at a low dose (10 J/m^2) (cluster c3) | 9.03816e-10 | 16/19 | 3 | 0.206 |
| HSA04340\_HEDGEHOG\_SIGNALING\_PATHWAY (c2) Genes involved in Hedgehog signaling pathway | 1.31567e-09 | 46/57 | 9 | 1.751 |
| TRANSMEMBRANE\_RECEPTOR\_ACTIVITY (c5) Genes annotated by the GO term GO:0004888. Combining with an extracellular or intracellular messenger to initiate a change in cell activity, and spanning to the membrane of either the cell or an organelle. | 1.34645e-08 | 354/418 | 21 | 6.948 |
| PHOSPHORIC\_MONOESTER\_HYDROLASE\_ACTIVITY (c5) Genes annotated by the GO term GO:0016791. Catalysis of the hydrolysis of phosphoric monoesters, releasing inorganic phosphate. | 1.72935e-08 | 94/111 | 11 | 2.438 |
| BRAIN\_DEVELOPMENT (c5) Genes annotated by the GO term GO:0007420. The process whose specific outcome is the progression of the brain over time, from its formation to the mature structure. The brain is one of the two components of the central nervous system and is the center of thought and emotion. It is responsible for the coordination and control of bodily activities and the interpretation of information from the senses (sight, hearing, smell, etc.). | 2.89074e-08 | 40/51 | 5 | 0.705 |
| chr12p11 (c1) Genes in cytogenetic band chr12p11 | 3.18697e-08 | 19/30 | 2 | 0.119 |
| PHOSPHOPROTEIN\_PHOSPHATASE\_ACTIVITY (c5) Genes annotated by the GO term GO:0004721. Catalysis of the reaction: a phosphoprotein + H2O = a protein + phosphate. Together with protein kinases, these enzymes control the state of phosphorylation of cell proteins and thereby provide an important mechanism for regulating cellular activity. | 6.9332e-08 | 73/81 | 10 | 2.227 |
| CENTRAL\_NERVOUS\_SYSTEM\_DEVELOPMENT (c5) Genes annotated by the GO term GO:0007417. The process whose specific outcome is the progression of the central nervous system over time, from its formation to the mature structure. The central nervous system is the core nervous system that serves an integrating and coordinating function. In vertebrates it consists of the brain, spinal cord and spinal nerves. In those invertebrates with a central nervous system it typically consists of a brain, cerebral ganglia and a nerve cord. | 7.49545e-08 | 101/123 | 10 | 2.236 |
| HEART\_DEVELOPMENT (c5) Genes annotated by the GO term GO:0007507. The process whose specific outcome is the progression of the heart over time, from its formation to the mature structure. The heart is a hollow, muscular organ, which, by contracting rhythmically, keeps up the circulation of the blood. | 7.50407e-08 | 31/37 | 6 | 0.982 |
| chr11p13 (c1) Genes in cytogenetic band chr11p13 | 1.27052e-07 | 17/38 | 4 | 0.468 |
| WNTPATHWAY (c2) The Wnt glycoprotein binds to membrane-bound receptors such as Frizzled to activate a number of signaling pathways, including that of beta-catenin. | 3.647e-07 | 22/24 | 11 | 3.142 |
| SANSOM\_APC\_LOSS4\_UP (c2) The top 174 genes upregulated following Apc loss at day 4 | 3.70515e-07 | 88/111 | 9 | 2.015 |
| WNT\_TARGETS (c2) WNT target genes from literatures | 4.06835e-07 | 20/22 | 7 | 1.496 |
| PHOSPHORIC\_ESTER\_HYDROLASE\_ACTIVITY (c5) Genes annotated by the GO term GO:0042578. Catalysis of the reaction: RPO-R' + H2O = RPOOH + R'H. This reaction is the hydrolysis of any phosphoric ester bond, any ester formed from orthophosphoric acid, O=P(OH)3. | 8.76252e-07 | 125/151 | 11 | 2.962 |
| chr7q36 (c1) Genes in cytogenetic band chr7q36 | 9.15625e-07 | 26/68 | 3 | 0.333 |
| CTTTGT\_V$LEF1\_Q2 (c3) Genes with promoter regions [-2kb,2kb] around transcription start site containing the motif CTTTGT which matches annotation for LEF1: lymphoid enhancer-binding factor 1 | 9.62674e-07 | 1140/1460 | 52 | 28.448 |
| PITX2PATHWAY (c2) The bicoid-related transcription factor Pitx2 is activated by Wnt binding to the Frizzled receptor and induces tissue-specific cell proliferation. | 1.43978e-06 | 14/15 | 9 | 2.551 |
| BRENTANI\_CELL\_ADHESION (c2) Cancer related genes involved in cell adhesion and metalloproteinases | 1.53315e-06 | 88/93 | 12 | 3.519 |
| SHHPATHWAY (c2) Sonic hedgehog (Shh) signaling in the developing CNS induces neuronal proliferation via interaction with the patched (Ptc-1) and smoothened receptors. | 2.08721e-06 | 12/14 | 4 | 0.549 |
| HSA05210\_COLORECTAL\_CANCER (c2) Genes involved in colorectal cancer | 2.09146e-06 | 83/84 | 25 | 11.568 |
| CELL\_FATE\_COMMITMENT (c5) Genes annotated by the GO term GO:0045165. The commitment of cells to specific cell fates and their capacity to differentiate into particular kinds of cells. Positional information is established through protein signals that emanate from a localized source within a cell (the initial one-cell zygote) or within a developmental field. | 2.10272e-06 | 11/13 | 4 | 0.658 |
| ACTIN\_FILAMENT\_BUNDLE\_FORMATION (c5) Genes annotated by the GO term GO:0051017. The assembly of actin filament bundles; actin filaments are on the same axis but may be oriented with the same or opposite polarities and may be packed with different levels of tightness. | 2.42409e-06 | 10/13 | 3 | 0.354 |
| RIBOFLAVIN\_METABOLISM (c2) | 2.66273e-06 | 7/10 | 1 | 0.046 |
| chr2q12 (c1) Genes in cytogenetic band chr2q12 | 3.05242e-06 | 22/36 | 3 | 0.349 |
| V$COMP1\_01 (c3) Genes with promoter regions [-2kb,2kb] around transcription start site containing the motif NVTNWTGATTGACNACAAVARRBN which matches annotation for MYOG: myogenin (myogenic factor 4) | 4.62938e-06 | 76/94 | 7 | 1.605 |
| V$T3R\_Q6 (c3) Genes with promoter regions [-2kb,2kb] around transcription start site containing motif MNTGWCCTN. Motif does not match any known transcription factor | 5.08828e-06 | 147/194 | 9 | 2.421 |
