# Supplementary material for: Looking at Cerebellar Malformations through Text-Mined Interactomes of Mice and Humans
Source: PLoS Comput Biol. 2009 Nov 6;5(11):e1000559. doi: 10.1371/journal.pcbi.1000559 (PMC2767227; doi:10.1371/journal.pcbi.1000559)
Supplement: Dataset S1 — All enrichment results. (0.20 MB ZIP) [file pcbi.1000559.s012.zip › enrichment_results/Table L. enrichment_physical-all.html]

Complete Clustering results for network physical and phenotype all (FDR <= 0.001)


# Complete Clustering results for network physical and phenotype all (FDR <= 0.001)

| Set | p-Value | Gene Count | Interaction Count | Expected Interection Count |
| --- | --- | --- | --- | --- |
| KINASE\_ACTIVITY (c5) Genes annotated by the GO term GO:0016301. Catalysis of the transfer of a phosphate group, usually from ATP, to a substrate molecule. | 1.9984e-15 | 331/363 | 485 | 352.306 |
| HSA04012\_ERBB\_SIGNALING\_PATHWAY (c2) Genes involved in ErbB signaling pathway | 8.88178e-15 | 85/87 | 364 | 256.693 |
| ST\_INTEGRIN\_SIGNALING\_PATHWAY (c2) Integrins are transmembrane receptors that mediate cell growth, survival, and migration by binding to ligands in the extracellular matrix. | 1.12133e-14 | 76/79 | 271 | 179.646 |
| PHOSPHOTRANSFERASE\_ACTIVITY\_\_ALCOHOL\_GROUP\_AS\_ACCEPTOR (c5) Genes annotated by the GO term GO:0016773. Catalysis of the transfer of a phosphorus-containing group from one compound (donor) to an alcohol group (acceptor). | 1.82077e-14 | 303/329 | 462 | 335.486 |
| TRANSFERASE\_ACTIVITY\_\_TRANSFERRING\_PHOSPHORUS\_CONTAINING\_GROUPS (c5) Genes annotated by the GO term GO:0016772. Catalysis of the transfer of a phosphorus-containing group from one compound (donor) to another (acceptor). | 8.84848e-14 | 374/418 | 493 | 364.302 |
| PROTEIN\_KINASE\_ACTIVITY (c5) Genes annotated by the GO term GO:0004672. Catalysis of the phosphorylation of an amino acid residue in a protein, usually according to the reaction: a protein + ATP = a phosphoprotein + ADP. | 2.4325e-13 | 267/280 | 440 | 321.391 |
| SYNAPSE (c5) Genes annotated by the GO term GO:0045202. The junction between a nerve fiber of one neuron and another neuron or muscle fiber or glial cell; the site of interneuronal communication. As the nerve fiber approaches the synapse it enlarges into a specialized structure, the presynaptic nerve ending, which contains mitochondria and synaptic vesicles. At the tip of the nerve ending is the presynaptic membrane; facing it, and separated from it by a minute cleft (the synaptic cleft) is a specialized area of membrane on the receiving cell, known as the postsynaptic membrane. In response to the arrival of nerve impulses, the presynaptic nerve ending secretes molecules of neurotransmitters into the synaptic cleft. These diffuse across the cleft and transmit the signal to the postsynaptic membrane. | 4.25215e-13 | 25/27 | 60 | 26.035 |
| MAPKKK\_CASCADE\_GO\_0000165 (c5) Genes annotated by the GO term GO:0000165. Cascade of at least three protein kinase activities culminating in the phosphorylation and activation of a MAP kinase. MAPKKK cascades lie downstream of numerous signaling pathways. | 8.65197e-13 | 98/102 | 166 | 100.988 |
| STRESS\_ACTIVATED\_PROTEIN\_KINASE\_SIGNALING\_PATHWAY (c5) Genes annotated by the GO term GO:0031098. A series of molecular signals in which a stress-activated protein kinase (SAPK) cascade relays one or more of the signals. | 9.47242e-13 | 45/47 | 96 | 49.665 |
| REELINPATHWAY (c2) Reelin is secreted by neurons and recognized by receptors including cadherin related neuronal receptors, which promote phosphorylation of Dab1. | 2.33058e-12 | 6/7 | 44 | 16.717 |
| JNK\_CASCADE (c5) Genes annotated by the GO term GO:0007254. A cascade of protein kinase activities, culminating in the phosphorylation and activation of a member of the JUN kinase subfamily of stress-activated protein kinases, which in turn are a subfamily of mitogen-activated protein (MAP) kinases that is activated primarily by cytokines and exposure to environmental stress. | 8.34988e-12 | 44/45 | 92 | 48.452 |
| S\_PHASE\_OF\_MITOTIC\_CELL\_CYCLE (c5) Genes annotated by the GO term GO:0000084. Progression through S phase, the part of the mitotic cell cycle during which DNA synthesis takes place. | 1.42765e-11 | 9/10 | 29 | 9.625 |
| HSA04115\_P53\_SIGNALING\_PATHWAY (c2) Genes involved in p53 signaling pathway | 5.40292e-11 | 59/66 | 180 | 113.731 |
| GROWTH\_CONE (c5) Genes annotated by the GO term GO:0030426. The migrating motile tip of a growing nerve cell axon or dendrite. | 5.6307e-11 | 9/10 | 37 | 14.027 |
| ST\_DIFFERENTIATION\_PATHWAY\_IN\_PC12\_CELLS (c2) Rat-derived PC12 cells respond to nerve growth factor (NGF) and PACAP to differentiate into neuronal cells. | 3.13791e-10 | 40/42 | 204 | 141.569 |
| NERVOUS\_SYSTEM\_DEVELOPMENT (c5) Genes annotated by the GO term GO:0007399. The process whose specific outcome is the progression of nervous tissue over time, from its formation to its mature state. | 9.27345e-10 | 306/382 | 294 | 210.828 |
| module\_274 (c4) Genes in module\_274 | 1.36428e-09 | 75/82 | 103 | 58.824 |
| MAP\_KINASE\_ACTIVITY (c5) Genes annotated by the GO term GO:0004707. Catalysis of the phosphorylation of proteins. Mitogen-activated protein kinase; a family of protein kinases that perform a crucial step in relaying signals from the plasma membrane to the nucleus. They are activated by a wide range of proliferation- or differentiation-inducing signals; activation is strong with agonists such as polypeptide growth factors and tumor-promoting phorbol esters, but weak (in most cell backgrounds) by stress stimuli. | 1.51495e-09 | 11/12 | 66 | 33.932 |
| SITE\_OF\_POLARIZED\_GROWTH (c5) Genes annotated by the GO term GO:0030427. Any part of a cell where non-isotropic growth takes place. | 1.55896e-09 | 10/11 | 38 | 15.534 |
| TRANSMEMBRANE\_RECEPTOR\_PROTEIN\_TYROSINE\_KINASE\_SIGNALING\_PATHWAY (c5) Genes annotated by the GO term GO:0007169. The series of molecular signals generated as a consequence of a transmembrane receptor tyrosine kinase binding to its physiological ligand. | 2.31646e-09 | 80/83 | 230 | 163.618 |
| chr7q36 (c1) Genes in cytogenetic band chr7q36 | 2.37274e-09 | 26/68 | 27 | 9.918 |
| ST\_GRANULE\_CELL\_SURVIVAL\_PATHWAY (c2) The survival and differentiation of granule cells in the brain is controlled by pro-growth PACAP and pro-apoptotic ceramides. | 2.77144e-09 | 24/26 | 123 | 77.079 |
| AXON (c5) Genes annotated by the GO term GO:0030424. The long process of a neuron that conducts nerve impulses, usually away from the cell body to the terminals and varicosities, which are sites of storage and release of neurotransmitter. | 4.75821e-09 | 11/12 | 36 | 15.226 |
| HSA01510\_NEURODEGENERATIVE\_DISEASES (c2) Genes involved in neurodegenerative diseases | 5.41722e-09 | 36/38 | 174 | 119.361 |
| PROTEIN\_SERINE\_THREONINE\_KINASE\_ACTIVITY (c5) Genes annotated by the GO term GO:0004674. Catalysis of the reaction: ATP + a protein serine/threonine = ADP + protein serine/threonine phosphate. | 5.58752e-09 | 192/201 | 304 | 224.175 |
| HSA05010\_ALZHEIMERS\_DISEASE (c2) Genes involved in Alzheimer's disease | 5.98644e-09 | 27/28 | 107 | 64.525 |
| module\_66 (c4) Genes in module\_66 | 8.5819e-09 | 459/543 | 366 | 277.246 |
| LEARNING\_AND\_OR\_MEMORY (c5) Genes annotated by the GO term GO:0007611. The acquisition and processing of information and/or the storage and retrieval of this information over time. | 9.3418e-09 | 11/14 | 46 | 21.715 |
| module\_137 (c4) Genes in module\_137 | 2.77533e-08 | 453/539 | 357 | 272.67 |
| module\_100 (c4) Genes in module\_100 | 3.02319e-08 | 452/536 | 355 | 271.301 |
| chr5p11 (c1) Genes in cytogenetic band chr5p11 | 3.1493e-08 | 0/1 | 1 | 0.033 |
| HSA05214\_GLIOMA (c2) Genes involved in glioma | 5.6521e-08 | 61/64 | 268 | 198.97 |
| NEURITE\_DEVELOPMENT (c5) Genes annotated by the GO term GO:0031175. The process whose specific outcome is the progression of the neurite over time, from its formation to the mature structure. The neurite is any process extending from a neural cell, such as axons or dendrites. | 1.18553e-07 | 50/53 | 70 | 39.74 |
| NEURON\_DEVELOPMENT (c5) Genes annotated by the GO term GO:0048666. The process whose specific outcome is the progression of a neuron over time, from initial commitment of the cell to a specific fate, to the fully functional differentiated cell. | 1.21717e-07 | 58/61 | 77 | 44.748 |
| REGULATION\_OF\_NEURON\_APOPTOSIS (c5) Genes annotated by the GO term GO:0043523. Any process that modulates the occurrence or rate of cell death by apoptosis in neurons. | 1.60783e-07 | 11/12 | 35 | 15.974 |
| V$E47\_01 (c3) Genes with promoter regions [-2kb,2kb] around transcription start site containing the motif VSNGCAGGTGKNCNN which matches annotation for TCF3: transcription factor 3 (E2A immunoglobulin enhancer binding factors E12/E47) | 1.70563e-07 | 155/200 | 154 | 106.317 |
| NEURON\_PROJECTION (c5) Genes annotated by the GO term GO:0043005. A prolongation or process extending from a nerve cell, e.g. an axon or dendrite. | 2.13205e-07 | 19/20 | 40 | 18.96 |
| S\_PHASE (c5) Genes annotated by the GO term GO:0051320. Progression through S phase, the part of the cell cycle during which DNA synthesis takes place. | 2.30766e-07 | 13/14 | 31 | 13.712 |
| FASPATHWAY (c2) Binding of the Fas ligand to the Fas receptor induces caspase activation and consequent apoptosis in the Fas-expressing cell. | 2.33459e-07 | 26/27 | 114 | 75.301 |
| BIOPEPTIDESPATHWAY (c2) Extracellular signaling peptides exert biological effects via G-protein coupled receptors (GPCRs), which activate intracellular GTPases. | 2.83956e-07 | 37/38 | 211 | 152.638 |
| module\_12 (c4) Genes in module\_12 | 3.54979e-07 | 301/354 | 290 | 220.208 |
| CELLCYCLEPATHWAY (c2) Cyclins interact with cyclin-dependent kinases to form active kinase complexes that regulate progression through the cell cycle. | 4.12571e-07 | 22/23 | 91 | 55.781 |
| DENDRITE (c5) Genes annotated by the GO term GO:0030425. A branching protoplasmic process of a neuron that receive and integrate signals coming from axons of other neurons, and convey the resulting signal to the body of the cell. | 4.69085e-07 | 15/16 | 31 | 14.242 |
| CYSTEINE\_TYPE\_PEPTIDASE\_ACTIVITY (c5) Genes annotated by the GO term GO:0008234. Catalysis of the hydrolysis of peptide linkages in oligopeptides or polypeptides; a cysteine residue is at the active center. | 4.79501e-07 | 43/54 | 67 | 38.925 |
| module\_11 (c4) Genes in module\_11 | 5.58067e-07 | 445/533 | 346 | 269.633 |
| CELL\_SOMA (c5) Genes annotated by the GO term GO:0043025. The portion of a cell bearing surface projections such as axons, dendrites, cilia, or flagella that includes the nucleus, but excludes all cell projections. | 5.82105e-07 | 9/10 | 23 | 8.928 |
| V$IK3\_01 (c3) Genes with promoter regions [-2kb,2kb] around transcription start site containing motif TNYTGGGAATACC. Motif does not match any known transcription factor | 6.37171e-07 | 131/169 | 107 | 70.186 |
| NEUROGENESIS (c5) Genes annotated by the GO term GO:0022008. Generation of cells within the nervous system. | 6.53407e-07 | 87/93 | 105 | 67.625 |
| METPATHWAY (c2) The hepatocyte growth factor receptor c-Met stimulates proliferation and alters cell motility and adhesion on binding the ligand HGF. | 6.76345e-07 | 34/35 | 213 | 160.637 |
| SA\_REG\_CASCADE\_OF\_CYCLIN\_EXPR (c2) Expression of cyclins regulates progression through the cell cycle by activating cyclin-dependent kinases. | 7.65827e-07 | 12/13 | 58 | 31.923 |
| CELL\_PROJECTION (c5) Genes annotated by the GO term GO:0042995. A prolongation or process extending from a cell, e.g. a flagellum or axon. | 8.09406e-07 | 93/108 | 106 | 67.553 |
| HSA05223\_NON\_SMALL\_CELL\_LUNG\_CANCER (c2) Genes involved in non-small cell lung cancer | 8.30365e-07 | 53/54 | 233 | 175.662 |
| ASTON\_DEPRESSION\_DN (c2) Genes downregulated in major depressive disorder (p < 0.05, fold change > 1.4, mean average difference > 150 in at least one of the groups, called present in greater than 20% of all samples) | 9.06971e-07 | 117/140 | 144 | 99.821 |
| chr11p1 (c1) Genes in cytogenetic band chr11p1 | 9.8457e-07 | 0/1 | 7 | 1.491 |
| ERK5PATHWAY (c2) Signaling between a tissue and its innervating axon stimulates retrograde transport via Trk receptors, which activate Erk5, which induces transcription of anti-apoptotic factors. | 1.06099e-06 | 16/17 | 127 | 87.08 |
| INTEGRIN\_MEDIATED\_CELL\_ADHESION\_KEGG (c2) | 1.18178e-06 | 82/90 | 215 | 162.045 |
| SIGNAL\_TRANSDUCTION (c5) Genes annotated by the GO term GO:0007165. The cascade of processes by which a signal interacts with a receptor, causing a change in the level or activity of a second messenger or other downstream target, and ultimately effecting a change in the functioning of the cell. | 1.4028e-06 | 1468/1625 | 1402 | 1268.46 |
| PROTEIN\_MODIFICATION\_PROCESS (c5) Genes annotated by the GO term GO:0006464. The covalent alteration of one or more amino acids occurring in proteins, peptides and nascent polypeptides (co-translational, post-translational modifications). Includes the modification of charged tRNAs that are destined to occur in a protein (pre-translation modification). | 1.73075e-06 | 544/623 | 599 | 506.21 |
| ABRAHAM\_AL\_VS\_MM\_DN (c2) Genes with significantly lower average gene expression in AL plasma cells than in MM cells | 1.80019e-06 | 17/18 | 83 | 52.467 |
| TRANSMEMBRANE\_RECEPTOR\_PROTEIN\_TYROSINE\_KINASE\_ACTIVITY (c5) Genes annotated by the GO term GO:0004714. Catalysis of the reaction: ATP + a protein-L-tyrosine = ADP + a protein-L-tyrosine phosphate, to initiate a change in cell activity. | 2.07603e-06 | 39/43 | 89 | 56.107 |
| CORTEX\_ENRICHMENT\_LATE\_UP (c2) Up-regulated in the cortex of mice that are exposed to an enriched environmental habitat for 2 or 14 days | 2.10468e-06 | 17/20 | 33 | 15.673 |
| FOSBPATHWAY (c2) FOSB gene expression and drug abuse | 2.28019e-06 | 4/5 | 20 | 7.547 |
| HSA04510\_FOCAL\_ADHESION (c2) Genes involved in focal adhesion | 2.60747e-06 | 183/192 | 445 | 368.799 |
| chr20p11 (c1) Genes in cytogenetic band chr20p11 | 3.208e-06 | 23/68 | 18 | 6.75 |
| BIOPOLYMER\_MODIFICATION (c5) Genes annotated by the GO term GO:0043412. The covalent alteration of one or more monomeric units in a polypeptide, polynucleotide, polysaccharide, or other biological polymer, resulting in a change in its properties. | 3.47663e-06 | 562/642 | 611 | 520.836 |
| SHHPATHWAY (c2) Sonic hedgehog (Shh) signaling in the developing CNS induces neuronal proliferation via interaction with the patched (Ptc-1) and smoothened receptors. | 4.59944e-06 | 12/14 | 30 | 14.229 |
| module\_154 (c4) Genes in module\_154 | 6.11098e-06 | 56/75 | 57 | 32.826 |
| POST\_TRANSLATIONAL\_PROTEIN\_MODIFICATION (c5) Genes annotated by the GO term GO:0043687. The covalent alteration of one or more amino acids occurring in a protein after the protein has been completely translated and released from the ribosome. | 6.2511e-06 | 435/470 | 541 | 457.13 |
| EPIDERMAL\_GROWTH\_FACTOR\_RECEPTOR\_SIGNALING\_PATHWAY (c5) Genes annotated by the GO term GO:0007173. The series of molecular signals generated as a consequence of an epidermal growth factor receptor binding to one of its physiological ligands. | 7.18203e-06 | 21/22 | 85 | 55.812 |
| BRAIN\_DEVELOPMENT (c5) Genes annotated by the GO term GO:0007420. The process whose specific outcome is the progression of the brain over time, from its formation to the mature structure. The brain is one of the two components of the central nervous system and is the center of thought and emotion. It is responsible for the coordination and control of bodily activities and the interpretation of information from the senses (sight, hearing, smell, etc.). | 7.97462e-06 | 40/51 | 34 | 16.541 |
| CYSTEINE\_TYPE\_ENDOPEPTIDASE\_ACTIVITY (c5) Genes annotated by the GO term GO:0004197. Catalysis of the hydrolysis of nonterminal peptide linkages in oligopeptides or polypeptides; a cysteine residue is at the active center. | 1.05618e-05 | 31/40 | 55 | 32.659 |
| CAGGTG\_V$E12\_Q6 (c3) Genes with promoter regions [-2kb,2kb] around transcription start site containing the motif CAGGTG which matches annotation for TCF3: transcription factor 3 (E2A immunoglobulin enhancer binding factors E12/E47) | 1.16835e-05 | 1379/1832 | 962 | 859.327 |
| P35ALZHEIMERSPATHWAY (c2) p35, a neuron-specific activator of cyclin-dependent kinase 5, is cleaved to p25 in Alzheimer's disease and promotoes hyperphosphorylated tau formation and apoptosis. | 1.22865e-05 | 10/11 | 51 | 29.234 |
| module\_2 (c4) Genes in module\_2 | 1.28014e-05 | 328/381 | 302 | 239.816 |
