# Supplementary material for: Looking at Cerebellar Malformations through Text-Mined Interactomes of Mice and Humans
Source: PLoS Comput Biol. 2009 Nov 6;5(11):e1000559. doi: 10.1371/journal.pcbi.1000559 (PMC2767227; doi:10.1371/journal.pcbi.1000559)
Supplement: Dataset S1 — All enrichment results. (0.20 MB ZIP) [file pcbi.1000559.s012.zip › enrichment_results/Table M. enrichment_physical-ataxia.html]

Complete Clustering results for network physical and phenotype ataxia (FDR <= 0.001)


# Complete Clustering results for network physical and phenotype ataxia (FDR <= 0.001)

| Set | p-Value | Gene Count | Interaction Count | Expected Interection Count |
| --- | --- | --- | --- | --- |
| PHOSPHOTRANSFERASE\_ACTIVITY\_\_ALCOHOL\_GROUP\_AS\_ACCEPTOR (c5) Genes annotated by the GO term GO:0016773. Catalysis of the transfer of a phosphorus-containing group from one compound (donor) to an alcohol group (acceptor). | 1e-20 | 303/329 | 346 | 226.47 |
| KINASE\_ACTIVITY (c5) Genes annotated by the GO term GO:0016301. Catalysis of the transfer of a phosphate group, usually from ATP, to a substrate molecule. | 1e-20 | 331/363 | 365 | 237.945 |
| TRANSFERASE\_ACTIVITY\_\_TRANSFERRING\_PHOSPHORUS\_CONTAINING\_GROUPS (c5) Genes annotated by the GO term GO:0016772. Catalysis of the transfer of a phosphorus-containing group from one compound (donor) to another (acceptor). | 1e-20 | 374/418 | 370 | 245.852 |
| PROTEIN\_KINASE\_ACTIVITY (c5) Genes annotated by the GO term GO:0004672. Catalysis of the phosphorylation of an amino acid residue in a protein, usually according to the reaction: a protein + ATP = a phosphoprotein + ADP. | 1.11022e-16 | 267/280 | 330 | 217.028 |
| STRESS\_ACTIVATED\_PROTEIN\_KINASE\_SIGNALING\_PATHWAY (c5) Genes annotated by the GO term GO:0031098. A series of molecular signals in which a stress-activated protein kinase (SAPK) cascade relays one or more of the signals. | 1.55431e-15 | 45/47 | 77 | 33.246 |
| ST\_INTEGRIN\_SIGNALING\_PATHWAY (c2) Integrins are transmembrane receptors that mediate cell growth, survival, and migration by binding to ligands in the extracellular matrix. | 3.9968e-15 | 76/79 | 195 | 120.077 |
| JNK\_CASCADE (c5) Genes annotated by the GO term GO:0007254. A cascade of protein kinase activities, culminating in the phosphorylation and activation of a member of the JUN kinase subfamily of stress-activated protein kinases, which in turn are a subfamily of mitogen-activated protein (MAP) kinases that is activated primarily by cytokines and exposure to environmental stress. | 8.50431e-14 | 44/45 | 73 | 32.406 |
| module\_274 (c4) Genes in module\_274 | 2.30038e-13 | 75/82 | 82 | 39.564 |
| MAP\_KINASE\_ACTIVITY (c5) Genes annotated by the GO term GO:0004707. Catalysis of the phosphorylation of proteins. Mitogen-activated protein kinase; a family of protein kinases that perform a crucial step in relaying signals from the plasma membrane to the nucleus. They are activated by a wide range of proliferation- or differentiation-inducing signals; activation is strong with agonists such as polypeptide growth factors and tumor-promoting phorbol esters, but weak (in most cell backgrounds) by stress stimuli. | 2.71894e-13 | 11/12 | 54 | 22.646 |
| PROTEIN\_SERINE\_THREONINE\_KINASE\_ACTIVITY (c5) Genes annotated by the GO term GO:0004674. Catalysis of the reaction: ATP + a protein serine/threonine = ADP + protein serine/threonine phosphate. | 9.39804e-13 | 192/201 | 229 | 149.933 |
| MAPKKK\_CASCADE\_GO\_0000165 (c5) Genes annotated by the GO term GO:0000165. Cascade of at least three protein kinase activities culminating in the phosphorylation and activation of a MAP kinase. MAPKKK cascades lie downstream of numerous signaling pathways. | 1.5149e-12 | 98/102 | 121 | 67.776 |
| chr5p11 (c1) Genes in cytogenetic band chr5p11 | 1.33877e-11 | 0/1 | 1 | 0.022 |
| LEARNING\_AND\_OR\_MEMORY (c5) Genes annotated by the GO term GO:0007611. The acquisition and processing of information and/or the storage and retrieval of this information over time. | 6.01251e-11 | 11/14 | 37 | 14.522 |
| S\_PHASE\_OF\_MITOTIC\_CELL\_CYCLE (c5) Genes annotated by the GO term GO:0000084. Progression through S phase, the part of the mitotic cell cycle during which DNA synthesis takes place. | 1.16326e-10 | 9/10 | 22 | 6.437 |
| REELINPATHWAY (c2) Reelin is secreted by neurons and recognized by receptors including cadherin related neuronal receptors, which promote phosphorylation of Dab1. | 1.72349e-10 | 6/7 | 31 | 11.393 |
| SYNAPSE (c5) Genes annotated by the GO term GO:0045202. The junction between a nerve fiber of one neuron and another neuron or muscle fiber or glial cell; the site of interneuronal communication. As the nerve fiber approaches the synapse it enlarges into a specialized structure, the presynaptic nerve ending, which contains mitochondria and synaptic vesicles. At the tip of the nerve ending is the presynaptic membrane; facing it, and separated from it by a minute cleft (the synaptic cleft) is a specialized area of membrane on the receiving cell, known as the postsynaptic membrane. In response to the arrival of nerve impulses, the presynaptic nerve ending secretes molecules of neurotransmitters into the synaptic cleft. These diffuse across the cleft and transmit the signal to the postsynaptic membrane. | 2.42696e-10 | 25/27 | 43 | 17.596 |
| HSA05010\_ALZHEIMERS\_DISEASE (c2) Genes involved in Alzheimer's disease | 3.53684e-10 | 27/28 | 81 | 43.164 |
| ST\_GRANULE\_CELL\_SURVIVAL\_PATHWAY (c2) The survival and differentiation of granule cells in the brain is controlled by pro-growth PACAP and pro-apoptotic ceramides. | 8.82863e-10 | 24/26 | 92 | 52.148 |
| ST\_DIFFERENTIATION\_PATHWAY\_IN\_PC12\_CELLS (c2) Rat-derived PC12 cells respond to nerve growth factor (NGF) and PACAP to differentiate into neuronal cells. | 9.66413e-10 | 40/42 | 145 | 94.654 |
| HSA04115\_P53\_SIGNALING\_PATHWAY (c2) Genes involved in p53 signaling pathway | 1.03445e-09 | 59/66 | 127 | 76.853 |
| HSA04012\_ERBB\_SIGNALING\_PATHWAY (c2) Genes involved in ErbB signaling pathway | 9.54057e-09 | 85/87 | 237 | 172.36 |
| SIG\_CD40PATHWAYMAP (c2) Genes related to CD40 signaling | 1.10986e-08 | 30/33 | 102 | 61.027 |
| TRANSMEMBRANE\_RECEPTOR\_PROTEIN\_TYROSINE\_KINASE\_ACTIVITY (c5) Genes annotated by the GO term GO:0004714. Catalysis of the reaction: ATP + a protein-L-tyrosine = ADP + a protein-L-tyrosine phosphate, to initiate a change in cell activity. | 6.19108e-08 | 39/43 | 68 | 38.464 |
| HSA01510\_NEURODEGENERATIVE\_DISEASES (c2) Genes involved in neurodegenerative diseases | 8.4799e-08 | 36/38 | 124 | 79.874 |
| TRANSMISSION\_OF\_NERVE\_IMPULSE (c5) Genes annotated by the GO term GO:0019226. The sequential electrochemical polarization and depolarization that travels across the membrane of a nerve cell (neuron) in response to stimulation. | 9.32456e-08 | 156/188 | 117 | 74.792 |
| INTEGRAL\_TO\_PLASMA\_MEMBRANE (c5) Genes annotated by the GO term GO:0005887. Penetrating at least one phospholipid bilayer of a plasma membrane. May also refer to the state of being buried in the bilayer with no exposure outside the bilayer. | 9.45974e-08 | 774/977 | 332 | 254.014 |
| HSA04664\_FC\_EPSILON\_RI\_SIGNALING\_PATHWAY (c2) Genes involved in Fc epsilon RI signaling pathway | 1.0616e-07 | 69/75 | 179 | 126.856 |
| NERVOUS\_SYSTEM\_DEVELOPMENT (c5) Genes annotated by the GO term GO:0007399. The process whose specific outcome is the progression of nervous tissue over time, from its formation to its mature state. | 1.10638e-07 | 306/382 | 202 | 142.349 |
| INTRINSIC\_TO\_PLASMA\_MEMBRANE (c5) Genes annotated by the GO term GO:0031226. Located in the plasma membrane such that some covalently attached portion of the gene product, for example part of a peptide sequence or some other covalently attached moiety such as a GPI anchor, spans or is embedded in one or both leaflets of the membrane. | 1.22042e-07 | 786/991 | 333 | 255.622 |
| MAPKPATHWAY (c2) The mitogen-activated protein (MAP) kinase pathway is a common signaling mechanism and has four main sub-pathways: Erk, JNK/SAPK, p53, and ERK5. | 1.85805e-07 | 84/85 | 209 | 153.168 |
| GROWTH\_CONE (c5) Genes annotated by the GO term GO:0030426. The migrating motile tip of a growing nerve cell axon or dendrite. | 2.43379e-07 | 9/10 | 24 | 9.64 |
| SYNAPTIC\_TRANSMISSION (c5) Genes annotated by the GO term GO:0007268. The process of communication from a neuron to a target (neuron, muscle, or secretory cell) across a synapse. | 2.44371e-07 | 143/173 | 110 | 70.212 |
| RECEPTOR\_SIGNALING\_PROTEIN\_SERINE\_THREONINE\_KINASE\_ACTIVITY (c5) Genes annotated by the GO term GO:0004702. | 3.92068e-07 | 32/33 | 73 | 43.194 |
| ASTON\_DEPRESSION\_DN (c2) Genes downregulated in major depressive disorder (p < 0.05, fold change > 1.4, mean average difference > 150 in at least one of the groups, called present in greater than 20% of all samples) | 6.60437e-07 | 117/140 | 106 | 66.889 |
| LOW\_DENSITY\_LIPOPROTEIN\_BINDING (c5) Genes annotated by the GO term GO:0030169. Interacting selectively with low-density lipoprotein, one of the classes of lipoproteins found in blood plasma in many animals (data normally relate to humans). | 8.96787e-07 | 11/12 | 10 | 2.506 |
| S\_PHASE (c5) Genes annotated by the GO term GO:0051320. Progression through S phase, the part of the cell cycle during which DNA synthesis takes place. | 9.49542e-07 | 13/14 | 23 | 9.117 |
| CELLCYCLEPATHWAY (c2) Cyclins interact with cyclin-dependent kinases to form active kinase complexes that regulate progression through the cell cycle. | 9.51534e-07 | 22/23 | 64 | 37.6 |
| SITE\_OF\_POLARIZED\_GROWTH (c5) Genes annotated by the GO term GO:0030427. Any part of a cell where non-isotropic growth takes place. | 1.02207e-06 | 10/11 | 25 | 10.602 |
| HSA04510\_FOCAL\_ADHESION (c2) Genes involved in focal adhesion | 1.37896e-06 | 183/192 | 315 | 249.19 |
| module\_12 (c4) Genes in module\_12 | 1.4623e-06 | 301/354 | 204 | 149.393 |
| PROTEIN\_MODIFICATION\_PROCESS (c5) Genes annotated by the GO term GO:0006464. The covalent alteration of one or more amino acids occurring in proteins, peptides and nascent polypeptides (co-translational, post-translational modifications). Includes the modification of charged tRNAs that are destined to occur in a protein (pre-translation modification). | 1.5441e-06 | 544/623 | 419 | 342.026 |
| INTEGRAL\_TO\_MEMBRANE (c5) Genes annotated by the GO term GO:0016021. Penetrating at least one phospholipid bilayer of a membrane. May also refer to the state of being buried in the bilayer with no exposure outside the bilayer. When used to describe a protein, indicates that all or part of the peptide sequence is embedded in the membrane. | 1.54479e-06 | 1033/1325 | 386 | 310.464 |
| HSA04912\_GNRH\_SIGNALING\_PATHWAY (c2) Genes involved in GnRH signaling pathway | 1.62742e-06 | 84/97 | 187 | 137.407 |
| module\_236 (c4) Genes in module\_236 | 1.6398e-06 | 15/18 | 23 | 8.936 |
| BIOPOLYMER\_MODIFICATION (c5) Genes annotated by the GO term GO:0043412. The covalent alteration of one or more monomeric units in a polypeptide, polynucleotide, polysaccharide, or other biological polymer, resulting in a change in its properties. | 2.10061e-06 | 562/642 | 429 | 351.954 |
| PROTEIN\_AMINO\_ACID\_AUTOPHOSPHORYLATION (c5) Genes annotated by the GO term GO:0046777. The phosphorylation by a protein of one or more of its own amino acid residues, or residues on an identical protein. | 2.19319e-06 | 27/29 | 45 | 25.321 |
| INTRINSIC\_TO\_MEMBRANE (c5) Genes annotated by the GO term GO:0031224. Located in a membrane such that some covalently attached portion of the gene product, for example part of a peptide sequence or some other covalently attached moiety such as a GPI anchor, spans or is embedded in one or both leaflets of the membrane. | 2.24319e-06 | 1049/1343 | 388 | 313.334 |
| PROTEIN\_AUTOPROCESSING (c5) Genes annotated by the GO term GO:0016540. Processing which a protein carries out itself. This involves actions such as the autolytic removal of residues to generate the mature form of the protein. | 2.3228e-06 | 28/30 | 45 | 25.407 |
| PROTEIN\_SERINE\_THREONINE\_TYROSINE\_KINASE\_ACTIVITY (c5) Genes annotated by the GO term GO:0004712. Catalysis of the reaction: ATP + a protein serine/threonine/tyrosine = ADP + protein serine/threonine/tyrosine phosphate. | 2.70953e-06 | 9/10 | 24 | 10.282 |
| module\_66 (c4) Genes in module\_66 | 3.07182e-06 | 459/543 | 248 | 186.582 |
| CYSTEINE\_TYPE\_PEPTIDASE\_ACTIVITY (c5) Genes annotated by the GO term GO:0008234. Catalysis of the hydrolysis of peptide linkages in oligopeptides or polypeptides; a cysteine residue is at the active center. | 3.14677e-06 | 43/54 | 48 | 26.277 |
| AGED\_MOUSE\_HYPOTH\_DN (c2) Down-regulated in the hypothalamus of aged (22 months) BALB/c mice, compared to young (2 months) controls | 3.31261e-06 | 34/38 | 51 | 28.458 |
| LIPOPROTEIN\_BINDING (c5) Genes annotated by the GO term GO:0008034. Interacting selectively with any conjugated, water-soluble protein in which the nonprotein moiety consists of a lipid or lipids. | 3.56623e-06 | 16/17 | 20 | 7.831 |
| CREBPATHWAY (c2) CREB is a transcription factor that binds to cAMP-responsive elements (CREs) to activate transcription in response to extracellular signaling. | 4.39388e-06 | 25/27 | 103 | 69.721 |
| CELL\_DEATH (c2) Activation or halting of cellular processes so that vital functions markedly cease, culminating in cell death. | 4.43539e-06 | 10/12 | 20 | 8.014 |
| ARENRF2PATHWAY (c2) Nrf1 and nrf2 are transcription factors that bind to antioxidant response elements (AREs), promoters of genes involved in oxidative damage control. | 4.55754e-06 | 13/14 | 69 | 42.865 |
| HSA04010\_MAPK\_SIGNALING\_PATHWAY (c2) Genes involved in MAPK signaling pathway | 4.6191e-06 | 226/252 | 339 | 274.398 |
| chr19q (c1) Genes in cytogenetic band chr19q | 5.35951e-06 | 2/4 | 5 | 0.95 |
| SA\_REG\_CASCADE\_OF\_CYCLIN\_EXPR (c2) Expression of cyclins regulates progression through the cell cycle by activating cyclin-dependent kinases. | 6.44408e-06 | 12/13 | 41 | 21.558 |
| PROTEIN\_TYROSINE\_KINASE\_ACTIVITY (c5) Genes annotated by the GO term GO:0004713. Catalysis of the reaction: ATP + a protein tyrosine = ADP + protein tyrosine phosphate. | 6.60948e-06 | 59/63 | 91 | 59.888 |
| module\_100 (c4) Genes in module\_100 | 6.96271e-06 | 452/536 | 241 | 182.666 |
| module\_137 (c4) Genes in module\_137 | 7.10418e-06 | 453/539 | 242 | 183.549 |
| REGULATION\_OF\_MAPKKK\_CASCADE (c5) Genes annotated by the GO term GO:0043408. Any process that modulates the frequency, rate or extent of signal transduction mediated by the MAPKKK cascade. | 9.79356e-06 | 19/20 | 21 | 8.863 |
| CYSTEINE\_TYPE\_ENDOPEPTIDASE\_ACTIVITY (c5) Genes annotated by the GO term GO:0004197. Catalysis of the hydrolysis of nonterminal peptide linkages in oligopeptides or polypeptides; a cysteine residue is at the active center. | 9.91406e-06 | 31/40 | 41 | 22.034 |
| PHOSPHORYLATION (c5) Genes annotated by the GO term GO:0016310. The process of introducing a phosphate group into a molecule, usually with the formation of a phosphoric ester, a phosphoric anhydride or a phosphoric amide. | 1.0385e-05 | 289/307 | 296 | 236.982 |
| ERK5PATHWAY (c2) Signaling between a tissue and its innervating axon stimulates retrograde transport via Trk receptors, which activate Erk5, which induces transcription of anti-apoptotic factors. | 1.14385e-05 | 16/17 | 87 | 58.04 |
| TRANSMEMBRANE\_RECEPTOR\_PROTEIN\_TYROSINE\_KINASE\_SIGNALING\_PATHWAY (c5) Genes annotated by the GO term GO:0007169. The series of molecular signals generated as a consequence of a transmembrane receptor tyrosine kinase binding to its physiological ligand. | 1.18397e-05 | 80/83 | 152 | 111.172 |
