# Supplementary material for: Looking at Cerebellar Malformations through Text-Mined Interactomes of Mice and Humans
Source: PLoS Comput Biol. 2009 Nov 6;5(11):e1000559. doi: 10.1371/journal.pcbi.1000559 (PMC2767227; doi:10.1371/journal.pcbi.1000559)
Supplement: Dataset S1 — All enrichment results. (0.20 MB ZIP) [file pcbi.1000559.s012.zip › enrichment_results/Table N. enrichment_physical-degeneration.html]

Complete Clustering results for network physical and phenotype degeneration (FDR <= 0.001)


# Complete Clustering results for network physical and phenotype degeneration (FDR <= 0.001)

| Set | p-Value | Gene Count | Interaction Count | Expected Interection Count |
| --- | --- | --- | --- | --- |
| SINGLE\_STRANDED\_DNA\_BINDING (c5) Genes annotated by the GO term GO:0003697. Interacting selectively with single-stranded DNA. | 1e-20 | 33/35 | 22 | 4.291 |
| GNF2\_SPRR1B (c4) Neighborhood of SPRR1B | 1e-20 | 17/24 | 8 | 0.621 |
| chr5p11 (c1) Genes in cytogenetic band chr5p11 | 1e-20 | 0/1 | 1 | 0.008 |
| STRUCTURE\_SPECIFIC\_DNA\_BINDING (c5) Genes annotated by the GO term GO:0043566. Interacting selectively with DNA of a specific structure or configuration e.g. triplex DNA binding or bent DNA binding. | 1.11022e-16 | 53/56 | 28 | 7.31 |
| DNA\_REPAIR (c5) Genes annotated by the GO term GO:0006281. The process of restoring DNA after damage. Genomes are subject to damage by chemical and physical agents in the environment (e.g. UV and ionizing radiations, chemical mutagens, fungal and bacterial toxins, etc.) and by free radicals or alkylating agents endogenously generated in metabolism. DNA is also damaged because of errors during its replication. A variety of different DNA repair pathways have been reported that include direct reversal, base excision repair, nucleotide excision repair, photoreactivation, bypass, double-strand break repair pathway, and mismatch repair pathway. | 6.77236e-15 | 117/125 | 42 | 14.23 |
| NUCLEAR\_PORE (c5) Genes annotated by the GO term GO:0005643. Any of the numerous similar discrete openings in the nuclear envelope of a eukaryotic cell, where the inner and outer nuclear membranes are joined. | 2.25375e-14 | 30/31 | 15 | 2.825 |
| HSA00600\_SPHINGOLIPID\_METABOLISM (c2) Genes involved in sphingolipid metabolism | 3.80806e-14 | 25/38 | 6 | 0.526 |
| GLYCOSPHINGOLIPID\_METABOLISM (c2) | 1.09945e-12 | 20/22 | 5 | 0.424 |
| RESPONSE\_TO\_DNA\_DAMAGE\_STIMULUS (c5) Genes annotated by the GO term GO:0006974. A change in state or activity of a cell or an organism (in terms of movement, secretion, enzyme production, gene expression, etc.) as a result of a stimulus indicating damage to its DNA from environmental insults or errors during metabolism. | 1.41642e-12 | 150/161 | 47 | 18.037 |
| ARYLSULFATASE\_ACTIVITY (c5) Genes annotated by the GO term GO:0004065. Catalysis of the reaction: a phenol sulfate + H2O = a phenol + SO4(2-) (sulfate). | 1.64857e-12 | 5/12 | 2 | 0.076 |
| KERATINOCYTE\_DIFFERENTIATION (c5) Genes annotated by the GO term GO:0030216. The process whereby a relatively unspecialized cell acquires specialized features of a keratinocyte. | 8.50753e-12 | 13/15 | 6 | 0.703 |
| BRENTANI\_REPAIR (c2) Cancer related genes involved in DNA repair | 2.67192e-11 | 35/36 | 19 | 4.808 |
| PORE\_COMPLEX (c5) Genes annotated by the GO term GO:0046930. Any small opening in a membrane that allows the passage of gases and/or liquids. | 2.78458e-11 | 35/36 | 15 | 3.324 |
| SULFURIC\_ESTER\_HYDROLASE\_ACTIVITY (c5) Genes annotated by the GO term GO:0008484. Catalysis of the reaction: RSO-R' + H2O = RSOOH + R'H. This reaction is the hydrolysis of any sulfuric ester bond, any ester formed from sulfuric acid, O=SO(OH)2. | 3.85415e-11 | 7/16 | 2 | 0.088 |
| RESPONSE\_TO\_ENDOGENOUS\_STIMULUS (c5) Genes annotated by the GO term GO:0009719. A change in state or activity of a cell or an organism (in terms of movement, secretion, enzyme production, gene expression, etc.) as a result of an endogenous stimulus. | 6.26068e-11 | 184/198 | 49 | 20.599 |
| APPEL\_IMATINIB\_UP (c2) Genes up-regulated by imatinib during dendritic cell differentiation | 1.07913e-10 | 25/31 | 12 | 2.357 |
| DNA\_METABOLIC\_PROCESS (c5) Genes annotated by the GO term GO:0006259. The chemical reactions and pathways involving DNA, deoxyribonucleic acid, one of the two main types of nucleic acid, consisting of a long, unbranched macromolecule formed from one, or more commonly, two, strands of linked deoxyribonucleotides. | 1.14389e-10 | 236/256 | 59 | 27.142 |
| MEIOTIC\_RECOMBINATION (c5) Genes annotated by the GO term GO:0007131. The cell cycle process whereby double strand breaks are formed and repaired through a double Holliday junction intermediate. This results in the equal exchange of genetic material between non-sister chromatids in a pair of homologous chromosomes. These reciprocal recombinant products ensure the proper segregation of homologous chromosomes during meiosis I and create genetic diversity. | 1.20526e-10 | 15/17 | 9 | 1.487 |
| CELL\_CYCLE\_CHECKPOINT (c2) A point in the eukaryotic cell cycle where progress through the cycle can be halted until conditions are suitable for the cell to proceed to the next stage. | 2.95441e-10 | 23/24 | 21 | 6.381 |
| ATRBRCAPATHWAY (c2) BRCA1 and 2 block cell cycle progression in response to DNA damage and promote double-stranded break repair; mutations induce breast cancer susceptibility. | 3.99159e-10 | 20/21 | 18 | 5.16 |
| module\_283 (c4) Genes in module\_283 | 4.14515e-10 | 6/7 | 6 | 0.736 |
| MEIOSIS\_I (c5) Genes annotated by the GO term GO:0007127. Progression through the first phase of meiosis, in which cells divide and homologous chromosomes are paired and segregated from each other, producing two daughter cells. | 7.23074e-10 | 17/19 | 9 | 1.567 |
| NUCLEAR\_MEMBRANE\_PART (c5) Genes annotated by the GO term GO:0044453. Any constituent part of the nuclear membrane, the envelope that surrounds the nucleus of eukaryotic cells. | 7.9907e-10 | 39/42 | 15 | 3.737 |
| DOUBLE\_STRAND\_BREAK\_REPAIR (c5) Genes annotated by the GO term GO:0006302. The repair of double-strand breaks in DNA via homologous and nonhomologous mechanisms to reform a continuous DNA helix. | 1.4408e-09 | 22/23 | 14 | 3.424 |
| NUCLEAR\_CHROMOSOME (c5) Genes annotated by the GO term GO:0000228. A chromosome found in the nucleus of a eukaryotic cell. | 2.71266e-09 | 44/53 | 19 | 5.653 |
| DNA\_REPLICATION (c5) Genes annotated by the GO term GO:0006260. The process whereby new strands of DNA are synthesized. The template for replication can either be an existing DNA molecule or RNA. | 7.62804e-09 | 94/101 | 26 | 9.403 |
| DNA\_DEPENDENT\_DNA\_REPLICATION (c5) Genes annotated by the GO term GO:0006261. The process whereby new strands of DNA are synthesized, using parental DNA as a template for the DNA-dependent DNA polymerases that synthesize the new strands. | 8.62536e-09 | 51/56 | 19 | 5.819 |
| module\_164 (c4) Genes in module\_164 | 2.49738e-08 | 56/61 | 18 | 5.383 |
| NUCLEAR\_MEMBRANE (c5) Genes annotated by the GO term GO:0031965. Either of the lipid bilayers that surround the nucleus and form the nuclear envelope; excludes the intermembrane space. | 2.53622e-08 | 45/50 | 16 | 4.684 |
| MEIOTIC\_CELL\_CYCLE (c5) Genes annotated by the GO term GO:0051321. Progression through the phases of the meiotic cell cycle, in which canonically a cell replicates to produce four offspring with half the chromosomal content of the progenitor cell. | 6.79042e-08 | 31/34 | 10 | 2.217 |
| CASPASEPATHWAY (c2) Caspases are cysteine proteases active in apoptosis; caspase-8 and 9 cleave and activate other caspases, while 3, 6, and 7 cleave cellular targets. | 7.52611e-08 | 20/21 | 16 | 4.71 |
| CASPASE\_ACTIVITY (c2) Genes related to caspase activity | 7.77763e-08 | 7/8 | 10 | 2.37 |
| H2O2\_CSBRESCUED\_C2\_UP (c2) Upregulated by H2O2 in CSB-rescued fibroblasts (Table 1, cluster 2) | 8.57481e-08 | 7/8 | 4 | 0.471 |
| GOLDRATH\_CYTOLYTIC (c2) Genes associated with cytolytic effector function induced during antigen activation of CD8+ T cells. | 1.18899e-07 | 16/18 | 12 | 3.209 |
| SODIUM\_CHANNEL\_ACTIVITY (c5) Genes annotated by the GO term GO:0005272. Catalysis of facilitated diffusion of a sodium ion (by an energy-independent process) involving passage through a transmembrane aqueous pore or channel without evidence for a carrier-mediated mechanism. | 1.95838e-07 | 13/17 | 4 | 0.491 |
| CORNIFIED\_ENVELOPE (c5) Genes annotated by the GO term GO:0001533. An insoluble protein structure formed under the plasma membrane of cornifying epithelial cells. | 4.95785e-07 | 10/13 | 4 | 0.545 |
| MITOCHONDRIAPATHWAY (c2) Pro-apoptotic signaling induces mitochondria to release cytochrome c, which stimulates Apaf-1 to activate caspase 9. | 6.018e-07 | 18/19 | 16 | 5.167 |
| DNAFRAGMENTPATHWAY (c2) DNA fragmentation during apoptosis is effected by DFF, a caspase-activated DNAse, and by endonuclease G. | 7.92036e-07 | 9/10 | 10 | 2.566 |
| NUCLEAR\_ENVELOPE (c5) Genes annotated by the GO term GO:0005635. The double lipid bilayer enclosing the nucleus and separating its contents from the rest of the cytoplasm; includes the intermembrane space, a gap of width 20-40 nm (also called the perinuclear space). | 9.01136e-07 | 66/73 | 19 | 7.046 |
| NUCLEAR\_REPLICATION\_FORK (c5) Genes annotated by the GO term GO:0043596. The Y-shaped region of a nuclear replicating DNA molecule, resulting from the separation of the DNA strands and in which the synthesis of new strands takes place. Also includes associated protein complexes. | 9.08421e-07 | 9/10 | 6 | 1.091 |
| ENVELOPE (c5) Genes annotated by the GO term GO:0031975. A multilayered structure surrounding all or part of a cell; encompasses one or more lipid bilayers, and may include a cell wall layer, also includes the space between layers. | 9.53242e-07 | 126/168 | 25 | 10.619 |
| ORGANELLE\_ENVELOPE (c5) Genes annotated by the GO term GO:0031967. A double membrane structure enclosing an organelle, including two lipid bilayers and the region between them. In some cases, an organelle envelope may have more than two membranes. | 9.53242e-07 | 126/168 | 25 | 10.619 |
| DNA\_DAMAGE\_SIGNALING (c2) Genes involved in DNA damage signaling | 1.12406e-06 | 86/89 | 31 | 14.378 |
| NUCLEOTIDE\_EXCISION\_REPAIR (c5) Genes annotated by the GO term GO:0006289. In nucleotide excision repair a small region of the strand surrounding the damage is removed from the DNA helix as an oligonucleotide. The small gap left in the DNA helix is filled in by the sequential action of DNA polymerase and DNA ligase. Nucleotide excision repair recognizes a wide range of substrates, including damage caused by UV irradiation (pyrimidine dimers and 6-4 photoproducts) and chemicals (intrastrand cross-links and bulky adducts). | 1.22492e-06 | 19/20 | 11 | 3.178 |
| SULFOTRANSFERASE\_ACTIVITY (c5) Genes annotated by the GO term GO:0008146. Catalysis of the transfer of a sulfate group from 3'-phosphoadenosine 5'-phosphosulfate to the hydroxyl group of an acceptor, producing the sulfated derivative and 3'-phosphoadenosine 5'-phosphate. | 1.55987e-06 | 10/27 | 2 | 0.159 |
| module\_281 (c4) Genes in module\_281 | 1.61477e-06 | 27/28 | 10 | 2.534 |
| DNA\_RECOMBINATION (c5) Genes annotated by the GO term GO:0006310. The processes by which a new genotype is formed by reassortment of genes resulting in gene combinations different from those that were present in the parents. In eukaryotes genetic recombination can occur by chromosome assortment, intrachromosomal recombination, or nonreciprocal interchromosomal recombination. Intrachromosomal recombination occurs by crossing over. In bacteria it may occur by genetic transformation, conjugation, transduction, or F-duction. | 2.12978e-06 | 42/47 | 16 | 5.61 |
| DAMAGED\_DNA\_BINDING (c5) Genes annotated by the GO term GO:0003684. Interacting selectively with damaged DNA. | 2.22665e-06 | 18/21 | 6 | 1.12 |
| VOLTAGE\_GATED\_SODIUM\_CHANNEL\_ACTIVITY (c5) Genes annotated by the GO term GO:0005248. Catalysis of the transmembrane transfer of a sodium ion by a voltage-gated channel. | 2.59825e-06 | 7/11 | 3 | 0.34 |
| SODIUM\_ION\_TRANSPORT (c5) Genes annotated by the GO term GO:0006814. The directed movement of sodium ions (Na+) into, out of, within or between cells. | 4.42431e-06 | 12/22 | 4 | 0.606 |
| CONDENSED\_NUCLEAR\_CHROMOSOME (c5) Genes annotated by the GO term GO:0000794. A highly compacted molecule of DNA and associated proteins resulting in a cytologically distinct structure that remains in the nucleus. | 7.01804e-06 | 14/17 | 8 | 1.939 |
| HDACI\_COLON\_SUL\_UP (c2) Upregulated by sulindac at any timepoint up to 48 hrs in SW260 colon carcinoma cells | 7.30104e-06 | 90/117 | 24 | 10.467 |
| DOUBLE\_STRANDED\_DNA\_BINDING (c5) Genes annotated by the GO term GO:0003690. Interacting selectively with double-stranded DNA. | 7.43544e-06 | 30/32 | 15 | 5.574 |
| chr15q15 (c1) Genes in cytogenetic band chr15q15 | 7.61462e-06 | 37/70 | 8 | 2.005 |
