# Supplementary material for: Looking at Cerebellar Malformations through Text-Mined Interactomes of Mice and Humans
Source: PLoS Comput Biol. 2009 Nov 6;5(11):e1000559. doi: 10.1371/journal.pcbi.1000559 (PMC2767227; doi:10.1371/journal.pcbi.1000559)
Supplement: Dataset S1 — All enrichment results. (0.20 MB ZIP) [file pcbi.1000559.s012.zip › enrichment_results/Table O. enrichment_physical-development.html]

Complete Clustering results for network physical and phenotype development (FDR <= 0.001)


# Complete Clustering results for network physical and phenotype development (FDR <= 0.001)

| Set | p-Value | Gene Count | Interaction Count | Expected Interection Count |
| --- | --- | --- | --- | --- |
| HSA04115\_P53\_SIGNALING\_PATHWAY (c2) Genes involved in p53 signaling pathway | 8.88178e-15 | 59/66 | 84 | 39.111 |
| SA\_REG\_CASCADE\_OF\_CYCLIN\_EXPR (c2) Expression of cyclins regulates progression through the cell cycle by activating cyclin-dependent kinases. | 9.10383e-15 | 12/13 | 35 | 11.209 |
| CELLCYCLEPATHWAY (c2) Cyclins interact with cyclin-dependent kinases to form active kinase complexes that regulate progression through the cell cycle. | 8.53762e-14 | 22/23 | 49 | 19.192 |
| NUCLEUS (c5) Genes annotated by the GO term GO:0005634. A membrane-bounded organelle of eukaryotic cells in which chromosomes are housed and replicated. In most cells, the nucleus contains all of the cell's chromosomes except the organellar chromosomes, and is the site of RNA synthesis and processing. In some species, or in specialized cell types, RNA metabolism or DNA replication may be absent. | 1.46438e-13 | 1255/1417 | 473 | 357.634 |
| G1\_TO\_S\_CELL\_CYCLE\_REACTOME (c2) | 8.48654e-13 | 65/66 | 81 | 39.412 |
| CELL\_CYCLE (c2) The progression of biochemical and morphological events that occur during nuclear or cellular replication. | 9.80216e-13 | 73/76 | 109 | 58.236 |
| CELL\_CYCLE\_KEGG (c2) | 1.96021e-12 | 80/84 | 116 | 64.001 |
| G1PATHWAY (c2) CDK4/6-cyclin D and CDK2-cyclin E phosphorylate Rb, which allows the transcription of genes needed for the G1/S cell cycle transition. | 4.61237e-11 | 25/26 | 64 | 30.577 |
| HSA04110\_CELL\_CYCLE (c2) Genes involved in cell cycle | 1.97819e-10 | 109/112 | 143 | 89.164 |
| BRENTANI\_CELL\_CYCLE (c2) Cancer related genes involved in the cell cycle | 2.03806e-10 | 78/79 | 81 | 43.624 |
| BRAIN\_DEVELOPMENT (c5) Genes annotated by the GO term GO:0007420. The process whose specific outcome is the progression of the brain over time, from its formation to the mature structure. The brain is one of the two components of the central nervous system and is the center of thought and emotion. It is responsible for the coordination and control of bodily activities and the interpretation of information from the senses (sight, hearing, smell, etc.). | 3.36754e-10 | 40/51 | 20 | 5.848 |
| module\_57 (c4) Genes in module\_57 | 1.34217e-09 | 54/56 | 80 | 44.01 |
| module\_98 (c4) Genes in module\_98 | 3.16234e-09 | 366/391 | 200 | 136.744 |
| EMBRYONIC\_MORPHOGENESIS (c5) Genes annotated by the GO term GO:0048598. The process by which anatomical structures are generated and organized during the embryonic phase. Morphogenesis pertains to the creation of form. The embryonic phase begins with zygote formation. The end of the embryonic phase is organism-specific. For example, it would be at birth for mammals, larval hatching for insects and seed dormancy in plants. | 3.86662e-09 | 13/17 | 11 | 2.293 |
| CELL\_CYCLE\_GO\_0007049 (c5) Genes annotated by the GO term GO:0007049. The progression of biochemical and morphological phases and events that occur in a cell during successive cell replication or nuclear replication events. Canonically, the cell cycle comprises the replication and segregation of genetic material followed by the division of the cell, but in endocycles or syncytial cells nuclear replication or nuclear division may not be followed by cell division. | 4.06881e-09 | 293/311 | 149 | 96.013 |
| HSA05218\_MELANOMA (c2) Genes involved in melanoma | 6.61596e-09 | 64/71 | 95 | 56.804 |
| SHHPATHWAY (c2) Sonic hedgehog (Shh) signaling in the developing CNS induces neuronal proliferation via interaction with the patched (Ptc-1) and smoothened receptors. | 6.80756e-09 | 12/14 | 17 | 5.043 |
| FOSBPATHWAY (c2) FOSB gene expression and drug abuse | 9.89267e-09 | 4/5 | 11 | 2.563 |
| module\_198 (c4) Genes in module\_198 | 1.48084e-08 | 284/301 | 169 | 113.863 |
| BREASTCA\_TWO\_CLASSES (c2) Gene set that can be used to differentiate BRCA1-linked and BRCA2-linked breast cancers | 2.24631e-08 | 118/132 | 96 | 56.216 |
| chr7q36 (c1) Genes in cytogenetic band chr7q36 | 2.92993e-08 | 26/68 | 13 | 3.384 |
| HSA04012\_ERBB\_SIGNALING\_PATHWAY (c2) Genes involved in ErbB signaling pathway | 5.32637e-08 | 85/87 | 137 | 91.249 |
| RACCYCDPATHWAY (c2) Ras, Rac, and Rho coordinate to induce cyclin D1 expression and activate cdk2 to promote the G1/S transition. | 7.36157e-08 | 21/22 | 67 | 38.7 |
| ST\_INTEGRIN\_SIGNALING\_PATHWAY (c2) Integrins are transmembrane receptors that mediate cell growth, survival, and migration by binding to ligands in the extracellular matrix. | 8.62325e-08 | 76/79 | 101 | 63.25 |
| module\_252 (c4) Genes in module\_252 | 8.96927e-08 | 221/235 | 140 | 93.408 |
| PROLIFERATION\_GENES (c2) Proliferation related genes | 1.39727e-07 | 333/359 | 161 | 111.284 |
| module\_197 (c4) Genes in module\_197 | 1.63876e-07 | 152/173 | 102 | 63.725 |
| HSA04340\_HEDGEHOG\_SIGNALING\_PATHWAY (c2) Genes involved in Hedgehog signaling pathway | 1.76022e-07 | 46/57 | 35 | 15.783 |
| NEGATIVE\_REGULATION\_OF\_CELLULAR\_PROCESS (c5) Genes annotated by the GO term GO:0048523. Any process that stops, prevents or reduces the frequency, rate or extent of cellular processes, those that are carried out at the cellular level, but are not necessarily restricted to a single cell. For example, cell communication occurs among more than one cell, but occurs at the cellular level. | 1.9005e-07 | 581/640 | 277 | 212.147 |
| REELINPATHWAY (c2) Reelin is secreted by neurons and recognized by receptors including cadherin related neuronal receptors, which promote phosphorylation of Dab1. | 2.3322e-07 | 6/7 | 17 | 5.704 |
| NEGATIVE\_REGULATION\_OF\_BIOLOGICAL\_PROCESS (c5) Genes annotated by the GO term GO:0048519. Any process that stops, prevents or reduces the frequency, rate or extent of a biological process. Biological processes are regulated by many means; examples include the control of gene expression, protein modification or interaction with a protein or substrate molecule. | 2.33587e-07 | 609/670 | 285 | 219.488 |
| INTERPHASE (c5) Genes annotated by the GO term GO:0051325. Progression through interphase, the stage of cell cycle between successive rounds of chromosome segregation. Canonically, interphase is the stage of the cell cycle during which the biochemical and physiologic functions of the cell are performed and replication of chromatin occurs. | 2.82487e-07 | 67/68 | 53 | 27.738 |
| INTERPHASE\_OF\_MITOTIC\_CELL\_CYCLE (c5) Genes annotated by the GO term GO:0051329. Progression through interphase, the stage of cell cycle between successive rounds of mitosis. Canonically, interphase is the stage of the cell cycle during which the biochemical and physiologic functions of the cell are performed and replication of chromatin occurs. | 2.96014e-07 | 61/62 | 48 | 24.36 |
| HSA05214\_GLIOMA (c2) Genes involved in glioma | 3.30398e-07 | 61/64 | 107 | 69.546 |
| GLAND\_DEVELOPMENT (c5) Genes annotated by the GO term GO:0048732. The process whose specific outcome is the progression of a gland over time, from its formation to the mature structure. A gland is an organ specialised for secretion. | 3.46473e-07 | 12/13 | 14 | 4.334 |
| V$LMO2COM\_02 (c3) Genes with promoter regions [-2kb,2kb] around transcription start site containing the motif NMGATANSG which matches annotation for LMO2: LIM domain only 2 (rhombotin-like 1) | 3.65205e-07 | 156/193 | 59 | 32.043 |
| SA\_G1\_AND\_S\_PHASES (c2) Cdk2, 4, and 6 bind cyclin D in G1, while cdk2/cyclin E promotes the G1/S transition. | 3.98734e-07 | 14/15 | 35 | 17.136 |
| V$E2F1\_Q3\_01 (c3) Genes with promoter regions [-2kb,2kb] around transcription start site containing the motif TTGGCGCGRAANNGNM which matches annotation for E2F1: E2F transcription factor 1 | 7.81735e-07 | 162/193 | 66 | 38.327 |
| CELL\_PROLIFERATION\_GO\_0008283 (c5) Genes annotated by the GO term GO:0008283. The multiplication or reproduction of cells, resulting in the expansion of a cell population. | 8.07451e-07 | 464/513 | 195 | 142.376 |
| DNA\_BINDING (c5) Genes annotated by the GO term GO:0003677. Interacting selectively with DNA (deoxyribonucleic acid). | 8.43729e-07 | 513/600 | 231 | 174.479 |
| DNA\_METABOLIC\_PROCESS (c5) Genes annotated by the GO term GO:0006259. The chemical reactions and pathways involving DNA, deoxyribonucleic acid, one of the two main types of nucleic acid, consisting of a long, unbranched macromolecule formed from one, or more commonly, two, strands of linked deoxyribonucleotides. | 9.85618e-07 | 236/256 | 115 | 76.146 |
| HSA04510\_FOCAL\_ADHESION (c2) Genes involved in focal adhesion | 1.01424e-06 | 183/192 | 179 | 130.581 |
| V$FAC1\_01 (c3) Genes with promoter regions [-2kb,2kb] around transcription start site containing the motif NNNCAMAACACRNA which matches annotation for FALZ: fetal Alzheimer antigen | 1.08015e-06 | 129/158 | 53 | 28.665 |
| REGULATION\_OF\_CELL\_CYCLE (c5) Genes annotated by the GO term GO:0051726. Any process that modulates the rate or extent of progression through the cell cycle. | 1.15752e-06 | 170/180 | 98 | 63.352 |
| TRANSCRIPTION (c5) Genes annotated by the GO term GO:0006350. The synthesis of either RNA on a template of DNA or DNA on a template of RNA. | 1.18714e-06 | 692/750 | 305 | 238.317 |
| RNA\_BIOSYNTHETIC\_PROCESS (c5) Genes annotated by the GO term GO:0032774. The chemical reactions and pathways resulting in the formation of RNA, ribonucleic acid, one of the two main type of nucleic acid, consisting of a long, unbranched macromolecule formed from ribonucleotides joined in 3',5'-phosphodiester linkage. Includes polymerization of ribonucleotide monomers. | 1.31272e-06 | 586/636 | 254 | 193.355 |
| PARP\_KO\_DN (c2) Downregulated in MEF cells from PARP knockout mice | 1.46831e-06 | 11/14 | 15 | 4.873 |
| ZHAN\_MULTIPLE\_MYELOMA\_VS\_NORMAL\_UP (c2) The 70 most significantly up-regulated genes in MM in comparison with normal bone marrow PCs | 1.58918e-06 | 49/60 | 35 | 16.662 |
| DNA\_DAMAGE\_SIGNALING (c2) Genes involved in DNA damage signaling | 1.60214e-06 | 86/89 | 67 | 40.045 |
| P53\_SIGNALING (c2) Genes involved in p53 signaling | 1.69098e-06 | 86/91 | 130 | 90.037 |
| METPATHWAY (c2) The hepatocyte growth factor receptor c-Met stimulates proliferation and alters cell motility and adhesion on binding the ligand HGF. | 1.77177e-06 | 34/35 | 88 | 56.683 |
| module\_403 (c4) Genes in module\_403 | 1.79881e-06 | 41/46 | 42 | 22.071 |
| TRANSCRIPTION\_\_DNA\_DEPENDENT (c5) Genes annotated by the GO term GO:0006351. The synthesis of RNA on a template of DNA. | 1.86434e-06 | 584/634 | 253 | 193.308 |
| CELL\_DEVELOPMENT (c5) Genes annotated by the GO term GO:0048468. The process whose specific outcome is the progression of the cell over time, from its formation to the mature structure. Cell development does not include the steps involved in committing a cell to a specific fate. | 1.91053e-06 | 538/571 | 275 | 216.201 |
| NERVOUS\_SYSTEM\_DEVELOPMENT (c5) Genes annotated by the GO term GO:0007399. The process whose specific outcome is the progression of nervous tissue over time, from its formation to its mature state. | 2.00218e-06 | 306/382 | 112 | 74.098 |
| SARCOMAS\_SYNOVIAL\_UP (c2) Top 20 positive significant genes associated with synovial sarcomas, versus other soft-tissue tumors. | 2.18531e-06 | 8/12 | 4 | 0.57 |
| chr20p11 (c1) Genes in cytogenetic band chr20p11 | 2.19272e-06 | 23/68 | 9 | 2.235 |
| P27PATHWAY (c2) p27 blocks the G1/S transition by inhibiting the checkpoint kinase cdk2/cyclin E and is inhibited by cdk2-mediated ubiquitination. | 2.22483e-06 | 11/12 | 23 | 9.744 |
| REGULATION\_OF\_CYCLIN\_DEPENDENT\_PROTEIN\_KINASE\_ACTIVITY (c5) Genes annotated by the GO term GO:0000079. Any process that modulates the frequency, rate or extent of CDK activity. | 2.2512e-06 | 42/43 | 30 | 13.776 |
| BREAST\_CANCER\_ESTROGEN\_SIGNALING (c2) Genes preferentially expressed in breast cancers, especially those involved in estrogen-receptor-dependent signal transduction. | 2.36118e-06 | 86/92 | 90 | 57.98 |
| GNF2\_CENPF (c4) Neighborhood of CENPF | 2.37762e-06 | 52/58 | 31 | 14.412 |
| P53PATHWAY (c2) p53 induces cell cycle arrest or apoptosis under conditions of DNA damage. | 3.41259e-06 | 15/16 | 42 | 22.616 |
| INTEGRIN\_COMPLEX (c5) Genes annotated by the GO term GO:0008305. Any member of a family of heterodimeric transmembrane receptors for cell-adhesion molecules. The alpha and beta subunits are noncovalently bonded. | 3.45198e-06 | 18/19 | 12 | 3.603 |
| NUCLEOBASE\_\_NUCLEOSIDE\_\_NUCLEOTIDE\_AND\_NUCLEIC\_ACID\_METABOLIC\_PROCESS (c5) Genes annotated by the GO term GO:0006139. The chemical reactions and pathways involving nucleobases, nucleosides, nucleotides and nucleic acids. | 3.56179e-06 | 1112/1234 | 386 | 314.577 |
| V$TFIII\_Q6 (c3) Genes with promoter regions [-2kb,2kb] around transcription start site containing the motif RGAGGKAGG which matches annotation for GTF2A1: general transcription factor IIA, 1, 19/37kDa  GTF2A2: general transcription factor IIA, 2, 12kDa | 3.78018e-06 | 133/165 | 58 | 33.598 |
| PROTEIN\_DNA\_COMPLEX\_ASSEMBLY | 4.42741e-06 | 45/49 | 29 | 13.876 |
| CORTEX\_ENRICHMENT\_LATE\_UP (c2) Up-regulated in the cortex of mice that are exposed to an enriched environmental habitat for 2 or 14 days | 4.66527e-06 | 17/20 | 15 | 5.289 |
| MENSSEN\_MYC\_UP (c2) Genes up-regulated by MYC in HUVEC (umbilical vein endothelial cell) | 4.82428e-06 | 25/28 | 30 | 14.024 |
| REGULATION\_OF\_NUCLEOBASE\_\_NUCLEOSIDE\_\_NUCLEOTIDE\_AND\_NUCLEIC\_ACID\_METABOLIC\_PROCESS (c5) Genes annotated by the GO term GO:0019219. Any process that modulates the frequency, rate or extent of the chemical reactions and pathways involving nucleobases, nucleosides, nucleotides and nucleic acids. | 5.14949e-06 | 560/614 | 258 | 200.021 |
| GROWTH\_CONE (c5) Genes annotated by the GO term GO:0030426. The migrating motile tip of a growing nerve cell axon or dendrite. | 5.26168e-06 | 9/10 | 14 | 4.781 |
| V$COMP1\_01 (c3) Genes with promoter regions [-2kb,2kb] around transcription start site containing the motif NVTNWTGATTGACNACAAVARRBN which matches annotation for MYOG: myogenin (myogenic factor 4) | 5.43205e-06 | 76/94 | 31 | 14.499 |
| BIOPOLYMER\_METABOLIC\_PROCESS (c5) Genes annotated by the GO term GO:0043283. The chemical reactions and pathways involving biopolymers, long, repeating chains of monomers found in nature e.g. polysaccharides and proteins. | 5.54868e-06 | 1493/1667 | 504 | 428.369 |
| module\_124 (c4) Genes in module\_124 | 5.60495e-06 | 91/96 | 53 | 30.838 |
| GATTGGY\_V$NFY\_Q6\_01 (c3) Genes with promoter regions [-2kb,2kb] around transcription start site containing motif GATTGGY. Motif does not match any known transcription factor | 6.61666e-06 | 657/856 | 211 | 161.158 |
| CELL\_SOMA (c5) Genes annotated by the GO term GO:0043025. The portion of a cell bearing surface projections such as axons, dendrites, cilia, or flagella that includes the nucleus, but excludes all cell projections. | 6.68199e-06 | 9/10 | 10 | 2.975 |
| APOPTOSIS\_GO (c5) Genes annotated by the GO term GO:0006915. A form of programmed cell death induced by external or internal signals that trigger the activity of proteolytic caspases, whose actions dismantle the cell and result in cell death. Apoptosis begins internally with condensation and subsequent fragmentation of the cell nucleus (blebbing) while the plasma membrane remains intact. Other characteristics of apoptosis include DNA fragmentation and the exposure of phosphatidyl serine on the cell surface. | 6.88843e-06 | 404/425 | 231 | 180.858 |
| PROGRAMMED\_CELL\_DEATH (c5) Genes annotated by the GO term GO:0012501. Cell death resulting from activation of endogenous cellular processes. | 6.90555e-06 | 405/426 | 231 | 180.878 |
| TRANSCRIPTION\_FACTOR\_ACTIVITY (c5) Genes annotated by the GO term GO:0003700. The function of binding to a specific DNA sequence in order to modulate transcription. The transcription factor may or may not also interact selectively with a protein or macromolecular complex. | 6.9407e-06 | 297/353 | 158 | 116.591 |
| HSA05220\_CHRONIC\_MYELOID\_LEUKEMIA (c2) Genes involved in chronic myeloid leukemia | 7.15044e-06 | 74/76 | 130 | 94.135 |
| TCCCRNNRTGC\_UNKNOWN (c3) Genes with promoter regions [-2kb,2kb] around transcription start site containing motif TCCCRNNRTGC. Motif does not match any known transcription factor | 7.3703e-06 | 98/132 | 49 | 27.872 |
| module\_254 (c4) Genes in module\_254 | 7.52983e-06 | 56/58 | 43 | 23.416 |
| chr13q32 (c1) Genes in cytogenetic band chr13q32 | 7.77149e-06 | 12/30 | 5 | 0.907 |
| HEMATOPOESIS\_RELATED\_TRANSCRIPTION\_FACTORS (c2) Transcription factors involved in hematopoiesis | 8.28427e-06 | 79/83 | 87 | 57.707 |
| GNF2\_PCNA (c4) Neighborhood of PCNA | 8.32402e-06 | 58/65 | 33 | 16.389 |
| PROTEIN\_COMPLEX\_BINDING (c5) Genes annotated by the GO term GO:0032403. Interacting selectively with any protein complex (a complex of two or more proteins that may include other nonprotein molecules). | 9.44552e-06 | 49/54 | 38 | 20.184 |
| INTEGRINPATHWAY (c2) Integrins are cell surface receptors commonly present at focal adhensions that interact with the extracellular matrix and transduce extracellular signaling. | 1.0886e-05 | 34/35 | 78 | 51.053 |
| ABRAHAM\_AL\_VS\_MM\_DN (c2) Genes with significantly lower average gene expression in AL plasma cells than in MM cells | 1.14418e-05 | 17/18 | 35 | 18.451 |
| VERNELL\_PRB\_CLSTR1 (c2) pRB pathway target genes CLUSTER 1 The listed genes were found regulated by pRB and p16 and one of the E2Fs (E2F1, E2F2, or E2F3) Cluster 1 genes are up-regulated by E2F and down-regulated by pRB and p16 | 1.14526e-05 | 50/61 | 29 | 14.211 |
| TRANSCRIPTION\_INITIATION (c5) Genes annotated by the GO term GO:0006352. Processes involved in the assembly of the RNA polymerase complex at the promoter region of a DNA template resulting in the subsequent synthesis of RNA from that promoter. | 1.39107e-05 | 34/35 | 22 | 9.776 |
| SKP2E2FPATHWAY (c2) E2F-1, a transcription factor that promotes the G1/S transition, is repressed by Rb and activated by cdk2/cyclin E. | 1.40128e-05 | 8/9 | 20 | 8.56 |
| REGULATION\_OF\_METABOLIC\_PROCESS (c5) Genes annotated by the GO term GO:0019222. Any process that modulates the frequency, rate or extent of the chemical reactions and pathways within a cell or an organism. | 1.48457e-05 | 724/794 | 316 | 255.147 |
| CELL\_CYCLE\_REGULATOR (c2) Obsolete by GO - was not defined before being made obsolete | 1.52814e-05 | 19/21 | 25 | 11.514 |
| NGFPATHWAY (c2) Nerve growth factor (NGF) stimulates neural survival and proliferation via the TrkA and p75 receptors, which induce DAG and IP3 production and activate Ras. | 1.69301e-05 | 17/18 | 55 | 34.124 |
| BIOPEPTIDESPATHWAY (c2) Extracellular signaling peptides exert biological effects via G-protein coupled receptors (GPCRs), which activate intracellular GTPases. | 1.70429e-05 | 37/38 | 82 | 53.92 |
