# Supplementary material for: Looking at Cerebellar Malformations through Text-Mined Interactomes of Mice and Humans
Source: PLoS Comput Biol. 2009 Nov 6;5(11):e1000559. doi: 10.1371/journal.pcbi.1000559 (PMC2767227; doi:10.1371/journal.pcbi.1000559)
Supplement: Dataset S1 — All enrichment results. (0.20 MB ZIP) [file pcbi.1000559.s012.zip › enrichment_results/Table P. enrichment_physical-small cerebellum.html]

Complete Clustering results for network physical and phenotype small cerebellum (FDR <= 0.001)


# Complete Clustering results for network physical and phenotype small cerebellum (FDR <= 0.001)

| Set | p-Value | Gene Count | Interaction Count | Expected Interection Count |
| --- | --- | --- | --- | --- |
| SA\_REG\_CASCADE\_OF\_CYCLIN\_EXPR (c2) Expression of cyclins regulates progression through the cell cycle by activating cyclin-dependent kinases. | 1.40443e-12 | 12/13 | 28 | 8.617 |
| REELINPATHWAY (c2) Reelin is secreted by neurons and recognized by receptors including cadherin related neuronal receptors, which promote phosphorylation of Dab1. | 2.24731e-12 | 6/7 | 19 | 4.621 |
| CELLCYCLEPATHWAY (c2) Cyclins interact with cyclin-dependent kinases to form active kinase complexes that regulate progression through the cell cycle. | 2.68556e-11 | 22/23 | 38 | 14.778 |
| V$E47\_01 (c3) Genes with promoter regions [-2kb,2kb] around transcription start site containing the motif VSNGCAGGTGKNCNN which matches annotation for TCF3: transcription factor 3 (E2A immunoglobulin enhancer binding factors E12/E47) | 3.56535e-11 | 155/200 | 63 | 29.146 |
| CORTEX\_ENRICHMENT\_LATE\_UP (c2) Up-regulated in the cortex of mice that are exposed to an enriched environmental habitat for 2 or 14 days | 8.56715e-11 | 17/20 | 17 | 4.284 |
| module\_275 (c4) Genes in module\_275 | 1.72104e-10 | 15/16 | 16 | 3.628 |
| HSA05214\_GLIOMA (c2) Genes involved in glioma | 2.90083e-10 | 61/64 | 96 | 54.388 |
| INTEGRIN\_COMPLEX (c5) Genes annotated by the GO term GO:0008305. Any member of a family of heterodimeric transmembrane receptors for cell-adhesion molecules. The alpha and beta subunits are noncovalently bonded. | 4.84502e-10 | 18/19 | 13 | 2.808 |
| HSA04512\_ECM\_RECEPTOR\_INTERACTION (c2) Genes involved in ECM-receptor interaction | 5.72635e-10 | 78/86 | 45 | 19.287 |
| HSA04510\_FOCAL\_ADHESION (c2) Genes involved in focal adhesion | 9.63161e-10 | 183/192 | 153 | 100.82 |
| HSA04012\_ERBB\_SIGNALING\_PATHWAY (c2) Genes involved in ErbB signaling pathway | 1.11844e-09 | 85/87 | 116 | 70.347 |
| FOSBPATHWAY (c2) FOSB gene expression and drug abuse | 1.27377e-09 | 4/5 | 11 | 2.15 |
| G1PATHWAY (c2) CDK4/6-cyclin D and CDK2-cyclin E phosphorylate Rb, which allows the transcription of genes needed for the G1/S cell cycle transition. | 1.30728e-09 | 25/26 | 51 | 25.058 |
| PROLIFERATION\_GENES (c2) Proliferation related genes | 1.01838e-08 | 333/359 | 134 | 86.338 |
| CELL\_ADHESION\_RECEPTOR\_ACTIVITY (c2) Obsolete by GO - combining with cell adhesion molecules to initiate a change in cell activity. | 1.20258e-08 | 30/33 | 21 | 6.772 |
| V$ZF5\_B (c3) Genes with promoter regions [-2kb,2kb] around transcription start site containing the motif NRNGNGCGCGCWN which matches annotation for ZFP161: zinc finger protein 161 homolog (mouse) | 1.72341e-08 | 148/188 | 45 | 20.792 |
| V$TFIII\_Q6 (c3) Genes with promoter regions [-2kb,2kb] around transcription start site containing the motif RGAGGKAGG which matches annotation for GTF2A1: general transcription factor IIA, 1, 19/37kDa  GTF2A2: general transcription factor IIA, 2, 12kDa | 2.11082e-08 | 133/165 | 53 | 26.58 |
| G1\_TO\_S\_CELL\_CYCLE\_REACTOME | 2.98472e-08 | 65/66 | 60 | 31.587 |
| EPIDERMAL\_GROWTH\_FACTOR\_RECEPTOR\_SIGNALING\_PATHWAY (c5) Genes annotated by the GO term GO:0007173. The series of molecular signals generated as a consequence of an epidermal growth factor receptor binding to one of its physiological ligands. | 5.05804e-08 | 21/22 | 35 | 15.206 |
| SHEPARD\_CELL\_PROLIFERATION (c2) Cell proliferation genes determined in zebra fish | 5.56633e-08 | 184/201 | 83 | 48.74 |
| CELL\_PROLIFERATION (c2) The multiplication or reproduction of cells, resulting in the rapid expansion of a cell population. | 5.56633e-08 | 184/201 | 83 | 48.74 |
| CELL\_PROLIFERATION\_GO\_0008283 (c5) Genes annotated by the GO term GO:0008283. The multiplication or reproduction of cells, resulting in the expansion of a cell population. | 5.86859e-08 | 464/513 | 163 | 112.53 |
| V$E2F1\_Q3\_01 (c3) Genes with promoter regions [-2kb,2kb] around transcription start site containing the motif TTGGCGCGRAANNGNM which matches annotation for E2F1: E2F transcription factor 1 | 5.86985e-08 | 162/193 | 57 | 29.822 |
| INTERPHASE (c5) Genes annotated by the GO term GO:0051325. Progression through interphase, the stage of cell cycle between successive rounds of chromosome segregation. Canonically, interphase is the stage of the cell cycle during which the biochemical and physiologic functions of the cell are performed and replication of chromatin occurs. | 6.37495e-08 | 67/68 | 45 | 21.714 |
| SKP2E2FPATHWAY (c2) E2F-1, a transcription factor that promotes the G1/S transition, is repressed by Rb and activated by cdk2/cyclin E. | 9.4734e-08 | 8/9 | 19 | 6.682 |
| BRENTANI\_CELL\_CYCLE (c2) Cancer related genes involved in the cell cycle | 1.01028e-07 | 78/79 | 64 | 35.844 |
| BREAST\_CANCER\_ESTROGEN\_SIGNALING (c2) Genes preferentially expressed in breast cancers, especially those involved in estrogen-receptor-dependent signal transduction. | 1.49825e-07 | 86/92 | 78 | 45.965 |
| module\_412 (c4) Genes in module\_412 | 1.94084e-07 | 12/13 | 13 | 3.366 |
| INTERPHASE\_OF\_MITOTIC\_CELL\_CYCLE (c5) Genes annotated by the GO term GO:0051329. Progression through interphase, the stage of cell cycle between successive rounds of mitosis. Canonically, interphase is the stage of the cell cycle during which the biochemical and physiologic functions of the cell are performed and replication of chromatin occurs. | 2.20942e-07 | 61/62 | 40 | 19.089 |
| BREASTCA\_TWO\_CLASSES (c2) Gene set that can be used to differentiate BRCA1-linked and BRCA2-linked breast cancers | 2.79791e-07 | 118/132 | 75 | 43.199 |
| module\_220 (c4) Genes in module\_220 | 2.82403e-07 | 304/329 | 97 | 60.335 |
| UNDERHILL\_PROLIFERATION (c2) Cell cycle- and proliferation-related genes underexpressed in plasma cells. | 2.88194e-07 | 16/18 | 18 | 6.14 |
| V$LMO2COM\_02 (c3) Genes with promoter regions [-2kb,2kb] around transcription start site containing the motif NMGATANSG which matches annotation for LMO2: LIM domain only 2 (rhombotin-like 1) | 3.63292e-07 | 156/193 | 49 | 25.248 |
| CELL\_CYCLE (c2) The progression of biochemical and morphological events that occur during nuclear or cellular replication. | 4.73124e-07 | 73/76 | 76 | 46.036 |
| HSA04070\_PHOSPHATIDYLINOSITOL\_SIGNALING\_SYSTEM (c2) Genes involved in phosphatidylinositol signaling system | 4.83779e-07 | 61/76 | 34 | 15.637 |
| HSA05223\_NON\_SMALL\_CELL\_LUNG\_CANCER (c2) Genes involved in non-small cell lung cancer | 5.4544e-07 | 53/54 | 78 | 47.572 |
| SARCOMAS\_SYNOVIAL\_UP (c2) Top 20 positive significant genes associated with synovial sarcomas, versus other soft-tissue tumors. | 5.6279e-07 | 8/12 | 4 | 0.508 |
| HSA04115\_P53\_SIGNALING\_PATHWAY (c2) Genes involved in p53 signaling pathway | 5.91876e-07 | 59/66 | 58 | 31.488 |
| CELL\_CYCLE\_KEGG (c2) | 7.26218e-07 | 80/84 | 82 | 50.594 |
| GRAEBER\_BETA2\_INTEGRINS (c2) Genes in the beta2 integrins family | 7.36309e-07 | 10/11 | 10 | 2.532 |
| UVB\_NHEK3\_C4 (c2) Regulated by UV-B light in normal human epidermal keratinocytes, cluster 4 | 7.40519e-07 | 9/12 | 11 | 2.915 |
| CELL\_SOMA (c5) Genes annotated by the GO term GO:0043025. The portion of a cell bearing surface projections such as axons, dendrites, cilia, or flagella that includes the nucleus, but excludes all cell projections. | 8.20807e-07 | 9/10 | 10 | 2.525 |
| P27PATHWAY (c2) p27 blocks the G1/S transition by inhibiting the checkpoint kinase cdk2/cyclin E and is inhibited by cdk2-mediated ubiquitination. | 8.60894e-07 | 11/12 | 20 | 7.6 |
| SYNAPSE (c5) Genes annotated by the GO term GO:0045202. The junction between a nerve fiber of one neuron and another neuron or muscle fiber or glial cell; the site of interneuronal communication. As the nerve fiber approaches the synapse it enlarges into a specialized structure, the presynaptic nerve ending, which contains mitochondria and synaptic vesicles. At the tip of the nerve ending is the presynaptic membrane; facing it, and separated from it by a minute cleft (the synaptic cleft) is a specialized area of membrane on the receiving cell, known as the postsynaptic membrane. In response to the arrival of nerve impulses, the presynaptic nerve ending secretes molecules of neurotransmitters into the synaptic cleft. These diffuse across the cleft and transmit the signal to the postsynaptic membrane. | 8.69804e-07 | 25/27 | 20 | 7.29 |
| EGF\_RECEPTOR\_SIGNALING\_PATHWAY (c2) EDF receptor signaling pathway | 1.06746e-06 | 12/13 | 27 | 11.796 |
| BRENTANI\_CELL\_ADHESION (c2) Cancer related genes involved in cell adhesion and metalloproteinases | 1.08027e-06 | 88/93 | 48 | 25.339 |
| BIOPEPTIDESPATHWAY (c2) Extracellular signaling peptides exert biological effects via G-protein coupled receptors (GPCRs), which activate intracellular GTPases. | 1.39733e-06 | 37/38 | 71 | 42.203 |
| chr20p11 (c1) Genes in cytogenetic band chr20p11 | 1.49571e-06 | 23/68 | 8 | 1.835 |
| MMS\_HUMAN\_LYMPH\_HIGH\_24HRS\_UP (c2) Up-regulated at 24 hours following treatment of human lymphocytes (TK6) with a high dose of methyl methanesulfonate (MMS) | 1.57411e-06 | 17/18 | 10 | 2.636 |
| EMBRYONIC\_MORPHOGENESIS (c5) Genes annotated by the GO term GO:0048598. The process by which anatomical structures are generated and organized during the embryonic phase. Morphogenesis pertains to the creation of form. The embryonic phase begins with zygote formation. The end of the embryonic phase is organism-specific. For example, it would be at birth for mammals, larval hatching for insects and seed dormancy in plants. | 1.79046e-06 | 13/17 | 8 | 1.805 |
| GROWTH\_CONE (c5) Genes annotated by the GO term GO:0030426. The migrating motile tip of a growing nerve cell axon or dendrite. | 2.47301e-06 | 9/10 | 13 | 4.063 |
| CAGGTG\_V$E12\_Q6 (c3) Genes with promoter regions [-2kb,2kb] around transcription start site containing the motif CAGGTG which matches annotation for TCF3: transcription factor 3 (E2A immunoglobulin enhancer binding factors E12/E47) | 2.8653e-06 | 1379/1832 | 296 | 237.562 |
| HSA05218\_MELANOMA (c2) Genes involved in melanoma | 3.18816e-06 | 64/71 | 73 | 44.708 |
| POSITIVE\_REGULATION\_OF\_EPITHELIAL\_CELL\_PROLIFERATION (c5) Genes annotated by the GO term GO:0050679. Any process that activates or increases the rate or extent of epithelial cell proliferation. | 4.00181e-06 | 9/10 | 14 | 4.674 |
| EMBRYONIC\_DEVELOPMENT (c5) Genes annotated by the GO term GO:0009790. The process whose specific outcome is the progression of an embryo from its formation until the end of its embryonic life stage. The end of the embryonic stage is organism-specific. For example, for mammals, the process would begin with zygote formation and end with birth. For insects, the process would begin at zygote formation and end with larval hatching. For plant zygotic embryos, this would be from zygote formation to the end of seed dormancy. For plant vegetative embryos, this would be from the initial determination of the cell or group of cells to form an embryo until the point when the embryo becomes independent of the parent plant. | 4.74529e-06 | 46/57 | 31 | 14.589 |
| HSA04340\_HEDGEHOG\_SIGNALING\_PATHWAY (c2) Genes involved in Hedgehog signaling pathway | 5.05542e-06 | 46/57 | 27 | 12.41 |
| V$AML\_Q6 (c3) Genes with promoter regions [-2kb,2kb] around transcription start site containing the motif NNGKNTGTGGTTWNC which matches annotation for RUNX1: runt-related transcription factor 1 (acute myeloid leukemia 1; aml1 oncogene) | 6.31374e-06 | 158/198 | 63 | 38.269 |
| V$SOX5\_01 (c3) Genes with promoter regions [-2kb,2kb] around transcription start site containing the motif NNAACAATNN which matches annotation for SOX5: SRY (sex determining region Y)-box 5 | 6.33747e-06 | 158/195 | 52 | 29.867 |
| LEARNING\_AND\_OR\_MEMORY (c5) Genes annotated by the GO term GO:0007611. The acquisition and processing of information and/or the storage and retrieval of this information over time. | 6.38652e-06 | 11/14 | 16 | 5.888 |
| NERVOUS\_SYSTEM\_DEVELOPMENT (c5) Genes annotated by the GO term GO:0007399. The process whose specific outcome is the progression of nervous tissue over time, from its formation to its mature state. | 6.53277e-06 | 306/382 | 90 | 58.602 |
| DENDRITE (c5) Genes annotated by the GO term GO:0030425. A branching protoplasmic process of a neuron that receive and integrate signals coming from axons of other neurons, and convey the resulting signal to the body of the cell. | 6.81473e-06 | 15/16 | 13 | 4.1 |
| PROTEIN\_COMPLEX\_BINDING (c5) Genes annotated by the GO term GO:0032403. Interacting selectively with any protein complex (a complex of two or more proteins that may include other nonprotein molecules). | 7.20734e-06 | 49/54 | 33 | 15.862 |
| RACCYCDPATHWAY (c2) Ras, Rac, and Rho coordinate to induce cyclin D1 expression and activate cdk2 to promote the G1/S transition. | 7.28997e-06 | 21/22 | 51 | 29.739 |
| chr7q36 (c1) Genes in cytogenetic band chr7q36 | 8.36464e-06 | 26/68 | 10 | 2.817 |
| V$CEBPDELTA\_Q6 (c3) Genes with promoter regions [-2kb,2kb] around transcription start site containing the motif MATTKCNTMAYY which matches annotation for CEBPD: CCAAT/enhancer binding protein (C/EBP), delta | 8.63563e-06 | 143/183 | 58 | 33.933 |
| TRANSMEMBRANE\_RECEPTOR\_PROTEIN\_TYROSINE\_KINASE\_SIGNALING\_PATHWAY (c5) Genes annotated by the GO term GO:0007169. The series of molecular signals generated as a consequence of a transmembrane receptor tyrosine kinase binding to its physiological ligand. | 1.09351e-05 | 80/83 | 72 | 44.817 |
