# Supplementary material for: Looking at Cerebellar Malformations through Text-Mined Interactomes of Mice and Humans
Source: PLoS Comput Biol. 2009 Nov 6;5(11):e1000559. doi: 10.1371/journal.pcbi.1000559 (PMC2767227; doi:10.1371/journal.pcbi.1000559)
Supplement: Dataset S1 — All enrichment results. (0.20 MB ZIP) [file pcbi.1000559.s012.zip › enrichment_results/Table Q. enrichment_whole-abnormal foliation.html]

Complete Clustering results for network whole and phenotype abnormal foliation (FDR <= 0.001)


# Complete Clustering results for network whole and phenotype abnormal foliation (FDR <= 0.001)

| Set | p-Value | Gene Count | Interaction Count | Expected Interection Count |
| --- | --- | --- | --- | --- |
| HSA05217\_BASAL\_CELL\_CARCINOMA (c2) Genes involved in basal cell carcinoma | 1e-20 | 53/55 | 92 | 41.228 |
| chr6p11 (c1) Genes in cytogenetic band chr6p11 | 1e-20 | 3/6 | 3 | 0.104 |
| HSA04340\_HEDGEHOG\_SIGNALING\_PATHWAY (c2) Genes involved in Hedgehog signaling pathway | 1e-20 | 53/57 | 106 | 34.627 |
| SHHPATHWAY (c2) Sonic hedgehog (Shh) signaling in the developing CNS induces neuronal proliferation via interaction with the patched (Ptc-1) and smoothened receptors. | 4.26326e-14 | 12/14 | 30 | 9.237 |
| HSA04115\_P53\_SIGNALING\_PATHWAY (c2) Genes involved in p53 signaling pathway | 5.82867e-14 | 64/66 | 132 | 76.055 |
| REGULATION\_OF\_CELL\_CYCLE (c5) Genes annotated by the GO term GO:0051726. Any process that modulates the rate or extent of progression through the cell cycle. | 2.20324e-12 | 174/180 | 190 | 120.67 |
| MULTICELLULAR\_ORGANISMAL\_DEVELOPMENT (c5) Genes annotated by the GO term GO:0007275. The biological process whose specific outcome is the progression of an organism over time from an initial condition (e.g. a zygote or a young adult) to a later condition (e.g. a multicellular animal or an aged adult). | 1.17046e-11 | 926/1045 | 583 | 465.388 |
| NERVOUS\_SYSTEM\_DEVELOPMENT (c5) Genes annotated by the GO term GO:0007399. The process whose specific outcome is the progression of nervous tissue over time, from its formation to its mature state. | 1.89259e-11 | 331/382 | 203 | 136.472 |
| CELL\_CYCLE\_GO\_0007049 (c5) Genes annotated by the GO term GO:0007049. The progression of biochemical and morphological phases and events that occur in a cell during successive cell replication or nuclear replication events. Canonically, the cell cycle comprises the replication and segregation of genetic material followed by the division of the cell, but in endocycles or syncytial cells nuclear replication or nuclear division may not be followed by cell division. | 2.25249e-11 | 299/311 | 244 | 167.396 |
| ANATOMICAL\_STRUCTURE\_MORPHOGENESIS (c5) Genes annotated by the GO term GO:0009653. The process by which anatomical structures are generated and organized. Morphogenesis pertains to the creation of form. | 1.43251e-10 | 345/379 | 261 | 185.379 |
| SYSTEM\_DEVELOPMENT (c5) Genes annotated by the GO term GO:0048731. The process whose specific outcome is the progression of an organismal system over time, from its formation to the mature structure. A system is a regularly interacting or interdependent group of organs or tissues that work together to carry out a given biological process. | 3.71552e-10 | 777/858 | 502 | 399.687 |
| GLAND\_DEVELOPMENT (c5) Genes annotated by the GO term GO:0048732. The process whose specific outcome is the progression of a gland over time, from its formation to the mature structure. A gland is an organ specialised for secretion. | 3.99439e-10 | 12/13 | 31 | 12.045 |
| ANATOMICAL\_STRUCTURE\_DEVELOPMENT (c5) Genes annotated by the GO term GO:0048856. The biological process whose specific outcome is the progression of an anatomical structure from an initial condition to its mature state. This process begins with the formation of the structure and ends with the mature structure, whatever form that may be including its natural destruction. An anatomical structure is any biological entity that occupies space and is distinguished from its surroundings. Anatomical structures can be macroscopic such as a carpel, or microscopic such as an acrosome. | 4.47305e-10 | 908/1012 | 548 | 442.685 |
| BRAIN\_DEVELOPMENT (c5) Genes annotated by the GO term GO:0007420. The process whose specific outcome is the progression of the brain over time, from its formation to the mature structure. The brain is one of the two components of the central nervous system and is the center of thought and emotion. It is responsible for the coordination and control of bodily activities and the interpretation of information from the senses (sight, hearing, smell, etc.). | 1.29428e-09 | 44/51 | 36 | 14.923 |
| BRENTANI\_CELL\_CYCLE (c2) Cancer related genes involved in the cell cycle | 1.45423e-09 | 78/79 | 122 | 76.944 |
| NEURITE\_DEVELOPMENT (c5) Genes annotated by the GO term GO:0031175. The process whose specific outcome is the progression of the neurite over time, from its formation to the mature structure. The neurite is any process extending from a neural cell, such as axons or dendrites. | 2.08083e-09 | 51/53 | 52 | 24.53 |
| HSA04510\_FOCAL\_ADHESION (c2) Genes involved in focal adhesion | 2.7488e-09 | 184/192 | 300 | 227.816 |
| GROWTH\_CONE (c5) Genes annotated by the GO term GO:0030426. The migrating motile tip of a growing nerve cell axon or dendrite. | 3.60936e-09 | 9/10 | 25 | 8.978 |
| CELLCYCLEPATHWAY (c2) Cyclins interact with cyclin-dependent kinases to form active kinase complexes that regulate progression through the cell cycle. | 3.91403e-09 | 22/23 | 64 | 34.589 |
| P35ALZHEIMERSPATHWAY (c2) p35, a neuron-specific activator of cyclin-dependent kinase 5, is cleaved to p25 in Alzheimer's disease and promotoes hyperphosphorylated tau formation and apoptosis. | 7.66212e-09 | 10/11 | 33 | 14.278 |
| CELL\_DEVELOPMENT (c5) Genes annotated by the GO term GO:0048468. The process whose specific outcome is the progression of the cell over time, from its formation to the mature structure. Cell development does not include the steps involved in committing a cell to a specific fate. | 9.00086e-09 | 549/571 | 511 | 419.966 |
| AXONOGENESIS (c5) Genes annotated by the GO term GO:0007409. Generation of a long process of a neuron, that carries efferent (outgoing) action potentials from the cell body towards target cells. | 2.17966e-08 | 41/43 | 40 | 18.107 |
| CELL\_FATE\_COMMITMENT (c5) Genes annotated by the GO term GO:0045165. The commitment of cells to specific cell fates and their capacity to differentiate into particular kinds of cells. Positional information is established through protein signals that emanate from a localized source within a cell (the initial one-cell zygote) or within a developmental field. | 2.30443e-08 | 11/13 | 27 | 11.132 |
| SITE\_OF\_POLARIZED\_GROWTH (c5) Genes annotated by the GO term GO:0030427. Any part of a cell where non-isotropic growth takes place. | 2.78895e-08 | 10/11 | 25 | 9.509 |
| V$FAC1\_01 (c3) Genes with promoter regions [-2kb,2kb] around transcription start site containing the motif NNNCAMAACACRNA which matches annotation for FALZ: fetal Alzheimer antigen | 2.92089e-08 | 143/158 | 91 | 54.622 |
| HSA04110\_CELL\_CYCLE (c2) Genes involved in cell cycle | 3.08697e-08 | 110/112 | 184 | 131.074 |
| RORIE\_ES\_PNET\_DN (c2) The 30 genes showing the greatest decrease in expression in NBa Ews/Fli-1 infectants | 4.84338e-08 | 24/26 | 24 | 8.746 |
| EMBRYONIC\_MORPHOGENESIS (c5) Genes annotated by the GO term GO:0048598. The process by which anatomical structures are generated and organized during the embryonic phase. Morphogenesis pertains to the creation of form. The embryonic phase begins with zygote formation. The end of the embryonic phase is organism-specific. For example, it would be at birth for mammals, larval hatching for insects and seed dormancy in plants. | 5.26926e-08 | 14/17 | 21 | 7.699 |
| WNT\_SIGNALING (c2) Wnt signaling genes | 7.20013e-08 | 58/61 | 104 | 67.06 |
| V$LEF1\_Q6 (c3) Genes with promoter regions [-2kb,2kb] around transcription start site containing the motif SWWCAAAGGG which matches annotation for LEF1: lymphoid enhancer-binding factor 1  TCF1: transcription factor 1, hepatic; LF-B1, hepatic nuclear factor (HNF1), albumin proximal factor | 9.00006e-08 | 174/212 | 75 | 43.637 |
| REGULATION\_OF\_PHOSPHORYLATION | 1.06714e-07 | 48/49 | 95 | 61.245 |
| ORGAN\_MORPHOGENESIS (c5) Genes annotated by the GO term GO:0009887. Morphogenesis of an organ. An organ is defined as a tissue or set of tissues that work together to perform a specific function or functions. Morphogenesis is the process by which anatomical structures are generated and organized. Organs are commonly observed as visibly distinct structures, but may also exist as loosely associated clusters of cells that work together to perform a specific function or functions. | 1.70781e-07 | 136/145 | 142 | 97.807 |
| CELLULAR\_MORPHOGENESIS\_DURING\_DIFFERENTIATION (c5) Genes annotated by the GO term GO:0000904. The change in form (cell shape and size) that occurs when relatively unspecialized cells, e.g. embryonic or regenerative cells, acquire specialized structural and/or functional features that characterize the cells, tissues, or organs of the mature organism or some other relatively stable phase of the organism's life history. | 2.1317e-07 | 46/49 | 41 | 19.65 |
| G1PATHWAY (c2) CDK4/6-cyclin D and CDK2-cyclin E phosphorylate Rb, which allows the transcription of genes needed for the G1/S cell cycle transition. | 2.2096e-07 | 25/26 | 88 | 55.757 |
| NEGATIVE\_REGULATION\_OF\_BIOLOGICAL\_PROCESS (c5) Genes annotated by the GO term GO:0048519. Any process that stops, prevents or reduces the frequency, rate or extent of a biological process. Biological processes are regulated by many means; examples include the control of gene expression, protein modification or interaction with a protein or substrate molecule. | 2.58254e-07 | 633/670 | 530 | 446.21 |
| ABBUD\_LIF\_DN (c2) Genes down-regulated by LIF treatment (10 ng/ml, overnight) in AtT20 cells | 3.19219e-07 | 20/24 | 15 | 4.83 |
| FOSBPATHWAY (c2) FOSB gene expression and drug abuse | 3.26946e-07 | 4/5 | 14 | 4.4 |
| POSITIVE\_REGULATION\_OF\_CELLULAR\_PROCESS (c5) Genes annotated by the GO term GO:0048522. Any process that activates or increases the frequency, rate or extent of cellular processes, those that are carried out at the cellular level, but are not necessarily restricted to a single cell. For example, cell communication occurs among more than one cell, but occurs at the cellular level. | 3.27223e-07 | 624/659 | 534 | 446.101 |
| NEURON\_DEVELOPMENT (c5) Genes annotated by the GO term GO:0048666. The process whose specific outcome is the progression of a neuron over time, from initial commitment of the cell to a specific fate, to the fully functional differentiated cell. | 3.45047e-07 | 59/61 | 54 | 28.783 |
| NEGATIVE\_REGULATION\_OF\_CELLULAR\_PROCESS (c5) Genes annotated by the GO term GO:0048523. Any process that stops, prevents or reduces the frequency, rate or extent of cellular processes, those that are carried out at the cellular level, but are not necessarily restricted to a single cell. For example, cell communication occurs among more than one cell, but occurs at the cellular level. | 3.53637e-07 | 604/640 | 510 | 428.501 |
| DNA\_BINDING (c5) Genes annotated by the GO term GO:0003677. Interacting selectively with DNA (deoxyribonucleic acid). | 3.792e-07 | 540/600 | 365 | 295.856 |
| V$PAX4\_03 (c3) Genes with promoter regions [-2kb,2kb] around transcription start site containing the motif NNNNNYCACCCB which matches annotation for PAX4: paired box gene 4 | 4.40311e-07 | 169/200 | 102 | 66.573 |
| NEURON\_DIFFERENTIATION (c5) Genes annotated by the GO term GO:0030182. The process whereby a relatively unspecialized cell acquires specialized features of a neuron. | 4.57035e-07 | 72/76 | 61 | 34.054 |
| chr20p11 (c1) Genes in cytogenetic band chr20p11 | 6.1249e-07 | 26/68 | 12 | 3.582 |
| NEUROGENESIS (c5) Genes annotated by the GO term GO:0022008. Generation of cells within the nervous system. | 6.15766e-07 | 89/93 | 70 | 41.057 |
| CENTRAL\_NERVOUS\_SYSTEM\_DEVELOPMENT (c5) Genes annotated by the GO term GO:0007417. The process whose specific outcome is the progression of the central nervous system over time, from its formation to the mature structure. The central nervous system is the core nervous system that serves an integrating and coordinating function. In vertebrates it consists of the brain, spinal cord and spinal nerves. In those invertebrates with a central nervous system it typically consists of a brain, cerebral ganglia and a nerve cord. | 6.62324e-07 | 109/123 | 64 | 37.325 |
| AXON\_GUIDANCE (c5) Genes annotated by the GO term GO:0007411. The process by which the migration of an axon growth cone is directed to a specific target site in response to a combination of attractive and repulsive cues. | 7.30489e-07 | 20/22 | 23 | 9.359 |
| TRANSCRIPTION\_FACTOR\_ACTIVITY (c5) Genes annotated by the GO term GO:0003700. The function of binding to a specific DNA sequence in order to modulate transcription. The transcription factor may or may not also interact selectively with a protein or macromolecular complex. | 7.43511e-07 | 316/353 | 270 | 211.939 |
| CATTGTYY\_V$SOX9\_B1 (c3) Genes with promoter regions [-2kb,2kb] around transcription start site containing the motif CATTGTYY which matches annotation for SOX9: SRY (sex determining region Y)-box 9 (campomelic dysplasia, autosomal sex-reversal) | 8.29917e-07 | 239/279 | 150 | 106.892 |
| GENERATION\_OF\_NEURONS (c5) Genes annotated by the GO term GO:0048699. The process by which nerve cells are generated. This includes the production of neuroblasts and their differentiation into neurons. | 8.89873e-07 | 79/83 | 65 | 37.473 |
| V$PBX1\_02 (c3) Genes with promoter regions [-2kb,2kb] around transcription start site containing the motif NNCATCAATCAANNW which matches annotation for PBX1: pre-B-cell leukemia transcription factor 1 | 8.97894e-07 | 92/106 | 79 | 49.757 |
| CELL\_CYCLE\_CHECKPOINT\_GO\_0000075 (c5) Genes annotated by the GO term GO:0000075. A point in the eukaryotic cell cycle where progress through the cycle can be halted until conditions are suitable for the cell to proceed to the next stage. | 1.04948e-06 | 45/47 | 55 | 31.594 |
| G1\_TO\_S\_CELL\_CYCLE\_REACTOME (c2) | 1.12214e-06 | 65/66 | 102 | 68.275 |
| V$LMO2COM\_01 (c3) Genes with promoter regions [-2kb,2kb] around transcription start site containing the motif CNNCAGGTGBNN which matches annotation for LMO2: LIM domain only 2 (rhombotin-like 1) | 1.26674e-06 | 159/195 | 94 | 61.193 |
| RADIATION\_SENSITIVITY (c2) Genes related to radiation sensitivity | 1.44154e-06 | 23/24 | 79 | 51.01 |
| CELL\_CYCLE (c2) The progression of biochemical and morphological events that occur during nuclear or cellular replication. | 1.61162e-06 | 73/76 | 130 | 91.656 |
| NEGATIVE\_REGULATION\_OF\_CELL\_CYCLE (c5) Genes annotated by the GO term GO:0045786. Any process that stops, prevents or reduces the rate or extent of progression through the cell cycle. | 1.6502e-06 | 74/77 | 95 | 62.575 |
| PITX2PATHWAY (c2) The bicoid-related transcription factor Pitx2 is activated by Wnt binding to the Frizzled receptor and induces tissue-specific cell proliferation. | 1.78936e-06 | 14/15 | 53 | 30.629 |
| POSITIVE\_REGULATION\_OF\_DEVELOPMENTAL\_PROCESS (c5) Genes annotated by the GO term GO:0051094. Any process that activates or increases the rate or extent of development, the biological process whose specific outcome is the progression of an organism over time from an initial condition (e.g. a zygote, or a young adult) to a later condition (e.g. a multicellular animal or an aged adult). | 1.82971e-06 | 203/215 | 203 | 153.639 |
| V$AP2\_Q3 (c3) Genes with promoter regions [-2kb,2kb] around transcription start site containing the motif GSCCSCRGGCNRNRNN which matches annotation for GTF3A: general transcription factor IIIA | 1.84243e-06 | 167/195 | 93 | 59.994 |
| PHOSPHORYLATION (c5) Genes annotated by the GO term GO:0016310. The process of introducing a phosphate group into a molecule, usually with the formation of a phosphoric ester, a phosphoric anhydride or a phosphoric amide. | 2.11974e-06 | 293/307 | 276 | 218.588 |
| REGULATION\_OF\_PROTEIN\_AMINO\_ACID\_PHOSPHORYLATION (c5) Genes annotated by the GO term GO:0001932. Any process that modulates the frequency, rate or extent of addition of phosphate groups into an amino acid in a protein. | 2.13048e-06 | 29/30 | 52 | 30.418 |
| BASSO\_GERMINAL\_CENTER\_CD40\_DN (c2) CD40 down-regulated genes | 2.52233e-06 | 58/64 | 43 | 22.877 |
| POSITIVE\_REGULATION\_OF\_BIOLOGICAL\_PROCESS (c5) Genes annotated by the GO term GO:0048518. Any process that activates or increases the frequency, rate or extent of a biological process. Biological processes are regulated by many means; examples include the control of gene expression, protein modification or interaction with a protein or substrate molecule. | 2.53598e-06 | 664/700 | 559 | 477.156 |
| SHEPARD\_CRASH\_AND\_BURN\_MUT\_VS\_WT\_DN (c2) Genes upregulated in zebra fish wild type compared to the crash and burn mutant | 2.70137e-06 | 133/150 | 69 | 41.99 |
| CELL\_CYCLE\_KEGG (c2) | 2.91662e-06 | 80/84 | 140 | 100.217 |
| WNTPATHWAY (c2) The Wnt glycoprotein binds to membrane-bound receptors such as Frizzled to activate a number of signaling pathways, including that of beta-catenin. | 3.13665e-06 | 23/24 | 62 | 38.314 |
| REGULATION\_OF\_CYCLIN\_DEPENDENT\_PROTEIN\_KINASE\_ACTIVITY (c5) Genes annotated by the GO term GO:0000079. Any process that modulates the frequency, rate or extent of CDK activity. | 3.5877e-06 | 42/43 | 47 | 26.369 |
| CORTEX\_ENRICHMENT\_LATE\_UP (c2) Up-regulated in the cortex of mice that are exposed to an enriched environmental habitat for 2 or 14 days | 4.13173e-06 | 18/20 | 19 | 7.765 |
| GGGAGGRR\_V$MAZ\_Q6 (c3) Genes with promoter regions [-2kb,2kb] around transcription start site containing the motif GGGAGGRR which matches annotation for MAZ: MYC-associated zinc finger protein (purine-binding transcription factor) | 4.2232e-06 | 1470/1733 | 669 | 580.7 |
| POSITIVE\_REGULATION\_OF\_PHOSPHATE\_METABOLIC\_PROCESS (c5) Genes annotated by the GO term GO:0045937. Any process that activates or increases the frequency, rate or extent of the chemical reactions and pathways involving phosphates. | 4.7113e-06 | 27/28 | 71 | 45.717 |
| PROLIFERATION\_GENES (c2) Proliferation related genes | 5.43702e-06 | 343/359 | 318 | 258.391 |
| NUCLEOTIDE\_METABOLISM (c2) | 5.44957e-06 | 12/14 | 9 | 2.468 |
| CELL\_CYCLE\_REGULATOR (c2) Obsolete by GO - was not defined before being made obsolete | 5.52085e-06 | 19/21 | 37 | 19.144 |
| CPR\_NULL\_LIVER\_DN (c2) Down-regulated in mouse liver tissue from mice in which NADPH-cytochrome P450 reductase (CPR) was specifically deleted in the liver by cre-lox recombination, versus lox-only controls | 5.53004e-06 | 14/18 | 10 | 2.847 |
| DNA\_DEPENDENT\_DNA\_REPLICATION (c5) Genes annotated by the GO term GO:0006261. The process whereby new strands of DNA are synthesized, using parental DNA as a template for the DNA-dependent DNA polymerases that synthesize the new strands. | 6.05073e-06 | 51/56 | 44 | 24.181 |
| BIOPOLYMER\_METABOLIC\_PROCESS (c5) Genes annotated by the GO term GO:0043283. The chemical reactions and pathways involving biopolymers, long, repeating chains of monomers found in nature e.g. polysaccharides and proteins. | 6.44238e-06 | 1549/1667 | 803 | 711.281 |
| BLEO\_HUMAN\_LYMPH\_HIGH\_4HRS\_UP (c2) Up-regulated at 4 hours following treatment of human lymphocytes (TK6) with a high dose of bleomycin | 6.63472e-06 | 19/20 | 20 | 8.335 |
| V$ATF3\_Q6 (c3) Genes with promoter regions [-2kb,2kb] around transcription start site containing the motif CBCTGACGTCANCS which matches annotation for ATF3: activating transcription factor 3 | 8.05149e-06 | 167/196 | 108 | 75.216 |
| SHH\_UP (c2) Upregulated by sonic hedgehog (shh) expression in murine neuronal precursor cells | 8.15664e-06 | 8/9 | 14 | 5.244 |
| module\_220 (c4) Genes in module\_220 | 8.64229e-06 | 316/329 | 233 | 182.111 |
| STURLA\_SONIC\_HEDGEHOG (c2) Sonic hedgehog related genes | 8.98295e-06 | 14/16 | 29 | 14.377 |
| CMV\_UV-CMV\_COMMON\_HCMV\_6HRS\_DN (c2) Down-regulated in fibroblasts at 6 hours following infection with either human cytomegalovirus (CMV) or UV-inactivated CMV | 9.6769e-06 | 22/27 | 23 | 10.491 |
| PATTERN\_SPECIFICATION\_PROCESS (c5) Genes annotated by the GO term GO:0007389. The developmental processes that result in the creation of defined areas or spaces within an organism to which cells respond and eventually are instructed to differentiate. | 9.81009e-06 | 27/31 | 38 | 20.483 |
| AAAYWAACM\_V$HFH4\_01 (c3) Genes with promoter regions [-2kb,2kb] around transcription start site containing the motif AAAYWAACM which matches annotation for FOXJ1: forkhead box J1 | 1.10646e-05 | 163/195 | 88 | 58.146 |
| REGULATION\_OF\_METABOLIC\_PROCESS (c5) Genes annotated by the GO term GO:0019222. Any process that modulates the frequency, rate or extent of the chemical reactions and pathways within a cell or an organism. | 1.12286e-05 | 751/794 | 534 | 458.199 |
| BRENTANI\_TRANSCRIPTION\_FACTORS (c2) Cancer related genes that are also transcription factors | 1.14853e-05 | 61/62 | 117 | 85.254 |
| CMV\_HCMV\_6HRS\_DN (c2) Down-regulated in fibroblasts at 6 hours following infection with human cytomegalovirus (CMV) | 1.26792e-05 | 44/51 | 35 | 18.356 |
| REELINPATHWAY (c2) Reelin is secreted by neurons and recognized by receptors including cadherin related neuronal receptors, which promote phosphorylation of Dab1. | 1.41355e-05 | 6/7 | 18 | 7.699 |
| HEMATOPOESIS\_RELATED\_TRANSCRIPTION\_FACTORS (c2) Transcription factors involved in hematopoiesis | 1.43112e-05 | 82/83 | 142 | 106.338 |
| POSITIVE\_REGULATION\_OF\_PHOSPHORYLATION (c5) Genes annotated by the GO term GO:0042327. Any process that activates or increases the frequency, rate or extent of addition of phosphate groups to a molecule. | 1.56476e-05 | 25/26 | 60 | 38.056 |
| ROTH\_HTERT\_DIFF (c2) Expression of selected genes involved in DNA repair and cell-cycle control in hTERT-transduced T cells | 1.64143e-05 | 28/29 | 67 | 44.211 |
| REGULATION\_OF\_PROTEIN\_MODIFICATION\_PROCESS (c5) Genes annotated by the GO term GO:0031399. Any process that modulates the frequency, rate or extent of the covalent alteration of one or more amino acid residues within a protein. | 1.64889e-05 | 43/44 | 69 | 45.521 |
| PROTEIN\_MODIFICATION\_PROCESS (c5) Genes annotated by the GO term GO:0006464. The covalent alteration of one or more amino acids occurring in proteins, peptides and nascent polypeptides (co-translational, post-translational modifications). Includes the modification of charged tRNAs that are destined to occur in a protein (pre-translation modification). | 1.66315e-05 | 569/623 | 341 | 282.491 |
