# Supplementary material for: Looking at Cerebellar Malformations through Text-Mined Interactomes of Mice and Humans
Source: PLoS Comput Biol. 2009 Nov 6;5(11):e1000559. doi: 10.1371/journal.pcbi.1000559 (PMC2767227; doi:10.1371/journal.pcbi.1000559)
Supplement: Dataset S1 — All enrichment results. (0.20 MB ZIP) [file pcbi.1000559.s012.zip › enrichment_results/Table R. enrichment_whole-abnormal vermis.html]

Complete Clustering results for network whole and phenotype abnormal vermis (FDR <= 0.001)


# Complete Clustering results for network whole and phenotype abnormal vermis (FDR <= 0.001)

| Set | p-Value | Gene Count | Interaction Count | Expected Interection Count |
| --- | --- | --- | --- | --- |
| module\_98 (c4) Genes in module\_98 | 1e-20 | 369/391 | 227 | 140.531 |
| HSA04340\_HEDGEHOG\_SIGNALING\_PATHWAY (c2) Genes involved in Hedgehog signaling pathway | 1e-20 | 53/57 | 59 | 23.156 |
| module\_198 (c4) Genes in module\_198 | 5.55112e-16 | 285/301 | 189 | 115.527 |
| G1\_TO\_S\_CELL\_CYCLE\_REACTOME (c2) | 1.66533e-15 | 65/66 | 88 | 43.833 |
| CELL\_CYCLE (c2) The progression of biochemical and morphological events that occur during nuclear or cellular replication. | 9.21485e-15 | 73/76 | 110 | 59.006 |
| HSA04115\_P53\_SIGNALING\_PATHWAY (c2) Genes involved in p53 signaling pathway | 9.32587e-15 | 64/66 | 96 | 51.826 |
| NUCLEUS (c5) Genes annotated by the GO term GO:0005634. A membrane-bounded organelle of eukaryotic cells in which chromosomes are housed and replicated. In most cells, the nucleus contains all of the cell's chromosomes except the organellar chromosomes, and is the site of RNA synthesis and processing. In some species, or in specialized cell types, RNA metabolism or DNA replication may be absent. | 3.01981e-14 | 1296/1417 | 481 | 366.791 |
| chr5p11 (c1) Genes in cytogenetic band chr5p11 | 7.82707e-14 | 0/1 | 1 | 0.018 |
| CELLCYCLEPATHWAY (c2) Cyclins interact with cyclin-dependent kinases to form active kinase complexes that regulate progression through the cell cycle. | 9.28146e-14 | 22/23 | 52 | 22.72 |
| module\_252 (c4) Genes in module\_252 | 3.10973e-13 | 222/235 | 158 | 97.914 |
| NUCLEOBASE\_\_NUCLEOSIDE\_\_NUCLEOTIDE\_AND\_NUCLEIC\_ACID\_METABOLIC\_PROCESS (c5) Genes annotated by the GO term GO:0006139. The chemical reactions and pathways involving nucleobases, nucleosides, nucleotides and nucleic acids. | 5.79758e-13 | 1151/1234 | 465 | 360.148 |
| BRENTANI\_CELL\_CYCLE (c2) Cancer related genes involved in the cell cycle | 1.68554e-12 | 78/79 | 92 | 50.32 |
| CELL\_CYCLE\_KEGG (c2) | 2.54963e-12 | 80/84 | 112 | 64.187 |
| CELL\_CYCLE\_GO\_0007049 (c5) Genes annotated by the GO term GO:0007049. The progression of biochemical and morphological phases and events that occur in a cell during successive cell replication or nuclear replication events. Canonically, the cell cycle comprises the replication and segregation of genetic material followed by the division of the cell, but in endocycles or syncytial cells nuclear replication or nuclear division may not be followed by cell division. | 2.94498e-12 | 299/311 | 175 | 111.568 |
| SA\_REG\_CASCADE\_OF\_CYCLIN\_EXPR (c2) Expression of cyclins regulates progression through the cell cycle by activating cyclin-dependent kinases. | 9.54992e-12 | 12/13 | 37 | 15.054 |
| module\_197 (c4) Genes in module\_197 | 1.11647e-11 | 156/173 | 127 | 76.378 |
| module\_57 (c4) Genes in module\_57 | 2.74487e-11 | 54/56 | 80 | 43.523 |
| REGULATION\_OF\_CELL\_CYCLE (c5) Genes annotated by the GO term GO:0051726. Any process that modulates the rate or extent of progression through the cell cycle. | 6.98747e-11 | 174/180 | 130 | 80.323 |
| TRANSCRIPTION (c5) Genes annotated by the GO term GO:0006350. The synthesis of either RNA on a template of DNA or DNA on a template of RNA. | 7.61009e-11 | 713/750 | 358 | 273.139 |
| module\_124 (c4) Genes in module\_124 | 2.62177e-10 | 94/96 | 64 | 32.232 |
| RNA\_BIOSYNTHETIC\_PROCESS (c5) Genes annotated by the GO term GO:0032774. The chemical reactions and pathways resulting in the formation of RNA, ribonucleic acid, one of the two main type of nucleic acid, consisting of a long, unbranched macromolecule formed from ribonucleotides joined in 3',5'-phosphodiester linkage. Includes polymerization of ribonucleotide monomers. | 2.66419e-10 | 604/636 | 305 | 227.965 |
| DNA\_BINDING (c5) Genes annotated by the GO term GO:0003677. Interacting selectively with DNA (deoxyribonucleic acid). | 2.89868e-10 | 540/600 | 273 | 198.86 |
| module\_403 (c4) Genes in module\_403 | 3.3803e-10 | 43/46 | 46 | 21.105 |
| TRANSCRIPTION\_\_DNA\_DEPENDENT (c5) Genes annotated by the GO term GO:0006351. The synthesis of RNA on a template of DNA. | 4.36475e-10 | 602/634 | 304 | 227.95 |
| HSA05217\_BASAL\_CELL\_CARCINOMA (c2) Genes involved in basal cell carcinoma | 4.88935e-10 | 53/55 | 54 | 27.309 |
| CELL\_CYCLE\_CHECKPOINT\_GO\_0000075 (c5) Genes annotated by the GO term GO:0000075. A point in the eukaryotic cell cycle where progress through the cycle can be halted until conditions are suitable for the cell to proceed to the next stage. | 9.55012e-10 | 45/47 | 44 | 20.657 |
| NUCLEAR\_PART (c5) Genes annotated by the GO term GO:0044428. Any constituent part of the nucleus, a membrane-bounded organelle of eukaryotic cells in which chromosomes are housed and replicated. | 1.7542e-09 | 527/572 | 202 | 140.93 |
| HSA04110\_CELL\_CYCLE (c2) Genes involved in cell cycle | 2.18896e-09 | 110/112 | 132 | 86.127 |
| YTAATTAA\_V$LHX3\_01 (c3) Genes with promoter regions [-2kb,2kb] around transcription start site containing the motif YTAATTAA which matches annotation for LHX3: LIM homeobox 3 | 2.74959e-09 | 119/145 | 41 | 18.133 |
| NUCLEAR\_PORE (c5) Genes annotated by the GO term GO:0005643. Any of the numerous similar discrete openings in the nuclear envelope of a eukaryotic cell, where the inner and outer nuclear membranes are joined. | 5.80631e-09 | 30/31 | 20 | 6.603 |
| DNA\_METABOLIC\_PROCESS (c5) Genes annotated by the GO term GO:0006259. The chemical reactions and pathways involving DNA, deoxyribonucleic acid, one of the two main types of nucleic acid, consisting of a long, unbranched macromolecule formed from one, or more commonly, two, strands of linked deoxyribonucleotides. | 6.72594e-09 | 243/256 | 138 | 91.627 |
| RESPONSE\_TO\_DNA\_DAMAGE\_STIMULUS (c5) Genes annotated by the GO term GO:0006974. A change in state or activity of a cell or an organism (in terms of movement, secretion, enzyme production, gene expression, etc.) as a result of a stimulus indicating damage to its DNA from environmental insults or errors during metabolism. | 7.05706e-09 | 153/161 | 90 | 53.6 |
| BRAIN\_DEVELOPMENT (c5) Genes annotated by the GO term GO:0007420. The process whose specific outcome is the progression of the brain over time, from its formation to the mature structure. The brain is one of the two components of the central nervous system and is the center of thought and emotion. It is responsible for the coordination and control of bodily activities and the interpretation of information from the senses (sight, hearing, smell, etc.). | 9.51374e-09 | 44/51 | 27 | 10.394 |
| TRANSCRIPTION\_FROM\_RNA\_POLYMERASE\_II\_PROMOTER (c5) Genes annotated by the GO term GO:0006366. The synthesis of RNA from a DNA template by RNA polymerase II (Pol II), originating at a Pol II-specific promoter. Includes transcription of messenger RNA (mRNA) and certain small nuclear RNAs (snRNAs). | 1.08211e-08 | 435/456 | 229 | 167.748 |
| SHEPARD\_CRASH\_AND\_BURN\_MUT\_VS\_WT\_DN (c2) Genes upregulated in zebra fish wild type compared to the crash and burn mutant | 1.33612e-08 | 133/150 | 55 | 28.069 |
| HSA04130\_SNARE\_INTERACTIONS\_IN\_VESICULAR\_TRANSPORT (c2) Genes involved in SNARE interactions in vesicular transport | 1.63662e-08 | 30/36 | 15 | 4.274 |
| CROONQUIST\_IL6\_STARVE\_UP (c2) Genes upregulated in multiple myeloma cells exposed to the pro-proliferative cytokine IL-6 versus those that were IL-6-starved. | 2.12906e-08 | 31/33 | 25 | 9.441 |
| module\_54 (c4) Genes in module\_54 | 2.18522e-08 | 211/255 | 82 | 47.483 |
| G1PATHWAY (c2) CDK4/6-cyclin D and CDK2-cyclin E phosphorylate Rb, which allows the transcription of genes needed for the G1/S cell cycle transition. | 2.18643e-08 | 25/26 | 63 | 35.754 |
| V$E2F\_Q6\_01 (c3) Genes with promoter regions [-2kb,2kb] around transcription start site containing the motif NKCGCGCSAAAN which matches annotation for E2F  TFDP1: transcription factor Dp-1 | 2.56491e-08 | 141/171 | 74 | 43.261 |
| NUCLEAR\_ENVELOPE | 2.5925e-08 | 68/73 | 45 | 22.271 |
| V$E2F1\_Q4\_01 (c3) Genes with promoter regions [-2kb,2kb] around transcription start site containing the motif TTTSGCGSG which matches annotation for E2F  TFDP1: transcription factor Dp-1 | 2.69394e-08 | 141/172 | 78 | 45.742 |
| GNF2\_PCNA | 2.82469e-08 | 59/65 | 36 | 16.246 |
| REGULATION\_OF\_TRANSCRIPTION (c5) Genes annotated by the GO term GO:0045449. Any process that modulates the frequency, rate or extent of the synthesis of either RNA on a template of DNA or DNA on a template of RNA. | 4.5567e-08 | 534/563 | 274 | 209.806 |
| V$E2F1\_Q6 (c3) Genes with promoter regions [-2kb,2kb] around transcription start site containing the motif TTTSGCGS which matches annotation for E2F1: E2F transcription factor 1 | 6.44082e-08 | 147/174 | 75 | 43.955 |
| REGULATION\_OF\_NUCLEOBASE\_\_NUCLEOSIDE\_\_NUCLEOTIDE\_AND\_NUCLEIC\_ACID\_METABOLIC\_PROCESS (c5) Genes annotated by the GO term GO:0019219. Any process that modulates the frequency, rate or extent of the chemical reactions and pathways involving nucleobases, nucleosides, nucleotides and nucleic acids. | 6.57162e-08 | 581/614 | 290 | 224.506 |
| V$E2F\_Q3\_01 (c3) Genes with promoter regions [-2kb,2kb] around transcription start site containing the motif TTTSGCGSG which matches annotation for E2F  TFDP1: transcription factor Dp-1 | 6.6212e-08 | 143/176 | 79 | 47.198 |
| GNF2\_CENPF | 8.63538e-08 | 52/58 | 32 | 14.341 |
| GNF2\_CDC2 (c4) Neighborhood of CDC2 | 8.70157e-08 | 52/58 | 31 | 13.721 |
| TAATTA\_V$CHX10\_01 (c3) Genes with promoter regions [-2kb,2kb] around transcription start site containing the motif TAATTA which matches annotation for VSX1: visual system homeobox 1 homolog, CHX10-like (zebrafish) | 1.2189e-07 | 485/612 | 153 | 105.926 |
| LAMB\_CYCLIN\_D3\_GLOCUS (c2) E2F target genes highly correlated with cyclin D3 expression (p = 0.002) | 1.4777e-07 | 13/15 | 13 | 3.903 |
| HSA00534\_HEPARAN\_SULFATE\_BIOSYNTHESIS (c2) Genes involved in heparan sulfate biosynthesis | 1.66542e-07 | 6/19 | 3 | 0.296 |
| V$E2F4DP1\_01 (c3) Genes with promoter regions [-2kb,2kb] around transcription start site containing the motif TTTSGCGC which matches annotation for E2F4: E2F transcription factor 4, p107/p130-binding  TFDP1: transcription factor Dp-1 | 1.83404e-07 | 150/180 | 72 | 42.805 |
| V$E2F\_Q4 | 2.80456e-07 | 144/175 | 71 | 42.031 |
| V$E2F\_Q4\_01 (c3) Genes with promoter regions [-2kb,2kb] around transcription start site containing the motif NCSCGCSAAAN which matches annotation for E2F  TFDP1: transcription factor Dp-1 | 2.81051e-07 | 144/175 | 73 | 44.093 |
| LE\_MYELIN\_UP | 3.07341e-07 | 74/87 | 49 | 26.079 |
| V$E2F\_Q6 (c3) Genes with promoter regions [-2kb,2kb] around transcription start site containing motif TTTSGCGS. Motif does not match any known transcription factor | 3.15903e-07 | 143/173 | 69 | 40.857 |
| RNA\_METABOLIC\_PROCESS (c5) Genes annotated by the GO term GO:0016070. The chemical reactions and pathways involving RNA, ribonucleic acid, one of the two main type of nucleic acid, consisting of a long, unbranched macromolecule formed from ribonucleotides joined in 3',5'-phosphodiester linkage. | 3.47589e-07 | 784/835 | 313 | 249.186 |
| BIOPOLYMER\_METABOLIC\_PROCESS (c5) Genes annotated by the GO term GO:0043283. The chemical reactions and pathways involving biopolymers, long, repeating chains of monomers found in nature e.g. polysaccharides and proteins. | 3.93104e-07 | 1549/1667 | 558 | 478.246 |
| GNF2\_RRM1 (c4) Neighborhood of RRM1 | 4.58982e-07 | 79/85 | 38 | 18.814 |
| DNA\_POLYMERASE\_ACTIVITY (c5) Genes annotated by the GO term GO:0034061. Catalysis of the reaction: deoxynucleoside triphosphate + DNA(n) = diphosphate + DNA(n+1); the synthesis of DNA from deoxyribonucleotide triphosphates in the presence of a nucleic acid template and primer. | 4.63202e-07 | 15/18 | 9 | 2.197 |
| chr10q25 (c1) Genes in cytogenetic band chr10q25 | 4.69813e-07 | 35/46 | 18 | 6.366 |
| TRANSCRIPTION\_INITIATION (c5) Genes annotated by the GO term GO:0006352. Processes involved in the assembly of the RNA polymerase complex at the promoter region of a DNA template resulting in the subsequent synthesis of RNA from that promoter. | 4.82887e-07 | 34/35 | 22 | 8.44 |
| DNA\_DAMAGE\_SIGNALING (c2) Genes involved in DNA damage signaling | 5.17032e-07 | 87/89 | 81 | 51.083 |
| V$E2F4DP2\_01 (c3) Genes with promoter regions [-2kb,2kb] around transcription start site containing the motif TTTCSCGC which matches annotation for E2F4: E2F transcription factor 4, p107/p130-binding  TFDP2: transcription factor Dp-2 (E2F dimerization partner 2) | 5.31332e-07 | 144/175 | 71 | 42.841 |
| V$E2F1DP1\_01 | 5.31332e-07 | 144/175 | 71 | 42.841 |
| V$E2F1DP2\_01 | 5.31332e-07 | 144/175 | 71 | 42.841 |
| V$E2F\_02 | 5.63874e-07 | 145/175 | 71 | 42.899 |
| GLAND\_DEVELOPMENT (c5) Genes annotated by the GO term GO:0048732. The process whose specific outcome is the progression of a gland over time, from its formation to the mature structure. A gland is an organ specialised for secretion. | 6.20685e-07 | 12/13 | 20 | 8.254 |
| BLEO\_HUMAN\_LYMPH\_HIGH\_4HRS\_UP (c2) Up-regulated at 4 hours following treatment of human lymphocytes (TK6) with a high dose of bleomycin | 6.40849e-07 | 19/20 | 16 | 5.593 |
| V$E2F\_03 (c3) Genes with promoter regions [-2kb,2kb] around transcription start site containing motif TTTSGCGCGMNR. Motif does not match any known transcription factor | 9.41597e-07 | 143/176 | 65 | 39.206 |
| MULTICELLULAR\_ORGANISMAL\_DEVELOPMENT (c5) Genes annotated by the GO term GO:0007275. The biological process whose specific outcome is the progression of an organism over time from an initial condition (e.g. a zygote or a young adult) to a later condition (e.g. a multicellular animal or an aged adult). | 1.06783e-06 | 926/1045 | 379 | 311.491 |
| GNF2\_SMC4L1 (c4) Neighborhood of SMC4L1 | 1.15947e-06 | 76/81 | 36 | 17.949 |
| ANATOMICAL\_STRUCTURE\_DEVELOPMENT (c5) Genes annotated by the GO term GO:0048856. The biological process whose specific outcome is the progression of an anatomical structure from an initial condition to its mature state. This process begins with the formation of the structure and ends with the mature structure, whatever form that may be including its natural destruction. An anatomical structure is any biological entity that occupies space and is distinguished from its surroundings. Anatomical structures can be macroscopic such as a carpel, or microscopic such as an acrosome. | 1.26538e-06 | 908/1012 | 361 | 296.124 |
| REGULATION\_OF\_TRANSCRIPTION\_\_DNA\_DEPENDENT (c5) Genes annotated by the GO term GO:0006355. Any process that modulates the frequency, rate or extent of DNA-dependent transcription. | 1.28658e-06 | 434/459 | 217 | 166.358 |
| PORE\_COMPLEX (c5) Genes annotated by the GO term GO:0046930. Any small opening in a membrane that allows the passage of gases and/or liquids. | 1.29944e-06 | 35/36 | 22 | 9.229 |
| HSA05218\_MELANOMA (c2) Genes involved in melanoma | 1.57855e-06 | 65/71 | 111 | 77.548 |
| P27PATHWAY (c2) p27 blocks the G1/S transition by inhibiting the checkpoint kinase cdk2/cyclin E and is inhibited by cdk2-mediated ubiquitination. | 1.78234e-06 | 11/12 | 23 | 10.576 |
| SKP2E2FPATHWAY (c2) E2F-1, a transcription factor that promotes the G1/S transition, is repressed by Rb and activated by cdk2/cyclin E. | 1.87956e-06 | 8/9 | 20 | 8.645 |
| VERNELL\_PRB\_CLSTR1 (c2) pRB pathway target genes CLUSTER 1 The listed genes were found regulated by pRB and p16 and one of the E2Fs (E2F1, E2F2, or E2F3) Cluster 1 genes are up-regulated by E2F and down-regulated by pRB and p16 | 1.94167e-06 | 52/61 | 31 | 14.596 |
| REGULATION\_OF\_TRANSCRIPTION\_FROM\_RNA\_POLYMERASE\_II\_PROMOTER (c5) Genes annotated by the GO term GO:0006357. Any process that modulates the frequency, rate or extent of transcription from an RNA polymerase II promoter. | 1.9509e-06 | 271/288 | 147 | 106.419 |
| POD1\_KO\_UP (c2) Up-regulated in glomeruli isolated from Pod1 knockout mice, versus wild-type controls | 1.97423e-06 | 305/369 | 113 | 77.557 |
| SGCGSSAAA\_V$E2F1DP2\_01 (c3) Genes with promoter regions [-2kb,2kb] around transcription start site containing the motif SGCGSSAAA which matches annotation for E2F1: E2F transcription factor 1  TFDP1: transcription factor Dp-1  RB1: retinoblastoma 1 (including osteosarcoma) | 2.11482e-06 | 105/127 | 51 | 29.436 |
| TRANSCRIPTION\_FACTOR\_ACTIVITY | 2.16843e-06 | 316/353 | 188 | 141.753 |
| WCAANNNYCAG\_UNKNOWN | 2.20647e-06 | 143/184 | 62 | 37.634 |
| REGULATION\_OF\_RNA\_METABOLIC\_PROCESS (c5) Genes annotated by the GO term GO:0051252. Any process that modulates the frequency, rate or extent of the chemical reactions and pathways involving RNA. | 2.2462e-06 | 442/468 | 218 | 168.171 |
| ANATOMICAL\_STRUCTURE\_MORPHOGENESIS (c5) Genes annotated by the GO term GO:0009653. The process by which anatomical structures are generated and organized. Morphogenesis pertains to the creation of form. | 2.28645e-06 | 345/379 | 167 | 123.744 |
| module\_337 (c4) Genes in module\_337 | 2.62304e-06 | 41/61 | 20 | 8.118 |
| V$POU1F1\_Q6 (c3) Genes with promoter regions [-2kb,2kb] around transcription start site containing the motif ATGAATAAWT which matches annotation for POU1F1: POU domain, class 1, transcription factor 1 (Pit1, growth hormone factor 1) | 3.01304e-06 | 157/183 | 65 | 39.709 |
| PTC1PATHWAY (c2) The binding of extracellular signaling protein Sonic hedgehog to the Patched receptor (Ptc1) allows progression through G1 and may inhibit the G2/M transition. | 3.16928e-06 | 9/10 | 20 | 8.104 |
| CENTRAL\_NERVOUS\_SYSTEM\_DEVELOPMENT (c5) Genes annotated by the GO term GO:0007417. The process whose specific outcome is the progression of the central nervous system over time, from its formation to the mature structure. The central nervous system is the core nervous system that serves an integrating and coordinating function. In vertebrates it consists of the brain, spinal cord and spinal nerves. In those invertebrates with a central nervous system it typically consists of a brain, cerebral ganglia and a nerve cord. | 3.23544e-06 | 109/123 | 46 | 25.559 |
| DNA\_REPAIR (c5) Genes annotated by the GO term GO:0006281. The process of restoring DNA after damage. Genomes are subject to damage by chemical and physical agents in the environment (e.g. UV and ionizing radiations, chemical mutagens, fungal and bacterial toxins, etc.) and by free radicals or alkylating agents endogenously generated in metabolism. DNA is also damaged because of errors during its replication. A variety of different DNA repair pathways have been reported that include direct reversal, base excision repair, nucleotide excision repair, photoreactivation, bypass, double-strand break repair pathway, and mismatch repair pathway. | 3.24013e-06 | 119/125 | 66 | 40.925 |
| GATTGGY\_V$NFY\_Q6\_01 (c3) Genes with promoter regions [-2kb,2kb] around transcription start site containing motif GATTGGY. Motif does not match any known transcription factor | 3.29995e-06 | 703/856 | 234 | 180.41 |
| CMV\_IE86\_UP (c2) Upregulated by expression of cytomegalovirus IE86 protein in primary human fibroblasts | 3.6186e-06 | 46/50 | 37 | 19.502 |
| V$TCF4\_Q5 (c3) Genes with promoter regions [-2kb,2kb] around transcription start site containing the motif SCTTTGAW which matches annotation for TCF4: transcription factor 4 | 3.6554e-06 | 157/187 | 57 | 32.8 |
| GNF2\_CCNB2 (c4) Neighborhood of CCNB2 | 3.90425e-06 | 50/55 | 29 | 14.056 |
| V$TEF\_Q6 (c3) Genes with promoter regions [-2kb,2kb] around transcription start site containing the motif ATGTTWAYATAA which matches annotation for TEF: thyrotrophic embryonic factor | 3.98416e-06 | 162/195 | 60 | 35.245 |
| SYSTEM\_DEVELOPMENT (c5) Genes annotated by the GO term GO:0048731. The process whose specific outcome is the progression of an organismal system over time, from its formation to the mature structure. A system is a regularly interacting or interdependent group of organs or tissues that work together to carry out a given biological process. | 4.16437e-06 | 777/858 | 328 | 268.092 |
| V$COMP1\_01 (c3) Genes with promoter regions [-2kb,2kb] around transcription start site containing the motif NVTNWTGATTGACNACAAVARRBN which matches annotation for MYOG: myogenin (myogenic factor 4) | 4.31783e-06 | 86/94 | 37 | 18.83 |
| REGULATION\_OF\_CELLULAR\_METABOLIC\_PROCESS (c5) Genes annotated by the GO term GO:0031323. Any process that modulates the frequency, rate or extent of the chemical reactions and pathways by which individual cells transform chemical substances. | 4.79886e-06 | 739/782 | 363 | 301.982 |
| V$PR\_Q2 (c3) Genes with promoter regions [-2kb,2kb] around transcription start site containing the motif NWNAGRACAN which matches annotation for NR3C1: nuclear receptor subfamily 3, group C, member 1 (glucocorticoid receptor) | 5.36924e-06 | 167/194 | 54 | 31.832 |
| RESPONSE\_TO\_ENDOGENOUS\_STIMULUS (c5) Genes annotated by the GO term GO:0009719. A change in state or activity of a cell or an organism (in terms of movement, secretion, enzyme production, gene expression, etc.) as a result of an endogenous stimulus. | 5.4582e-06 | 187/198 | 94 | 62.824 |
| HDACI\_COLON\_TSABUT\_DN (c2) Downregulated by both butyrate and TSA at any timepoint up to 48 hrs in SW260 colon carcinoma cells | 5.59016e-06 | 14/19 | 18 | 7.535 |
| SASAKI\_TCELL\_LYMPHOMA\_VS\_CD4\_UP (c2) Genes overexpressed at least twofold in adult T-cell lymphoma (ATL) cells versus normal CD4+ and CD4+/CD45RO+ T cells. | 5.96222e-06 | 143/161 | 83 | 55.761 |
| SASAKI\_ATL\_UP (c2) Highly expressed genes in ATL cells compared with normal CD4 and CD4 CD45RO T cells | 5.96222e-06 | 143/161 | 83 | 55.761 |
| GH\_EXOGENOUS\_EARLY\_DN (c2) Down-regulated at early time points (1-4 hours) following treatment of mammary carcinoma cells (MCF-7) with exogenous human growth hormone | 6.12616e-06 | 6/8 | 7 | 1.726 |
| SHEPARD\_BMYB\_MORPHOLINO\_DN (c2) Genes upregulated in control vs bmyb morpholino knockdown in zebra fish | 6.17759e-06 | 152/165 | 56 | 33.014 |
| V$LEF1\_Q6 (c3) Genes with promoter regions [-2kb,2kb] around transcription start site containing the motif SWWCAAAGGG which matches annotation for LEF1: lymphoid enhancer-binding factor 1  TCF1: transcription factor 1, hepatic; LF-B1, hepatic nuclear factor (HNF1), albumin proximal factor | 6.97281e-06 | 174/212 | 51 | 28.729 |
| ORGANELLE\_ENVELOPE | 7.15665e-06 | 137/168 | 57 | 34.308 |
| ENVELOPE (c5) Genes annotated by the GO term GO:0031975. A multilayered structure surrounding all or part of a cell; encompasses one or more lipid bilayers, and may include a cell wall layer, also includes the space between layers. | 7.15665e-06 | 137/168 | 57 | 34.308 |
| PROTEIN\_DNA\_COMPLEX\_ASSEMBLY (c5) Genes annotated by the GO term GO:0065004. The aggregation, arrangement and bonding together of proteins and DNA molecules to form a protein-DNA complex. | 7.28975e-06 | 45/49 | 26 | 11.915 |
| module\_495 (c4) Genes in module\_495 | 7.59731e-06 | 8/16 | 4 | 0.607 |
| V$E2F1\_Q4 (c3) Genes with promoter regions [-2kb,2kb] around transcription start site containing the motif NTTSGCGG which matches annotation for E2F1: E2F transcription factor 1 | 7.8611e-06 | 156/182 | 83 | 54.971 |
| LEE\_TCELLS9\_UP (c2) Transcripts showing SP4>CB4>AB4 pattern | 8.04572e-06 | 20/25 | 12 | 3.817 |
| module\_18 (c4) Genes in module\_18 | 8.11677e-06 | 402/447 | 158 | 118.397 |
| GNF2\_CENPE (c4) Neighborhood of CENPE | 8.23102e-06 | 35/39 | 24 | 11.2 |
| CELL\_CYCLE\_PROCESS (c5) Genes annotated by the GO term GO:0022402. A cellular process that is involved in the progression of biochemical and morphological phases and events that occur in a cell during successive cell replication or nuclear replication events. | 9.36541e-06 | 182/191 | 94 | 64.137 |
| NUCLEOTIDE\_METABOLISM (c2) | 9.39931e-06 | 12/14 | 7 | 1.666 |
| SULFURIC\_ESTER\_HYDROLASE\_ACTIVITY (c5) Genes annotated by the GO term GO:0008484. Catalysis of the reaction: RSO-R' + H2O = RSOOH + R'H. This reaction is the hydrolysis of any sulfuric ester bond, any ester formed from sulfuric acid, O=SO(OH)2. | 1.03582e-05 | 9/16 | 4 | 0.677 |
| RUIZ\_TENASCIN\_TARGETS (c2) Tenascin-C target genes | 1.04393e-05 | 76/77 | 53 | 32.135 |
| REGULATION\_OF\_METABOLIC\_PROCESS (c5) Genes annotated by the GO term GO:0019222. Any process that modulates the frequency, rate or extent of the chemical reactions and pathways within a cell or an organism. | 1.07137e-05 | 751/794 | 368 | 308.478 |
| NEGATIVE\_REGULATION\_OF\_CELLULAR\_PROCESS (c5) Genes annotated by the GO term GO:0048523. Any process that stops, prevents or reduces the frequency, rate or extent of cellular processes, those that are carried out at the cellular level, but are not necessarily restricted to a single cell. For example, cell communication occurs among more than one cell, but occurs at the cellular level. | 1.12641e-05 | 604/640 | 341 | 285.766 |
| WNT\_TARGETS (c2) WNT target genes from literatures | 1.24325e-05 | 21/22 | 35 | 19.62 |
| MMS\_MOUSE\_LYMPH\_HIGH\_4HRS\_UP (c2) Up-regulated at 4 hours following treatment of mouse lymphocytes (TK 3.7.2C) with a high dose of methyl methanesulfonate (MMS) | 1.2882e-05 | 30/34 | 15 | 5.705 |
| GNF2\_H2AFX (c4) Neighborhood of H2AFX | 1.30279e-05 | 25/30 | 22 | 10.349 |
| NUCLEAR\_MEMBRANE\_PART (c5) Genes annotated by the GO term GO:0044453. Any constituent part of the nuclear membrane, the envelope that surrounds the nucleus of eukaryotic cells. | 1.40684e-05 | 40/42 | 22 | 9.968 |
| V$GR\_Q6\_01 (c3) Genes with promoter regions [-2kb,2kb] around transcription start site containing the motif NNTGTYCT which matches annotation for NR3C1: nuclear receptor subfamily 3, group C, member 1 (glucocorticoid receptor) | 1.49915e-05 | 166/196 | 62 | 38.785 |
| CELL\_CYCLE\_PHASE (c5) Genes annotated by the GO term GO:0022403. A cell cycle process comprising the steps by which a cell progresses through one of the biochemical and morphological phases and events that occur during successive cell replication or nuclear replication events. | 1.5703e-05 | 163/169 | 89 | 60.856 |
| module\_308 (c4) Genes in module\_308 | 1.99543e-05 | 58/70 | 29 | 14.533 |
| PATTERN\_SPECIFICATION\_PROCESS (c5) Genes annotated by the GO term GO:0007389. The developmental processes that result in the creation of defined areas or spaces within an organism to which cells respond and eventually are instructed to differentiate. | 2.08799e-05 | 27/31 | 27 | 14.109 |
| NEGATIVE\_REGULATION\_OF\_CELL\_CYCLE (c5) Genes annotated by the GO term GO:0045786. Any process that stops, prevents or reduces the rate or extent of progression through the cell cycle. | 2.16471e-05 | 74/77 | 64 | 41.343 |
| EMBRYONIC\_MORPHOGENESIS (c5) Genes annotated by the GO term GO:0048598. The process by which anatomical structures are generated and organized during the embryonic phase. Morphogenesis pertains to the creation of form. The embryonic phase begins with zygote formation. The end of the embryonic phase is organism-specific. For example, it would be at birth for mammals, larval hatching for insects and seed dormancy in plants. | 2.20358e-05 | 14/17 | 14 | 5.54 |
| TRANSCRIPTION\_INITIATION\_FROM\_RNA\_POLYMERASE\_II\_PROMOTER (c5) Genes annotated by the GO term GO:0006367. Processes involved in starting transcription from the RNA polymerase II promoter. | 2.26782e-05 | 28/29 | 19 | 7.911 |
| MORF\_BUB1B (c4) Neighborhood of BUB1B | 2.34227e-05 | 59/65 | 27 | 12.885 |
| SA\_G1\_AND\_S\_PHASES (c2) Cdk2, 4, and 6 bind cyclin D in G1, while cdk2/cyclin E promotes the G1/S transition. | 2.42994e-05 | 14/15 | 37 | 21.676 |
