# Supplementary material for: Looking at Cerebellar Malformations through Text-Mined Interactomes of Mice and Humans
Source: PLoS Comput Biol. 2009 Nov 6;5(11):e1000559. doi: 10.1371/journal.pcbi.1000559 (PMC2767227; doi:10.1371/journal.pcbi.1000559)
Supplement: Dataset S1 — All enrichment results. (0.20 MB ZIP) [file pcbi.1000559.s012.zip › enrichment_results/Table S. enrichment_whole-absent cerebellum.html]

Complete Clustering results for network whole and phenotype absent cerebellum (FDR <= 0.001)


# Complete Clustering results for network whole and phenotype absent cerebellum (FDR <= 0.001)

| Set | p-Value | Gene Count | Interaction Count | Expected Interection Count |
| --- | --- | --- | --- | --- |
| V$TST1\_01 (c3) Genes with promoter regions [-2kb,2kb] around transcription start site containing the motif NNKGAATTAVAVTDN which matches annotation for POU3F1: POU domain, class 3, transcription factor 1 | 1e-20 | 163/204 | 39 | 11.474 |
| YTAATTAA\_V$LHX3\_01 (c3) Genes with promoter regions [-2kb,2kb] around transcription start site containing the motif YTAATTAA which matches annotation for LHX3: LIM homeobox 3 | 1e-20 | 119/145 | 25 | 4.471 |
| CTTTGA\_V$LEF1\_Q2 (c3) Genes with promoter regions [-2kb,2kb] around transcription start site containing the motif CTTTGA which matches annotation for LEF1: lymphoid enhancer-binding factor 1 | 1e-20 | 738/901 | 98 | 39.983 |
| V$PAX4\_02 (c3) Genes with promoter regions [-2kb,2kb] around transcription start site containing the motif NAAWAATTANS which matches annotation for PAX4: paired box gene 4 | 1e-20 | 155/191 | 35 | 8.388 |
| V$HNF6\_Q6 (c3) Genes with promoter regions [-2kb,2kb] around transcription start site containing the motif HWAAATCAATAW which matches annotation for ONECUT1: one cut domain, family member 1 | 1e-20 | 150/183 | 45 | 14.67 |
| V$CHX10\_01 (c3) Genes with promoter regions [-2kb,2kb] around transcription start site containing the motif NNNTAATTAGCNNN which matches annotation for VSX1: visual system homeobox 1 homolog, CHX10-like (zebrafish) | 1e-20 | 149/184 | 44 | 10.616 |
| ORGAN\_DEVELOPMENT (c5) Genes annotated by the GO term GO:0048513. Development of a tissue or tissues that work together to perform a specific function or functions. Development pertains to the process whose specific outcome is the progression of a structure over time, from its formation to the mature structure. Organs are commonly observed as visibly distinct structures, but may also exist as loosely associated clusters of cells that work together to perform a specific function or functions. | 1e-20 | 530/569 | 114 | 57.111 |
| RUIZ\_TENASCIN\_TARGETS (c2) Tenascin-C target genes | 1e-20 | 76/77 | 31 | 8.52 |
| CENTRAL\_NERVOUS\_SYSTEM\_DEVELOPMENT (c5) Genes annotated by the GO term GO:0007417. The process whose specific outcome is the progression of the central nervous system over time, from its formation to the mature structure. The central nervous system is the core nervous system that serves an integrating and coordinating function. In vertebrates it consists of the brain, spinal cord and spinal nerves. In those invertebrates with a central nervous system it typically consists of a brain, cerebral ganglia and a nerve cord. | 1e-20 | 109/123 | 38 | 6.271 |
| PS1PATHWAY (c2) Presenilin is required for gamma-secretase activity to activate Notch signaling; presenilin also inhibits beta-catenin in the Wnt/Frizzled pathway. | 1e-20 | 11/12 | 18 | 3.095 |
| TAATTA\_V$CHX10\_01 (c3) Genes with promoter regions [-2kb,2kb] around transcription start site containing the motif TAATTA which matches annotation for VSX1: visual system homeobox 1 homolog, CHX10-like (zebrafish) | 1e-20 | 485/612 | 85 | 27.667 |
| CTTTGT\_V$LEF1\_Q2 (c3) Genes with promoter regions [-2kb,2kb] around transcription start site containing the motif CTTTGT which matches annotation for LEF1: lymphoid enhancer-binding factor 1 | 1e-20 | 1197/1460 | 150 | 76.52 |
| EMBRYONIC\_DEVELOPMENT (c5) Genes annotated by the GO term GO:0009790. The process whose specific outcome is the progression of an embryo from its formation until the end of its embryonic life stage. The end of the embryonic stage is organism-specific. For example, for mammals, the process would begin with zygote formation and end with birth. For insects, the process would begin at zygote formation and end with larval hatching. For plant zygotic embryos, this would be from zygote formation to the end of seed dormancy. For plant vegetative embryos, this would be from the initial determination of the cell or group of cells to form an embryo until the point when the embryo becomes independent of the parent plant. | 1e-20 | 47/57 | 26 | 5.787 |
| ANATOMICAL\_STRUCTURE\_DEVELOPMENT (c5) Genes annotated by the GO term GO:0048856. The biological process whose specific outcome is the progression of an anatomical structure from an initial condition to its mature state. This process begins with the formation of the structure and ends with the mature structure, whatever form that may be including its natural destruction. An anatomical structure is any biological entity that occupies space and is distinguished from its surroundings. Anatomical structures can be macroscopic such as a carpel, or microscopic such as an acrosome. | 1e-20 | 908/1012 | 171 | 81.29 |
| V$TCF4\_Q5 (c3) Genes with promoter regions [-2kb,2kb] around transcription start site containing the motif SCTTTGAW which matches annotation for TCF4: transcription factor 4 | 1e-20 | 157/187 | 31 | 8.212 |
| V$LHX3\_01 (c3) Genes with promoter regions [-2kb,2kb] around transcription start site containing the motif AATTAATTAA which matches annotation for LHX3: LIM homeobox 3 | 1e-20 | 151/183 | 30 | 7.663 |
| SYSTEM\_DEVELOPMENT (c5) Genes annotated by the GO term GO:0048731. The process whose specific outcome is the progression of an organismal system over time, from its formation to the mature structure. A system is a regularly interacting or interdependent group of organs or tissues that work together to carry out a given biological process. | 1e-20 | 777/858 | 145 | 74.11 |
| AXON\_GUIDANCE (c5) Genes annotated by the GO term GO:0007411. The process by which the migration of an axon growth cone is directed to a specific target site in response to a combination of attractive and repulsive cues. | 1e-20 | 20/22 | 12 | 1.689 |
| NERVOUS\_SYSTEM\_DEVELOPMENT (c5) Genes annotated by the GO term GO:0007399. The process whose specific outcome is the progression of nervous tissue over time, from its formation to its mature state. | 1e-20 | 331/382 | 68 | 24.821 |
| PATTERN\_SPECIFICATION\_PROCESS (c5) Genes annotated by the GO term GO:0007389. The developmental processes that result in the creation of defined areas or spaces within an organism to which cells respond and eventually are instructed to differentiate. | 1e-20 | 27/31 | 23 | 3.89 |
| ANATOMICAL\_STRUCTURE\_MORPHOGENESIS (c5) Genes annotated by the GO term GO:0009653. The process by which anatomical structures are generated and organized. Morphogenesis pertains to the creation of form. | 1e-20 | 345/379 | 86 | 33.971 |
| MULTICELLULAR\_ORGANISMAL\_DEVELOPMENT (c5) Genes annotated by the GO term GO:0007275. The biological process whose specific outcome is the progression of an organism over time from an initial condition (e.g. a zygote or a young adult) to a later condition (e.g. a multicellular animal or an aged adult). | 1e-20 | 926/1045 | 161 | 86.667 |
| BRAIN\_DEVELOPMENT (c5) Genes annotated by the GO term GO:0007420. The process whose specific outcome is the progression of the brain over time, from its formation to the mature structure. The brain is one of the two components of the central nervous system and is the center of thought and emotion. It is responsible for the coordination and control of bodily activities and the interpretation of information from the senses (sight, hearing, smell, etc.). | 1e-20 | 44/51 | 25 | 2.618 |
| HSA05217\_BASAL\_CELL\_CARCINOMA (c2) Genes involved in basal cell carcinoma | 1e-20 | 53/55 | 59 | 7.865 |
| HSA04340\_HEDGEHOG\_SIGNALING\_PATHWAY (c2) Genes involved in Hedgehog signaling pathway | 1e-20 | 53/57 | 44 | 6.533 |
| HSA04310\_WNT\_SIGNALING\_PATHWAY (c2) Genes involved in Wnt signaling pathway | 1e-20 | 138/147 | 83 | 30.183 |
| HSA00534\_HEPARAN\_SULFATE\_BIOSYNTHESIS (c2) Genes involved in heparan sulfate biosynthesis | 1e-20 | 6/19 | 5 | 0.079 |
| EMBRYONIC\_MORPHOGENESIS (c5) Genes annotated by the GO term GO:0048598. The process by which anatomical structures are generated and organized during the embryonic phase. Morphogenesis pertains to the creation of form. The embryonic phase begins with zygote formation. The end of the embryonic phase is organism-specific. For example, it would be at birth for mammals, larval hatching for insects and seed dormancy in plants. | 1e-20 | 14/17 | 13 | 1.431 |
| V$MEIS1AHOXA9\_01 (c3) Genes with promoter regions [-2kb,2kb] around transcription start site containing the motif TGACAGKTTTAYGA which matches annotation for MEIS1: Meis1, myeloid ecotropic viral integration site 1 homolog (mouse)  HOXA9: homeobox A9 | 1e-20 | 77/93 | 22 | 4.72 |
| V$NKX62\_Q2 | 1e-20 | 151/186 | 36 | 9.712 |
| V$TEF\_Q6 (c3) Genes with promoter regions [-2kb,2kb] around transcription start site containing the motif ATGTTWAYATAA which matches annotation for TEF: thyrotrophic embryonic factor | 1e-20 | 162/195 | 34 | 9.245 |
| V$IPF1\_Q4 (c3) Genes with promoter regions [-2kb,2kb] around transcription start site containing the motif GHNNTAATGACM which matches annotation for IPF1: insulin promoter factor 1, homeodomain transcription factor | 1e-20 | 159/186 | 35 | 9.41 |
| V$LEF1\_Q6 (c3) Genes with promoter regions [-2kb,2kb] around transcription start site containing the motif SWWCAAAGGG which matches annotation for LEF1: lymphoid enhancer-binding factor 1  TCF1: transcription factor 1, hepatic; LF-B1, hepatic nuclear factor (HNF1), albumin proximal factor | 1e-20 | 174/212 | 30 | 7.051 |
| ALKPATHWAY | 1.11022e-16 | 31/32 | 31 | 7.93 |
| V$HNF3ALPHA\_Q6 (c3) Genes with promoter regions [-2kb,2kb] around transcription start site containing the motif TRTTTGYTYWN which matches annotation for FOXA1: forkhead box A1 | 2.22045e-16 | 141/160 | 30 | 7.658 |
| V$PIT1\_Q6 (c3) Genes with promoter regions [-2kb,2kb] around transcription start site containing the motif NMTTCATAAWTATWNMNA which matches annotation for POU1F1: POU domain, class 1, transcription factor 1 (Pit1, growth hormone factor 1) | 2.22045e-16 | 148/180 | 33 | 9.488 |
| YATTNATC\_UNKNOWN (c3) Genes with promoter regions [-2kb,2kb] around transcription start site containing motif YATTNATC. Motif does not match any known transcription factor | 3.33067e-16 | 235/281 | 46 | 15.341 |
| AACTTT\_UNKNOWN (c3) Genes with promoter regions [-2kb,2kb] around transcription start site containing motif AACTTT. Motif does not match any known transcription factor | 3.33067e-16 | 1218/1458 | 160 | 92.289 |
| WNTPATHWAY (c2) The Wnt glycoprotein binds to membrane-bound receptors such as Frizzled to activate a number of signaling pathways, including that of beta-catenin. | 4.44089e-16 | 23/24 | 28 | 7.437 |
| V$OCT1\_03 (c3) Genes with promoter regions [-2kb,2kb] around transcription start site containing the motif NNNRTAATNANNN which matches annotation for POU2F1: POU domain, class 2, transcription factor 1 | 7.77156e-16 | 143/167 | 32 | 8.821 |
| V$GATA6\_01 (c3) Genes with promoter regions [-2kb,2kb] around transcription start site containing the motif NNNGATWANN which matches annotation for GATA6: GATA binding protein 6 | 7.77156e-16 | 164/194 | 31 | 8.47 |
| GH\_EXOGENOUS\_EARLY\_DN (c2) Down-regulated at early time points (1-4 hours) following treatment of mammary carcinoma cells (MCF-7) with exogenous human growth hormone | 8.88178e-16 | 6/8 | 6 | 0.497 |
| V$POU1F1\_Q6 (c3) Genes with promoter regions [-2kb,2kb] around transcription start site containing the motif ATGAATAAWT which matches annotation for POU1F1: POU domain, class 1, transcription factor 1 (Pit1, growth hormone factor 1) | 1.9984e-15 | 157/183 | 35 | 10.636 |
| module\_220 (c4) Genes in module\_220 | 4.88498e-15 | 316/329 | 73 | 33.098 |
| chr7q36 (c1) Genes in cytogenetic band chr7q36 | 7.99361e-15 | 31/68 | 10 | 1.436 |
| WNT\_SIGNALING (c2) Wnt signaling genes | 8.21565e-15 | 58/61 | 38 | 13.161 |
| V$COMP1\_01 (c3) Genes with promoter regions [-2kb,2kb] around transcription start site containing the motif NVTNWTGATTGACNACAAVARRBN which matches annotation for MYOG: myogenin (myogenic factor 4) | 9.32587e-15 | 86/94 | 21 | 4.967 |
| V$CDP\_02 (c3) Genes with promoter regions [-2kb,2kb] around transcription start site containing the motif NWNATCGATTANYNN which matches annotation for CUTL1: cut-like 1, CCAAT displacement protein (Drosophila) | 1.18794e-14 | 74/86 | 21 | 4.895 |
| REGIONALIZATION (c5) Genes annotated by the GO term GO:0003002. The pattern specification process by which an axis or axes is subdivided in space to define an area or volume in which specific patterns of cell differentiation will take place or in which cells interpret a specific environment. | 1.31006e-14 | 12/15 | 12 | 1.831 |
| 1\_AND\_2\_METHYLNAPHTHALENE\_DEGRADATION (c2) | 2.28706e-14 | 4/7 | 2 | 0.066 |
| V$PBX1\_02 (c3) Genes with promoter regions [-2kb,2kb] around transcription start site containing the motif NNCATCAATCAANNW which matches annotation for PBX1: pre-B-cell leukemia transcription factor 1 | 2.68674e-14 | 92/106 | 30 | 8.845 |
| SKELETAL\_DEVELOPMENT (c5) Genes annotated by the GO term GO:0001501. The process whose specific outcome is the progression of the skeleton over time, from its formation to the mature structure. The skeleton is the bony framework of the body in vertebrates (endoskeleton) or the hard outer envelope of insects (exoskeleton or dermoskeleton). | 6.43929e-14 | 95/102 | 31 | 9.586 |
| V$CDPCR3\_01 (c3) Genes with promoter regions [-2kb,2kb] around transcription start site containing the motif CACCRATANNTATBG which matches annotation for CUTL1: cut-like 1, CCAAT displacement protein (Drosophila) | 1.21236e-13 | 32/39 | 14 | 2.718 |
| ST\_WNT\_BETA\_CATENIN\_PATHWAY (c2) Beta-catenin is degraded in the absence of Wnt signaling; when extracellular Wnt binds Frizzled receptors, beta-catenin accumulates in the nucleus and may promote cell survival. | 3.94018e-13 | 29/31 | 21 | 5.764 |
| HEART\_DEVELOPMENT (c5) Genes annotated by the GO term GO:0007507. The process whose specific outcome is the progression of the heart over time, from its formation to the mature structure. The heart is a hollow, muscular organ, which, by contracting rhythmically, keeps up the circulation of the blood. | 8.31557e-13 | 32/37 | 17 | 3.72 |
| CELL\_FATE\_COMMITMENT (c5) Genes annotated by the GO term GO:0045165. The commitment of cells to specific cell fates and their capacity to differentiate into particular kinds of cells. Positional information is established through protein signals that emanate from a localized source within a cell (the initial one-cell zygote) or within a developmental field. | 1.00508e-12 | 11/13 | 12 | 2.213 |
| AXONOGENESIS (c5) Genes annotated by the GO term GO:0007409. Generation of a long process of a neuron, that carries efferent (outgoing) action potentials from the cell body towards target cells. | 1.27254e-12 | 41/43 | 15 | 3.15 |
| V$GR\_Q6\_01 (c3) Genes with promoter regions [-2kb,2kb] around transcription start site containing the motif NNTGTYCT which matches annotation for NR3C1: nuclear receptor subfamily 3, group C, member 1 (glucocorticoid receptor) | 1.68643e-12 | 166/196 | 32 | 10.49 |
| WCAANNNYCAG\_UNKNOWN (c3) Genes with promoter regions [-2kb,2kb] around transcription start site containing motif WCAANNNYCAG. Motif does not match any known transcription factor | 1.77602e-12 | 143/184 | 32 | 10.108 |
| module\_234 (c4) Genes in module\_234 | 4.1448e-12 | 49/54 | 27 | 9.128 |
| V$HNF3B\_01 (c3) Genes with promoter regions [-2kb,2kb] around transcription start site containing the motif KGNANTRTTTRYTTW which matches annotation for FOXA2: forkhead box A2 | 5.72686e-12 | 139/171 | 30 | 10.76 |
| TTGTTT\_V$FOXO4\_01 (c3) Genes with promoter regions [-2kb,2kb] around transcription start site containing the motif TTGTTT which matches annotation for MLLT7: myeloid/lymphoid or mixed-lineage leukemia (trithorax homolog, Drosophila); translocated to, 7 | 8.88534e-12 | 1248/1511 | 136 | 82.247 |
| SARCOMAS\_LIPOSARCOMA\_DN (c2) Top 20 negative significant genes associated with liposarcomas, versus other soft-tissue tumors. | 1.1199e-11 | 9/11 | 4 | 0.31 |
| V$HFH1\_01 (c3) Genes with promoter regions [-2kb,2kb] around transcription start site containing the motif NATTGTTTATWT which matches annotation for FOXQ1: forkhead box Q1 | 1.75814e-11 | 159/186 | 27 | 8.384 |
| AAAYWAACM\_V$HFH4\_01 (c3) Genes with promoter regions [-2kb,2kb] around transcription start site containing the motif AAAYWAACM which matches annotation for FOXJ1: forkhead box J1 | 2.3413e-11 | 163/195 | 30 | 9.637 |
| YCATTAA\_UNKNOWN (c3) Genes with promoter regions [-2kb,2kb] around transcription start site containing motif YCATTAA. Motif does not match any known transcription factor | 2.54322e-11 | 352/433 | 43 | 16.815 |
| CELLULAR\_MORPHOGENESIS\_DURING\_DIFFERENTIATION (c5) Genes annotated by the GO term GO:0000904. The change in form (cell shape and size) that occurs when relatively unspecialized cells, e.g. embryonic or regenerative cells, acquire specialized structural and/or functional features that characterize the cells, tissues, or organs of the mature organism or some other relatively stable phase of the organism's life history. | 2.58076e-11 | 46/49 | 15 | 3.407 |
| V$NKX61\_01 (c3) Genes with promoter regions [-2kb,2kb] around transcription start site containing the motif TWTTTAATTGGTT which matches annotation for NKX6-1: NK6 transcription factor related, locus 1 (Drosophila) | 2.61474e-11 | 146/177 | 20 | 5.297 |
| GPCRDB\_CLASS\_A\_RHODOPSIN\_LIKE2 (c2) | 3.67022e-11 | 6/13 | 1 | 0.023 |
| SHEPARD\_CRASH\_AND\_BURN\_MUT\_VS\_WT\_DN (c2) Genes upregulated in zebra fish wild type compared to the crash and burn mutant | 3.95785e-11 | 133/150 | 24 | 7.092 |
| CACGTTT,MIR-302A (c3) Targets of MicroRNA CACGTTT,MIR-302A | 4.56402e-11 | 23/26 | 8 | 1.191 |
| GGATTA\_V$PITX2\_Q2 (c3) Genes with promoter regions [-2kb,2kb] around transcription start site containing the motif GGATTA which matches annotation for PITX2: paired-like homeodomain transcription factor 2 | 5.36596e-11 | 377/456 | 51 | 22.6 |
| HSA04916\_MELANOGENESIS (c2) Genes involved in melanogenesis | 9.99656e-11 | 92/101 | 41 | 17.718 |
| PITX2PATHWAY (c2) The bicoid-related transcription factor Pitx2 is activated by Wnt binding to the Frizzled receptor and induces tissue-specific cell proliferation. | 1.07299e-10 | 14/15 | 20 | 5.932 |
| NEUROGENESIS (c5) Genes annotated by the GO term GO:0022008. Generation of cells within the nervous system. | 1.36336e-10 | 89/93 | 23 | 7.083 |
| WTTGKCTG\_UNKNOWN (c3) Genes with promoter regions [-2kb,2kb] around transcription start site containing motif WTTGKCTG. Motif does not match any known transcription factor | 1.51139e-10 | 324/390 | 49 | 21.038 |
| chr10q25 (c1) Genes in cytogenetic band chr10q25 | 1.51321e-10 | 35/46 | 9 | 1.419 |
| TRANSCRIPTION\_FACTOR\_ACTIVITY (c5) Genes annotated by the GO term GO:0003700. The function of binding to a specific DNA sequence in order to modulate transcription. The transcription factor may or may not also interact selectively with a protein or macromolecular complex. | 1.53862e-10 | 316/353 | 77 | 41.547 |
| V$PBX1\_01 (c3) Genes with promoter regions [-2kb,2kb] around transcription start site containing the motif ANCAATCAW which matches annotation for PBX1: pre-B-cell leukemia transcription factor 1 | 1.64184e-10 | 164/191 | 31 | 11.062 |
| V$PITX2\_Q2 (c3) Genes with promoter regions [-2kb,2kb] around transcription start site containing the motif WNTAATCCCAR which matches annotation for PITX2: paired-like homeodomain transcription factor 2 | 2.04735e-10 | 161/206 | 36 | 14.766 |
| V$HOXA4\_Q2 (c3) Genes with promoter regions [-2kb,2kb] around transcription start site containing the motif AWAATTRG which matches annotation for HOXA4: homeobox A4 | 2.15805e-10 | 173/202 | 32 | 11.944 |
| chr1p11 (c1) Genes in cytogenetic band chr1p11 | 2.16363e-10 | 1/7 | 1 | 0.023 |
| V$DBP\_Q6 (c3) Genes with promoter regions [-2kb,2kb] around transcription start site containing the motif AGCAHAC which matches annotation for DBP: D site of albumin promoter (albumin D-box) binding protein | 2.30643e-10 | 167/192 | 24 | 7.107 |
| CAGGTG\_V$E12\_Q6 (c3) Genes with promoter regions [-2kb,2kb] around transcription start site containing the motif CAGGTG which matches annotation for TCF3: transcription factor 3 (E2A immunoglobulin enhancer binding factors E12/E47) | 2.94071e-10 | 1486/1832 | 148 | 96.293 |
| V$CDC5\_01 (c3) Genes with promoter regions [-2kb,2kb] around transcription start site containing the motif GATTTAACATAA which matches annotation for CDC5L: CDC5 cell division cycle 5-like (S. pombe) | 3.52311e-10 | 174/205 | 33 | 12.196 |
| V$CRX\_Q4 (c3) Genes with promoter regions [-2kb,2kb] around transcription start site containing the motif YNNNTAATCYCMN which matches annotation for CRX: cone-rod homeobox | 4.44424e-10 | 175/210 | 28 | 9.627 |
| GCTNWTTGK\_UNKNOWN (c3) Genes with promoter regions [-2kb,2kb] around transcription start site containing motif GCTNWTTGK. Motif does not match any known transcription factor | 5.36607e-10 | 198/230 | 40 | 16.659 |
| GGGAGGRR\_V$MAZ\_Q6 (c3) Genes with promoter regions [-2kb,2kb] around transcription start site containing the motif GGGAGGRR which matches annotation for MAZ: MYC-associated zinc finger protein (purine-binding transcription factor) | 6.48618e-10 | 1470/1733 | 159 | 104.679 |
| V$FREAC3\_01 (c3) Genes with promoter regions [-2kb,2kb] around transcription start site containing the motif NNNNNGTAAATAAACA which matches annotation for FOXC1: forkhead box C1 | 6.89487e-10 | 157/187 | 29 | 10.382 |
| NEURON\_DIFFERENTIATION (c5) Genes annotated by the GO term GO:0030182. The process whereby a relatively unspecialized cell acquires specialized features of a neuron. | 6.98249e-10 | 72/76 | 20 | 5.925 |
| V$S8\_01 (c3) Genes with promoter regions [-2kb,2kb] around transcription start site containing the motif WNNANYYAATTANCNN which matches annotation for PRRX2: paired related homeobox 2 | 6.99962e-10 | 154/195 | 30 | 11.835 |
| V$AP2\_Q3 (c3) Genes with promoter regions [-2kb,2kb] around transcription start site containing the motif GSCCSCRGGCNRNRNN which matches annotation for GTF3A: general transcription factor IIIA | 8.66154e-10 | 167/195 | 29 | 10.258 |
| V$MEIS1BHOXA9\_01 (c3) Genes with promoter regions [-2kb,2kb] around transcription start site containing the motif TGACAGTTTTAYGR which matches annotation for MEIS1: Meis1, myeloid ecotropic viral integration site 1 homolog (mouse)  HOXA9: homeobox A9 | 9.19627e-10 | 93/110 | 20 | 6.019 |
| GENERATION\_OF\_NEURONS | 1.14728e-09 | 79/83 | 21 | 6.473 |
| NEURITE\_DEVELOPMENT (c5) Genes annotated by the GO term GO:0031175. The process whose specific outcome is the progression of the neurite over time, from its formation to the mature structure. The neurite is any process extending from a neural cell, such as axons or dendrites. | 1.282e-09 | 51/53 | 16 | 4.257 |
| V$HNF4ALPHA\_Q6 (c3) Genes with promoter regions [-2kb,2kb] around transcription start site containing the motif VTGAACTTTGMMB which matches annotation for HNF4A: hepatocyte nuclear factor 4, alpha | 1.34808e-09 | 168/206 | 27 | 9.185 |
| HYPERME\_COLONCA\_SW48 (c2) Gene identified by chromatin IP and CpG island microarray as being hypermethylated in SW48 colon cancer cells, versus normal colon murcosa or WI38 fibroblasts | 1.49126e-09 | 9/14 | 8 | 1.411 |
| MORF\_MYC (c4) Neighborhood of MYC | 1.95285e-09 | 64/74 | 20 | 6.369 |
| chr9q21 (c1) Genes in cytogenetic band chr9q21 | 1.98133e-09 | 22/46 | 7 | 1.051 |
| WNT\_TARGETS (c2) WNT target genes from literatures | 2.07407e-09 | 21/22 | 19 | 5.954 |
| V$HNF1\_C (c3) Genes with promoter regions [-2kb,2kb] around transcription start site containing the motif DGTTAATKAWTNACCAM which matches annotation for TCF1: transcription factor 1, hepatic; LF-B1, hepatic nuclear factor (HNF1), albumin proximal factor | 2.32764e-09 | 146/176 | 24 | 7.825 |
| SHHPATHWAY (c2) Sonic hedgehog (Shh) signaling in the developing CNS induces neuronal proliferation via interaction with the patched (Ptc-1) and smoothened receptors. | 2.73132e-09 | 12/14 | 9 | 1.752 |
| V$GATA1\_03 (c3) Genes with promoter regions [-2kb,2kb] around transcription start site containing the motif ANGNDGATAANNGN which matches annotation for GATA1: GATA binding protein 1 (globin transcription factor 1) | 3.5412e-09 | 171/193 | 31 | 12.038 |
| MORF\_FLT1 (c4) Neighborhood of FLT1 | 3.70583e-09 | 93/118 | 24 | 8.204 |
| V$BRN2\_01 (c3) Genes with promoter regions [-2kb,2kb] around transcription start site containing the motif NNCATNSRWAATNMRN which matches annotation for POU3F2: POU domain, class 3, transcription factor 2 | 4.13247e-09 | 161/190 | 30 | 11.742 |
| V$GR\_Q6 (c3) Genes with promoter regions [-2kb,2kb] around transcription start site containing the motif NNNNNNCNNTNTGTNCTNN which matches annotation for NR3C1: nuclear receptor subfamily 3, group C, member 1 (glucocorticoid receptor) | 4.76863e-09 | 172/207 | 28 | 10.118 |
| V$HNF3\_Q6 (c3) Genes with promoter regions [-2kb,2kb] around transcription start site containing the motif NWRARYAAAYANN which matches annotation for FOXA1: forkhead box A1 | 5.95966e-09 | 128/151 | 23 | 7.546 |
| module\_514 (c4) Genes in module\_514 | 7.68331e-09 | 8/10 | 2 | 0.114 |
| V$NKX25\_02 (c3) Genes with promoter regions [-2kb,2kb] around transcription start site containing the motif CWTAATTG which matches annotation for NKX2-5: NK2 transcription factor related, locus 5 (Drosophila) | 7.71513e-09 | 163/203 | 28 | 10.496 |
| V$ZF5\_B (c3) Genes with promoter regions [-2kb,2kb] around transcription start site containing the motif NRNGNGCGCGCWN which matches annotation for ZFP161: zinc finger protein 161 homolog (mouse) | 8.74763e-09 | 154/188 | 23 | 7.551 |
| TRANSMEMBRANE\_RECEPTOR\_PROTEIN\_PHOSPHATASE\_ACTIVITY (c5) Genes annotated by the GO term GO:0019198. The catalysis of phosphate removal from a phosphotyrosine using aspartic acid as a nucleophile in a metal-dependent manner. | 9.40637e-09 | 18/19 | 6 | 0.854 |
| V$POU6F1\_01 (c3) Genes with promoter regions [-2kb,2kb] around transcription start site containing the motif GCATAAWTTAT which matches annotation for POU6F1: POU domain, class 6, transcription factor 1 | 9.62048e-09 | 158/184 | 23 | 7.76 |
| V$CIZ\_01 (c3) Genes with promoter regions [-2kb,2kb] around transcription start site containing the motif SAAAAANNN which matches annotation for ZNF384: zinc finger protein 384 | 9.67e-09 | 144/174 | 25 | 8.854 |
| V$MZF1\_01 (c3) Genes with promoter regions [-2kb,2kb] around transcription start site containing motif NGNGGGGA. Motif does not match any known transcription factor | 1.28369e-08 | 147/180 | 24 | 8.394 |
| V$NKX25\_01 (c3) Genes with promoter regions [-2kb,2kb] around transcription start site containing the motif TYAAGTG which matches annotation for NKX2-5: NK2 transcription factor related, locus 5 (Drosophila) | 1.55726e-08 | 83/97 | 24 | 8.982 |
| CTTTAAR\_UNKNOWN (c3) Genes with promoter regions [-2kb,2kb] around transcription start site containing motif CTTTAAR. Motif does not match any known transcription factor | 1.83012e-08 | 602/751 | 69 | 38.365 |
| GLUCOSAMINE\_METABOLIC\_PROCESS (c5) Genes annotated by the GO term GO:0006041. The chemical reactions and pathways involving glucosamine (2-amino-2-deoxyglucopyranose), an aminodeoxysugar that occurs in combined form in chitin. | 1.93355e-08 | 7/13 | 1 | 0.032 |
| N\_ACETYLGLUCOSAMINE\_METABOLIC\_PROCESS (c5) Genes annotated by the GO term GO:0006044. The chemical reactions and pathways involving N-acetylglucosamine. The D isomer is a common structural unit of glycoproteins in plants, bacteria and animals; it is often the terminal sugar of an oligosaccharide moiety of a glycoprotein. | 1.93355e-08 | 6/12 | 1 | 0.032 |
| GPCRS\_CLASS\_A\_RHODOPSIN\_LIKE\_2 (c2) GPCR class A rhodopsin genes | 1.93355e-08 | 5/10 | 1 | 0.032 |
| PROTEOGLYCAN\_BIOSYNTHETIC\_PROCESS (c5) Genes annotated by the GO term GO:0030166. The chemical reactions and pathways resulting in the formation of proteoglycans, any glycoprotein in which the carbohydrate units are glycosaminoglycans. | 1.93355e-08 | 5/13 | 1 | 0.032 |
| CAGGTA\_V$AREB6\_01 (c3) Genes with promoter regions [-2kb,2kb] around transcription start site containing the motif CAGGTA which matches annotation for TCF8: transcription factor 8 (represses interleukin 2 expression) | 3.12967e-08 | 471/582 | 61 | 33.361 |
| module\_379 (c4) Genes in module\_379 | 3.22963e-08 | 158/198 | 21 | 6.913 |
| WGTTNNNNNAAA\_UNKNOWN (c3) Genes with promoter regions [-2kb,2kb] around transcription start site containing motif WGTTNNNNNAAA. Motif does not match any known transcription factor | 3.92424e-08 | 341/423 | 47 | 23.268 |
| NUCLEOTIDE\_SUGAR\_METABOLIC\_PROCESS (c5) Genes annotated by the GO term GO:0009225. The chemical reactions and pathways involving nucleotide-sugars, any nucleotide in which the distal phosphoric residue of a nucleoside 5'-diphosphate is in glycosidic linkage with a monosaccharide or monosaccharide derivative. | 4.9865e-08 | 5/9 | 1 | 0.034 |
| DNA\_BINDING (c5) Genes annotated by the GO term GO:0003677. Interacting selectively with DNA (deoxyribonucleic acid). | 5.32055e-08 | 540/600 | 91 | 56.2 |
| V$POU3F2\_02 (c3) Genes with promoter regions [-2kb,2kb] around transcription start site containing the motif TTATGYTAAT which matches annotation for POU3F2: POU domain, class 3, transcription factor 2 | 6.75265e-08 | 153/197 | 28 | 11.676 |
| V$GATA\_C (c3) Genes with promoter regions [-2kb,2kb] around transcription start site containing the motif NGATAAGNMNN which matches annotation for GATA1: GATA binding protein 1 (globin transcription factor 1) | 7.0327e-08 | 174/204 | 30 | 12.801 |
| V$FOXD3\_01 (c3) Genes with promoter regions [-2kb,2kb] around transcription start site containing the motif NAWTGTTTRTTT which matches annotation for FOXD3: forkhead box D3 | 7.08044e-08 | 142/163 | 26 | 9.973 |
| V$SMAD4\_Q6 (c3) Genes with promoter regions [-2kb,2kb] around transcription start site containing the motif GKSRKKCAGMCANCY which matches annotation for SMAD4: SMAD, mothers against DPP homolog 4 (Drosophila) | 7.66382e-08 | 153/180 | 26 | 10.164 |
| V$CART1\_01 (c3) Genes with promoter regions [-2kb,2kb] around transcription start site containing the motif NNNTAATTNNCATTANCN which matches annotation for CART1: cartilage paired-class homeoprotein 1 | 7.70744e-08 | 137/168 | 21 | 7.354 |
| ACETYLGALACTOSAMINYLTRANSFERASE\_ACTIVITY (c5) Genes annotated by the GO term GO:0008376. Catalysis of the transfer of an N-acetylgalactosaminyl residue from UDP-N-acetyl-galactosamine to an oligosaccharide. | 1.15931e-07 | 8/11 | 1 | 0.036 |
| NEURON\_DEVELOPMENT (c5) Genes annotated by the GO term GO:0048666. The process whose specific outcome is the progression of a neuron over time, from initial commitment of the cell to a specific fate, to the fully functional differentiated cell. | 1.31137e-07 | 59/61 | 16 | 4.959 |
| HDACI\_COLON\_SUL2HRS\_DN (c2) Downregulated by sulindac at 2 hrs in SW260 colon carcinoma cells | 1.35724e-07 | 12/14 | 6 | 0.926 |
| V$TFIII\_Q6 (c3) Genes with promoter regions [-2kb,2kb] around transcription start site containing the motif RGAGGKAGG which matches annotation for GTF2A1: general transcription factor IIA, 1, 19/37kDa  GTF2A2: general transcription factor IIA, 2, 12kDa | 1.36583e-07 | 146/165 | 26 | 9.905 |
| V$HFH4\_01 | 1.51553e-07 | 135/160 | 21 | 7.485 |
| HSA01030\_GLYCAN\_STRUCTURES\_BIOSYNTHESIS\_1 (c2) Genes involved in glycan structures - biosynthesis 1 | 1.6687e-07 | 56/109 | 4 | 0.507 |
| V$WHN\_B (c3) Genes with promoter regions [-2kb,2kb] around transcription start site containing the motif ANNGACGCTNN which matches annotation for FOXN1: forkhead box N1 | 1.86343e-07 | 167/191 | 27 | 11.191 |
| HDACI\_COLON\_TSABUT\_DN (c2) Downregulated by both butyrate and TSA at any timepoint up to 48 hrs in SW260 colon carcinoma cells | 1.92876e-07 | 14/19 | 9 | 2.055 |
| YTAAYNGCT\_UNKNOWN (c3) Genes with promoter regions [-2kb,2kb] around transcription start site containing motif YTAAYNGCT. Motif does not match any known transcription factor | 2.40946e-07 | 98/115 | 18 | 6.368 |
| TTANTCA\_UNKNOWN (c3) Genes with promoter regions [-2kb,2kb] around transcription start site containing motif TTANTCA. Motif does not match any known transcription factor | 2.53793e-07 | 567/706 | 68 | 38.945 |
| V$T3R\_Q6 (c3) Genes with promoter regions [-2kb,2kb] around transcription start site containing motif MNTGWCCTN. Motif does not match any known transcription factor | 2.76369e-07 | 160/194 | 21 | 7.532 |
| V$FREAC4\_01 (c3) Genes with promoter regions [-2kb,2kb] around transcription start site containing the motif CTWAWGTAAACANWGN which matches annotation for FOXD1: forkhead box D1 | 2.93152e-07 | 88/113 | 16 | 5.096 |
| YKACATTT\_UNKNOWN (c3) Genes with promoter regions [-2kb,2kb] around transcription start site containing motif YKACATTT. Motif does not match any known transcription factor | 2.98349e-07 | 157/216 | 29 | 12.404 |
| V$PAX\_Q6 (c3) Genes with promoter regions [-2kb,2kb] around transcription start site containing the motif CTGGAACTMAC which matches annotation for PAX2: paired box gene 2 | 2.98651e-07 | 166/202 | 25 | 9.893 |
| V$GATA3\_01 (c3) Genes with promoter regions [-2kb,2kb] around transcription start site containing the motif NNGATARNG which matches annotation for GATA3: GATA binding protein 3 | 3.24288e-07 | 159/185 | 31 | 13.578 |
| TSA\_CD4\_DN (c2) Down-regulated in mouse CD4+ T-cells following 4 hour treatment with 100 nM trichostatin A | 3.30768e-07 | 17/18 | 8 | 1.631 |
| ZHAN\_MM\_CD138\_MS\_VS\_REST (c2) 50 top ranked SAM-defined over-expressed genes in each subgroup\_MS | 3.49216e-07 | 30/43 | 8 | 1.627 |
| CCAWNWWNNNGGC\_UNKNOWN (c3) Genes with promoter regions [-2kb,2kb] around transcription start site containing motif CCAWNWWNNNGGC. Motif does not match any known transcription factor | 3.63592e-07 | 45/58 | 9 | 2.094 |
| V$FAC1\_01 (c3) Genes with promoter regions [-2kb,2kb] around transcription start site containing the motif NNNCAMAACACRNA which matches annotation for FALZ: fetal Alzheimer antigen | 3.73555e-07 | 143/158 | 24 | 9.531 |
| V$LYF1\_01 (c3) Genes with promoter regions [-2kb,2kb] around transcription start site containing motif TTTGGGAGR. Motif does not match any known transcription factor | 3.76952e-07 | 173/201 | 22 | 8.573 |
| V$AREB6\_01 (c3) Genes with promoter regions [-2kb,2kb] around transcription start site containing the motif NNYNYACCTGWVT which matches annotation for TCF8: transcription factor 8 (represses interleukin 2 expression) | 4.20967e-07 | 165/204 | 28 | 12.715 |
| ATGTTAA,MIR-302C (c3) Targets of MicroRNA ATGTTAA,MIR-302C | 4.66681e-07 | 171/219 | 24 | 9.519 |
| chr14q22 (c1) Genes in cytogenetic band chr14q22 | 4.71619e-07 | 29/60 | 6 | 1.103 |
| V$SP1\_Q2\_01 (c3) Genes with promoter regions [-2kb,2kb] around transcription start site containing the motif CCCCGCCCCN which matches annotation for SP1: Sp1 transcription factor | 5.05999e-07 | 171/191 | 28 | 11.582 |
| V$PAX3\_01 (c3) Genes with promoter regions [-2kb,2kb] around transcription start site containing the motif TCGTCACRCTTHM which matches annotation for PAX3: paired box gene 3 (Waardenburg syndrome 1) | 5.67008e-07 | 12/15 | 4 | 0.533 |
| V$ALPHACP1\_01 (c3) Genes with promoter regions [-2kb,2kb] around transcription start site containing the motif CAGCCAATGAG which matches annotation for PCBP1: poly(rC) binding protein 1 | 5.84057e-07 | 165/190 | 26 | 10.76 |
| CATTGTYY\_V$SOX9\_B1 (c3) Genes with promoter regions [-2kb,2kb] around transcription start site containing the motif CATTGTYY which matches annotation for SOX9: SRY (sex determining region Y)-box 9 (campomelic dysplasia, autosomal sex-reversal) | 6.04143e-07 | 239/279 | 39 | 19.012 |
| GLAND\_DEVELOPMENT (c5) Genes annotated by the GO term GO:0048732. The process whose specific outcome is the progression of a gland over time, from its formation to the mature structure. A gland is an organ specialised for secretion. | 6.09389e-07 | 12/13 | 9 | 2.203 |
| MORF\_LTK (c4) Neighborhood of LTK | 6.12143e-07 | 110/135 | 18 | 6.065 |
| chr4q23 (c1) Genes in cytogenetic band chr4q23 | 6.15271e-07 | 11/19 | 4 | 0.549 |
| CMV\_HCMV\_TIMECOURSE\_10HRS\_DN (c2) Down-regulated in fibroblasts following infection with human cytomegalovirus (at least 3-fold, with Affymetrix change call, in at least two consectutive timepoints), with maximum change at 10 hours | 6.74423e-07 | 10/14 | 3 | 0.316 |
| TGACAGNY\_V$MEIS1\_01 (c3) Genes with promoter regions [-2kb,2kb] around transcription start site containing the motif TGACAGNY which matches annotation for MEIS1: Meis1, myeloid ecotropic viral integration site 1 homolog (mouse) | 6.82179e-07 | 521/618 | 56 | 29.996 |
| module\_242 (c4) Genes in module\_242 | 6.90884e-07 | 166/208 | 22 | 8.189 |
| GATTGGY\_V$NFY\_Q6\_01 (c3) Genes with promoter regions [-2kb,2kb] around transcription start site containing motif GATTGGY. Motif does not match any known transcription factor | 7.48211e-07 | 703/856 | 80 | 49.014 |
| HTERT\_UP (c2) Upregulated in hTERT-immortalized fibroblasts vs. non-immortalized controls | 7.5214e-07 | 53/65 | 11 | 2.992 |
| GGGCGGR\_V$SP1\_Q6 (c3) Genes with promoter regions [-2kb,2kb] around transcription start site containing the motif GGGCGGR which matches annotation for SP1: Sp1 transcription factor | 9.47979e-07 | 1819/2167 | 159 | 118.408 |
| YATGNWAAT\_V$OCT\_C (c3) Genes with promoter regions [-2kb,2kb] around transcription start site containing motif YATGNWAAT. Motif does not match any known transcription factor | 9.69356e-07 | 229/289 | 34 | 15.803 |
| V$NKX3A\_01 (c3) Genes with promoter regions [-2kb,2kb] around transcription start site containing the motif NWATAAGTATWT which matches annotation for NKX3-1: NK3 transcription factor related, locus 1 (Drosophila) | 1.03702e-06 | 131/170 | 22 | 8.63 |
| V$ER\_Q6\_01 (c3) Genes with promoter regions [-2kb,2kb] around transcription start site containing the motif KDMAYYNTGACCT which matches annotation for AR: androgen receptor (dihydrotestosterone receptor; testicular feminization; spinal and bulbar muscular atrophy; Kennedy disease) | 1.03982e-06 | 173/201 | 23 | 9.052 |
| V$IRF1\_01 (c3) Genes with promoter regions [-2kb,2kb] around transcription start site containing the motif SAAAAGYGAAACC which matches annotation for IRF1: interferon regulatory factor 1 | 1.03991e-06 | 162/196 | 33 | 15.155 |
| V$EVI1\_02 (c3) Genes with promoter regions [-2kb,2kb] around transcription start site containing the motif AGAYAAGATAA which matches annotation for EVI1: ecotropic viral integration site 1 | 1.05949e-06 | 74/96 | 15 | 4.963 |
| ACAWYAAAG\_UNKNOWN (c3) Genes with promoter regions [-2kb,2kb] around transcription start site containing motif ACAWYAAAG. Motif does not match any known transcription factor | 1.11888e-06 | 61/84 | 11 | 3.174 |
| V$SP3\_Q3 (c3) Genes with promoter regions [-2kb,2kb] around transcription start site containing the motif ASMCTTGGGSRGGG which matches annotation for SP3: Sp3 transcription factor | 1.19296e-06 | 154/187 | 26 | 11.149 |
| V$CACCCBINDINGFACTOR\_Q6 (c3) Genes with promoter regions [-2kb,2kb] around transcription start site containing motif CANCCNNWGGGTGDGG. Motif does not match any known transcription factor | 1.3425e-06 | 181/211 | 27 | 12.326 |
| V$SRY\_02 (c3) Genes with promoter regions [-2kb,2kb] around transcription start site containing the motif NWWAACAAWANN which matches annotation for SRY: sex determining region Y | 1.38147e-06 | 160/188 | 28 | 12.943 |
| HOX\_GENES (c2) HOX genes related to hematopoiesis | 1.48942e-06 | 48/53 | 9 | 2.253 |
| V$CEBPA\_01 (c3) Genes with promoter regions [-2kb,2kb] around transcription start site containing the motif NNATTRCNNAANNN which matches annotation for CEBPA: CCAAT/enhancer binding protein (C/EBP), alpha | 1.51271e-06 | 161/190 | 27 | 11.849 |
| GSK3PATHWAY (c2) Bacterial lipopolysaccharide activates AKT to promote the survival and activation of macrophages and inhibits Gsk3-beta to promote beta-catenin accumulation in the nucleus. | 1.53833e-06 | 25/26 | 25 | 11.415 |
| HEMATOPOESIS\_RELATED\_TRANSCRIPTION\_FACTORS (c2) Transcription factors involved in hematopoiesis | 1.55746e-06 | 82/83 | 41 | 21.667 |
| chr9q12 (c1) Genes in cytogenetic band chr9q12 | 1.58933e-06 | 4/21 | 1 | 0.042 |
| V$LMO2COM\_01 (c3) Genes with promoter regions [-2kb,2kb] around transcription start site containing the motif CNNCAGGTGBNN which matches annotation for LMO2: LIM domain only 2 (rhombotin-like 1) | 1.61394e-06 | 159/195 | 25 | 10.322 |
| chr2q37 (c1) Genes in cytogenetic band chr2q37 | 1.62277e-06 | 60/114 | 6 | 1.104 |
| CTTTGTA,MIR-524 (c3) Targets of MicroRNA CTTTGTA,MIR-524 | 1.77393e-06 | 319/391 | 45 | 24.473 |
| GH\_EXOGENOUS\_ANY\_DN (c2) Down-regulated at any time point (1-24 hours) following treatment of mammary carcinoma cells (MCF-7) with exogenous human growth hormone | 1.79158e-06 | 63/83 | 10 | 2.638 |
| V$PR\_Q2 (c3) Genes with promoter regions [-2kb,2kb] around transcription start site containing the motif NWNAGRACAN which matches annotation for NR3C1: nuclear receptor subfamily 3, group C, member 1 (glucocorticoid receptor) | 1.92873e-06 | 167/194 | 21 | 8.213 |
| V$CDX2\_Q5 (c3) Genes with promoter regions [-2kb,2kb] around transcription start site containing the motif ANANTTTTATKRCC which matches annotation for CDX2: caudal type homeobox transcription factor 2 | 2.04018e-06 | 164/195 | 23 | 9.328 |
| CGGTGTG,MIR-220 (c3) Targets of MicroRNA CGGTGTG,MIR-220 | 2.26129e-06 | 4/6 | 3 | 0.336 |
| V$AREB6\_04 (c3) Genes with promoter regions [-2kb,2kb] around transcription start site containing the motif CBGTTTSNN which matches annotation for TCF8: transcription factor 8 (represses interleukin 2 expression) | 2.35245e-06 | 163/193 | 31 | 14.655 |
| GATAAGR\_V$GATA\_C (c3) Genes with promoter regions [-2kb,2kb] around transcription start site containing motif GATAAGR. Motif does not match any known transcription factor | 2.37673e-06 | 195/237 | 30 | 13.999 |
| V$FREAC7\_01 (c3) Genes with promoter regions [-2kb,2kb] around transcription start site containing the motif WNNANATAAAYANNNN which matches annotation for FOXL1: forkhead box L1 | 2.44423e-06 | 122/148 | 20 | 7.819 |
| CMV\_HCMV\_TIMECOURSE\_10HRS\_UP (c2) Up-regulated in fibroblasts following infection with human cytomegalovirus (at least 3-fold, with Affymetrix change call, in at least two consectutive timepoints), with maximum change at 10 hours | 2.64621e-06 | 6/8 | 3 | 0.341 |
| V$NFY\_Q6 (c3) Genes with promoter regions [-2kb,2kb] around transcription start site containing motif TRRCCAATSRN. Motif does not match any known transcription factor | 2.75831e-06 | 158/197 | 27 | 11.661 |
| V$FOXO3\_01 (c3) Genes with promoter regions [-2kb,2kb] around transcription start site containing the motif TNNTTGTTTACNTW which matches annotation for FOXO3A: forkhead box O3A | 3.0788e-06 | 150/178 | 23 | 9.722 |
| V$FREAC2\_01 (c3) Genes with promoter regions [-2kb,2kb] around transcription start site containing the motif NNANNGTAAACAANNN which matches annotation for FOXF2: forkhead box F2 | 3.17042e-06 | 172/196 | 25 | 10.924 |
| IRS\_KO\_ADIP\_DN (c2) Progressively down-regulated in brown preadipocytes from four Irs-knockout mouse lines with increasingly severe defects in adipocyte differentiation (wt, Irs4 KO, Irs2 KO, Irs3 KO, Irs1 KO) | 3.29395e-06 | 34/37 | 6 | 1.23 |
| V$HEN1\_01 (c3) Genes with promoter regions [-2kb,2kb] around transcription start site containing the motif NNNGGNCNCAGCTGCGNCCCNN which matches annotation for NHLH1: nescient helix loop helix 1 | 3.31936e-06 | 123/155 | 23 | 10.152 |
| V$GATA4\_Q3 (c3) Genes with promoter regions [-2kb,2kb] around transcription start site containing the motif AGATADMAGGGA which matches annotation for GATA4: GATA binding protein 4 | 3.45257e-06 | 158/185 | 27 | 12.125 |
| V$IRF1\_Q6 (c3) Genes with promoter regions [-2kb,2kb] around transcription start site containing the motif TTCACTT which matches annotation for IRF1: interferon regulatory factor 1 | 3.63327e-06 | 175/203 | 23 | 9.654 |
| V$NFY\_C (c3) Genes with promoter regions [-2kb,2kb] around transcription start site containing motif NCTGATTGGYTASY. Motif does not match any known transcription factor | 3.76314e-06 | 156/187 | 25 | 11.305 |
| MORF\_RAGE (c4) Neighborhood of RAGE | 3.81485e-06 | 113/134 | 17 | 6.083 |
| HSA00641\_3\_CHLOROACRYLIC\_ACID\_DEGRADATION (c2) Genes involved in 3-chloroacrylic acid degradation | 3.84471e-06 | 9/15 | 2 | 0.163 |
| V$TTF1\_Q6 (c3) Genes with promoter regions [-2kb,2kb] around transcription start site containing the motif NNNNCAAGNRNN which matches annotation for TITF1: thyroid transcription factor 1 | 3.94731e-06 | 160/198 | 26 | 11.866 |
| CRGAARNNNNCGA\_UNKNOWN (c3) Genes with promoter regions [-2kb,2kb] around transcription start site containing motif CRGAARNNNNCGA. Motif does not match any known transcription factor | 4.1427e-06 | 31/35 | 11 | 3.423 |
| TATTATA,MIR-374 (c3) Targets of MicroRNA TATTATA,MIR-374 | 4.19759e-06 | 199/247 | 32 | 15.647 |
| V$CMYB\_01 (c3) Genes with promoter regions [-2kb,2kb] around transcription start site containing the motif NCNRNNGRCNGTTGGKGG which matches annotation for MYB: v-myb myeloblastosis viral oncogene homolog (avian) | 4.31712e-06 | 155/181 | 24 | 10.349 |
| AACYNNNNTTCCS\_UNKNOWN (c3) Genes with promoter regions [-2kb,2kb] around transcription start site containing motif AACYNNNNTTCCS. Motif does not match any known transcription factor | 4.31751e-06 | 51/64 | 13 | 4.308 |
| NEGATIVE\_REGULATION\_OF\_CELL\_DIFFERENTIATION (c5) Genes annotated by the GO term GO:0045596. Any process that stops, prevents or reduces the frequency, rate or extent of cell differentiation. | 4.37565e-06 | 26/28 | 13 | 4.304 |
| V$AP2\_Q6 (c3) Genes with promoter regions [-2kb,2kb] around transcription start site containing the motif MKCCCSCNGGCG which matches annotation for GTF3A: general transcription factor IIIA | 4.38707e-06 | 162/192 | 24 | 10.562 |
| AGCYRWTTC\_UNKNOWN (c3) Genes with promoter regions [-2kb,2kb] around transcription start site containing motif AGCYRWTTC. Motif does not match any known transcription factor | 4.57003e-06 | 73/84 | 16 | 5.592 |
| V$SMAD3\_Q6 (c3) Genes with promoter regions [-2kb,2kb] around transcription start site containing the motif TGTCTGTCT which matches annotation for SMAD3: SMAD, mothers against DPP homolog 3 (Drosophila) | 4.7884e-06 | 154/182 | 21 | 8.597 |
| TGATTTRY\_V$GFI1\_01 (c3) Genes with promoter regions [-2kb,2kb] around transcription start site containing the motif TGATTTRY which matches annotation for GFI1: growth factor independent 1 | 4.83261e-06 | 199/235 | 35 | 17.882 |
| V$EFC\_Q6 (c3) Genes with promoter regions [-2kb,2kb] around transcription start site containing the motif MGTTACYAGGCAAM which matches annotation for RFX1: regulatory factor X, 1 (influences HLA class II expression) | 5.37947e-06 | 171/210 | 24 | 10.42 |
| WGGAATGY\_V$TEF1\_Q6 (c3) Genes with promoter regions [-2kb,2kb] around transcription start site containing the motif WGGAATGY which matches annotation for TEAD1: TEA domain family member 1 (SV40 transcriptional enhancer factor) | 5.94537e-06 | 245/284 | 36 | 18.033 |
| GCCNNNWTAAR\_UNKNOWN (c3) Genes with promoter regions [-2kb,2kb] around transcription start site containing motif GCCNNNWTAAR. Motif does not match any known transcription factor | 6.52114e-06 | 100/117 | 21 | 8.584 |
| V$GATA1\_02 (c3) Genes with promoter regions [-2kb,2kb] around transcription start site containing the motif NNNNNGATANKGNN which matches annotation for GATA1: GATA binding protein 1 (globin transcription factor 1) | 6.52912e-06 | 158/186 | 27 | 12.899 |
| CACGTG\_V$MYC\_Q2 (c3) Genes with promoter regions [-2kb,2kb] around transcription start site containing the motif CACGTG which matches annotation for MYC: v-myc myelocytomatosis viral oncogene homolog (avian) | 6.95184e-06 | 615/756 | 59 | 34.664 |
| HDACI\_COLON\_SUL16HRS\_DN (c2) Downregulated by sulindac at 16 hrs in SW260 colon carcinoma cells | 7.48558e-06 | 54/71 | 10 | 2.807 |
| V$MSX1\_01 (c3) Genes with promoter regions [-2kb,2kb] around transcription start site containing the motif CNGTAWNTG which matches annotation for MSX1: msh homeobox homolog 1 (Drosophila) | 7.56647e-06 | 119/144 | 23 | 10.328 |
| V$FOXO1\_02 (c3) Genes with promoter regions [-2kb,2kb] around transcription start site containing the motif GNNTTGTTTACNTT which matches annotation for FOXO1A: forkhead box O1A (rhabdomyosarcoma) | 7.63103e-06 | 155/179 | 23 | 9.78 |
| V$GATA\_Q6 (c3) Genes with promoter regions [-2kb,2kb] around transcription start site containing the motif WGATARN which matches annotation for GATA1: GATA binding protein 1 (globin transcription factor 1) | 7.68293e-06 | 134/161 | 20 | 8.332 |
| V$MAZR\_01 (c3) Genes with promoter regions [-2kb,2kb] around transcription start site containing motif NSGGGGGGGGMCN. Motif does not match any known transcription factor | 9.71849e-06 | 149/171 | 22 | 9.32 |
| V$NFY\_01 (c3) Genes with promoter regions [-2kb,2kb] around transcription start site containing motif NNNRRCCAATSRGNNN. Motif does not match any known transcription factor | 1.06401e-05 | 152/186 | 24 | 10.766 |
| V$GFI1\_01 (c3) Genes with promoter regions [-2kb,2kb] around transcription start site containing the motif NNNNNNNAAATCACWGYNNNNNNN which matches annotation for GFI1: growth factor independent 1 | 1.07232e-05 | 177/204 | 25 | 11.153 |
| V$COUP\_01 (c3) Genes with promoter regions [-2kb,2kb] around transcription start site containing the motif TGAMCTTTGMMCYT which matches annotation for HNF4A: hepatocyte nuclear factor 4, alpha | 1.07399e-05 | 155/195 | 23 | 10.477 |
| V$HFH3\_01 (c3) Genes with promoter regions [-2kb,2kb] around transcription start site containing the motif KNNTRTTTRTTTA which matches annotation for FOXI1: forkhead box I1 | 1.0906e-05 | 128/156 | 23 | 10.494 |
| V$FOXO1\_01 (c3) Genes with promoter regions [-2kb,2kb] around transcription start site containing the motif NRWAAACAAN which matches annotation for FOXO1A: forkhead box O1A (rhabdomyosarcoma) | 1.14501e-05 | 158/190 | 21 | 9.006 |
| V$MMEF2\_Q6 (c3) Genes with promoter regions [-2kb,2kb] around transcription start site containing motif CKSNYTAAAAAWRMCY. Motif does not match any known transcription factor | 1.17329e-05 | 173/216 | 24 | 10.452 |
| V$PAX4\_04 (c3) Genes with promoter regions [-2kb,2kb] around transcription start site containing the motif RAAAAWTANNNNNNNNNNNNNNNYCACNCC which matches annotation for PAX4: paired box gene 4 | 1.19407e-05 | 143/163 | 20 | 8.101 |
| CELL\_RECOGNITION (c5) Genes annotated by the GO term GO:0008037. The process by which a cell in a multicellular organism interprets its surroundings. | 1.24213e-05 | 15/18 | 3 | 0.408 |
| V$HLF\_01 (c3) Genes with promoter regions [-2kb,2kb] around transcription start site containing the motif GTTACRYAAT which matches annotation for HLF: hepatic leukemia factor | 1.28617e-05 | 165/199 | 31 | 15.77 |
| MUSCLE\_CELL\_DIFFERENTIATION (c5) Genes annotated by the GO term GO:0042692. The process whereby a relatively unspecialized cell acquires specialized features of a muscle cell. | 1.2917e-05 | 21/22 | 8 | 2.019 |
| TGTTTGY\_V$HNF3\_Q6 (c3) Genes with promoter regions [-2kb,2kb] around transcription start site containing the motif TGTTTGY which matches annotation for FOXA1: forkhead box A1 | 1.38544e-05 | 473/562 | 54 | 31.693 |
| V$FOX\_Q2 (c3) Genes with promoter regions [-2kb,2kb] around transcription start site containing the motif KATTGTTTRTTTW which matches annotation for FOXF2: forkhead box F2 | 1.42981e-05 | 135/163 | 18 | 6.97 |
| SULFUR\_COMPOUND\_BIOSYNTHETIC\_PROCESS (c5) Genes annotated by the GO term GO:0044272. The chemical reactions and pathways resulting in the formation of compounds that contain sulfur, such as the amino acids methionine and cysteine or the tripeptide glutathione. | 1.43666e-05 | 7/16 | 1 | 0.054 |
| MORF\_FDXR (c4) Neighborhood of FDXR | 1.4593e-05 | 175/208 | 25 | 11.361 |
| V$FOXO4\_01 (c3) Genes with promoter regions [-2kb,2kb] around transcription start site containing the motif RWAAACAANNN which matches annotation for MLLT7: myeloid/lymphoid or mixed-lineage leukemia (trithorax homolog, Drosophila); translocated to, 7 | 1.54824e-05 | 155/187 | 21 | 8.839 |
| V$SRY\_01 (c3) Genes with promoter regions [-2kb,2kb] around transcription start site containing the motif AAACWAM which matches annotation for SRY: sex determining region Y | 1.57203e-05 | 125/149 | 18 | 6.928 |
| CCCNNGGGAR\_V$OLF1\_01 (c3) Genes with promoter regions [-2kb,2kb] around transcription start site containing the motif CCCNNGGGAR which matches annotation for EBF2: early B-cell factor 2 | 1.58511e-05 | 193/242 | 23 | 10.431 |
| YNGTTNNNATT\_UNKNOWN (c3) Genes with promoter regions [-2kb,2kb] around transcription start site containing motif YNGTTNNNATT. Motif does not match any known transcription factor | 1.76572e-05 | 234/275 | 31 | 15.426 |
| V$LMO2COM\_02 (c3) Genes with promoter regions [-2kb,2kb] around transcription start site containing the motif NMGATANSG which matches annotation for LMO2: LIM domain only 2 (rhombotin-like 1) | 1.81675e-05 | 169/193 | 21 | 9.196 |
| CELL\_MIGRATION (c5) Genes annotated by the GO term GO:0016477. The orderly movement of cells from one site to another, often during the development of a multicellular organism. | 1.90713e-05 | 92/96 | 23 | 10.364 |
| PROTEIN\_TYROSINE\_PHOSPHATASE\_ACTIVITY (c5) Genes annotated by the GO term GO:0004725. Catalysis of the reaction: protein tyrosine phosphate + H2O = protein tyrosine + phosphate. | 1.96788e-05 | 50/53 | 11 | 3.47 |
| V$HMGIY\_Q6 (c3) Genes with promoter regions [-2kb,2kb] around transcription start site containing the motif GGAAAWT which matches annotation for HMGA1: high mobility group AT-hook 1 | 1.97005e-05 | 156/183 | 21 | 8.897 |
| TCTGGAC,MIR-198 (c3) Targets of MicroRNA TCTGGAC,MIR-198 | 1.99777e-05 | 51/70 | 11 | 3.553 |
| AAAYRNCTG\_UNKNOWN (c3) Genes with promoter regions [-2kb,2kb] around transcription start site containing motif AAAYRNCTG. Motif does not match any known transcription factor | 2.05243e-05 | 242/298 | 28 | 13.566 |
| LEE\_TCELLS9\_UP (c2) Transcripts showing SP4>CB4>AB4 pattern | 2.29134e-05 | 20/25 | 5 | 0.996 |
| ORGAN\_MORPHOGENESIS (c5) Genes annotated by the GO term GO:0009887. Morphogenesis of an organ. An organ is defined as a tissue or set of tissues that work together to perform a specific function or functions. Morphogenesis is the process by which anatomical structures are generated and organized. Organs are commonly observed as visibly distinct structures, but may also exist as loosely associated clusters of cells that work together to perform a specific function or functions. | 2.29174e-05 | 136/145 | 35 | 18.902 |
| SMTTTTGT\_UNKNOWN (c3) Genes with promoter regions [-2kb,2kb] around transcription start site containing motif SMTTTTGT. Motif does not match any known transcription factor | 2.40346e-05 | 279/321 | 36 | 19.382 |
| V$HFH8\_01 (c3) Genes with promoter regions [-2kb,2kb] around transcription start site containing the motif NNNTGTTTATNTR which matches annotation for FOXJ1: forkhead box J1 | 2.48972e-05 | 141/165 | 25 | 12.094 |
| ATGCTGC,MIR-103,MIR-107 (c3) Targets of MicroRNA ATGCTGC,MIR-103,MIR-107 | 2.50328e-05 | 147/198 | 22 | 9.62 |
| BECKER\_ESTROGEN\_RESPONSIVE\_SUBSET\_2 (c2) Estrogen-responsive genes which are downregulated in MaCa 3366/TAM compared to MaCa 3366 (fold change > 2) | 2.68164e-05 | 6/7 | 7 | 1.884 |
| RNGTGGGC\_UNKNOWN (c3) Genes with promoter regions [-2kb,2kb] around transcription start site containing motif RNGTGGGC. Motif does not match any known transcription factor | 2.68717e-05 | 476/564 | 52 | 31.283 |
| HSA04520\_ADHERENS\_JUNCTION (c2) Genes involved in adherens junction | 2.72233e-05 | 74/75 | 41 | 23.177 |
| chr10q26 (c1) Genes in cytogenetic band chr10q26 | 2.76243e-05 | 43/83 | 4 | 0.673 |
| NEGATIVE\_REGULATION\_OF\_CELL\_MIGRATION (c5) Genes annotated by the GO term GO:0030336. Any process that stops, prevents or reduces the frequency, rate or extent of cell migration. | 3.12885e-05 | 12/15 | 8 | 2.275 |
| GGGCATT,MIR-365 (c3) Targets of MicroRNA GGGCATT,MIR-365 | 3.17114e-05 | 77/102 | 12 | 4.22 |
| V$GATA1\_05 (c3) Genes with promoter regions [-2kb,2kb] around transcription start site containing the motif NCWGATAACA which matches annotation for GATA1: GATA binding protein 1 (globin transcription factor 1) | 3.31747e-05 | 178/214 | 33 | 18.06 |
| V$AR\_Q2 (c3) Genes with promoter regions [-2kb,2kb] around transcription start site containing the motif AGWACATNWTGTTCT which matches annotation for AR: androgen receptor (dihydrotestosterone receptor; testicular feminization; spinal and bulbar muscular atrophy; Kennedy disease) | 3.46322e-05 | 85/98 | 15 | 5.658 |
| V$STAT5A\_04 (c3) Genes with promoter regions [-2kb,2kb] around transcription start site containing the motif NNNTTCYN which matches annotation for STAT5A: signal transducer and activator of transcription 5A | 4.01762e-05 | 127/143 | 22 | 10.033 |
| WTGAAAT\_UNKNOWN (c3) Genes with promoter regions [-2kb,2kb] around transcription start site containing motif WTGAAAT. Motif does not match any known transcription factor | 4.03088e-05 | 358/459 | 44 | 25.38 |
| V$PPARG\_01 (c3) Genes with promoter regions [-2kb,2kb] around transcription start site containing the motif NNWGRGGTCAAAGGTCANNNN which matches annotation for PPARG: peroxisome proliferative activated receptor, gamma | 4.23853e-05 | 27/33 | 4 | 0.713 |
| V$PAX4\_03 (c3) Genes with promoter regions [-2kb,2kb] around transcription start site containing the motif NNNNNYCACCCB which matches annotation for PAX4: paired box gene 4 | 4.63726e-05 | 169/200 | 24 | 11.628 |
| module\_123 (c4) Genes in module\_123 | 4.63969e-05 | 222/247 | 53 | 32.41 |
| TISSUE\_DEVELOPMENT (c5) Genes annotated by the GO term GO:0009888. The process whose specific outcome is the progression of a tissue over time, from its formation to the mature structure. | 4.74053e-05 | 131/137 | 21 | 9.469 |
