# Supplementary material for: Looking at Cerebellar Malformations through Text-Mined Interactomes of Mice and Humans
Source: PLoS Comput Biol. 2009 Nov 6;5(11):e1000559. doi: 10.1371/journal.pcbi.1000559 (PMC2767227; doi:10.1371/journal.pcbi.1000559)
Supplement: Dataset S1 — All enrichment results. (0.20 MB ZIP) [file pcbi.1000559.s012.zip › enrichment_results/Table T. enrichment_whole-all.html]

Complete Clustering results for network whole and phenotype all (FDR <= 0.001)


# Complete Clustering results for network whole and phenotype all (FDR <= 0.001)

| Set | p-Value | Gene Count | Interaction Count | Expected Interection Count |
| --- | --- | --- | --- | --- |
| NERVOUS\_SYSTEM\_DEVELOPMENT (c5) Genes annotated by the GO term GO:0007399. The process whose specific outcome is the progression of nervous tissue over time, from its formation to its mature state. | 1e-20 | 331/382 | 663 | 458.077 |
| HSA04340\_HEDGEHOG\_SIGNALING\_PATHWAY (c2) Genes involved in Hedgehog signaling pathway | 1e-20 | 53/57 | 230 | 117.465 |
| HSA05217\_BASAL\_CELL\_CARCINOMA (c2) Genes involved in basal cell carcinoma | 1.88738e-15 | 53/55 | 228 | 142.688 |
| SYSTEM\_DEVELOPMENT (c5) Genes annotated by the GO term GO:0048731. The process whose specific outcome is the progression of an organismal system over time, from its formation to the mature structure. A system is a regularly interacting or interdependent group of organs or tissues that work together to carry out a given biological process. | 2.38365e-13 | 777/858 | 1589 | 1355.68 |
| ANATOMICAL\_STRUCTURE\_DEVELOPMENT (c5) Genes annotated by the GO term GO:0048856. The biological process whose specific outcome is the progression of an anatomical structure from an initial condition to its mature state. This process begins with the formation of the structure and ends with the mature structure, whatever form that may be including its natural destruction. An anatomical structure is any biological entity that occupies space and is distinguished from its surroundings. Anatomical structures can be macroscopic such as a carpel, or microscopic such as an acrosome. | 4.23439e-13 | 908/1012 | 1740 | 1499.7 |
| CENTRAL\_NERVOUS\_SYSTEM\_DEVELOPMENT (c5) Genes annotated by the GO term GO:0007417. The process whose specific outcome is the progression of the central nervous system over time, from its formation to the mature structure. The central nervous system is the core nervous system that serves an integrating and coordinating function. In vertebrates it consists of the brain, spinal cord and spinal nerves. In those invertebrates with a central nervous system it typically consists of a brain, cerebral ganglia and a nerve cord. | 4.70401e-13 | 109/123 | 194 | 120.83 |
| GROWTH\_CONE (c5) Genes annotated by the GO term GO:0030426. The migrating motile tip of a growing nerve cell axon or dendrite. | 8.82627e-13 | 9/10 | 68 | 32.01 |
| GATTGGY\_V$NFY\_Q6\_01 (c3) Genes with promoter regions [-2kb,2kb] around transcription start site containing motif GATTGGY. Motif does not match any known transcription factor | 3.01192e-12 | 703/856 | 1101 | 921.001 |
| MULTICELLULAR\_ORGANISMAL\_DEVELOPMENT (c5) Genes annotated by the GO term GO:0007275. The biological process whose specific outcome is the progression of an organism over time from an initial condition (e.g. a zygote or a young adult) to a later condition (e.g. a multicellular animal or an aged adult). | 5.80036e-12 | 926/1045 | 1813 | 1581.65 |
| SITE\_OF\_POLARIZED\_GROWTH (c5) Genes annotated by the GO term GO:0030427. Any part of a cell where non-isotropic growth takes place. | 5.98988e-12 | 10/11 | 69 | 33.837 |
| BRAIN\_DEVELOPMENT (c5) Genes annotated by the GO term GO:0007420. The process whose specific outcome is the progression of the brain over time, from its formation to the mature structure. The brain is one of the two components of the central nervous system and is the center of thought and emotion. It is responsible for the coordination and control of bodily activities and the interpretation of information from the senses (sight, hearing, smell, etc.). | 6.20404e-12 | 44/51 | 91 | 46.928 |
| REELINPATHWAY (c2) Reelin is secreted by neurons and recognized by receptors including cadherin related neuronal receptors, which promote phosphorylation of Dab1. | 8.24885e-12 | 6/7 | 59 | 27.206 |
| HSA04012\_ERBB\_SIGNALING\_PATHWAY (c2) Genes involved in ErbB signaling pathway | 9.37994e-12 | 85/87 | 687 | 557.852 |
| NEURITE\_DEVELOPMENT (c5) Genes annotated by the GO term GO:0031175. The process whose specific outcome is the progression of the neurite over time, from its formation to the mature structure. The neurite is any process extending from a neural cell, such as axons or dendrites. | 9.78106e-12 | 51/53 | 135 | 80.327 |
| NEURON\_DEVELOPMENT (c5) Genes annotated by the GO term GO:0048666. The process whose specific outcome is the progression of a neuron over time, from initial commitment of the cell to a specific fate, to the fully functional differentiated cell. | 1.13974e-11 | 59/61 | 152 | 93.248 |
| HSA04115\_P53\_SIGNALING\_PATHWAY (c2) Genes involved in p53 signaling pathway | 2.36468e-11 | 64/66 | 372 | 274.972 |
| CAGGTG\_V$E12\_Q6 (c3) Genes with promoter regions [-2kb,2kb] around transcription start site containing the motif CAGGTG which matches annotation for TCF3: transcription factor 3 (E2A immunoglobulin enhancer binding factors E12/E47) | 2.90037e-11 | 1486/1832 | 2044 | 1818.14 |
| module\_66 (c4) Genes in module\_66 | 3.68622e-11 | 488/543 | 763 | 622.577 |
| REGULATION\_OF\_CELL\_CYCLE (c5) Genes annotated by the GO term GO:0051726. Any process that modulates the rate or extent of progression through the cell cycle. | 4.35629e-11 | 174/180 | 534 | 424.345 |
| NEUROGENESIS (c5) Genes annotated by the GO term GO:0022008. Generation of cells within the nervous system. | 6.4947e-11 | 89/93 | 202 | 133.955 |
| EMBRYONIC\_MORPHOGENESIS (c5) Genes annotated by the GO term GO:0048598. The process by which anatomical structures are generated and organized during the embryonic phase. Morphogenesis pertains to the creation of form. The embryonic phase begins with zygote formation. The end of the embryonic phase is organism-specific. For example, it would be at birth for mammals, larval hatching for insects and seed dormancy in plants. | 1.94711e-10 | 14/17 | 53 | 24.286 |
| module\_100 (c4) Genes in module\_100 | 2.4755e-10 | 481/536 | 745 | 611.784 |
| SHHPATHWAY (c2) Sonic hedgehog (Shh) signaling in the developing CNS induces neuronal proliferation via interaction with the patched (Ptc-1) and smoothened receptors. | 2.52731e-10 | 12/14 | 62 | 30.655 |
| module\_137 (c4) Genes in module\_137 | 2.59723e-10 | 483/539 | 748 | 615.176 |
| KINASE\_ACTIVITY (c5) Genes annotated by the GO term GO:0016301. Catalysis of the transfer of a phosphate group, usually from ATP, to a substrate molecule. | 2.80249e-10 | 341/363 | 801 | 664.771 |
| HSA01510\_NEURODEGENERATIVE\_DISEASES (c2) Genes involved in neurodegenerative diseases | 3.98266e-10 | 37/38 | 311 | 229.605 |
| TRANSFERASE\_ACTIVITY\_\_TRANSFERRING\_PHOSPHORUS\_CONTAINING\_GROUPS (c5) Genes annotated by the GO term GO:0016772. Catalysis of the transfer of a phosphorus-containing group from one compound (donor) to another (acceptor). | 4.962e-10 | 386/418 | 821 | 684.581 |
| module\_274 (c4) Genes in module\_274 | 5.04999e-10 | 76/82 | 177 | 115.041 |
| GENERATION\_OF\_NEURONS (c5) Genes annotated by the GO term GO:0048699. The process by which nerve cells are generated. This includes the production of neuroblasts and their differentiation into neurons. | 6.65737e-10 | 79/83 | 184 | 122.398 |
| module\_220 (c4) Genes in module\_220 | 7.70727e-10 | 316/329 | 763 | 624.102 |
| FOSBPATHWAY (c2) FOSB gene expression and drug abuse | 1.2235e-09 | 4/5 | 37 | 15.618 |
| V$AP2\_Q3 (c3) Genes with promoter regions [-2kb,2kb] around transcription start site containing the motif GSCCSCRGGCNRNRNN which matches annotation for GTF3A: general transcription factor IIIA | 1.22651e-09 | 167/195 | 278 | 201.142 |
| SYNAPSE (c5) Genes annotated by the GO term GO:0045202. The junction between a nerve fiber of one neuron and another neuron or muscle fiber or glial cell; the site of interneuronal communication. As the nerve fiber approaches the synapse it enlarges into a specialized structure, the presynaptic nerve ending, which contains mitochondria and synaptic vesicles. At the tip of the nerve ending is the presynaptic membrane; facing it, and separated from it by a minute cleft (the synaptic cleft) is a specialized area of membrane on the receiving cell, known as the postsynaptic membrane. In response to the arrival of nerve impulses, the presynaptic nerve ending secretes molecules of neurotransmitters into the synaptic cleft. These diffuse across the cleft and transmit the signal to the postsynaptic membrane. | 1.87998e-09 | 25/27 | 76 | 40.554 |
| ANATOMICAL\_STRUCTURE\_MORPHOGENESIS (c5) Genes annotated by the GO term GO:0009653. The process by which anatomical structures are generated and organized. Morphogenesis pertains to the creation of form. | 2.08222e-09 | 345/379 | 761 | 628.993 |
| HSA05218\_MELANOMA (c2) Genes involved in melanoma | 3.02648e-09 | 65/71 | 535 | 432.61 |
| PHOSPHOTRANSFERASE\_ACTIVITY\_\_ALCOHOL\_GROUP\_AS\_ACCEPTOR (c5) Genes annotated by the GO term GO:0016773. Catalysis of the transfer of a phosphorus-containing group from one compound (donor) to an alcohol group (acceptor). | 3.75272e-09 | 309/329 | 762 | 636.373 |
| module\_11 (c4) Genes in module\_11 | 4.8264e-09 | 474/533 | 724 | 601.501 |
| YTAATTAA\_V$LHX3\_01 (c3) Genes with promoter regions [-2kb,2kb] around transcription start site containing the motif YTAATTAA which matches annotation for LHX3: LIM homeobox 3 | 6.06226e-09 | 119/145 | 147 | 94.469 |
| NEURON\_DIFFERENTIATION (c5) Genes annotated by the GO term GO:0030182. The process whereby a relatively unspecialized cell acquires specialized features of a neuron. | 8.91689e-09 | 72/76 | 166 | 111.833 |
| ABRAHAM\_AL\_VS\_MM\_DN (c2) Genes with significantly lower average gene expression in AL plasma cells than in MM cells | 8.95485e-09 | 17/18 | 174 | 120.033 |
| CELLCYCLEPATHWAY (c2) Cyclins interact with cyclin-dependent kinases to form active kinase complexes that regulate progression through the cell cycle. | 9.1313e-09 | 22/23 | 177 | 120.824 |
| AXON (c5) Genes annotated by the GO term GO:0030424. The long process of a neuron that conducts nerve impulses, usually away from the cell body to the terminals and varicosities, which are sites of storage and release of neurotransmitter. | 9.30386e-09 | 11/12 | 60 | 31.444 |
| GGGAGGRR\_V$MAZ\_Q6 (c3) Genes with promoter regions [-2kb,2kb] around transcription start site containing the motif GGGAGGRR which matches annotation for MAZ: MYC-associated zinc finger protein (purine-binding transcription factor) | 9.6074e-09 | 1470/1733 | 2145 | 1951.66 |
| TAATTA\_V$CHX10\_01 (c3) Genes with promoter regions [-2kb,2kb] around transcription start site containing the motif TAATTA which matches annotation for VSX1: visual system homeobox 1 homolog, CHX10-like (zebrafish) | 1.04239e-08 | 485/612 | 654 | 536.484 |
| V$LEF1\_Q6 (c3) Genes with promoter regions [-2kb,2kb] around transcription start site containing the motif SWWCAAAGGG which matches annotation for LEF1: lymphoid enhancer-binding factor 1  TCF1: transcription factor 1, hepatic; LF-B1, hepatic nuclear factor (HNF1), albumin proximal factor | 1.24104e-08 | 174/212 | 213 | 148.883 |
| CELL\_CYCLE\_GO\_0007049 | 1.40141e-08 | 299/311 | 695 | 583.465 |
| AXON\_GUIDANCE (c5) Genes annotated by the GO term GO:0007411. The process by which the migration of an axon growth cone is directed to a specific target site in response to a combination of attractive and repulsive cues. | 1.41707e-08 | 20/22 | 57 | 29.725 |
| RNGTGGGC\_UNKNOWN (c3) Genes with promoter regions [-2kb,2kb] around transcription start site containing motif RNGTGGGC. Motif does not match any known transcription factor | 1.4581e-08 | 476/564 | 708 | 589.189 |
| GLAND\_DEVELOPMENT (c5) Genes annotated by the GO term GO:0048732. The process whose specific outcome is the progression of a gland over time, from its formation to the mature structure. A gland is an organ specialised for secretion. | 1.48451e-08 | 12/13 | 71 | 39.476 |
| chr6p11 (c1) Genes in cytogenetic band chr6p11 | 1.51344e-08 | 3/6 | 4 | 0.446 |
| PROTEIN\_KINASE\_ACTIVITY (c5) Genes annotated by the GO term GO:0004672. Catalysis of the phosphorylation of an amino acid residue in a protein, usually according to the reaction: a protein + ATP = a phosphoprotein + ADP. | 1.55368e-08 | 269/280 | 722 | 603.959 |
| NEURON\_PROJECTION (c5) Genes annotated by the GO term GO:0043005. A prolongation or process extending from a nerve cell, e.g. an axon or dendrite. | 1.71021e-08 | 19/20 | 70 | 38.796 |
| V$TEF\_Q6 (c3) Genes with promoter regions [-2kb,2kb] around transcription start site containing the motif ATGTTWAYATAA which matches annotation for TEF: thyrotrophic embryonic factor | 2.04526e-08 | 162/195 | 246 | 180.703 |
| G1PATHWAY (c2) CDK4/6-cyclin D and CDK2-cyclin E phosphorylate Rb, which allows the transcription of genes needed for the G1/S cell cycle transition. | 2.19058e-08 | 25/26 | 267 | 200.827 |
| CELL\_DEVELOPMENT (c5) Genes annotated by the GO term GO:0048468. The process whose specific outcome is the progression of the cell over time, from its formation to the mature structure. Cell development does not include the steps involved in committing a cell to a specific fate. | 2.34726e-08 | 549/571 | 1633 | 1460.37 |
| V$CP2\_02 (c3) Genes with promoter regions [-2kb,2kb] around transcription start site containing the motif GCTGGNTNGNNCYNG which matches annotation for TFCP2: transcription factor CP2 | 2.59346e-08 | 157/181 | 333 | 258.492 |
| V$CHX10\_01 (c3) Genes with promoter regions [-2kb,2kb] around transcription start site containing the motif NNNTAATTAGCNNN which matches annotation for VSX1: visual system homeobox 1 homolog, CHX10-like (zebrafish) | 2.59435e-08 | 149/184 | 265 | 194.629 |
| HSA04310\_WNT\_SIGNALING\_PATHWAY (c2) Genes involved in Wnt signaling pathway | 2.70693e-08 | 138/147 | 614 | 508.226 |
| V$T3R\_Q6 (c3) Genes with promoter regions [-2kb,2kb] around transcription start site containing motif MNTGWCCTN. Motif does not match any known transcription factor | 3.10419e-08 | 160/194 | 207 | 145.617 |
| TTGTTT\_V$FOXO4\_01 (c3) Genes with promoter regions [-2kb,2kb] around transcription start site containing the motif TTGTTT which matches annotation for MLLT7: myeloid/lymphoid or mixed-lineage leukemia (trithorax homolog, Drosophila); translocated to, 7 | 3.12866e-08 | 1248/1511 | 1725 | 1545.99 |
| chr7q36 (c1) Genes in cytogenetic band chr7q36 | 3.50412e-08 | 31/68 | 50 | 25.208 |
| CELL\_PROJECTION (c5) Genes annotated by the GO term GO:0042995. A prolongation or process extending from a cell, e.g. a flagellum or axon. | 3.53275e-08 | 96/108 | 185 | 127.935 |
| HSA05214\_GLIOMA (c2) Genes involved in glioma | 3.61504e-08 | 61/64 | 536 | 438.826 |
| CORTEX\_ENRICHMENT\_LATE\_UP (c2) Up-regulated in the cortex of mice that are exposed to an enriched environmental habitat for 2 or 14 days | 3.67441e-08 | 18/20 | 52 | 26.316 |
| ST\_INTEGRIN\_SIGNALING\_PATHWAY (c2) Integrins are transmembrane receptors that mediate cell growth, survival, and migration by binding to ligands in the extracellular matrix. | 4.0336e-08 | 77/79 | 440 | 355.184 |
| V$SREBP\_Q3 (c3) Genes with promoter regions [-2kb,2kb] around transcription start site containing motif VNNVTCACCCYA. Motif does not match any known transcription factor | 4.63041e-08 | 162/186 | 321 | 244.335 |
| SA\_REG\_CASCADE\_OF\_CYCLIN\_EXPR (c2) Expression of cyclins regulates progression through the cell cycle by activating cyclin-dependent kinases. | 6.38388e-08 | 12/13 | 121 | 79.819 |
| AXONOGENESIS (c5) Genes annotated by the GO term GO:0007409. Generation of a long process of a neuron, that carries efferent (outgoing) action potentials from the cell body towards target cells. | 6.43869e-08 | 41/43 | 96 | 58.846 |
| S\_PHASE\_OF\_MITOTIC\_CELL\_CYCLE (c5) Genes annotated by the GO term GO:0000084. Progression through S phase, the part of the mitotic cell cycle during which DNA synthesis takes place. | 8.75142e-08 | 9/10 | 35 | 15.534 |
| WCAANNNYCAG\_UNKNOWN (c3) Genes with promoter regions [-2kb,2kb] around transcription start site containing motif WCAANNNYCAG. Motif does not match any known transcription factor | 9.39854e-08 | 143/184 | 265 | 197.492 |
| CELL\_CYCLE\_REGULATOR (c2) Obsolete by GO - was not defined before being made obsolete | 1.18205e-07 | 19/21 | 105 | 67.102 |
| CELLULAR\_MORPHOGENESIS\_DURING\_DIFFERENTIATION (c5) Genes annotated by the GO term GO:0000904. The change in form (cell shape and size) that occurs when relatively unspecialized cells, e.g. embryonic or regenerative cells, acquire specialized structural and/or functional features that characterize the cells, tissues, or organs of the mature organism or some other relatively stable phase of the organism's life history. | 1.2845e-07 | 46/49 | 101 | 63.381 |
| TRANSMEMBRANE\_RECEPTOR\_PROTEIN\_TYROSINE\_KINASE\_SIGNALING\_PATHWAY (c5) Genes annotated by the GO term GO:0007169. The series of molecular signals generated as a consequence of a transmembrane receptor tyrosine kinase binding to its physiological ligand. | 1.45376e-07 | 81/83 | 432 | 348.704 |
| CCTGTGA,MIR-513 (c3) Targets of MicroRNA CCTGTGA,MIR-513 | 1.64665e-07 | 84/111 | 144 | 98.407 |
| V$HOX13\_01 (c3) Genes with promoter regions [-2kb,2kb] around transcription start site containing the motif TGCNHNCWYCCYCATTAKTNNDCNMNHYCN which matches annotation for HOXA5: homeobox A5 | 1.7129e-07 | 25/32 | 80 | 48.52 |
| V$HEN1\_01 (c3) Genes with promoter regions [-2kb,2kb] around transcription start site containing the motif NNNGGNCNCAGCTGCGNCCCNN which matches annotation for NHLH1: nescient helix loop helix 1 | 1.87583e-07 | 123/155 | 245 | 185.325 |
| CYSTEINE\_TYPE\_PEPTIDASE\_ACTIVITY (c5) Genes annotated by the GO term GO:0008234. Catalysis of the hydrolysis of peptide linkages in oligopeptides or polypeptides; a cysteine residue is at the active center. | 2.00676e-07 | 43/54 | 122 | 80.792 |
| V$COMP1\_01 (c3) Genes with promoter regions [-2kb,2kb] around transcription start site containing the motif NVTNWTGATTGACNACAAVARRBN which matches annotation for MYOG: myogenin (myogenic factor 4) | 2.12869e-07 | 86/94 | 141 | 96.022 |
| HSA05215\_PROSTATE\_CANCER (c2) Genes involved in prostate cancer | 2.14689e-07 | 83/87 | 698 | 595.474 |
| V$IK2\_01 (c3) Genes with promoter regions [-2kb,2kb] around transcription start site containing motif NNNTGGGAWNNC. Motif does not match any known transcription factor | 2.81119e-07 | 177/206 | 314 | 242.861 |
| V$HNF6\_Q6 (c3) Genes with promoter regions [-2kb,2kb] around transcription start site containing the motif HWAAATCAATAW which matches annotation for ONECUT1: one cut domain, family member 1 | 2.96481e-07 | 150/183 | 333 | 263.175 |
| V$TFIII\_Q6 (c3) Genes with promoter regions [-2kb,2kb] around transcription start site containing the motif RGAGGKAGG which matches annotation for GTF2A1: general transcription factor IIA, 1, 19/37kDa  GTF2A2: general transcription factor IIA, 2, 12kDa | 3.10173e-07 | 146/165 | 254 | 190.353 |
| V$EGR1\_01 | 3.55037e-07 | 174/195 | 345 | 271.428 |
| CAGCTG\_V$AP4\_Q5 (c3) Genes with promoter regions [-2kb,2kb] around transcription start site containing the motif CAGCTG which matches annotation for REPIN1: replication initiator 1 | 3.58837e-07 | 926/1126 | 1376 | 1231.11 |
| P35ALZHEIMERSPATHWAY (c2) p35, a neuron-specific activator of cyclin-dependent kinase 5, is cleaved to p25 in Alzheimer's disease and promotoes hyperphosphorylated tau formation and apoptosis. | 4.01063e-07 | 10/11 | 82 | 50.627 |
| CATTGTYY\_V$SOX9\_B1 (c3) Genes with promoter regions [-2kb,2kb] around transcription start site containing the motif CATTGTYY which matches annotation for SOX9: SRY (sex determining region Y)-box 9 (campomelic dysplasia, autosomal sex-reversal) | 4.05937e-07 | 239/279 | 451 | 365.778 |
| V$IK3\_01 (c3) Genes with promoter regions [-2kb,2kb] around transcription start site containing motif TNYTGGGAATACC. Motif does not match any known transcription factor | 5.14216e-07 | 141/169 | 218 | 159.953 |
| RORIE\_ES\_PNET\_DN (c2) The 30 genes showing the greatest decrease in expression in NBa Ews/Fli-1 infectants | 5.27034e-07 | 24/26 | 56 | 30.005 |
| MORF\_MYC (c4) Neighborhood of MYC | 5.73832e-07 | 64/74 | 160 | 113.278 |
| ABRAHAM\_MM\_VS\_AL\_UP (c2) Genes highly expressed in multiple myeloma (MM) versus immunoglobulin light chain amyloidosis (AL) in plasma cells. | 6.00725e-07 | 18/19 | 205 | 153.552 |
| CTGCAGY\_UNKNOWN (c3) Genes with promoter regions [-2kb,2kb] around transcription start site containing motif CTGCAGY. Motif does not match any known transcription factor | 6.02226e-07 | 469/568 | 789 | 677.57 |
| chr20p11 (c1) Genes in cytogenetic band chr20p11 | 6.43895e-07 | 26/68 | 30 | 13.31 |
| V$PAX4\_03 (c3) Genes with promoter regions [-2kb,2kb] around transcription start site containing the motif NNNNNYCACCCB which matches annotation for PAX4: paired box gene 4 | 6.98723e-07 | 169/200 | 296 | 228.045 |
| CAGGTA\_V$AREB6\_01 (c3) Genes with promoter regions [-2kb,2kb] around transcription start site containing the motif CAGGTA which matches annotation for TCF8: transcription factor 8 (represses interleukin 2 expression) | 7.09681e-07 | 471/582 | 735 | 626.712 |
| V$E2F1\_Q3\_01 (c3) Genes with promoter regions [-2kb,2kb] around transcription start site containing the motif TTGGCGCGRAANNGNM which matches annotation for E2F1: E2F transcription factor 1 | 8.62706e-07 | 168/193 | 270 | 206.213 |
| NEURON\_APOPTOSIS (c5) Genes annotated by the GO term GO:0051402. The process of apoptosis in neurons, the basic cellular unit of nervous tissue. Each neuron consists of a body, an axon, and dendrites. Their purpose is to receive, conduct, and transmit impulses in the nervous system. | 1.08006e-06 | 16/17 | 105 | 70.005 |
| NEGATIVE\_REGULATION\_OF\_CELL\_CYCLE (c5) Genes annotated by the GO term GO:0045786. Any process that stops, prevents or reduces the rate or extent of progression through the cell cycle. | 1.14723e-06 | 74/77 | 279 | 219.906 |
| V$NFY\_Q6\_01 (c3) Genes with promoter regions [-2kb,2kb] around transcription start site containing motif NNNNRRCCAATSR. Motif does not match any known transcription factor | 1.24945e-06 | 159/197 | 332 | 265.593 |
| MITOCHONDRIAPATHWAY (c2) Pro-apoptotic signaling induces mitochondria to release cytochrome c, which stimulates Apaf-1 to activate caspase 9. | 1.26962e-06 | 18/19 | 151 | 109.894 |
| PROTEIN\_PROCESSING (c5) Genes annotated by the GO term GO:0016485. The posttranslational modification of a protein, particularly secretory proteins and proteins targeted for membranes or specific cellular locations. | 1.29941e-06 | 45/47 | 140 | 99.31 |
| AACTTT\_UNKNOWN (c3) Genes with promoter regions [-2kb,2kb] around transcription start site containing motif AACTTT. Motif does not match any known transcription factor | 1.35715e-06 | 1218/1458 | 1837 | 1678.26 |
| V$LMO2COM\_01 (c3) Genes with promoter regions [-2kb,2kb] around transcription start site containing the motif CNNCAGGTGBNN which matches annotation for LMO2: LIM domain only 2 (rhombotin-like 1) | 1.47688e-06 | 159/195 | 268 | 204.605 |
| POD1\_KO\_MOST\_UP (c2) Most strongly up-regulated in glomeruli isolated from Pod1 knockout mice, versus wild-type controls | 1.51285e-06 | 26/28 | 82 | 51.168 |
| V$SP1\_Q2\_01 (c3) Genes with promoter regions [-2kb,2kb] around transcription start site containing the motif CCCCGCCCCN which matches annotation for SP1: Sp1 transcription factor | 1.58004e-06 | 171/191 | 296 | 229.537 |
| V$NKX25\_02 (c3) Genes with promoter regions [-2kb,2kb] around transcription start site containing the motif CWTAATTG which matches annotation for NKX2-5: NK2 transcription factor related, locus 5 (Drosophila) | 1.63542e-06 | 163/203 | 266 | 204.994 |
| HSA05223\_NON\_SMALL\_CELL\_LUNG\_CANCER (c2) Genes involved in non-small cell lung cancer | 1.65054e-06 | 53/54 | 461 | 383.649 |
| HSA04510\_FOCAL\_ADHESION (c2) Genes involved in focal adhesion | 1.72815e-06 | 184/192 | 916 | 807.903 |
| ST\_DIFFERENTIATION\_PATHWAY\_IN\_PC12\_CELLS (c2) Rat-derived PC12 cells respond to nerve growth factor (NGF) and PACAP to differentiate into neuronal cells. | 1.74453e-06 | 40/42 | 392 | 319.303 |
| V$TCF11\_01 (c3) Genes with promoter regions [-2kb,2kb] around transcription start site containing the motif GTCATNNWNNNNN which matches annotation for NFE2L1: nuclear factor (erythroid-derived 2)-like 1 | 1.83485e-06 | 148/174 | 293 | 230.417 |
| MORF\_ARL3 (c4) Neighborhood of ARL3 | 2.1805e-06 | 221/288 | 397 | 322.555 |
| AAAYWAACM\_V$HFH4\_01 (c3) Genes with promoter regions [-2kb,2kb] around transcription start site containing the motif AAAYWAACM which matches annotation for FOXJ1: forkhead box J1 | 2.2727e-06 | 163/195 | 251 | 194.401 |
| V$FOX\_Q2 (c3) Genes with promoter regions [-2kb,2kb] around transcription start site containing the motif KATTGTTTRTTTW which matches annotation for FOXF2: forkhead box F2 | 2.47811e-06 | 135/163 | 189 | 139.688 |
| V$IPF1\_Q4 (c3) Genes with promoter regions [-2kb,2kb] around transcription start site containing the motif GHNNTAATGACM which matches annotation for IPF1: insulin promoter factor 1, homeodomain transcription factor | 2.50772e-06 | 159/186 | 248 | 189.325 |
| DNA\_BINDING (c5) Genes annotated by the GO term GO:0003677. Interacting selectively with DNA (deoxyribonucleic acid). | 2.6551e-06 | 540/600 | 1123 | 1001.54 |
| CACAMPATHWAY (c2) Calcium functions as a second messenger activating the calcium/calmodulin-dependent kinases, which phosphorylate targets such as CREB. | 2.82538e-06 | 13/14 | 71 | 43.807 |
| GCM\_MAPK10 (c4) Neighborhood of MAPK10 | 3.01824e-06 | 61/78 | 88 | 56.436 |
| PROTEIN\_SERINE\_THREONINE\_KINASE\_ACTIVITY (c5) Genes annotated by the GO term GO:0004674. Catalysis of the reaction: ATP + a protein serine/threonine = ADP + protein serine/threonine phosphate. | 3.05972e-06 | 193/201 | 483 | 402.759 |
| HSA05010\_ALZHEIMERS\_DISEASE (c2) Genes involved in Alzheimer's disease | 3.27486e-06 | 27/28 | 231 | 180.414 |
| WNT\_SIGNALING (c2) Wnt signaling genes | 3.59203e-06 | 58/61 | 297 | 236.463 |
| HSA00534\_HEPARAN\_SULFATE\_BIOSYNTHESIS (c2) Genes involved in heparan sulfate biosynthesis | 3.89668e-06 | 6/19 | 8 | 1.992 |
| MAPKKK\_CASCADE\_GO\_0000165 (c5) Genes annotated by the GO term GO:0000165. Cascade of at least three protein kinase activities culminating in the phosphorylation and activation of a MAP kinase. MAPKKK cascades lie downstream of numerous signaling pathways. | 3.95085e-06 | 101/102 | 321 | 255.612 |
| CYSTEINE\_TYPE\_ENDOPEPTIDASE\_ACTIVITY (c5) Genes annotated by the GO term GO:0004197. Catalysis of the hydrolysis of nonterminal peptide linkages in oligopeptides or polypeptides; a cysteine residue is at the active center. | 3.99826e-06 | 31/40 | 106 | 71.409 |
| TRANSCRIPTION\_FACTOR\_ACTIVITY (c5) Genes annotated by the GO term GO:0003700. The function of binding to a specific DNA sequence in order to modulate transcription. The transcription factor may or may not also interact selectively with a protein or macromolecular complex. | 4.27912e-06 | 316/353 | 823 | 720.056 |
| V$SMAD4\_Q6 (c3) Genes with promoter regions [-2kb,2kb] around transcription start site containing the motif GKSRKKCAGMCANCY which matches annotation for SMAD4: SMAD, mothers against DPP homolog 4 (Drosophila) | 4.94727e-06 | 153/180 | 259 | 203.081 |
| MORF\_STK17A (c4) Neighborhood of STK17A | 5.04989e-06 | 121/156 | 272 | 215.598 |
| V$NKX62\_Q2 (c3) Genes with promoter regions [-2kb,2kb] around transcription start site containing the motif NWADTAAWTANN which matches annotation for NKX6-2: NK6 transcription factor related, locus 2 (Drosophila) | 5.66415e-06 | 151/186 | 238 | 182.952 |
| MORF\_TNFRSF25 (c4) Neighborhood of TNFRSF25 | 6.1403e-06 | 196/243 | 358 | 292.572 |
| MORF\_BNIP1 (c4) Neighborhood of BNIP1 | 6.35379e-06 | 136/180 | 290 | 231.831 |
| STRESS\_ACTIVATED\_PROTEIN\_KINASE\_SIGNALING\_PATHWAY (c5) Genes annotated by the GO term GO:0031098. A series of molecular signals in which a stress-activated protein kinase (SAPK) cascade relays one or more of the signals. | 6.58211e-06 | 46/47 | 145 | 104.845 |
| CTTTGT\_V$LEF1\_Q2 (c3) Genes with promoter regions [-2kb,2kb] around transcription start site containing the motif CTTTGT which matches annotation for LEF1: lymphoid enhancer-binding factor 1 | 6.62771e-06 | 1197/1460 | 1594 | 1451.49 |
| AMYLOID\_PRECURSOR\_PROTEIN\_METABOLIC\_PROCESS (c5) Genes annotated by the GO term GO:0042982. The chemical reactions and pathways involving amyloid precursor protein (APP), the precursor of beta-amyloid, a glycoprotein associated with Alzheimer's disease. | 6.96834e-06 | 9/10 | 27 | 12.697 |
| BRENTANI\_CELL\_CYCLE (c2) Cancer related genes involved in the cell cycle | 7.33333e-06 | 78/79 | 338 | 275.364 |
| GCACTTT,MIR-17-5P,MIR-20A,MIR-106A,MIR-106B,MIR-20B,MIR-519D (c3) Targets of MicroRNA GCACTTT,MIR-17-5P,MIR-20A,MIR-106A,MIR-106B,MIR-20B,MIR-519D | 8.11158e-06 | 394/528 | 582 | 498.547 |
| V$MEIS1AHOXA9\_01 (c3) Genes with promoter regions [-2kb,2kb] around transcription start site containing the motif TGACAGKTTTAYGA which matches annotation for MEIS1: Meis1, myeloid ecotropic viral integration site 1 homolog (mouse)  HOXA9: homeobox A9 | 9.07398e-06 | 77/93 | 124 | 87.712 |
| V$MYOD\_Q6\_01 | 9.07588e-06 | 156/185 | 287 | 229.555 |
| JNK\_CASCADE (c5) Genes annotated by the GO term GO:0007254. A cascade of protein kinase activities, culminating in the phosphorylation and activation of a member of the JUN kinase subfamily of stress-activated protein kinases, which in turn are a subfamily of mitogen-activated protein (MAP) kinases that is activated primarily by cytokines and exposure to environmental stress. | 1.01569e-05 | 44/45 | 141 | 102.065 |
| TGCCTTA,MIR-124A (c3) Targets of MicroRNA TGCCTTA,MIR-124A | 1.0381e-05 | 375/495 | 537 | 454.622 |
| MUSCLE\_CELL\_DIFFERENTIATION (c5) Genes annotated by the GO term GO:0042692. The process whereby a relatively unspecialized cell acquires specialized features of a muscle cell. | 1.06346e-05 | 21/22 | 66 | 40.393 |
| V$POU1F1\_Q6 (c3) Genes with promoter regions [-2kb,2kb] around transcription start site containing the motif ATGAATAAWT which matches annotation for POU1F1: POU domain, class 1, transcription factor 1 (Pit1, growth hormone factor 1) | 1.177e-05 | 157/183 | 260 | 204.389 |
| HSA04010\_MAPK\_SIGNALING\_PATHWAY (c2) Genes involved in MAPK signaling pathway | 1.35871e-05 | 229/252 | 1113 | 1004.61 |
| CHESLER\_D6MIT150\_NEURAL\_TARGETS\_GLOCUS (c2) Neurologically relevant downstream targets of an important regulatory locus. This locus is on chromosome 6 and near the D6Mit150 locus. | 1.36233e-05 | 7/8 | 50 | 30.175 |
| PROTEIN\_MODIFICATION\_PROCESS (c5) Genes annotated by the GO term GO:0006464. The covalent alteration of one or more amino acids occurring in proteins, peptides and nascent polypeptides (co-translational, post-translational modifications). Includes the modification of charged tRNAs that are destined to occur in a protein (pre-translation modification). | 1.46467e-05 | 569/623 | 1087 | 971.691 |
| V$PITX2\_Q2 (c3) Genes with promoter regions [-2kb,2kb] around transcription start site containing the motif WNTAATCCCAR which matches annotation for PITX2: paired-like homeodomain transcription factor 2 | 1.46956e-05 | 161/206 | 338 | 274.359 |
| V$SP3\_Q3 (c3) Genes with promoter regions [-2kb,2kb] around transcription start site containing the motif ASMCTTGGGSRGGG which matches annotation for SP3: Sp3 transcription factor | 1.50764e-05 | 154/187 | 267 | 211.905 |
| AGGTGCA,MIR-500 (c3) Targets of MicroRNA AGGTGCA,MIR-500 | 1.51538e-05 | 64/82 | 88 | 58.023 |
| V$PPARA\_02 (c3) Genes with promoter regions [-2kb,2kb] around transcription start site containing the motif NNRGGTCATWGGGGTSANG which matches annotation for PPARA: peroxisome proliferative activated receptor, alpha | 1.52052e-05 | 83/96 | 114 | 79.6 |
| V$S8\_01 (c3) Genes with promoter regions [-2kb,2kb] around transcription start site containing the motif WNNANYYAATTANCNN which matches annotation for PRRX2: paired related homeobox 2 | 1.55675e-05 | 154/195 | 265 | 212.014 |
| V$PBX1\_01 (c3) Genes with promoter regions [-2kb,2kb] around transcription start site containing the motif ANCAATCAW which matches annotation for PBX1: pre-B-cell leukemia transcription factor 1 | 1.55736e-05 | 164/191 | 266 | 210.983 |
| SHEPARD\_CRASH\_AND\_BURN\_MUT\_VS\_WT\_DN (c2) Genes upregulated in zebra fish wild type compared to the crash and burn mutant | 1.56084e-05 | 133/150 | 192 | 142.776 |
| V$FOXM1\_01 (c3) Genes with promoter regions [-2kb,2kb] around transcription start site containing the motif ARATKGAST which matches annotation for FOXM1: forkhead box M1 | 1.71386e-05 | 160/188 | 284 | 228.626 |
| V$NRSF\_01 (c3) Genes with promoter regions [-2kb,2kb] around transcription start site containing the motif TTCAGCACCACGGACAGMGCC which matches annotation for REST: RE1-silencing transcription factor | 1.77935e-05 | 63/76 | 124 | 87.384 |
| module\_12 (c4) Genes in module\_12 | 1.80708e-05 | 315/354 | 634 | 548.596 |
| V$PAX3\_01 (c3) Genes with promoter regions [-2kb,2kb] around transcription start site containing the motif TCGTCACRCTTHM which matches annotation for PAX3: paired box gene 3 (Waardenburg syndrome 1) | 1.87605e-05 | 12/15 | 25 | 11.607 |
| V$CRX\_Q4 (c3) Genes with promoter regions [-2kb,2kb] around transcription start site containing the motif YNNNTAATCYCMN which matches annotation for CRX: cone-rod homeobox | 1.89318e-05 | 175/210 | 249 | 195.201 |
| MORF\_TFDP2 (c4) Neighborhood of TFDP2 | 1.97461e-05 | 168/216 | 331 | 270.735 |
| V$SOX5\_01 (c3) Genes with promoter regions [-2kb,2kb] around transcription start site containing the motif NNAACAATNN which matches annotation for SOX5: SRY (sex determining region Y)-box 5 | 2.00301e-05 | 166/195 | 273 | 219.994 |
| chr5p11 (c1) Genes in cytogenetic band chr5p11 | 2.29118e-05 | 0/1 | 2 | 0.201 |
| MORF\_FDXR (c4) Neighborhood of FDXR | 2.31465e-05 | 175/208 | 268 | 213.011 |
| MORF\_LMO1 (c4) Neighborhood of LMO1 | 2.42245e-05 | 40/47 | 74 | 48.551 |
| PROTEIN\_AMINO\_ACID\_AUTOPHOSPHORYLATION (c5) Genes annotated by the GO term GO:0046777. The phosphorylation by a protein of one or more of its own amino acid residues, or residues on an identical protein. | 2.4858e-05 | 27/29 | 107 | 76.88 |
| SKELETAL\_DEVELOPMENT (c5) Genes annotated by the GO term GO:0001501. The process whose specific outcome is the progression of the skeleton over time, from its formation to the mature structure. The skeleton is the bony framework of the body in vertebrates (endoskeleton) or the hard outer envelope of insects (exoskeleton or dermoskeleton). | 2.5489e-05 | 95/102 | 232 | 181.453 |
| IGLESIAS\_E2FMINUS\_DN (c2) Genes that decrease in the absence of E2F1 and E2F2 | 2.60414e-05 | 13/14 | 50 | 30.519 |
| ORGAN\_MORPHOGENESIS (c5) Genes annotated by the GO term GO:0009887. Morphogenesis of an organ. An organ is defined as a tissue or set of tissues that work together to perform a specific function or functions. Morphogenesis is the process by which anatomical structures are generated and organized. Organs are commonly observed as visibly distinct structures, but may also exist as loosely associated clusters of cells that work together to perform a specific function or functions. | 2.61381e-05 | 136/145 | 402 | 335.568 |
| CGGTGTG,MIR-220 (c3) Targets of MicroRNA CGGTGTG,MIR-220 | 2.75548e-05 | 4/6 | 17 | 6.774 |
| CASPASEPATHWAY (c2) Caspases are cysteine proteases active in apoptosis; caspase-8 and 9 cleave and activate other caspases, while 3, 6, and 7 cleave cellular targets. | 2.80863e-05 | 20/21 | 117 | 84.068 |
| ABRAHAM\_AL\_VS\_MM\_UP (c2) Genes with significantly higher average gene expression in AL plasma cells compared to MM | 2.89733e-05 | 22/23 | 193 | 149.593 |
