# Supplementary material for: Looking at Cerebellar Malformations through Text-Mined Interactomes of Mice and Humans
Source: PLoS Comput Biol. 2009 Nov 6;5(11):e1000559. doi: 10.1371/journal.pcbi.1000559 (PMC2767227; doi:10.1371/journal.pcbi.1000559)
Supplement: Dataset S1 — All enrichment results. (0.20 MB ZIP) [file pcbi.1000559.s012.zip › enrichment_results/Table U. enrichment_whole-ataxia.html]

Complete Clustering results for network whole and phenotype ataxia (FDR <= 0.001)


# Complete Clustering results for network whole and phenotype ataxia (FDR <= 0.001)

| Set | p-Value | Gene Count | Interaction Count | Expected Interection Count |
| --- | --- | --- | --- | --- |
| NERVOUS\_SYSTEM\_DEVELOPMENT (c5) Genes annotated by the GO term GO:0007399. The process whose specific outcome is the progression of nervous tissue over time, from its formation to its mature state. | 1.22125e-15 | 331/382 | 432 | 305.419 |
| module\_274 (c4) Genes in module\_274 | 9.32587e-15 | 76/82 | 138 | 76.175 |
| HSA01510\_NEURODEGENERATIVE\_DISEASES (c2) Genes involved in neurodegenerative diseases | 1.88738e-14 | 37/38 | 231 | 151.774 |
| TRANSFERASE\_ACTIVITY\_\_TRANSFERRING\_PHOSPHORUS\_CONTAINING\_GROUPS (c5) Genes annotated by the GO term GO:0016772. Catalysis of the transfer of a phosphorus-containing group from one compound (donor) to another (acceptor). | 6.4615e-14 | 386/418 | 596 | 458.119 |
| KINASE\_ACTIVITY (c5) Genes annotated by the GO term GO:0016301. Catalysis of the transfer of a phosphate group, usually from ATP, to a substrate molecule. | 1.07137e-13 | 341/363 | 580 | 445.02 |
| HSA04115\_P53\_SIGNALING\_PATHWAY (c2) Genes involved in p53 signaling pathway | 5.94857e-13 | 64/66 | 267 | 184.343 |
| PHOSPHOTRANSFERASE\_ACTIVITY\_\_ALCOHOL\_GROUP\_AS\_ACCEPTOR (c5) Genes annotated by the GO term GO:0016773. Catalysis of the transfer of a phosphorus-containing group from one compound (donor) to an alcohol group (acceptor). | 4.10294e-12 | 309/329 | 549 | 426.171 |
| REELINPATHWAY (c2) Reelin is secreted by neurons and recognized by receptors including cadherin related neuronal receptors, which promote phosphorylation of Dab1. | 4.67115e-12 | 6/7 | 45 | 18.138 |
| PROTEIN\_KINASE\_ACTIVITY (c5) Genes annotated by the GO term GO:0004672. Catalysis of the phosphorylation of an amino acid residue in a protein, usually according to the reaction: a protein + ATP = a phosphoprotein + ADP. | 9.54936e-12 | 269/280 | 521 | 404.671 |
| HSA05010\_ALZHEIMERS\_DISEASE (c2) Genes involved in Alzheimer's disease | 4.17477e-11 | 27/28 | 178 | 118.681 |
| CELL\_DEVELOPMENT (c5) Genes annotated by the GO term GO:0048468. The process whose specific outcome is the progression of the cell over time, from its formation to the mature structure. Cell development does not include the steps involved in committing a cell to a specific fate. | 2.92379e-10 | 549/571 | 1132 | 970.059 |
| ST\_INTEGRIN\_SIGNALING\_PATHWAY (c2) Integrins are transmembrane receptors that mediate cell growth, survival, and migration by binding to ligands in the extracellular matrix. | 5.92866e-10 | 77/79 | 311 | 234.963 |
| TRANSMISSION\_OF\_NERVE\_IMPULSE (c5) Genes annotated by the GO term GO:0019226. The sequential electrochemical polarization and depolarization that travels across the membrane of a nerve cell (neuron) in response to stimulation. | 3.63124e-09 | 163/188 | 204 | 142.051 |
| SYNAPSE (c5) Genes annotated by the GO term GO:0045202. The junction between a nerve fiber of one neuron and another neuron or muscle fiber or glial cell; the site of interneuronal communication. As the nerve fiber approaches the synapse it enlarges into a specialized structure, the presynaptic nerve ending, which contains mitochondria and synaptic vesicles. At the tip of the nerve ending is the presynaptic membrane; facing it, and separated from it by a minute cleft (the synaptic cleft) is a specialized area of membrane on the receiving cell, known as the postsynaptic membrane. In response to the arrival of nerve impulses, the presynaptic nerve ending secretes molecules of neurotransmitters into the synaptic cleft. These diffuse across the cleft and transmit the signal to the postsynaptic membrane. | 7.47244e-09 | 25/27 | 55 | 27.054 |
| SYNAPTIC\_TRANSMISSION (c5) Genes annotated by the GO term GO:0007268. The process of communication from a neuron to a target (neuron, muscle, or secretory cell) across a synapse. | 1.01617e-08 | 150/173 | 187 | 130.004 |
| MITOCHONDRIAPATHWAY (c2) Pro-apoptotic signaling induces mitochondria to release cytochrome c, which stimulates Apaf-1 to activate caspase 9. | 1.48275e-08 | 18/19 | 116 | 72.537 |
| CYSTEINE\_TYPE\_PEPTIDASE\_ACTIVITY (c5) Genes annotated by the GO term GO:0008234. Catalysis of the hydrolysis of peptide linkages in oligopeptides or polypeptides; a cysteine residue is at the active center. | 1.52288e-08 | 43/54 | 91 | 53.576 |
| PROTEIN\_SERINE\_THREONINE\_KINASE\_ACTIVITY (c5) Genes annotated by the GO term GO:0004674. Catalysis of the reaction: ATP + a protein serine/threonine = ADP + protein serine/threonine phosphate. | 1.5692e-08 | 193/201 | 347 | 268.218 |
| CELL\_PROJECTION (c5) Genes annotated by the GO term GO:0042995. A prolongation or process extending from a cell, e.g. a flagellum or axon. | 2.19892e-08 | 96/108 | 133 | 85.684 |
| APOPTOSIS\_GO (c5) Genes annotated by the GO term GO:0006915. A form of programmed cell death induced by external or internal signals that trigger the activity of proteolytic caspases, whose actions dismantle the cell and result in cell death. Apoptosis begins internally with condensation and subsequent fragmentation of the cell nucleus (blebbing) while the plasma membrane remains intact. Other characteristics of apoptosis include DNA fragmentation and the exposure of phosphatidyl serine on the cell surface. | 2.25272e-08 | 410/425 | 972 | 839.122 |
| PROGRAMMED\_CELL\_DEATH (c5) Genes annotated by the GO term GO:0012501. Cell death resulting from activation of endogenous cellular processes. | 2.27613e-08 | 411/426 | 972 | 839.18 |
| module\_66 (c4) Genes in module\_66 | 2.89983e-08 | 488/543 | 514 | 414.747 |
| STRESS\_ACTIVATED\_PROTEIN\_KINASE\_SIGNALING\_PATHWAY (c5) Genes annotated by the GO term GO:0031098. A series of molecular signals in which a stress-activated protein kinase (SAPK) cascade relays one or more of the signals. | 2.97282e-08 | 46/47 | 111 | 69.703 |
| CYSTEINE\_TYPE\_ENDOPEPTIDASE\_ACTIVITY (c5) Genes annotated by the GO term GO:0004197. Catalysis of the hydrolysis of nonterminal peptide linkages in oligopeptides or polypeptides; a cysteine residue is at the active center. | 4.99997e-08 | 31/40 | 81 | 47.44 |
| V$EGR1\_01 (c3) Genes with promoter regions [-2kb,2kb] around transcription start site containing the motif WTGCGTGGGCGK which matches annotation for EGR1: early growth response 1 | 6.41838e-08 | 174/195 | 242 | 179.454 |
| JNK\_CASCADE (c5) Genes annotated by the GO term GO:0007254. A cascade of protein kinase activities, culminating in the phosphorylation and activation of a member of the JUN kinase subfamily of stress-activated protein kinases, which in turn are a subfamily of mitogen-activated protein (MAP) kinases that is activated primarily by cytokines and exposure to environmental stress. | 7.34514e-08 | 44/45 | 107 | 67.809 |
| module\_100 (c4) Genes in module\_100 | 7.73009e-08 | 481/536 | 503 | 407.59 |
| ABRAHAM\_AL\_VS\_MM\_DN (c2) Genes with significantly lower average gene expression in AL plasma cells than in MM cells | 7.92513e-08 | 17/18 | 121 | 79.902 |
| GLUTAMATE\_RECEPTOR\_ACTIVITY (c5) Genes annotated by the GO term GO:0008066. Combining with glutamate to initiate a change in cell activity. | 9.66585e-08 | 19/20 | 38 | 17.515 |
| ST\_DIFFERENTIATION\_PATHWAY\_IN\_PC12\_CELLS (c2) Rat-derived PC12 cells respond to nerve growth factor (NGF) and PACAP to differentiate into neuronal cells. | 1.00604e-07 | 40/42 | 269 | 209.61 |
| module\_137 (c4) Genes in module\_137 | 1.13267e-07 | 483/539 | 504 | 409.576 |
| HSA04012\_ERBB\_SIGNALING\_PATHWAY (c2) Genes involved in ErbB signaling pathway | 1.34352e-07 | 85/87 | 453 | 370.119 |
| HSA04510\_FOCAL\_ADHESION (c2) Genes involved in focal adhesion | 1.86942e-07 | 184/192 | 633 | 538.199 |
| SA\_REG\_CASCADE\_OF\_CYCLIN\_EXPR (c2) Expression of cyclins regulates progression through the cell cycle by activating cyclin-dependent kinases. | 2.12097e-07 | 12/13 | 87 | 53.779 |
| HSA04010\_MAPK\_SIGNALING\_PATHWAY (c2) Genes involved in MAPK signaling pathway | 2.25418e-07 | 229/252 | 780 | 668.246 |
| REGULATION\_OF\_APOPTOSIS (c5) Genes annotated by the GO term GO:0042981. Any process that modulates the occurrence or rate of cell death by apoptosis. | 3.10391e-07 | 326/337 | 786 | 675.541 |
| REGULATION\_OF\_PROGRAMMED\_CELL\_DEATH (c5) Genes annotated by the GO term GO:0043067. Any process that modulates the frequency, rate or extent of programmed cell death, cell death resulting from activation of endogenous cellular processes. | 3.13431e-07 | 327/338 | 786 | 675.599 |
| HSA04340\_HEDGEHOG\_SIGNALING\_PATHWAY (c2) Genes involved in Hedgehog signaling pathway | 4.61873e-07 | 53/57 | 119 | 78.195 |
| V$NRSF\_01 (c3) Genes with promoter regions [-2kb,2kb] around transcription start site containing the motif TTCAGCACCACGGACAGMGCC which matches annotation for REST: RE1-silencing transcription factor | 4.67359e-07 | 63/76 | 93 | 58.298 |
| IONOTROPIC\_GLUTAMATE\_RECEPTOR\_ACTIVITY (c5) Genes annotated by the GO term GO:0004970. Combining with glutamate to initiate a change in cell activity through the regulation of ion channels. | 5.21256e-07 | 9/10 | 25 | 10.001 |
| REGULATION\_OF\_DEVELOPMENTAL\_PROCESS (c5) Genes annotated by the GO term GO:0050793. Any process that modulates the frequency, rate or extent of development, the biological process whose specific outcome is the progression of a multicellular organism over time from an initial condition (e.g. a zygote, or a young adult) to a later condition (e.g. a multicellular animal or an aged adult). | 7.2164e-07 | 420/436 | 904 | 789.588 |
| S\_PHASE\_OF\_MITOTIC\_CELL\_CYCLE (c5) Genes annotated by the GO term GO:0000084. Progression through S phase, the part of the mitotic cell cycle during which DNA synthesis takes place. | 7.42554e-07 | 9/10 | 25 | 10.254 |
| CELLCYCLEPATHWAY (c2) Cyclins interact with cyclin-dependent kinases to form active kinase complexes that regulate progression through the cell cycle. | 9.8821e-07 | 22/23 | 121 | 81.805 |
| VOLTAGE\_GATED\_CHANNEL\_ACTIVITY (c5) Genes annotated by the GO term GO:0022832. Catalysis of the transmembrane transfer of a solute by a channel whose open state is dependent on the voltage across the membrane in which it is embedded. | 9.98857e-07 | 62/73 | 46 | 24.333 |
| ALZHEIMERS\_DISEASE\_DN (c2) Downregulated in correlation with overt Alzheimer's Disease, in the CA1 region of the hippocampus | 1.13681e-06 | 881/1043 | 709 | 605.138 |
| VOLTAGE\_GATED\_CATION\_CHANNEL\_ACTIVITY (c5) Genes annotated by the GO term GO:0022843. Catalysis of the transmembrane transfer of a cation by a voltage-gated channel. A cation is a positively charged ion. | 1.15797e-06 | 55/66 | 42 | 21.84 |
| GROWTH\_CONE (c5) Genes annotated by the GO term GO:0030426. The migrating motile tip of a growing nerve cell axon or dendrite. | 1.23764e-06 | 9/10 | 42 | 21.514 |
| MAPKKK\_CASCADE\_GO\_0000165 (c5) Genes annotated by the GO term GO:0000165. Cascade of at least three protein kinase activities culminating in the phosphorylation and activation of a MAP kinase. MAPKKK cascades lie downstream of numerous signaling pathways. | 1.54671e-06 | 101/102 | 222 | 169.922 |
| NEURON\_PROJECTION (c5) Genes annotated by the GO term GO:0043005. A prolongation or process extending from a nerve cell, e.g. an axon or dendrite. | 1.60654e-06 | 19/20 | 49 | 26.386 |
| REGULATION\_OF\_CELL\_CYCLE (c5) Genes annotated by the GO term GO:0051726. Any process that modulates the rate or extent of progression through the cell cycle. | 1.74122e-06 | 174/180 | 352 | 284.48 |
| P35ALZHEIMERSPATHWAY (c2) p35, a neuron-specific activator of cyclin-dependent kinase 5, is cleaved to p25 in Alzheimer's disease and promotoes hyperphosphorylated tau formation and apoptosis. | 1.8038e-06 | 10/11 | 58 | 33.793 |
| MAPKPATHWAY (c2) The mitogen-activated protein (MAP) kinase pathway is a common signaling mechanism and has four main sub-pathways: Erk, JNK/SAPK, p53, and ERK5. | 1.8133e-06 | 84/85 | 416 | 339.901 |
| VOLTAGE\_GATED\_CALCIUM\_CHANNEL\_ACTIVITY (c5) Genes annotated by the GO term GO:0005245. Catalysis of the transmembrane transfer of a calcium ion by a voltage-gated channel. | 1.84624e-06 | 13/18 | 12 | 3.659 |
| GNF2\_MAPT (c4) Neighborhood of MAPT | 1.87039e-06 | 32/38 | 48 | 25.261 |
| POSITIVE\_REGULATION\_OF\_DEVELOPMENTAL\_PROCESS (c5) Genes annotated by the GO term GO:0051094. Any process that activates or increases the rate or extent of development, the biological process whose specific outcome is the progression of an organism over time from an initial condition (e.g. a zygote, or a young adult) to a later condition (e.g. a multicellular animal or an aged adult). | 1.90872e-06 | 203/215 | 430 | 353.113 |
| CASPASEPATHWAY (c2) Caspases are cysteine proteases active in apoptosis; caspase-8 and 9 cleave and activate other caspases, while 3, 6, and 7 cleave cellular targets. | 2.12182e-06 | 20/21 | 87 | 55.361 |
| V$SREBP\_Q3 (c3) Genes with promoter regions [-2kb,2kb] around transcription start site containing motif VNNVTCACCCYA. Motif does not match any known transcription factor | 2.126e-06 | 162/186 | 216 | 162.562 |
| LIPOPROTEIN\_BINDING (c5) Genes annotated by the GO term GO:0008034. Interacting selectively with any conjugated, water-soluble protein in which the nonprotein moiety consists of a lipid or lipids. | 2.23863e-06 | 16/17 | 30 | 14.022 |
| module\_11 (c4) Genes in module\_11 | 2.29672e-06 | 474/533 | 484 | 402.686 |
| PROTEIN\_AMINO\_ACID\_AUTOPHOSPHORYLATION (c5) Genes annotated by the GO term GO:0046777. The phosphorylation by a protein of one or more of its own amino acid residues, or residues on an identical protein. | 2.38432e-06 | 27/29 | 78 | 50.88 |
| HSA04310\_WNT\_SIGNALING\_PATHWAY (c2) Genes involved in Wnt signaling pathway | 2.43568e-06 | 138/147 | 410 | 338.103 |
| GATTGGY\_V$NFY\_Q6\_01 (c3) Genes with promoter regions [-2kb,2kb] around transcription start site containing motif GATTGGY. Motif does not match any known transcription factor | 2.63448e-06 | 703/856 | 711 | 612.499 |
| AXON (c5) Genes annotated by the GO term GO:0030424. The long process of a neuron that conducts nerve impulses, usually away from the cell body to the terminals and varicosities, which are sites of storage and release of neurotransmitter. | 2.65246e-06 | 11/12 | 41 | 21.373 |
| AMYLOID\_PRECURSOR\_PROTEIN\_METABOLIC\_PROCESS (c5) Genes annotated by the GO term GO:0042982. The chemical reactions and pathways involving amyloid precursor protein (APP), the precursor of beta-amyloid, a glycoprotein associated with Alzheimer's disease. | 2.67421e-06 | 9/10 | 21 | 8.467 |
| ASTON\_DEPRESSION\_DN (c2) Genes downregulated in major depressive disorder (p < 0.05, fold change > 1.4, mean average difference > 150 in at least one of the groups, called present in greater than 20% of all samples) | 2.69879e-06 | 123/140 | 194 | 146.755 |
| SITE\_OF\_POLARIZED\_GROWTH (c5) Genes annotated by the GO term GO:0030427. Any part of a cell where non-isotropic growth takes place. | 3.24503e-06 | 10/11 | 43 | 22.74 |
| PROTEIN\_AUTOPROCESSING (c5) Genes annotated by the GO term GO:0016540. Processing which a protein carries out itself. This involves actions such as the autolytic removal of residues to generate the mature form of the protein. | 3.37216e-06 | 28/30 | 78 | 51.177 |
| CTGCAGY\_UNKNOWN (c3) Genes with promoter regions [-2kb,2kb] around transcription start site containing motif CTGCAGY. Motif does not match any known transcription factor | 3.57122e-06 | 469/568 | 532 | 450.461 |
| CHESLER\_D6MIT150\_NEURAL\_TARGETS\_GLOCUS (c2) Neurologically relevant downstream targets of an important regulatory locus. This locus is on chromosome 6 and near the D6Mit150 locus. | 4.06089e-06 | 7/8 | 37 | 19.876 |
| GLUTAMATE\_SIGNALING\_PATHWAY (c5) Genes annotated by the GO term GO:0007215. The series of molecular signals generated as a consequence of glutamate binding to a cell surface receptor. | 4.31275e-06 | 16/17 | 27 | 12.236 |
| PROTEIN\_MODIFICATION\_PROCESS (c5) Genes annotated by the GO term GO:0006464. The covalent alteration of one or more amino acids occurring in proteins, peptides and nascent polypeptides (co-translational, post-translational modifications). Includes the modification of charged tRNAs that are destined to occur in a protein (pre-translation modification). | 4.68215e-06 | 569/623 | 746 | 649.172 |
| HSA05222\_SMALL\_CELL\_LUNG\_CANCER (c2) Genes involved in small cell lung cancer | 4.85615e-06 | 82/86 | 369 | 305.63 |
| CCTGTGA,MIR-513 (c3) Targets of MicroRNA CCTGTGA,MIR-513 | 5.01501e-06 | 84/111 | 99 | 65.662 |
| FOSBPATHWAY (c2) FOSB gene expression and drug abuse | 5.06523e-06 | 4/5 | 24 | 10.658 |
| CELL\_CYCLE\_GO\_0007049 (c5) Genes annotated by the GO term GO:0007049. The progression of biochemical and morphological phases and events that occur in a cell during successive cell replication or nuclear replication events. Canonically, the cell cycle comprises the replication and segregation of genetic material followed by the division of the cell, but in endocycles or syncytial cells nuclear replication or nuclear division may not be followed by cell division. | 5.36817e-06 | 299/311 | 466 | 390.581 |
| REGULATION\_OF\_JNK\_CASCADE (c5) Genes annotated by the GO term GO:0046328. Any process that modulates the frequency, rate or extent of signal transduction mediated by the JNK cascade. | 5.4375e-06 | 11/12 | 23 | 9.683 |
| HSA05214\_GLIOMA (c2) Genes involved in glioma | 5.60981e-06 | 61/64 | 355 | 292.503 |
| CREBPATHWAY (c2) CREB is a transcription factor that binds to cAMP-responsive elements (CREs) to activate transcription in response to extracellular signaling. | 6.05687e-06 | 25/27 | 189 | 142.574 |
| V$IK3\_01 (c3) Genes with promoter regions [-2kb,2kb] around transcription start site containing motif TNYTGGGAATACC. Motif does not match any known transcription factor | 6.51426e-06 | 141/169 | 147 | 106.367 |
| BIOPOLYMER\_MODIFICATION (c5) Genes annotated by the GO term GO:0043412. The covalent alteration of one or more monomeric units in a polypeptide, polynucleotide, polysaccharide, or other biological polymer, resulting in a change in its properties. | 6.52732e-06 | 588/642 | 770 | 673.864 |
| HSA04742\_TASTE\_TRANSDUCTION (c2) Genes involved in taste transduction | 6.7227e-06 | 24/53 | 47 | 27.666 |
| ST\_GRANULE\_CELL\_SURVIVAL\_PATHWAY (c2) The survival and differentiation of granule cells in the brain is controlled by pro-growth PACAP and pro-apoptotic ceramides. | 6.96965e-06 | 25/26 | 176 | 134.236 |
| ABRAHAM\_MM\_VS\_AL\_UP (c2) Genes highly expressed in multiple myeloma (MM) versus immunoglobulin light chain amyloidosis (AL) in plasma cells. | 7.25637e-06 | 18/19 | 139 | 101.484 |
| NEUROLOGICAL\_SYSTEM\_PROCESS (c5) Genes annotated by the GO term GO:0050877. The processes pertaining to the functions of the nervous system of an organism. | 7.94963e-06 | 314/378 | 281 | 221.89 |
| NEURON\_DEVELOPMENT (c5) Genes annotated by the GO term GO:0048666. The process whose specific outcome is the progression of a neuron over time, from initial commitment of the cell to a specific fate, to the fully functional differentiated cell. | 9.2293e-06 | 59/61 | 94 | 62.715 |
| V$ZIC3\_01 (c3) Genes with promoter regions [-2kb,2kb] around transcription start site containing the motif NGGGKGGTC which matches annotation for ZIC3: Zic family member 3 heterotaxy 1 (odd-paired homolog, Drosophila) | 1.08313e-05 | 181/206 | 194 | 146.703 |
| HSA05050\_DENTATORUBROPALLIDOLUYSIAN\_ATROPHY (c2) Genes involved in dentatorubropallidoluysian atrophy (DRPLA) | 1.13873e-05 | 14/15 | 77 | 49.925 |
| TTGTTT\_V$FOXO4\_01 (c3) Genes with promoter regions [-2kb,2kb] around transcription start site containing the motif TTGTTT which matches annotation for MLLT7: myeloid/lymphoid or mixed-lineage leukemia (trithorax homolog, Drosophila); translocated to, 7 | 1.18304e-05 | 1248/1511 | 1143 | 1028.57 |
| MAP\_KINASE\_ACTIVITY (c5) Genes annotated by the GO term GO:0004707. Catalysis of the phosphorylation of proteins. Mitogen-activated protein kinase; a family of protein kinases that perform a crucial step in relaying signals from the plasma membrane to the nucleus. They are activated by a wide range of proliferation- or differentiation-inducing signals; activation is strong with agonists such as polypeptide growth factors and tumor-promoting phorbol esters, but weak (in most cell backgrounds) by stress stimuli. | 1.22848e-05 | 11/12 | 86 | 58.504 |
| CHEMICALPATHWAY (c2) DNA damage promotes Bid cleavage, which stimulates mitochondrial cytochrome c release and consequent caspase activation, resulting in apoptosis. | 1.26103e-05 | 20/21 | 193 | 150.113 |
| INTRACELLULAR\_SIGNALING\_CASCADE (c5) Genes annotated by the GO term GO:0007242. A series of reactions within the cell that occur as a result of a single trigger reaction or compound. | 1.30113e-05 | 628/658 | 1000 | 896.336 |
| PROTEIN\_PROCESSING (c5) Genes annotated by the GO term GO:0016485. The posttranslational modification of a protein, particularly secretory proteins and proteins targeted for membranes or specific cellular locations. | 1.48045e-05 | 45/47 | 95 | 66.018 |
| TRANSMEMBRANE\_RECEPTOR\_PROTEIN\_TYROSINE\_KINASE\_ACTIVITY (c5) Genes annotated by the GO term GO:0004714. Catalysis of the reaction: ATP + a protein-L-tyrosine = ADP + a protein-L-tyrosine phosphate, to initiate a change in cell activity. | 1.60543e-05 | 40/43 | 104 | 71.892 |
| BRENTANI\_CELL\_CYCLE (c2) Cancer related genes involved in the cell cycle | 1.62438e-05 | 78/79 | 232 | 184.426 |
| DEATHPATHWAY (c2) Death receptors such as Fas and DR3, 4, and 5 transduce pro-apoptotic signaling by oligomerizing to activate the caspase cascade. | 1.62841e-05 | 31/32 | 153 | 114.048 |
| V$E2F1\_Q3\_01 (c3) Genes with promoter regions [-2kb,2kb] around transcription start site containing the motif TTGGCGCGRAANNGNM which matches annotation for E2F1: E2F transcription factor 1 | 1.7665e-05 | 168/193 | 184 | 138.323 |
