# Supplementary material for: Looking at Cerebellar Malformations through Text-Mined Interactomes of Mice and Humans
Source: PLoS Comput Biol. 2009 Nov 6;5(11):e1000559. doi: 10.1371/journal.pcbi.1000559 (PMC2767227; doi:10.1371/journal.pcbi.1000559)
Supplement: Dataset S1 — All enrichment results. (0.20 MB ZIP) [file pcbi.1000559.s012.zip › enrichment_results/Table V. enrichment_whole-degeneration.html]

Complete Clustering results for network whole and phenotype degeneration (FDR <= 0.001)


# Complete Clustering results for network whole and phenotype degeneration (FDR <= 0.001)

| Set | p-Value | Gene Count | Interaction Count | Expected Interection Count |
| --- | --- | --- | --- | --- |
| STRUCTURE\_SPECIFIC\_DNA\_BINDING (c5) Genes annotated by the GO term GO:0043566. Interacting selectively with DNA of a specific structure or configuration e.g. triplex DNA binding or bent DNA binding. | 1e-20 | 53/56 | 43 | 13.89 |
| RESPONSE\_TO\_DNA\_DAMAGE\_STIMULUS (c5) Genes annotated by the GO term GO:0006974. A change in state or activity of a cell or an organism (in terms of movement, secretion, enzyme production, gene expression, etc.) as a result of a stimulus indicating damage to its DNA from environmental insults or errors during metabolism. | 1e-20 | 153/161 | 85 | 35.633 |
| DNA\_REPAIR (c5) Genes annotated by the GO term GO:0006281. The process of restoring DNA after damage. Genomes are subject to damage by chemical and physical agents in the environment (e.g. UV and ionizing radiations, chemical mutagens, fungal and bacterial toxins, etc.) and by free radicals or alkylating agents endogenously generated in metabolism. DNA is also damaged because of errors during its replication. A variety of different DNA repair pathways have been reported that include direct reversal, base excision repair, nucleotide excision repair, photoreactivation, bypass, double-strand break repair pathway, and mismatch repair pathway. | 1e-20 | 119/125 | 72 | 27.4 |
| SINGLE\_STRANDED\_DNA\_BINDING (c5) Genes annotated by the GO term GO:0003697. Interacting selectively with single-stranded DNA. | 1e-20 | 33/35 | 33 | 8.958 |
| BRENTANI\_REPAIR (c2) Cancer related genes involved in DNA repair | 3.33067e-16 | 35/36 | 34 | 9.712 |
| ATRBRCAPATHWAY (c2) BRCA1 and 2 block cell cycle progression in response to DNA damage and promote double-stranded break repair; mutations induce breast cancer susceptibility. | 1.25455e-14 | 20/21 | 35 | 11.577 |
| NUCLEOTIDE\_EXCISION\_REPAIR (c5) Genes annotated by the GO term GO:0006289. In nucleotide excision repair a small region of the strand surrounding the damage is removed from the DNA helix as an oligonucleotide. The small gap left in the DNA helix is filled in by the sequential action of DNA polymerase and DNA ligase. Nucleotide excision repair recognizes a wide range of substrates, including damage caused by UV irradiation (pyrimidine dimers and 6-4 photoproducts) and chemicals (intrastrand cross-links and bulky adducts). | 2.04281e-14 | 19/20 | 26 | 7.732 |
| RESPONSE\_TO\_ENDOGENOUS\_STIMULUS (c5) Genes annotated by the GO term GO:0009719. A change in state or activity of a cell or an organism (in terms of movement, secretion, enzyme production, gene expression, etc.) as a result of an endogenous stimulus. | 2.88658e-14 | 187/198 | 87 | 41.594 |
| GNF2\_SPRR1B (c4) Neighborhood of SPRR1B | 3.06422e-14 | 20/24 | 9 | 1.139 |
| DNA\_METABOLIC\_PROCESS (c5) Genes annotated by the GO term GO:0006259. The chemical reactions and pathways involving DNA, deoxyribonucleic acid, one of the two main types of nucleic acid, consisting of a long, unbranched macromolecule formed from one, or more commonly, two, strands of linked deoxyribonucleotides. | 1.05471e-13 | 243/256 | 117 | 62.161 |
| LYSOSOME\_ORGANIZATION\_AND\_BIOGENESIS (c5) Genes annotated by the GO term GO:0007040. A process that is carried out at the cellular level which results in the formation, arrangement of constituent parts, or disassembly of lysosomes. | 2.30482e-13 | 10/11 | 10 | 1.477 |
| DNA\_DAMAGE\_SIGNALING (c2) Genes involved in DNA damage signaling | 7.75158e-13 | 87/89 | 75 | 36.621 |
| ARYLSULFATASE\_ACTIVITY (c5) Genes annotated by the GO term GO:0004065. Catalysis of the reaction: a phenol sulfate + H2O = a phenol + SO4(2-) (sulfate). | 9.97868e-13 | 7/12 | 3 | 0.163 |
| VACUOLE\_ORGANIZATION\_AND\_BIOGENESIS (c5) Genes annotated by the GO term GO:0007033. A process that is carried out at the cellular level which results in the formation, arrangement of constituent parts, or disassembly of a vacuole. | 1.0727e-12 | 11/12 | 10 | 1.557 |
| NUCLEAR\_PORE (c5) Genes annotated by the GO term GO:0005643. Any of the numerous similar discrete openings in the nuclear envelope of a eukaryotic cell, where the inner and outer nuclear membranes are joined. | 8.26994e-12 | 30/31 | 18 | 4.199 |
| PORE\_COMPLEX (c5) Genes annotated by the GO term GO:0046930. Any small opening in a membrane that allows the passage of gases and/or liquids. | 2.96274e-11 | 35/36 | 22 | 5.885 |
| SULFURIC\_ESTER\_HYDROLASE\_ACTIVITY (c5) Genes annotated by the GO term GO:0008484. Catalysis of the reaction: RSO-R' + H2O = RSOOH + R'H. This reaction is the hydrolysis of any sulfuric ester bond, any ester formed from sulfuric acid, O=SO(OH)2. | 1.6085e-10 | 9/16 | 5 | 0.498 |
| DNA\_INTEGRITY\_CHECKPOINT (c5) Genes annotated by the GO term GO:0031570. Any cell cycle checkpoint that delays or arrests cell cycle progression in response to changes in DNA structure. | 4.47008e-10 | 21/23 | 18 | 4.944 |
| DNA\_DAMAGE\_CHECKPOINT (c5) Genes annotated by the GO term GO:0000077. A signal transduction pathway, induced by DNA damage, that blocks cell cycle progression (in G1, G2 or metaphase) or slows the rate at which S phase proceeds. | 5.11441e-10 | 17/19 | 16 | 4.124 |
| APPEL\_IMATINIB\_UP (c2) Genes up-regulated by imatinib during dendritic cell differentiation | 6.58119e-10 | 28/31 | 19 | 5.389 |
| NUCLEAR\_MEMBRANE (c5) Genes annotated by the GO term GO:0031965. Either of the lipid bilayers that surround the nucleus and form the nuclear envelope; excludes the intermembrane space. | 1.08183e-09 | 46/50 | 26 | 9.108 |
| DOUBLE\_STRAND\_BREAK\_REPAIR (c5) Genes annotated by the GO term GO:0006302. The repair of double-strand breaks in DNA via homologous and nonhomologous mechanisms to reform a continuous DNA helix. | 1.47016e-09 | 22/23 | 20 | 5.948 |
| REGULATION\_OF\_PH (c5) Genes annotated by the GO term GO:0006885. Any process that modulates the internal pH of an organism, part of an organism or a cell, measured by the concentration of the hydrogen ion. | 8.03194e-09 | 11/13 | 13 | 3.556 |
| MONOVALENT\_INORGANIC\_CATION\_HOMEOSTASIS (c5) Genes annotated by the GO term GO:0055067. The regulation of the levels, transport, and metabolism of monovalent inorganic cations. | 9.49685e-09 | 12/14 | 13 | 3.566 |
| DOUBLE\_STRANDED\_DNA\_BINDING (c5) Genes annotated by the GO term GO:0003690. Interacting selectively with double-stranded DNA. | 1.64263e-08 | 30/32 | 27 | 10.813 |
| REGULATION\_OF\_CELLULAR\_PH (c5) Genes annotated by the GO term GO:0030641. The regulation of the levels, transport, and metabolism of hydrogen ions (protons) within a cell or between a cell and its external environment. | 2.38883e-08 | 8/10 | 12 | 3.196 |
| CELLULAR\_MONOVALENT\_INORGANIC\_CATION\_HOMEOSTASIS (c5) Genes annotated by the GO term GO:0030004. The regulation of the levels, transport, and metabolism of monovalent inorganic cations within a cell or between a cell and its external environment. | 2.74874e-08 | 9/11 | 12 | 3.206 |
| DNA\_DAMAGE\_RESPONSE\_\_SIGNAL\_TRANSDUCTION (c5) Genes annotated by the GO term GO:0042770. A cascade of processes induced by the detection of DNA damage within a cell. | 3.95838e-08 | 31/34 | 31 | 13.284 |
| chr5p11 (c1) Genes in cytogenetic band chr5p11 | 7.69392e-08 | 0/1 | 1 | 0.035 |
| DNA\_REPLICATION (c5) Genes annotated by the GO term GO:0006260. The process whereby new strands of DNA are synthesized. The template for replication can either be an existing DNA molecule or RNA. | 8.24899e-08 | 95/101 | 44 | 20.744 |
| NUCLEAR\_MEMBRANE\_PART (c5) Genes annotated by the GO term GO:0044453. Any constituent part of the nuclear membrane, the envelope that surrounds the nucleus of eukaryotic cells. | 8.53966e-08 | 40/42 | 19 | 6.018 |
| NUCLEAR\_ENVELOPE (c5) Genes annotated by the GO term GO:0005635. The double lipid bilayer enclosing the nucleus and separating its contents from the rest of the cytoplasm; includes the intermembrane space, a gap of width 20-40 nm (also called the perinuclear space). | 9.25513e-08 | 68/73 | 33 | 14.142 |
| DNA\_CATABOLIC\_PROCESS (c5) Genes annotated by the GO term GO:0006308. The chemical reactions and pathways resulting in the breakdown of DNA, deoxyribonucleic acid, one of the two main types of nucleic acid, consisting of a long unbranched macromolecule formed from one or two strands of linked deoxyribonucleotides, the 3'-phosphate group of each constituent deoxyribonucleotide being joined in 3',5'-phosphodiester linkage to the 5'-hydroxyl group of the deoxyribose moiety of the next one. | 1.03574e-07 | 22/23 | 18 | 5.608 |
| NUCLEAR\_PART (c5) Genes annotated by the GO term GO:0044428. Any constituent part of the nucleus, a membrane-bounded organelle of eukaryotic cells in which chromosomes are housed and replicated. | 1.30069e-07 | 527/572 | 139 | 91.738 |
| CELL\_CYCLE\_CHECKPOINT (c2) A point in the eukaryotic cell cycle where progress through the cycle can be halted until conditions are suitable for the cell to proceed to the next stage. | 1.54673e-07 | 23/24 | 31 | 13.537 |
| ORGANELLE\_ENVELOPE (c5) Genes annotated by the GO term GO:0031967. A double membrane structure enclosing an organelle, including two lipid bilayers and the region between them. In some cases, an organelle envelope may have more than two membranes. | 2.29677e-07 | 137/168 | 45 | 22.133 |
| ENVELOPE (c5) Genes annotated by the GO term GO:0031975. A multilayered structure surrounding all or part of a cell; encompasses one or more lipid bilayers, and may include a cell wall layer, also includes the space between layers. | 2.29677e-07 | 137/168 | 45 | 22.133 |
| MEIOTIC\_RECOMBINATION (c5) Genes annotated by the GO term GO:0007131. The cell cycle process whereby double strand breaks are formed and repaired through a double Holliday junction intermediate. This results in the equal exchange of genetic material between non-sister chromatids in a pair of homologous chromosomes. These reciprocal recombinant products ensure the proper segregation of homologous chromosomes during meiosis I and create genetic diversity. | 2.62885e-07 | 15/17 | 12 | 3.209 |
| NUCLEUS (c5) Genes annotated by the GO term GO:0005634. A membrane-bounded organelle of eukaryotic cells in which chromosomes are housed and replicated. In most cells, the nucleus contains all of the cell's chromosomes except the organellar chromosomes, and is the site of RNA synthesis and processing. In some species, or in specialized cell types, RNA metabolism or DNA replication may be absent. | 3.63062e-07 | 1296/1417 | 296 | 232.128 |
| ENDODEOXYRIBONUCLEASE\_ACTIVITY (c5) Genes annotated by the GO term GO:0004520. Catalysis of the hydrolysis of ester linkages within deoxyribonucleic acid by creating internal breaks. | 4.51359e-07 | 10/11 | 7 | 1.33 |
| DNA\_DEPENDENT\_DNA\_REPLICATION (c5) Genes annotated by the GO term GO:0006261. The process whereby new strands of DNA are synthesized, using parental DNA as a template for the DNA-dependent DNA polymerases that synthesize the new strands. | 4.60099e-07 | 51/56 | 26 | 10.264 |
| DAMAGED\_DNA\_BINDING (c5) Genes annotated by the GO term GO:0003684. Interacting selectively with damaged DNA. | 5.67589e-07 | 18/21 | 9 | 1.926 |
| APOPTOSIS\_GENMAPP (c2) | 6.29768e-07 | 40/41 | 80 | 50.529 |
| MITOCHONDRIAPATHWAY (c2) Pro-apoptotic signaling induces mitochondria to release cytochrome c, which stimulates Apaf-1 to activate caspase 9. | 7.13488e-07 | 18/19 | 31 | 14.258 |
| DEOXYRIBONUCLEASE\_ACTIVITY (c5) Genes annotated by the GO term GO:0004536. Catalysis of the hydrolysis of ester linkages within deoxyribonucleic acid. | 7.84378e-07 | 19/22 | 9 | 2.093 |
| module\_283 (c4) Genes in module\_283 | 8.48484e-07 | 6/7 | 6 | 1.072 |
| MEIOSIS\_I (c5) Genes annotated by the GO term GO:0007127. Progression through the first phase of meiosis, in which cells divide and homologous chromosomes are paired and segregated from each other, producing two daughter cells. | 9.01935e-07 | 17/19 | 12 | 3.361 |
| ORGANELLE\_PART (c5) Genes annotated by the GO term GO:0044422. Any constituent part of an organelle, an organized structure of distinctive morphology and function. Includes constituent parts of the nucleus, mitochondria, plastids, vacuoles, vesicles, ribosomes and the cytoskeleton, but excludes the plasma membrane. | 1.55199e-06 | 1034/1182 | 208 | 154.305 |
| INTRACELLULAR\_ORGANELLE\_PART (c5) Genes annotated by the GO term GO:0044446. A constituent part of an intracellular organelle, an organized structure of distinctive morphology and function, occurring within the cell. Includes constituent parts of the nucleus, mitochondria, plastids, vacuoles, vesicles, ribosomes and the cytoskeleton but excludes the plasma membrane. | 1.56504e-06 | 1029/1177 | 207 | 153.314 |
| H2O2\_CSBRESCUED\_C2\_UP (c2) Upregulated by H2O2 in CSB-rescued fibroblasts (Table 1, cluster 2) | 1.58717e-06 | 7/8 | 5 | 0.865 |
| SPHINGOID\_METABOLIC\_PROCESS (c5) Genes annotated by the GO term GO:0046519. The chemical reactions and pathways involving sphingoids, any of a class of compounds comprising sphinganine and its homologues and stereoisomers, and derivatives of these compounds. | 1.59597e-06 | 10/12 | 4 | 0.562 |
| CELL\_CYCLE\_CHECKPOINT\_GO\_0000075 (c5) Genes annotated by the GO term GO:0000075. A point in the eukaryotic cell cycle where progress through the cycle can be halted until conditions are suitable for the cell to proceed to the next stage. | 1.79379e-06 | 45/47 | 31 | 13.81 |
| STEMCELL\_EMBRYONIC\_UP (c2) Enriched in mouse embryonic stem cells, compared to differentiated brain and bone marrow cells | 2.41807e-06 | 1029/1239 | 189 | 139.445 |
| NEGATIVE\_REGULATION\_OF\_DNA\_METABOLIC\_PROCESS (c5) Genes annotated by the GO term GO:0051053. Any process that stops, prevents or reduces the frequency, rate or extent of the chemical reactions and pathways involving DNA. | 2.47486e-06 | 16/18 | 10 | 2.64 |
| GLYCOSPHINGOLIPID\_METABOLISM (c2) | 2.72645e-06 | 21/22 | 7 | 1.485 |
| PROTEIN\_HOMOOLIGOMERIZATION (c5) Genes annotated by the GO term GO:0051260. The process of creating protein oligomers, compounds composed of a small number, usually between three and ten, of identical component monomers. Oligomers may be formed by the polymerization of a number of monomers or the depolymerization of a large protein polymer. | 2.92372e-06 | 21/22 | 12 | 3.674 |
| CERAMIDE\_METABOLIC\_PROCESS (c5) Genes annotated by the GO term GO:0006672. The chemical reactions and pathways involving ceramides, any N-acylated sphingoid. | 3.07949e-06 | 9/11 | 3 | 0.339 |
| HSA00600\_SPHINGOLIPID\_METABOLISM (c2) Genes involved in sphingolipid metabolism | 3.19244e-06 | 30/38 | 8 | 1.885 |
| PLK3PATHWAY (c2) Active Plk3 phosphorylates CDC25c, blocking the G2/M transition, and phosphorylates p53 to induce apoptosis. | 3.75187e-06 | 6/7 | 19 | 8.016 |
| HSA01510\_NEURODEGENERATIVE\_DISEASES (c2) Genes involved in neurodegenerative diseases | 4.48278e-06 | 37/38 | 51 | 29.439 |
| NUCLEAR\_CHROMOSOME (c5) Genes annotated by the GO term GO:0000228. A chromosome found in the nucleus of a eukaryotic cell. | 5.90509e-06 | 45/53 | 25 | 11.446 |
| OLDAGE\_DN (c2) Downregulated in fibroblasts from old individuals, compared to young | 6.88825e-06 | 44/45 | 23 | 9.799 |
| RESPONSE\_TO\_IONIZING\_RADIATION (c5) Genes annotated by the GO term GO:0010212. A change in state or activity of a cell or an organism (in terms of movement, secretion, enzyme production, gene expression, etc.) as a result of a ionizing radiation stimulus. Ionizing radiation is radiation with sufficient energy to remove electrons from atoms and may arise from spontaneous decay of unstable isotopes, resulting in alpha and beta particles and gamma rays. Ionizing radiation also includes X-rays. | 7.51764e-06 | 7/9 | 9 | 2.318 |
