# Supplementary material for: Looking at Cerebellar Malformations through Text-Mined Interactomes of Mice and Humans
Source: PLoS Comput Biol. 2009 Nov 6;5(11):e1000559. doi: 10.1371/journal.pcbi.1000559 (PMC2767227; doi:10.1371/journal.pcbi.1000559)
Supplement: Dataset S1 — All enrichment results. (0.20 MB ZIP) [file pcbi.1000559.s012.zip › enrichment_results/Table W. enrichment_whole-development.html]

Complete Clustering results for network whole and phenotype development (FDR <= 0.001)


# Complete Clustering results for network whole and phenotype development (FDR <= 0.001)

| Set | p-Value | Gene Count | Interaction Count | Expected Interection Count |
| --- | --- | --- | --- | --- |
| HSA04340\_HEDGEHOG\_SIGNALING\_PATHWAY (c2) Genes involved in Hedgehog signaling pathway | 1e-20 | 53/57 | 132 | 44.863 |
| REGULATION\_OF\_CELL\_CYCLE (c5) Genes annotated by the GO term GO:0051726. Any process that modulates the rate or extent of progression through the cell cycle. | 1e-20 | 174/180 | 246 | 157.819 |
| HSA05217\_BASAL\_CELL\_CARCINOMA (c2) Genes involved in basal cell carcinoma | 1e-20 | 53/55 | 118 | 54.607 |
| CELL\_CYCLE\_GO\_0007049 (c5) Genes annotated by the GO term GO:0007049. The progression of biochemical and morphological phases and events that occur in a cell during successive cell replication or nuclear replication events. Canonically, the cell cycle comprises the replication and segregation of genetic material followed by the division of the cell, but in endocycles or syncytial cells nuclear replication or nuclear division may not be followed by cell division. | 1e-20 | 299/311 | 319 | 217.613 |
| NERVOUS\_SYSTEM\_DEVELOPMENT (c5) Genes annotated by the GO term GO:0007399. The process whose specific outcome is the progression of nervous tissue over time, from its formation to its mature state. | 1.33227e-15 | 331/382 | 269 | 175.928 |
| CELL\_CYCLE (c2) The progression of biochemical and morphological events that occur during nuclear or cellular replication. | 1.9984e-15 | 73/76 | 195 | 119.858 |
| CELLCYCLEPATHWAY (c2) Cyclins interact with cyclin-dependent kinases to form active kinase complexes that regulate progression through the cell cycle. | 6.99441e-15 | 22/23 | 91 | 44.481 |
| MULTICELLULAR\_ORGANISMAL\_DEVELOPMENT (c5) Genes annotated by the GO term GO:0007275. The biological process whose specific outcome is the progression of an organism over time from an initial condition (e.g. a zygote or a young adult) to a later condition (e.g. a multicellular animal or an aged adult). | 7.10543e-15 | 926/1045 | 761 | 601.193 |
| HSA04115\_P53\_SIGNALING\_PATHWAY (c2) Genes involved in p53 signaling pathway | 1.23235e-14 | 64/66 | 168 | 101.183 |
| BRAIN\_DEVELOPMENT (c5) Genes annotated by the GO term GO:0007420. The process whose specific outcome is the progression of the brain over time, from its formation to the mature structure. The brain is one of the two components of the central nervous system and is the center of thought and emotion. It is responsible for the coordination and control of bodily activities and the interpretation of information from the senses (sight, hearing, smell, etc.). | 1.4988e-14 | 44/51 | 50 | 18.801 |
| CELL\_CYCLE\_KEGG (c2) | 5.973e-14 | 80/84 | 204 | 130.751 |
| SYSTEM\_DEVELOPMENT (c5) Genes annotated by the GO term GO:0048731. The process whose specific outcome is the progression of an organismal system over time, from its formation to the mature structure. A system is a regularly interacting or interdependent group of organs or tissues that work together to carry out a given biological process. | 6.27276e-14 | 777/858 | 663 | 517.197 |
| SHHPATHWAY (c2) Sonic hedgehog (Shh) signaling in the developing CNS induces neuronal proliferation via interaction with the patched (Ptc-1) and smoothened receptors. | 6.68354e-14 | 12/14 | 35 | 11.792 |
| BRENTANI\_CELL\_CYCLE (c2) Cancer related genes involved in the cell cycle | 7.61613e-14 | 78/79 | 165 | 100.207 |
| G1\_TO\_S\_CELL\_CYCLE\_REACTOME (c2) | 8.64864e-14 | 65/66 | 151 | 88.864 |
| ANATOMICAL\_STRUCTURE\_DEVELOPMENT (c5) Genes annotated by the GO term GO:0048856. The biological process whose specific outcome is the progression of an anatomical structure from an initial condition to its mature state. This process begins with the formation of the structure and ends with the mature structure, whatever form that may be including its natural destruction. An anatomical structure is any biological entity that occupies space and is distinguished from its surroundings. Anatomical structures can be macroscopic such as a carpel, or microscopic such as an acrosome. | 2.46692e-13 | 908/1012 | 718 | 571.095 |
| EMBRYONIC\_MORPHOGENESIS (c5) Genes annotated by the GO term GO:0048598. The process by which anatomical structures are generated and organized during the embryonic phase. Morphogenesis pertains to the creation of form. The embryonic phase begins with zygote formation. The end of the embryonic phase is organism-specific. For example, it would be at birth for mammals, larval hatching for insects and seed dormancy in plants. | 1.34481e-12 | 14/17 | 30 | 9.789 |
| ANATOMICAL\_STRUCTURE\_MORPHOGENESIS (c5) Genes annotated by the GO term GO:0009653. The process by which anatomical structures are generated and organized. Morphogenesis pertains to the creation of form. | 1.50857e-12 | 345/379 | 333 | 239.22 |
| chr6p11 (c1) Genes in cytogenetic band chr6p11 | 2.79254e-12 | 3/6 | 3 | 0.177 |
| GLAND\_DEVELOPMENT (c5) Genes annotated by the GO term GO:0048732. The process whose specific outcome is the progression of a gland over time, from its formation to the mature structure. A gland is an organ specialised for secretion. | 2.82996e-12 | 12/13 | 40 | 15.218 |
| G1PATHWAY (c2) CDK4/6-cyclin D and CDK2-cyclin E phosphorylate Rb, which allows the transcription of genes needed for the G1/S cell cycle transition. | 2.95552e-12 | 25/26 | 125 | 73.573 |
| HSA04110\_CELL\_CYCLE (c2) Genes involved in cell cycle | 3.45923e-12 | 110/112 | 250 | 171.479 |
| DNA\_BINDING (c5) Genes annotated by the GO term GO:0003677. Interacting selectively with DNA (deoxyribonucleic acid). | 1.54963e-11 | 540/600 | 494 | 384.447 |
| SA\_REG\_CASCADE\_OF\_CYCLIN\_EXPR (c2) Expression of cyclins regulates progression through the cell cycle by activating cyclin-dependent kinases. | 2.00308e-11 | 12/13 | 61 | 29.776 |
| NEURITE\_DEVELOPMENT (c5) Genes annotated by the GO term GO:0031175. The process whose specific outcome is the progression of the neurite over time, from its formation to the mature structure. The neurite is any process extending from a neural cell, such as axons or dendrites. | 2.22994e-11 | 51/53 | 65 | 31.372 |
| V$LEF1\_Q6 (c3) Genes with promoter regions [-2kb,2kb] around transcription start site containing the motif SWWCAAAGGG which matches annotation for LEF1: lymphoid enhancer-binding factor 1  TCF1: transcription factor 1, hepatic; LF-B1, hepatic nuclear factor (HNF1), albumin proximal factor | 2.86057e-11 | 174/212 | 102 | 55.226 |
| TRANSCRIPTION\_FACTOR\_ACTIVITY | 5.23135e-11 | 316/353 | 367 | 276.376 |
| module\_98 (c4) Genes in module\_98 | 7.61413e-11 | 369/391 | 367 | 275.091 |
| GGGAGGRR\_V$MAZ\_Q6 (c3) Genes with promoter regions [-2kb,2kb] around transcription start site containing the motif GGGAGGRR which matches annotation for MAZ: MYC-associated zinc finger protein (purine-binding transcription factor) | 8.3761e-11 | 1470/1733 | 884 | 747.79 |
| module\_220 (c4) Genes in module\_220 | 3.00728e-10 | 316/329 | 318 | 234.253 |
| NUCLEUS (c5) Genes annotated by the GO term GO:0005634. A membrane-bounded organelle of eukaryotic cells in which chromosomes are housed and replicated. In most cells, the nucleus contains all of the cell's chromosomes except the organellar chromosomes, and is the site of RNA synthesis and processing. In some species, or in specialized cell types, RNA metabolism or DNA replication may be absent. | 4.33139e-10 | 1296/1417 | 840 | 706.973 |
| GATTGGY\_V$NFY\_Q6\_01 (c3) Genes with promoter regions [-2kb,2kb] around transcription start site containing motif GATTGGY. Motif does not match any known transcription factor | 6.34588e-10 | 703/856 | 446 | 349.088 |
| CENTRAL\_NERVOUS\_SYSTEM\_DEVELOPMENT (c5) Genes annotated by the GO term GO:0007417. The process whose specific outcome is the progression of the central nervous system over time, from its formation to the mature structure. The central nervous system is the core nervous system that serves an integrating and coordinating function. In vertebrates it consists of the brain, spinal cord and spinal nerves. In those invertebrates with a central nervous system it typically consists of a brain, cerebral ganglia and a nerve cord. | 8.14328e-10 | 109/123 | 87 | 47.431 |
| HSA05218\_MELANOMA (c2) Genes involved in melanoma | 9.03614e-10 | 65/71 | 221 | 158.301 |
| module\_197 (c4) Genes in module\_197 | 9.30689e-10 | 156/173 | 213 | 149.582 |
| ORGAN\_MORPHOGENESIS (c5) Genes annotated by the GO term GO:0009887. Morphogenesis of an organ. An organ is defined as a tissue or set of tissues that work together to perform a specific function or functions. Morphogenesis is the process by which anatomical structures are generated and organized. Organs are commonly observed as visibly distinct structures, but may also exist as loosely associated clusters of cells that work together to perform a specific function or functions. | 2.39785e-09 | 136/145 | 187 | 127.687 |
| AXONOGENESIS (c5) Genes annotated by the GO term GO:0007409. Generation of a long process of a neuron, that carries efferent (outgoing) action potentials from the cell body towards target cells. | 2.68802e-09 | 41/43 | 49 | 23.19 |
| V$AP2\_Q3 (c3) Genes with promoter regions [-2kb,2kb] around transcription start site containing the motif GSCCSCRGGCNRNRNN which matches annotation for GTF3A: general transcription factor IIIA | 4.00265e-09 | 167/195 | 122 | 76.879 |
| P27PATHWAY (c2) p27 blocks the G1/S transition by inhibiting the checkpoint kinase cdk2/cyclin E and is inhibited by cdk2-mediated ubiquitination. | 4.75483e-09 | 11/12 | 43 | 20.646 |
| module\_198 (c4) Genes in module\_198 | 9.09353e-09 | 285/301 | 302 | 227.497 |
| NEURON\_DEVELOPMENT (c5) Genes annotated by the GO term GO:0048666. The process whose specific outcome is the progression of a neuron over time, from initial commitment of the cell to a specific fate, to the fully functional differentiated cell. | 1.00293e-08 | 59/61 | 68 | 36.685 |
| NUCLEOBASE\_\_NUCLEOSIDE\_\_NUCLEOTIDE\_AND\_NUCLEIC\_ACID\_METABOLIC\_PROCESS (c5) Genes annotated by the GO term GO:0006139. The chemical reactions and pathways involving nucleobases, nucleosides, nucleotides and nucleic acids. | 1.56344e-08 | 1151/1234 | 815 | 695.862 |
| TRANSCRIPTION (c5) Genes annotated by the GO term GO:0006350. The synthesis of either RNA on a template of DNA or DNA on a template of RNA. | 1.58945e-08 | 713/750 | 635 | 527.44 |
| NEGATIVE\_REGULATION\_OF\_CELL\_CYCLE (c5) Genes annotated by the GO term GO:0045786. Any process that stops, prevents or reduces the rate or extent of progression through the cell cycle. | 1.90379e-08 | 74/77 | 126 | 83.026 |
| AXON\_GUIDANCE (c5) Genes annotated by the GO term GO:0007411. The process by which the migration of an axon growth cone is directed to a specific target site in response to a combination of attractive and repulsive cues. | 1.95981e-08 | 20/22 | 29 | 12.019 |
| CELL\_CYCLE\_CHECKPOINT\_GO\_0000075 (c5) Genes annotated by the GO term GO:0000075. A point in the eukaryotic cell cycle where progress through the cycle can be halted until conditions are suitable for the cell to proceed to the next stage. | 2.68586e-08 | 45/47 | 73 | 40.959 |
| CELLULAR\_MORPHOGENESIS\_DURING\_DIFFERENTIATION (c5) Genes annotated by the GO term GO:0000904. The change in form (cell shape and size) that occurs when relatively unspecialized cells, e.g. embryonic or regenerative cells, acquire specialized structural and/or functional features that characterize the cells, tissues, or organs of the mature organism or some other relatively stable phase of the organism's life history. | 3.53008e-08 | 46/49 | 50 | 25.034 |
| CELL\_FATE\_COMMITMENT (c5) Genes annotated by the GO term GO:0045165. The commitment of cells to specific cell fates and their capacity to differentiate into particular kinds of cells. Positional information is established through protein signals that emanate from a localized source within a cell (the initial one-cell zygote) or within a developmental field. | 3.67431e-08 | 11/13 | 33 | 14.471 |
| NEUROGENESIS (c5) Genes annotated by the GO term GO:0022008. Generation of cells within the nervous system. | 3.78867e-08 | 89/93 | 88 | 52.388 |
| GENERATION\_OF\_NEURONS (c5) Genes annotated by the GO term GO:0048699. The process by which nerve cells are generated. This includes the production of neuroblasts and their differentiation into neurons. | 4.26761e-08 | 79/83 | 82 | 47.822 |
| GROWTH\_CONE (c5) Genes annotated by the GO term GO:0030426. The migrating motile tip of a growing nerve cell axon or dendrite. | 4.85381e-08 | 9/10 | 28 | 11.714 |
| V$CHX10\_01 (c3) Genes with promoter regions [-2kb,2kb] around transcription start site containing the motif NNNTAATTAGCNNN which matches annotation for VSX1: visual system homeobox 1 homolog, CHX10-like (zebrafish) | 4.9498e-08 | 149/184 | 115 | 74.058 |
| AAAYWAACM\_V$HFH4\_01 (c3) Genes with promoter regions [-2kb,2kb] around transcription start site containing the motif AAAYWAACM which matches annotation for FOXJ1: forkhead box J1 | 5.31297e-08 | 163/195 | 115 | 74.082 |
| WCAANNNYCAG\_UNKNOWN (c3) Genes with promoter regions [-2kb,2kb] around transcription start site containing motif WCAANNNYCAG. Motif does not match any known transcription factor | 5.50932e-08 | 143/184 | 114 | 73.907 |
| NEURON\_DIFFERENTIATION (c5) Genes annotated by the GO term GO:0030182. The process whereby a relatively unspecialized cell acquires specialized features of a neuron. | 6.00962e-08 | 72/76 | 76 | 43.605 |
| module\_57 (c4) Genes in module\_57 | 6.06475e-08 | 54/56 | 133 | 87.935 |
| RNA\_BIOSYNTHETIC\_PROCESS (c5) Genes annotated by the GO term GO:0032774. The chemical reactions and pathways resulting in the formation of RNA, ribonucleic acid, one of the two main type of nucleic acid, consisting of a long, unbranched macromolecule formed from ribonucleotides joined in 3',5'-phosphodiester linkage. Includes polymerization of ribonucleotide monomers. | 7.08582e-08 | 604/636 | 534 | 441.455 |
| V$HNF6\_Q6 (c3) Genes with promoter regions [-2kb,2kb] around transcription start site containing the motif HWAAATCAATAW which matches annotation for ONECUT1: one cut domain, family member 1 | 7.82043e-08 | 150/183 | 144 | 99.298 |
| ORGAN\_DEVELOPMENT (c5) Genes annotated by the GO term GO:0048513. Development of a tissue or tissues that work together to perform a specific function or functions. Development pertains to the process whose specific outcome is the progression of a structure over time, from its formation to the mature structure. Organs are commonly observed as visibly distinct structures, but may also exist as loosely associated clusters of cells that work together to perform a specific function or functions. | 8.02162e-08 | 530/569 | 485 | 395.518 |
| REGULATION\_OF\_METABOLIC\_PROCESS (c5) Genes annotated by the GO term GO:0019222. Any process that modulates the frequency, rate or extent of the chemical reactions and pathways within a cell or an organism. | 8.70865e-08 | 751/794 | 702 | 596.324 |
| REGULATION\_OF\_CELLULAR\_METABOLIC\_PROCESS (c5) Genes annotated by the GO term GO:0031323. Any process that modulates the frequency, rate or extent of the chemical reactions and pathways by which individual cells transform chemical substances. | 8.89302e-08 | 739/782 | 687 | 583.697 |
| YTAATTAA\_V$LHX3\_01 (c3) Genes with promoter regions [-2kb,2kb] around transcription start site containing the motif YTAATTAA which matches annotation for LHX3: LIM homeobox 3 | 8.91459e-08 | 119/145 | 64 | 35.194 |
| PROLIFERATION\_GENES (c2) Proliferation related genes | 9.28301e-08 | 343/359 | 415 | 336.443 |
| TRANSCRIPTION\_\_DNA\_DEPENDENT (c5) Genes annotated by the GO term GO:0006351. The synthesis of RNA on a template of DNA. | 9.57302e-08 | 602/634 | 533 | 441.406 |
| PATTERN\_SPECIFICATION\_PROCESS (c5) Genes annotated by the GO term GO:0007389. The developmental processes that result in the creation of defined areas or spaces within an organism to which cells respond and eventually are instructed to differentiate. | 9.96708e-08 | 27/31 | 51 | 26.49 |
| CAGGTA\_V$AREB6\_01 (c3) Genes with promoter regions [-2kb,2kb] around transcription start site containing the motif CAGGTA which matches annotation for TCF8: transcription factor 8 (represses interleukin 2 expression) | 1.53668e-07 | 471/582 | 304 | 235.269 |
| REGULATION\_OF\_PHOSPHORYLATION (c5) Genes annotated by the GO term GO:0042325. Any process that modulates the frequency, rate or extent of addition of phosphate groups into a molecule. | 1.65756e-07 | 48/49 | 120 | 81.265 |
| V$FAC1\_01 (c3) Genes with promoter regions [-2kb,2kb] around transcription start site containing the motif NNNCAMAACACRNA which matches annotation for FALZ: fetal Alzheimer antigen | 1.75547e-07 | 143/158 | 108 | 69.342 |
| V$T3R\_Q6 (c3) Genes with promoter regions [-2kb,2kb] around transcription start site containing motif MNTGWCCTN. Motif does not match any known transcription factor | 2.03024e-07 | 160/194 | 91 | 55.708 |
| V$COMP1\_01 (c3) Genes with promoter regions [-2kb,2kb] around transcription start site containing the motif NVTNWTGATTGACNACAAVARRBN which matches annotation for MYOG: myogenin (myogenic factor 4) | 2.16152e-07 | 86/94 | 64 | 36.214 |
| V$SP3\_Q3 (c3) Genes with promoter regions [-2kb,2kb] around transcription start site containing the motif ASMCTTGGGSRGGG which matches annotation for SP3: Sp3 transcription factor | 2.29247e-07 | 154/187 | 120 | 79.709 |
| SHEPARD\_CRASH\_AND\_BURN\_MUT\_VS\_WT\_DN (c2) Genes upregulated in zebra fish wild type compared to the crash and burn mutant | 2.49226e-07 | 133/150 | 89 | 53.837 |
| chr20p11 (c1) Genes in cytogenetic band chr20p11 | 2.99013e-07 | 26/68 | 15 | 4.75 |
| SITE\_OF\_POLARIZED\_GROWTH (c5) Genes annotated by the GO term GO:0030427. Any part of a cell where non-isotropic growth takes place. | 3.12133e-07 | 10/11 | 28 | 12.352 |
| RORIE\_ES\_PNET\_DN (c2) The 30 genes showing the greatest decrease in expression in NBa Ews/Fli-1 infectants | 3.40529e-07 | 24/26 | 27 | 11.015 |
| RUIZ\_TENASCIN\_TARGETS (c2) Tenascin-C target genes | 3.41551e-07 | 76/77 | 99 | 62.355 |
| CORTEX\_ENRICHMENT\_LATE\_UP (c2) Up-regulated in the cortex of mice that are exposed to an enriched environmental habitat for 2 or 14 days | 3.89645e-07 | 18/20 | 24 | 9.822 |
| CELL\_DEVELOPMENT (c5) Genes annotated by the GO term GO:0048468. The process whose specific outcome is the progression of the cell over time, from its formation to the mature structure. Cell development does not include the steps involved in committing a cell to a specific fate. | 4.14741e-07 | 549/571 | 650 | 552.489 |
| module\_252 (c4) Genes in module\_252 | 4.15124e-07 | 222/235 | 254 | 192.732 |
| NEGATIVE\_REGULATION\_OF\_CELLULAR\_PROCESS (c5) Genes annotated by the GO term GO:0048523. Any process that stops, prevents or reduces the frequency, rate or extent of cellular processes, those that are carried out at the cellular level, but are not necessarily restricted to a single cell. For example, cell communication occurs among more than one cell, but occurs at the cellular level. | 4.17769e-07 | 604/640 | 660 | 560.598 |
| V$GR\_Q6\_01 (c3) Genes with promoter regions [-2kb,2kb] around transcription start site containing the motif NNTGTYCT which matches annotation for NR3C1: nuclear receptor subfamily 3, group C, member 1 (glucocorticoid receptor) | 4.32577e-07 | 166/196 | 113 | 74.559 |
| STURLA\_SONIC\_HEDGEHOG (c2) Sonic hedgehog related genes | 4.40748e-07 | 14/16 | 37 | 18.332 |
| REGULATION\_OF\_TRANSCRIPTION (c5) Genes annotated by the GO term GO:0045449. Any process that modulates the frequency, rate or extent of the synthesis of either RNA on a template of DNA or DNA on a template of RNA. | 4.47164e-07 | 534/563 | 490 | 406.883 |
| NEGATIVE\_REGULATION\_OF\_BIOLOGICAL\_PROCESS (c5) Genes annotated by the GO term GO:0048519. Any process that stops, prevents or reduces the frequency, rate or extent of a biological process. Biological processes are regulated by many means; examples include the control of gene expression, protein modification or interaction with a protein or substrate molecule. | 4.63562e-07 | 633/670 | 684 | 583.574 |
| CELL\_CYCLE\_PROCESS (c5) Genes annotated by the GO term GO:0022402. A cellular process that is involved in the progression of biochemical and morphological phases and events that occur in a cell during successive cell replication or nuclear replication events. | 4.86692e-07 | 182/191 | 173 | 125.453 |
| HEMATOPOESIS\_RELATED\_TRANSCRIPTION\_FACTORS (c2) Transcription factors involved in hematopoiesis | 5.56317e-07 | 82/83 | 190 | 140.167 |
| V$TTF1\_Q6 (c3) Genes with promoter regions [-2kb,2kb] around transcription start site containing the motif NNNNCAAGNRNN which matches annotation for TITF1: thyroid transcription factor 1 | 6.42003e-07 | 160/198 | 124 | 84.362 |
| CATTGTYY\_V$SOX9\_B1 (c3) Genes with promoter regions [-2kb,2kb] around transcription start site containing the motif CATTGTYY which matches annotation for SOX9: SRY (sex determining region Y)-box 9 (campomelic dysplasia, autosomal sex-reversal) | 6.88175e-07 | 239/279 | 189 | 138.271 |
| CELL\_CYCLE\_PHASE (c5) Genes annotated by the GO term GO:0022403. A cell cycle process comprising the steps by which a cell progresses through one of the biochemical and morphological phases and events that occur during successive cell replication or nuclear replication events. | 7.44112e-07 | 163/169 | 165 | 119.269 |
| EMBRYONIC\_DEVELOPMENT (c5) Genes annotated by the GO term GO:0009790. The process whose specific outcome is the progression of an embryo from its formation until the end of its embryonic life stage. The end of the embryonic stage is organism-specific. For example, for mammals, the process would begin with zygote formation and end with birth. For insects, the process would begin at zygote formation and end with larval hatching. For plant zygotic embryos, this would be from zygote formation to the end of seed dormancy. For plant vegetative embryos, this would be from the initial determination of the cell or group of cells to form an embryo until the point when the embryo becomes independent of the parent plant. | 7.46924e-07 | 47/57 | 69 | 40.659 |
| V$NKX62\_Q2 (c3) Genes with promoter regions [-2kb,2kb] around transcription start site containing the motif NWADTAAWTANN which matches annotation for NKX6-2: NK6 transcription factor related, locus 2 (Drosophila) | 8.65656e-07 | 151/186 | 107 | 70.18 |
| V$LMO2COM\_02 (c3) Genes with promoter regions [-2kb,2kb] around transcription start site containing the motif NMGATANSG which matches annotation for LMO2: LIM domain only 2 (rhombotin-like 1) | 8.88873e-07 | 169/193 | 106 | 68.886 |
| REGULATION\_OF\_NUCLEOBASE\_\_NUCLEOSIDE\_\_NUCLEOTIDE\_AND\_NUCLEIC\_ACID\_METABOLIC\_PROCESS (c5) Genes annotated by the GO term GO:0019219. Any process that modulates the frequency, rate or extent of the chemical reactions and pathways involving nucleobases, nucleosides, nucleotides and nucleic acids. | 8.96061e-07 | 581/614 | 518 | 434.098 |
| CELL\_CYCLE\_REGULATOR (c2) Obsolete by GO - was not defined before being made obsolete | 9.04875e-07 | 19/21 | 46 | 24.981 |
| V$POU1F1\_Q6 (c3) Genes with promoter regions [-2kb,2kb] around transcription start site containing the motif ATGAATAAWT which matches annotation for POU1F1: POU domain, class 1, transcription factor 1 (Pit1, growth hormone factor 1) | 9.14475e-07 | 157/183 | 117 | 77.155 |
| HSA04012\_ERBB\_SIGNALING\_PATHWAY (c2) Genes involved in ErbB signaling pathway | 9.2441e-07 | 85/87 | 265 | 209.858 |
| WNT\_SIGNALING (c2) Wnt signaling genes | 9.35377e-07 | 58/61 | 127 | 88.612 |
| V$TFIII\_Q6 (c3) Genes with promoter regions [-2kb,2kb] around transcription start site containing the motif RGAGGKAGG which matches annotation for GTF2A1: general transcription factor IIA, 1, 19/37kDa  GTF2A2: general transcription factor IIA, 2, 12kDa | 9.36627e-07 | 146/165 | 111 | 72.568 |
| BIOPOLYMER\_METABOLIC\_PROCESS | 1.00813e-06 | 1549/1667 | 1034 | 921.635 |
| REGULATION\_OF\_CYCLIN\_DEPENDENT\_PROTEIN\_KINASE\_ACTIVITY (c5) Genes annotated by the GO term GO:0000079. Any process that modulates the frequency, rate or extent of CDK activity. | 1.07491e-06 | 42/43 | 60 | 34.536 |
| V$SOX9\_B1 (c3) Genes with promoter regions [-2kb,2kb] around transcription start site containing the motif NNNNAACAATRGNN which matches annotation for SOX9: SRY (sex determining region Y)-box 9 (campomelic dysplasia, autosomal sex-reversal) | 1.07788e-06 | 161/185 | 121 | 82.728 |
| FOSBPATHWAY (c2) FOSB gene expression and drug abuse | 1.17857e-06 | 4/5 | 16 | 5.802 |
| CTTTGA\_V$LEF1\_Q2 (c3) Genes with promoter regions [-2kb,2kb] around transcription start site containing the motif CTTTGA which matches annotation for LEF1: lymphoid enhancer-binding factor 1 | 1.26056e-06 | 738/901 | 370 | 298.139 |
| TRANSCRIPTION\_FROM\_RNA\_POLYMERASE\_II\_PROMOTER (c5) Genes annotated by the GO term GO:0006366. The synthesis of RNA from a DNA template by RNA polymerase II (Pol II), originating at a Pol II-specific promoter. Includes transcription of messenger RNA (mRNA) and certain small nuclear RNAs (snRNAs). | 1.26449e-06 | 435/456 | 397 | 324.033 |
| SKP2E2FPATHWAY (c2) E2F-1, a transcription factor that promotes the G1/S transition, is repressed by Rb and activated by cdk2/cyclin E. | 1.32525e-06 | 8/9 | 34 | 17.035 |
| POD1\_KO\_UP (c2) Up-regulated in glomeruli isolated from Pod1 knockout mice, versus wild-type controls | 1.46093e-06 | 305/369 | 204 | 152.207 |
| TAATTA\_V$CHX10\_01 (c3) Genes with promoter regions [-2kb,2kb] around transcription start site containing the motif TAATTA which matches annotation for VSX1: visual system homeobox 1 homolog, CHX10-like (zebrafish) | 1.48786e-06 | 485/612 | 263 | 203.22 |
| V$AHRARNT\_01 (c3) Genes with promoter regions [-2kb,2kb] around transcription start site containing the motif KNNKNNTYGCGTGCMS which matches annotation for AHR: aryl hydrocarbon receptor | 1.54246e-06 | 93/108 | 82 | 51.142 |
| chr7q36 (c1) Genes in cytogenetic band chr7q36 | 1.62326e-06 | 31/68 | 23 | 9.746 |
| V$PAX4\_03 (c3) Genes with promoter regions [-2kb,2kb] around transcription start site containing the motif NNNNNYCACCCB which matches annotation for PAX4: paired box gene 4 | 1.6313e-06 | 169/200 | 126 | 85.477 |
| INTERPHASE (c5) Genes annotated by the GO term GO:0051325. Progression through interphase, the stage of cell cycle between successive rounds of chromosome segregation. Canonically, interphase is the stage of the cell cycle during which the biochemical and physiologic functions of the cell are performed and replication of chromatin occurs. | 1.64496e-06 | 67/68 | 88 | 56.084 |
| V$TEF\_Q6 (c3) Genes with promoter regions [-2kb,2kb] around transcription start site containing the motif ATGTTWAYATAA which matches annotation for TEF: thyrotrophic embryonic factor | 1.67677e-06 | 162/195 | 101 | 67.288 |
| ABRAHAM\_AL\_VS\_MM\_DN (c2) Genes with significantly lower average gene expression in AL plasma cells than in MM cells | 2.1774e-06 | 17/18 | 71 | 45.066 |
| ROTH\_HTERT\_DIFF (c2) Expression of selected genes involved in DNA repair and cell-cycle control in hTERT-transduced T cells | 3.28228e-06 | 28/29 | 87 | 57.566 |
| RACCYCDPATHWAY (c2) Ras, Rac, and Rho coordinate to induce cyclin D1 expression and activate cdk2 to promote the G1/S transition. | 3.29287e-06 | 21/22 | 134 | 98.25 |
| chr5p11 (c1) Genes in cytogenetic band chr5p11 | 3.38959e-06 | 0/1 | 1 | 0.047 |
| CTTTGT\_V$LEF1\_Q2 (c3) Genes with promoter regions [-2kb,2kb] around transcription start site containing the motif CTTTGT which matches annotation for LEF1: lymphoid enhancer-binding factor 1 | 3.49578e-06 | 1197/1460 | 639 | 549.912 |
| NEGATIVE\_REGULATION\_OF\_GROWTH (c5) Genes annotated by the GO term GO:0045926. Any process that stops, prevents or reduces the rate or extent of growth, the increase in size or mass of all or part of an organism. | 3.67622e-06 | 34/40 | 81 | 53.38 |
| P35ALZHEIMERSPATHWAY (c2) p35, a neuron-specific activator of cyclin-dependent kinase 5, is cleaved to p25 in Alzheimer's disease and promotoes hyperphosphorylated tau formation and apoptosis. | 4.36991e-06 | 10/11 | 36 | 18.917 |
| V$TST1\_01 (c3) Genes with promoter regions [-2kb,2kb] around transcription start site containing the motif NNKGAATTAVAVTDN which matches annotation for POU3F1: POU domain, class 3, transcription factor 1 | 4.84854e-06 | 163/204 | 117 | 81.462 |
| module\_124 (c4) Genes in module\_124 | 5.00772e-06 | 94/96 | 96 | 62.623 |
| V$MEIS1AHOXA9\_01 (c3) Genes with promoter regions [-2kb,2kb] around transcription start site containing the motif TGACAGKTTTAYGA which matches annotation for MEIS1: Meis1, myeloid ecotropic viral integration site 1 homolog (mouse)  HOXA9: homeobox A9 | 5.22508e-06 | 77/93 | 56 | 33.153 |
| WTTGKCTG\_UNKNOWN | 5.24334e-06 | 324/390 | 201 | 152.38 |
| GGGTGGRR\_V$PAX4\_03 (c3) Genes with promoter regions [-2kb,2kb] around transcription start site containing the motif GGGTGGRR which matches annotation for PAX4: paired box gene 4 | 5.34426e-06 | 865/1011 | 586 | 504.885 |
| HSA05214\_GLIOMA (c2) Genes involved in glioma | 5.39489e-06 | 61/64 | 209 | 162.51 |
| ABBUD\_LIF\_DN (c2) Genes down-regulated by LIF treatment (10 ng/ml, overnight) in AtT20 cells | 5.48401e-06 | 20/24 | 17 | 6.375 |
| HSA04510\_FOCAL\_ADHESION (c2) Genes involved in focal adhesion | 5.62236e-06 | 184/192 | 364 | 301.874 |
| CAGGTG\_V$E12\_Q6 (c3) Genes with promoter regions [-2kb,2kb] around transcription start site containing the motif CAGGTG which matches annotation for TCF3: transcription factor 3 (E2A immunoglobulin enhancer binding factors E12/E47) | 5.74163e-06 | 1486/1832 | 787 | 693.38 |
| V$E2F1\_Q3\_01 (c3) Genes with promoter regions [-2kb,2kb] around transcription start site containing the motif TTGGCGCGRAANNGNM which matches annotation for E2F1: E2F transcription factor 1 | 6.00893e-06 | 168/193 | 114 | 78.645 |
| V$E2F1\_Q4\_01 (c3) Genes with promoter regions [-2kb,2kb] around transcription start site containing the motif TTTSGCGSG which matches annotation for E2F  TFDP1: transcription factor Dp-1 | 7.22113e-06 | 141/172 | 123 | 86.933 |
| V$LMO2COM\_01 | 7.32926e-06 | 159/195 | 115 | 78.332 |
| BRENTANI\_TRANSCRIPTION\_FACTORS (c2) Cancer related genes that are also transcription factors | 8.7092e-06 | 61/62 | 152 | 112.542 |
| V$FOX\_Q2 (c3) Genes with promoter regions [-2kb,2kb] around transcription start site containing the motif KATTGTTTRTTTW which matches annotation for FOXF2: forkhead box F2 | 9.11872e-06 | 135/163 | 81 | 52.749 |
| V$E2F\_Q3\_01 (c3) Genes with promoter regions [-2kb,2kb] around transcription start site containing the motif TTTSGCGSG which matches annotation for E2F  TFDP1: transcription factor Dp-1 | 9.45696e-06 | 143/176 | 125 | 89.235 |
| V$NFY\_Q6 | 9.61206e-06 | 158/197 | 117 | 82.258 |
| MITOTIC\_CELL\_CYCLE (c5) Genes annotated by the GO term GO:0000278. Progression through the phases of the mitotic cell cycle, the most common eukaryotic cell cycle, which canonically comprises four successive phases called G1, S, G2, and M and includes replication of the genome and the subsequent segregation of chromosomes into daughter cells. In some variant cell cycles nuclear replication or nuclear division may not be followed by cell division, or G1 and G2 phases may be absent. | 9.82109e-06 | 147/153 | 154 | 114.135 |
| V$PBX1\_02 (c3) Genes with promoter regions [-2kb,2kb] around transcription start site containing the motif NNCATCAATCAANNW which matches annotation for PBX1: pre-B-cell leukemia transcription factor 1 | 1.03949e-05 | 92/106 | 96 | 64.342 |
| INTERPHASE\_OF\_MITOTIC\_CELL\_CYCLE (c5) Genes annotated by the GO term GO:0051329. Progression through interphase, the stage of cell cycle between successive rounds of mitosis. Canonically, interphase is the stage of the cell cycle during which the biochemical and physiologic functions of the cell are performed and replication of chromatin occurs. | 1.06563e-05 | 61/62 | 77 | 49.522 |
| KCCGNSWTTT\_UNKNOWN (c3) Genes with promoter regions [-2kb,2kb] around transcription start site containing motif KCCGNSWTTT. Motif does not match any known transcription factor | 1.09248e-05 | 74/82 | 64 | 39.507 |
| DNA\_METABOLIC\_PROCESS (c5) Genes annotated by the GO term GO:0006259. The chemical reactions and pathways involving DNA, deoxyribonucleic acid, one of the two main types of nucleic acid, consisting of a long, unbranched macromolecule formed from one, or more commonly, two, strands of linked deoxyribonucleotides. | 1.34214e-05 | 243/256 | 231 | 181.656 |
| SHH\_UP (c2) Upregulated by sonic hedgehog (shh) expression in murine neuronal precursor cells | 1.41456e-05 | 8/9 | 17 | 6.798 |
| GROWTH\_FACTOR\_ACTIVITY (c5) Genes annotated by the GO term GO:0008083. The function that stimulates a cell to grow or proliferate. Most growth factors have other actions besides the induction of cell growth or proliferation. | 1.42045e-05 | 52/55 | 97 | 66.94 |
| V$PPARA\_02 (c3) Genes with promoter regions [-2kb,2kb] around transcription start site containing the motif NNRGGTCATWGGGGTSANG which matches annotation for PPARA: peroxisome proliferative activated receptor, alpha | 1.49901e-05 | 83/96 | 51 | 29.477 |
| REGULATION\_OF\_TRANSCRIPTION\_\_DNA\_DEPENDENT (c5) Genes annotated by the GO term GO:0006355. Any process that modulates the frequency, rate or extent of DNA-dependent transcription. | 1.65986e-05 | 434/459 | 386 | 323.919 |
| V$TCF11\_01 (c3) Genes with promoter regions [-2kb,2kb] around transcription start site containing the motif GTCATNNWNNNNN which matches annotation for NFE2L1: nuclear factor (erythroid-derived 2)-like 1 | 1.71515e-05 | 148/174 | 122 | 88.362 |
| CMV\_HCMV\_6HRS\_DN (c2) Down-regulated in fibroblasts at 6 hours following infection with human cytomegalovirus (CMV) | 1.75713e-05 | 44/51 | 43 | 23.663 |
| V$PR\_Q2 (c3) Genes with promoter regions [-2kb,2kb] around transcription start site containing the motif NWNAGRACAN which matches annotation for NR3C1: nuclear receptor subfamily 3, group C, member 1 (glucocorticoid receptor) | 1.78968e-05 | 167/194 | 92 | 60.946 |
| module\_403 (c4) Genes in module\_403 | 1.79379e-05 | 43/46 | 66 | 41.436 |
| HSA05215\_PROSTATE\_CANCER (c2) Genes involved in prostate cancer | 1.83825e-05 | 83/87 | 273 | 222.88 |
| VERNELL\_PRB\_CLSTR1 (c2) pRB pathway target genes CLUSTER 1 The listed genes were found regulated by pRB and p16 and one of the E2Fs (E2F1, E2F2, or E2F3) Cluster 1 genes are up-regulated by E2F and down-regulated by pRB and p16 | 1.84769e-05 | 52/61 | 48 | 28.589 |
| SMTTTTGT\_UNKNOWN (c3) Genes with promoter regions [-2kb,2kb] around transcription start site containing motif SMTTTTGT. Motif does not match any known transcription factor | 1.85611e-05 | 279/321 | 184 | 140.181 |
| V$AP2\_Q6 (c3) Genes with promoter regions [-2kb,2kb] around transcription start site containing the motif MKCCCSCNGGCG which matches annotation for GTF3A: general transcription factor IIIA | 1.86974e-05 | 162/192 | 110 | 77.192 |
| CPR\_NULL\_LIVER\_DN (c2) Down-regulated in mouse liver tissue from mice in which NADPH-cytochrome P450 reductase (CPR) was specifically deleted in the liver by cre-lox recombination, versus lox-only controls | 1.91099e-05 | 14/18 | 11 | 3.55 |
| V$FREAC4\_01 (c3) Genes with promoter regions [-2kb,2kb] around transcription start site containing the motif CTWAWGTAAACANWGN which matches annotation for FOXD1: forkhead box D1 | 1.93998e-05 | 88/113 | 61 | 38.161 |
| V$TCF4\_Q5 (c3) Genes with promoter regions [-2kb,2kb] around transcription start site containing the motif SCTTTGAW which matches annotation for TCF4: transcription factor 4 | 2.08017e-05 | 157/187 | 92 | 61.689 |
| MCAATNNNNNGCG\_UNKNOWN (c3) Genes with promoter regions [-2kb,2kb] around transcription start site containing motif MCAATNNNNNGCG. Motif does not match any known transcription factor | 2.09275e-05 | 51/62 | 48 | 28.386 |
| GGGCGGR\_V$SP1\_Q6 (c3) Genes with promoter regions [-2kb,2kb] around transcription start site containing the motif GGGCGGR which matches annotation for SP1: Sp1 transcription factor | 2.10069e-05 | 1819/2167 | 942 | 848.665 |
| POSITIVE\_REGULATION\_OF\_PHOSPHATE\_METABOLIC\_PROCESS (c5) Genes annotated by the GO term GO:0045937. Any process that activates or increases the frequency, rate or extent of the chemical reactions and pathways involving phosphates. | 2.15681e-05 | 27/28 | 88 | 61.077 |
| EPITHELIAL\_TO\_MESENCHYMAL\_TRANSITION (c5) Genes annotated by the GO term GO:0001837. A transition where an epithelial cell loses apical/basolateral polarity, severs intercellular adhesive junctions, degrades basement membrane components and becomes a migratory mesenchymal cell. | 2.19304e-05 | 9/10 | 35 | 19.102 |
| V$PAX4\_02 (c3) Genes with promoter regions [-2kb,2kb] around transcription start site containing the motif NAAWAATTANS which matches annotation for PAX4: paired box gene 4 | 2.23417e-05 | 155/191 | 91 | 62.933 |
| REGIONALIZATION (c5) Genes annotated by the GO term GO:0003002. The pattern specification process by which an axis or axes is subdivided in space to define an area or volume in which specific patterns of cell differentiation will take place or in which cells interpret a specific environment. | 2.30615e-05 | 12/15 | 25 | 12.327 |
| ATGCTGC,MIR-103,MIR-107 (c3) Targets of MicroRNA ATGCTGC,MIR-103,MIR-107 | 2.35677e-05 | 147/198 | 100 | 69.105 |
| WNT\_TARGETS (c2) WNT target genes from literatures | 2.56012e-05 | 21/22 | 60 | 39.065 |
| V$ZF5\_B (c3) Genes with promoter regions [-2kb,2kb] around transcription start site containing the motif NRNGNGCGCGCWN which matches annotation for ZFP161: zinc finger protein 161 homolog (mouse) | 2.60782e-05 | 154/188 | 86 | 58.092 |
| WNTPATHWAY (c2) The Wnt glycoprotein binds to membrane-bound receptors such as Frizzled to activate a number of signaling pathways, including that of beta-catenin. | 2.6519e-05 | 23/24 | 75 | 50.849 |
| CELL\_CYCLE\_ARREST (c2) Any process by which progression through the cell cycle is halted during one of the normal phases (G1, S, G2, M). | 2.72316e-05 | 29/30 | 53 | 32.514 |
| V$E2F\_Q6\_01 (c3) Genes with promoter regions [-2kb,2kb] around transcription start site containing the motif NKCGCGCSAAAN which matches annotation for E2F  TFDP1: transcription factor Dp-1 | 2.7339e-05 | 141/171 | 115 | 82.443 |
| TTGTTT\_V$FOXO4\_01 | 2.7695e-05 | 1248/1511 | 673 | 589.7 |
| POMEROY\_DESMOPLASIC\_VS\_CLASSIC\_MD\_UP (c2) Genes expressed in desmoplastic medulloblastomas. (p < 0.01) | 2.8359e-05 | 33/42 | 30 | 15.258 |
| YKACATTT\_UNKNOWN (c3) Genes with promoter regions [-2kb,2kb] around transcription start site containing motif YKACATTT. Motif does not match any known transcription factor | 2.85501e-05 | 157/216 | 124 | 88.636 |
| V$CIZ\_01 (c3) Genes with promoter regions [-2kb,2kb] around transcription start site containing the motif SAAAAANNN which matches annotation for ZNF384: zinc finger protein 384 | 2.91626e-05 | 144/174 | 95 | 65.23 |
| BREASTCA\_TWO\_CLASSES (c2) Gene set that can be used to differentiate BRCA1-linked and BRCA2-linked breast cancers | 3.1011e-05 | 121/132 | 155 | 117.835 |
