# Supplementary material for: Looking at Cerebellar Malformations through Text-Mined Interactomes of Mice and Humans
Source: PLoS Comput Biol. 2009 Nov 6;5(11):e1000559. doi: 10.1371/journal.pcbi.1000559 (PMC2767227; doi:10.1371/journal.pcbi.1000559)
Supplement: Dataset S1 — All enrichment results. (0.20 MB ZIP) [file pcbi.1000559.s012.zip › enrichment_results/Table X. enrichment_whole-small cerebellum.html]

Complete Clustering results for network whole and phenotype small cerebellum (FDR <= 0.001)


# Complete Clustering results for network whole and phenotype small cerebellum (FDR <= 0.001)

| Set | p-Value | Gene Count | Interaction Count | Expected Interection Count |
| --- | --- | --- | --- | --- |
| NERVOUS\_SYSTEM\_DEVELOPMENT (c5) Genes annotated by the GO term GO:0007399. The process whose specific outcome is the progression of nervous tissue over time, from its formation to its mature state. | 1e-20 | 331/382 | 220 | 130.64 |
| HSA04340\_HEDGEHOG\_SIGNALING\_PATHWAY (c2) Genes involved in Hedgehog signaling pathway | 1e-20 | 53/57 | 91 | 33.014 |
| HSA05217\_BASAL\_CELL\_CARCINOMA (c2) Genes involved in basal cell carcinoma | 2.22045e-16 | 53/55 | 89 | 41.219 |
| MULTICELLULAR\_ORGANISMAL\_DEVELOPMENT (c5) Genes annotated by the GO term GO:0007275. The biological process whose specific outcome is the progression of an organism over time from an initial condition (e.g. a zygote or a young adult) to a later condition (e.g. a multicellular animal or an aged adult). | 3.39728e-14 | 926/1045 | 585 | 453.164 |
| SYSTEM\_DEVELOPMENT (c5) Genes annotated by the GO term GO:0048731. The process whose specific outcome is the progression of an organismal system over time, from its formation to the mature structure. A system is a regularly interacting or interdependent group of organs or tissues that work together to carry out a given biological process. | 4.29878e-13 | 777/858 | 503 | 386.207 |
| ANATOMICAL\_STRUCTURE\_DEVELOPMENT (c5) Genes annotated by the GO term GO:0048856. The biological process whose specific outcome is the progression of an anatomical structure from an initial condition to its mature state. This process begins with the formation of the structure and ends with the mature structure, whatever form that may be including its natural destruction. An anatomical structure is any biological entity that occupies space and is distinguished from its surroundings. Anatomical structures can be macroscopic such as a carpel, or microscopic such as an acrosome. | 9.18932e-13 | 908/1012 | 550 | 427.825 |
| SA\_REG\_CASCADE\_OF\_CYCLIN\_EXPR (c2) Expression of cyclins regulates progression through the cell cycle by activating cyclin-dependent kinases. | 4.48086e-12 | 12/13 | 51 | 22.56 |
| CELLCYCLEPATHWAY (c2) Cyclins interact with cyclin-dependent kinases to form active kinase complexes that regulate progression through the cell cycle. | 5.90827e-12 | 22/23 | 69 | 33.533 |
| V$TFIII\_Q6 (c3) Genes with promoter regions [-2kb,2kb] around transcription start site containing the motif RGAGGKAGG which matches annotation for GTF2A1: general transcription factor IIA, 1, 19/37kDa  GTF2A2: general transcription factor IIA, 2, 12kDa | 1.54463e-11 | 146/165 | 99 | 53.77 |
| REELINPATHWAY | 1.56041e-11 | 6/7 | 24 | 7.698 |
| ANATOMICAL\_STRUCTURE\_MORPHOGENESIS (c5) Genes annotated by the GO term GO:0009653. The process by which anatomical structures are generated and organized. Morphogenesis pertains to the creation of form. | 2.51162e-11 | 345/379 | 258 | 178.58 |
| G1\_TO\_S\_CELL\_CYCLE\_REACTOME (c2) | 3.41518e-11 | 65/66 | 116 | 69.261 |
| EMBRYONIC\_MORPHOGENESIS (c5) Genes annotated by the GO term GO:0048598. The process by which anatomical structures are generated and organized during the embryonic phase. Morphogenesis pertains to the creation of form. The embryonic phase begins with zygote formation. The end of the embryonic phase is organism-specific. For example, it would be at birth for mammals, larval hatching for insects and seed dormancy in plants. | 1.35463e-10 | 14/17 | 22 | 6.669 |
| G1PATHWAY (c2) CDK4/6-cyclin D and CDK2-cyclin E phosphorylate Rb, which allows the transcription of genes needed for the G1/S cell cycle transition. | 1.87067e-10 | 25/26 | 99 | 57.512 |
| module\_220 (c4) Genes in module\_220 | 2.03404e-10 | 316/329 | 250 | 175.811 |
| CELL\_FATE\_COMMITMENT (c5) Genes annotated by the GO term GO:0045165. The commitment of cells to specific cell fates and their capacity to differentiate into particular kinds of cells. Positional information is established through protein signals that emanate from a localized source within a cell (the initial one-cell zygote) or within a developmental field. | 3.09718e-10 | 11/13 | 28 | 10.271 |
| BRENTANI\_CELL\_CYCLE (c2) Cancer related genes involved in the cell cycle | 3.26819e-10 | 78/79 | 128 | 78.753 |
| CORTEX\_ENRICHMENT\_LATE\_UP (c2) Up-regulated in the cortex of mice that are exposed to an enriched environmental habitat for 2 or 14 days | 3.69606e-10 | 18/20 | 23 | 7.347 |
| ACATATC,MIR-190 (c3) Targets of MicroRNA ACATATC,MIR-190 | 4.64983e-10 | 39/56 | 41 | 17.328 |
| V$T3R\_Q6 (c3) Genes with promoter regions [-2kb,2kb] around transcription start site containing motif MNTGWCCTN. Motif does not match any known transcription factor | 4.75358e-10 | 160/194 | 78 | 40.86 |
| TGCACTT,MIR-519C,MIR-519B,MIR-519A (c3) Targets of MicroRNA TGCACTT,MIR-519C,MIR-519B,MIR-519A | 6.52495e-10 | 299/394 | 152 | 99.471 |
| GROWTH\_CONE (c5) Genes annotated by the GO term GO:0030426. The migrating motile tip of a growing nerve cell axon or dendrite. | 1.21404e-09 | 9/10 | 26 | 9.152 |
| SHHPATHWAY (c2) Sonic hedgehog (Shh) signaling in the developing CNS induces neuronal proliferation via interaction with the patched (Ptc-1) and smoothened receptors. | 1.2149e-09 | 12/14 | 24 | 8.475 |
| EMBRYONIC\_DEVELOPMENT (c5) Genes annotated by the GO term GO:0009790. The process whose specific outcome is the progression of an embryo from its formation until the end of its embryonic life stage. The end of the embryonic stage is organism-specific. For example, for mammals, the process would begin with zygote formation and end with birth. For insects, the process would begin at zygote formation and end with larval hatching. For plant zygotic embryos, this would be from zygote formation to the end of seed dormancy. For plant vegetative embryos, this would be from the initial determination of the cell or group of cells to form an embryo until the point when the embryo becomes independent of the parent plant. | 1.24628e-09 | 47/57 | 61 | 29.297 |
| V$E2F1\_Q3\_01 (c3) Genes with promoter regions [-2kb,2kb] around transcription start site containing the motif TTGGCGCGRAANNGNM which matches annotation for E2F1: E2F transcription factor 1 | 2.07288e-09 | 168/193 | 101 | 58.387 |
| chr20p11 (c1) Genes in cytogenetic band chr20p11 | 2.96362e-09 | 26/68 | 15 | 3.85 |
| HSA05214\_GLIOMA (c2) Genes involved in glioma | 4.1089e-09 | 61/64 | 178 | 122.937 |
| MCAATNNNNNGCG\_UNKNOWN (c3) Genes with promoter regions [-2kb,2kb] around transcription start site containing motif MCAATNNNNNGCG. Motif does not match any known transcription factor | 5.71367e-09 | 51/62 | 47 | 21.509 |
| CENTRAL\_NERVOUS\_SYSTEM\_DEVELOPMENT (c5) Genes annotated by the GO term GO:0007417. The process whose specific outcome is the progression of the central nervous system over time, from its formation to the mature structure. The central nervous system is the core nervous system that serves an integrating and coordinating function. In vertebrates it consists of the brain, spinal cord and spinal nerves. In those invertebrates with a central nervous system it typically consists of a brain, cerebral ganglia and a nerve cord. | 6.45384e-09 | 109/123 | 65 | 34.051 |
| SITE\_OF\_POLARIZED\_GROWTH (c5) Genes annotated by the GO term GO:0030427. Any part of a cell where non-isotropic growth takes place. | 6.81323e-09 | 10/11 | 26 | 9.665 |
| GLAND\_DEVELOPMENT (c5) Genes annotated by the GO term GO:0048732. The process whose specific outcome is the progression of a gland over time, from its formation to the mature structure. A gland is an organ specialised for secretion. | 7.18785e-09 | 12/13 | 28 | 10.91 |
| chr6p11 (c1) Genes in cytogenetic band chr6p11 | 1.09446e-08 | 3/6 | 2 | 0.119 |
| CELL\_CYCLE (c2) The progression of biochemical and morphological events that occur during nuclear or cellular replication. | 1.17133e-08 | 73/76 | 139 | 92.058 |
| V$ZF5\_B (c3) Genes with promoter regions [-2kb,2kb] around transcription start site containing the motif NRNGNGCGCGCWN which matches annotation for ZFP161: zinc finger protein 161 homolog (mouse) | 1.63279e-08 | 154/188 | 78 | 42.95 |
| HSA05218\_MELANOMA (c2) Genes involved in melanoma | 1.71906e-08 | 65/71 | 174 | 121.317 |
| HSA04950\_MATURITY\_ONSET\_DIABETES\_OF\_THE\_YOUNG (c2) Genes involved in maturity onset diabetes of the young (MODY) | 1.90455e-08 | 23/25 | 38 | 17.1 |
| ORGAN\_MORPHOGENESIS (c5) Genes annotated by the GO term GO:0009887. Morphogenesis of an organ. An organ is defined as a tissue or set of tissues that work together to perform a specific function or functions. Morphogenesis is the process by which anatomical structures are generated and organized. Organs are commonly observed as visibly distinct structures, but may also exist as loosely associated clusters of cells that work together to perform a specific function or functions. | 2.1433e-08 | 136/145 | 141 | 94.447 |
| V$CIZ\_01 (c3) Genes with promoter regions [-2kb,2kb] around transcription start site containing the motif SAAAAANNN which matches annotation for ZNF384: zinc finger protein 384 | 2.27789e-08 | 144/174 | 82 | 47.87 |
| BRAIN\_DEVELOPMENT (c5) Genes annotated by the GO term GO:0007420. The process whose specific outcome is the progression of the brain over time, from its formation to the mature structure. The brain is one of the two components of the central nervous system and is the center of thought and emotion. It is responsible for the coordination and control of bodily activities and the interpretation of information from the senses (sight, hearing, smell, etc.). | 2.32077e-08 | 44/51 | 32 | 13.041 |
| CAGGTG\_V$E12\_Q6 (c3) Genes with promoter regions [-2kb,2kb] around transcription start site containing the motif CAGGTG which matches annotation for TCF3: transcription factor 3 (E2A immunoglobulin enhancer binding factors E12/E47) | 2.65429e-08 | 1486/1832 | 622 | 517.14 |
| YTAATTAA\_V$LHX3\_01 (c3) Genes with promoter regions [-2kb,2kb] around transcription start site containing the motif YTAATTAA which matches annotation for LHX3: LIM homeobox 3 | 5.14609e-08 | 119/145 | 53 | 26.436 |
| RUIZ\_TENASCIN\_TARGETS (c2) Tenascin-C target genes | 5.5477e-08 | 76/77 | 78 | 45.053 |
| HSA04115\_P53\_SIGNALING\_PATHWAY (c2) Genes involved in p53 signaling pathway | 6.05936e-08 | 64/66 | 119 | 78.176 |
| V$POU3F2\_02 (c3) Genes with promoter regions [-2kb,2kb] around transcription start site containing the motif TTATGYTAAT which matches annotation for POU3F2: POU domain, class 3, transcription factor 2 | 6.4302e-08 | 153/197 | 102 | 63.09 |
| HSA04510\_FOCAL\_ADHESION (c2) Genes involved in focal adhesion | 6.94266e-08 | 184/192 | 295 | 225.601 |
| V$MEIS1AHOXA9\_01 (c3) Genes with promoter regions [-2kb,2kb] around transcription start site containing the motif TGACAGKTTTAYGA which matches annotation for MEIS1: Meis1, myeloid ecotropic viral integration site 1 homolog (mouse)  HOXA9: homeobox A9 | 8.14994e-08 | 77/93 | 48 | 24.719 |
| GGGAGGRR\_V$MAZ\_Q6 | 8.1696e-08 | 1470/1733 | 665 | 559.5 |
| CGGTGTG,MIR-220 (c3) Targets of MicroRNA CGGTGTG,MIR-220 | 8.23995e-08 | 4/6 | 9 | 1.945 |
| CELL\_CYCLE\_KEGG (c2) | 9.33607e-08 | 80/84 | 146 | 100.391 |
| YATGNWAAT\_V$OCT\_C (c3) Genes with promoter regions [-2kb,2kb] around transcription start site containing motif YATGNWAAT. Motif does not match any known transcription factor | 1.19268e-07 | 229/289 | 131 | 85.259 |
| V$GR\_Q6 (c3) Genes with promoter regions [-2kb,2kb] around transcription start site containing the motif NNNNNNCNNTNTGTNCTNN which matches annotation for NR3C1: nuclear receptor subfamily 3, group C, member 1 (glucocorticoid receptor) | 1.64543e-07 | 172/207 | 90 | 54.7 |
| chr7q36 (c1) Genes in cytogenetic band chr7q36 | 1.71025e-07 | 31/68 | 20 | 7.109 |
| NEURITE\_DEVELOPMENT (c5) Genes annotated by the GO term GO:0031175. The process whose specific outcome is the progression of the neurite over time, from its formation to the mature structure. The neurite is any process extending from a neural cell, such as axons or dendrites. | 1.82339e-07 | 51/53 | 46 | 22.862 |
| chr1q24 (c1) Genes in cytogenetic band chr1q24 | 1.9241e-07 | 22/43 | 8 | 1.629 |
| V$FOXM1\_01 (c3) Genes with promoter regions [-2kb,2kb] around transcription start site containing the motif ARATKGAST which matches annotation for FOXM1: forkhead box M1 | 1.94628e-07 | 160/188 | 102 | 64.441 |
| SA\_G1\_AND\_S\_PHASES (c2) Cdk2, 4, and 6 bind cyclin D in G1, while cdk2/cyclin E promotes the G1/S transition. | 2.4289e-07 | 14/15 | 59 | 34.628 |
| INTERPHASE (c5) Genes annotated by the GO term GO:0051325. Progression through interphase, the stage of cell cycle between successive rounds of chromosome segregation. Canonically, interphase is the stage of the cell cycle during which the biochemical and physiologic functions of the cell are performed and replication of chromatin occurs. | 2.62397e-07 | 67/68 | 71 | 42.074 |
| V$E47\_01 (c3) Genes with promoter regions [-2kb,2kb] around transcription start site containing the motif VSNGCAGGTGKNCNN which matches annotation for TCF3: transcription factor 3 (E2A immunoglobulin enhancer binding factors E12/E47) | 2.63324e-07 | 168/200 | 90 | 54.655 |
| V$TEF\_Q6 (c3) Genes with promoter regions [-2kb,2kb] around transcription start site containing the motif ATGTTWAYATAA which matches annotation for TEF: thyrotrophic embryonic factor | 2.72766e-07 | 162/195 | 84 | 50.731 |
| POSITIVE\_REGULATION\_OF\_CELLULAR\_PROCESS (c5) Genes annotated by the GO term GO:0048522. Any process that activates or increases the frequency, rate or extent of cellular processes, those that are carried out at the cellular level, but are not necessarily restricted to a single cell. For example, cell communication occurs among more than one cell, but occurs at the cellular level. | 2.94945e-07 | 624/659 | 521 | 435.102 |
| MORPHOGENESIS\_OF\_AN\_EPITHELIUM (c5) Genes annotated by the GO term GO:0002009. The process by which the anatomical structures of epithelia are generated and organized. Morphogenesis pertains to the creation of form. An epithelium is a sheet of closely packed cells arranged in one or more layers, that covers the outer surfaces of the body or lines any internal cavity or tube. | 3.11285e-07 | 15/16 | 10 | 2.421 |
| REGULATION\_OF\_CELL\_CYCLE (c5) Genes annotated by the GO term GO:0051726. Any process that modulates the rate or extent of progression through the cell cycle. | 3.3528e-07 | 174/180 | 170 | 121.116 |
| WNT\_SIGNALING (c2) Wnt signaling genes | 3.4116e-07 | 58/61 | 103 | 66.698 |
| NEUROGENESIS (c5) Genes annotated by the GO term GO:0022008. Generation of cells within the nervous system. | 4.17859e-07 | 89/93 | 66 | 37.936 |
| P27PATHWAY (c2) p27 blocks the G1/S transition by inhibiting the checkpoint kinase cdk2/cyclin E and is inhibited by cdk2-mediated ubiquitination. | 4.64665e-07 | 11/12 | 33 | 15.439 |
| CAGCTG\_V$AP4\_Q5 (c3) Genes with promoter regions [-2kb,2kb] around transcription start site containing the motif CAGCTG which matches annotation for REPIN1: replication initiator 1 | 4.80564e-07 | 926/1126 | 429 | 349.519 |
| HSA04012\_ERBB\_SIGNALING\_PATHWAY (c2) Genes involved in ErbB signaling pathway | 4.86679e-07 | 85/87 | 207 | 156.418 |
| NEURON\_DEVELOPMENT (c5) Genes annotated by the GO term GO:0048666. The process whose specific outcome is the progression of a neuron over time, from initial commitment of the cell to a specific fate, to the fully functional differentiated cell. | 4.94018e-07 | 59/61 | 50 | 26.592 |
| FOSBPATHWAY (c2) FOSB gene expression and drug abuse | 5.01938e-07 | 4/5 | 14 | 4.467 |
| V$IPF1\_Q4 (c3) Genes with promoter regions [-2kb,2kb] around transcription start site containing the motif GHNNTAATGACM which matches annotation for IPF1: insulin promoter factor 1, homeodomain transcription factor | 5.27093e-07 | 159/186 | 88 | 53.554 |
| POMEROY\_DESMOPLASIC\_VS\_CLASSIC\_MD\_UP (c2) Genes expressed in desmoplastic medulloblastomas. (p < 0.01) | 5.48105e-07 | 33/42 | 27 | 11.088 |
| CAGGTA\_V$AREB6\_01 (c3) Genes with promoter regions [-2kb,2kb] around transcription start site containing the motif CAGGTA which matches annotation for TCF8: transcription factor 8 (represses interleukin 2 expression) | 5.77604e-07 | 471/582 | 235 | 176.941 |
| PROLIFERATION\_GENES (c2) Proliferation related genes | 6.23532e-07 | 343/359 | 320 | 251.851 |
| HSA05220\_CHRONIC\_MYELOID\_LEUKEMIA (c2) Genes involved in chronic myeloid leukemia | 6.51125e-07 | 74/76 | 209 | 157.495 |
| CELL\_PROLIFERATION\_GO\_0008283 (c5) Genes annotated by the GO term GO:0008283. The multiplication or reproduction of cells, resulting in the expansion of a cell population. | 6.85411e-07 | 487/513 | 369 | 295.993 |
| INTERPHASE\_OF\_MITOTIC\_CELL\_CYCLE (c5) Genes annotated by the GO term GO:0051329. Progression through interphase, the stage of cell cycle between successive rounds of mitosis. Canonically, interphase is the stage of the cell cycle during which the biochemical and physiologic functions of the cell are performed and replication of chromatin occurs. | 6.87728e-07 | 61/62 | 64 | 37.255 |
| HYPERME\_COLONCA\_SW48 (c2) Gene identified by chromatin IP and CpG island microarray as being hypermethylated in SW48 colon cancer cells, versus normal colon murcosa or WI38 fibroblasts | 7.07053e-07 | 9/14 | 19 | 6.924 |
| SYNAPSE (c5) Genes annotated by the GO term GO:0045202. The junction between a nerve fiber of one neuron and another neuron or muscle fiber or glial cell; the site of interneuronal communication. As the nerve fiber approaches the synapse it enlarges into a specialized structure, the presynaptic nerve ending, which contains mitochondria and synaptic vesicles. At the tip of the nerve ending is the presynaptic membrane; facing it, and separated from it by a minute cleft (the synaptic cleft) is a specialized area of membrane on the receiving cell, known as the postsynaptic membrane. In response to the arrival of nerve impulses, the presynaptic nerve ending secretes molecules of neurotransmitters into the synaptic cleft. These diffuse across the cleft and transmit the signal to the postsynaptic membrane. | 8.79825e-07 | 25/27 | 27 | 11.466 |
| GENERATION\_OF\_NEURONS (c5) Genes annotated by the GO term GO:0048699. The process by which nerve cells are generated. This includes the production of neuroblasts and their differentiation into neurons. | 9.17811e-07 | 79/83 | 61 | 34.783 |
| V$FAC1\_01 (c3) Genes with promoter regions [-2kb,2kb] around transcription start site containing the motif NNNCAMAACACRNA which matches annotation for FALZ: fetal Alzheimer antigen | 9.73407e-07 | 143/158 | 84 | 52.4 |
| HSA05215\_PROSTATE\_CANCER (c2) Genes involved in prostate cancer | 9.88909e-07 | 83/87 | 222 | 168.41 |
| CELL\_CYCLE\_GO\_0007049 (c5) Genes annotated by the GO term GO:0007049. The progression of biochemical and morphological phases and events that occur in a cell during successive cell replication or nuclear replication events. Canonically, the cell cycle comprises the replication and segregation of genetic material followed by the division of the cell, but in endocycles or syncytial cells nuclear replication or nuclear division may not be followed by cell division. | 1.09426e-06 | 299/311 | 222 | 166.257 |
| V$PIT1\_Q6 (c3) Genes with promoter regions [-2kb,2kb] around transcription start site containing the motif NMTTCATAAWTATWNMNA which matches annotation for POU1F1: POU domain, class 1, transcription factor 1 (Pit1, growth hormone factor 1) | 1.14701e-06 | 148/180 | 83 | 50.961 |
| WNT\_TARGETS (c2) WNT target genes from literatures | 1.22017e-06 | 21/22 | 51 | 28.815 |
| ABRAHAM\_AL\_VS\_MM\_DN (c2) Genes with significantly lower average gene expression in AL plasma cells than in MM cells | 1.31743e-06 | 17/18 | 58 | 34.144 |
| NEURON\_DIFFERENTIATION (c5) Genes annotated by the GO term GO:0030182. The process whereby a relatively unspecialized cell acquires specialized features of a neuron. | 1.62473e-06 | 72/76 | 56 | 31.839 |
| DIAB\_NEPH\_UP (c2) Upregulated in the glomeruli of cadaver kidneys from patients with diabetic nephropathy, compared to normal controls | 1.64246e-06 | 47/60 | 38 | 19.273 |
| HSA05223\_NON\_SMALL\_CELL\_LUNG\_CANCER (c2) Genes involved in non-small cell lung cancer | 1.70648e-06 | 53/54 | 147 | 106.572 |
| V$CDX2\_Q5 (c3) Genes with promoter regions [-2kb,2kb] around transcription start site containing the motif ANANTTTTATKRCC which matches annotation for CDX2: caudal type homeobox transcription factor 2 | 1.71499e-06 | 164/195 | 84 | 51.867 |
| POSITIVE\_REGULATION\_OF\_CELL\_PROLIFERATION (c5) Genes annotated by the GO term GO:0008284. Any process that activates or increases the rate or extent of cell proliferation. | 2.43531e-06 | 145/149 | 173 | 126.972 |
| V$MEIS1BHOXA9\_01 (c3) Genes with promoter regions [-2kb,2kb] around transcription start site containing the motif TGACAGTTTTAYGR which matches annotation for MEIS1: Meis1, myeloid ecotropic viral integration site 1 homolog (mouse)  HOXA9: homeobox A9 | 2.54916e-06 | 93/110 | 56 | 32.326 |
| RACCYCDPATHWAY | 2.68639e-06 | 21/22 | 107 | 73.693 |
| V$HNF6\_Q6 (c3) Genes with promoter regions [-2kb,2kb] around transcription start site containing the motif HWAAATCAATAW which matches annotation for ONECUT1: one cut domain, family member 1 | 2.99363e-06 | 150/183 | 108 | 74.349 |
| POSITIVE\_REGULATION\_OF\_BIOLOGICAL\_PROCESS (c5) Genes annotated by the GO term GO:0048518. Any process that activates or increases the frequency, rate or extent of a biological process. Biological processes are regulated by many means; examples include the control of gene expression, protein modification or interaction with a protein or substrate molecule. | 3.18524e-06 | 664/700 | 546 | 465.889 |
| CTTTGA\_V$LEF1\_Q2 (c3) Genes with promoter regions [-2kb,2kb] around transcription start site containing the motif CTTTGA which matches annotation for LEF1: lymphoid enhancer-binding factor 1 | 3.34743e-06 | 738/901 | 285 | 222.277 |
| V$FOX\_Q2 (c3) Genes with promoter regions [-2kb,2kb] around transcription start site containing the motif KATTGTTTRTTTW which matches annotation for FOXF2: forkhead box F2 | 4.12357e-06 | 135/163 | 66 | 39.262 |
| module\_137 (c4) Genes in module\_137 | 4.45987e-06 | 483/539 | 225 | 174.099 |
| HSA04070\_PHOSPHATIDYLINOSITOL\_SIGNALING\_SYSTEM (c2) Genes involved in phosphatidylinositol signaling system | 4.5077e-06 | 65/76 | 47 | 25.824 |
| GCACTTT,MIR-17-5P,MIR-20A,MIR-106A,MIR-106B,MIR-20B,MIR-519D (c3) Targets of MicroRNA GCACTTT,MIR-17-5P,MIR-20A,MIR-106A,MIR-106B,MIR-20B,MIR-519D | 4.60463e-06 | 394/528 | 189 | 141.185 |
| ATCMNTCCGY\_UNKNOWN (c3) Genes with promoter regions [-2kb,2kb] around transcription start site containing motif ATCMNTCCGY. Motif does not match any known transcription factor | 4.65297e-06 | 35/41 | 36 | 18.083 |
| V$HOX13\_01 (c3) Genes with promoter regions [-2kb,2kb] around transcription start site containing the motif TGCNHNCWYCCYCATTAKTNNDCNMNHYCN which matches annotation for HOXA5: homeobox A5 | 5.91146e-06 | 25/32 | 28 | 13.466 |
| GGCKCATGS\_UNKNOWN (c3) Genes with promoter regions [-2kb,2kb] around transcription start site containing motif GGCKCATGS. Motif does not match any known transcription factor | 6.05199e-06 | 34/44 | 36 | 18.274 |
| V$LMO2COM\_02 (c3) Genes with promoter regions [-2kb,2kb] around transcription start site containing the motif NMGATANSG which matches annotation for LMO2: LIM domain only 2 (rhombotin-like 1) | 6.23046e-06 | 169/193 | 83 | 52.188 |
| SKP2E2FPATHWAY (c2) E2F-1, a transcription factor that promotes the G1/S transition, is repressed by Rb and activated by cdk2/cyclin E. | 6.38801e-06 | 8/9 | 27 | 12.744 |
| AAAYWAACM\_V$HFH4\_01 (c3) Genes with promoter regions [-2kb,2kb] around transcription start site containing the motif AAAYWAACM which matches annotation for FOXJ1: forkhead box J1 | 6.51992e-06 | 163/195 | 86 | 54.794 |
| GCCNNNWTAAR\_UNKNOWN (c3) Genes with promoter regions [-2kb,2kb] around transcription start site containing motif GCCNNNWTAAR. Motif does not match any known transcription factor | 6.57128e-06 | 100/117 | 72 | 44.622 |
| V$GFI1\_01 (c3) Genes with promoter regions [-2kb,2kb] around transcription start site containing the motif NNNNNNNAAATCACWGYNNNNNNN which matches annotation for GFI1: growth factor independent 1 | 6.80564e-06 | 177/204 | 95 | 62.727 |
| CELL\_CYCLE\_REGULATOR (c2) Obsolete by GO - was not defined before being made obsolete | 6.8958e-06 | 19/21 | 36 | 18.993 |
| UNDERHILL\_PROLIFERATION (c2) Cell cycle- and proliferation-related genes underexpressed in plasma cells. | 7.50215e-06 | 16/18 | 28 | 13.224 |
| DNA\_BINDING (c5) Genes annotated by the GO term GO:0003677. Interacting selectively with DNA (deoxyribonucleic acid). | 7.6959e-06 | 540/600 | 350 | 284.499 |
| TRANSCRIPTION\_FACTOR\_ACTIVITY (c5) Genes annotated by the GO term GO:0003700. The function of binding to a specific DNA sequence in order to modulate transcription. The transcription factor may or may not also interact selectively with a protein or macromolecular complex. | 8.25788e-06 | 316/353 | 261 | 204.46 |
| REGULATION\_OF\_PHOSPHORYLATION (c5) Genes annotated by the GO term GO:0042325. Any process that modulates the frequency, rate or extent of addition of phosphate groups into a molecule. | 8.95071e-06 | 48/49 | 88 | 59.386 |
| GATTGGY\_V$NFY\_Q6\_01 (c3) Genes with promoter regions [-2kb,2kb] around transcription start site containing motif GATTGGY. Motif does not match any known transcription factor | 9.37483e-06 | 703/856 | 322 | 260.184 |
| V$OCT1\_04 (c3) Genes with promoter regions [-2kb,2kb] around transcription start site containing the motif NNNNNNNWATGCAAATNNNWNNA which matches annotation for POU2F1: POU domain, class 2, transcription factor 1 | 9.50039e-06 | 154/191 | 87 | 55.373 |
| CTTTGT\_V$LEF1\_Q2 (c3) Genes with promoter regions [-2kb,2kb] around transcription start site containing the motif CTTTGT which matches annotation for LEF1: lymphoid enhancer-binding factor 1 | 9.66035e-06 | 1197/1460 | 488 | 413.745 |
| module\_100 (c4) Genes in module\_100 | 9.73464e-06 | 481/536 | 222 | 173.121 |
| V$LMO2COM\_01 (c3) Genes with promoter regions [-2kb,2kb] around transcription start site containing the motif CNNCAGGTGBNN which matches annotation for LMO2: LIM domain only 2 (rhombotin-like 1) | 9.87877e-06 | 159/195 | 89 | 57.954 |
| REGULATION\_OF\_DNA\_REPLICATION (c5) Genes annotated by the GO term GO:0006275. Any process that modulates the frequency, rate or extent of DNA replication. | 1.00354e-05 | 18/20 | 23 | 10.175 |
| BREAST\_CANCER\_ESTROGEN\_SIGNALING (c2) Genes preferentially expressed in breast cancers, especially those involved in estrogen-receptor-dependent signal transduction. | 1.11132e-05 | 88/92 | 153 | 113.826 |
| POSITIVE\_REGULATION\_OF\_CELL\_CYCLE (c5) Genes annotated by the GO term GO:0045787. Any process that activates or increases the rate or extent of progression through the cell cycle. | 1.13043e-05 | 14/16 | 37 | 20.547 |
| module\_57 (c4) Genes in module\_57 | 1.1613e-05 | 54/56 | 98 | 66.916 |
| V$LEF1\_Q6 (c3) Genes with promoter regions [-2kb,2kb] around transcription start site containing the motif SWWCAAAGGG which matches annotation for LEF1: lymphoid enhancer-binding factor 1  TCF1: transcription factor 1, hepatic; LF-B1, hepatic nuclear factor (HNF1), albumin proximal factor | 1.16656e-05 | 174/212 | 69 | 41.961 |
| module\_275 | 1.21273e-05 | 15/16 | 18 | 7.138 |
| module\_66 (c4) Genes in module\_66 | 1.27922e-05 | 488/543 | 225 | 176.153 |
| HSA04110\_CELL\_CYCLE (c2) Genes involved in cell cycle | 1.29367e-05 | 110/112 | 173 | 130.751 |
| POD1\_KO\_MOST\_UP (c2) Most strongly up-regulated in glomeruli isolated from Pod1 knockout mice, versus wild-type controls | 1.41098e-05 | 26/28 | 30 | 14.588 |
| PATTERN\_SPECIFICATION\_PROCESS (c5) Genes annotated by the GO term GO:0007389. The developmental processes that result in the creation of defined areas or spaces within an organism to which cells respond and eventually are instructed to differentiate. | 1.49092e-05 | 27/31 | 37 | 19.269 |
| module\_308 (c4) Genes in module\_308 | 1.5037e-05 | 58/70 | 38 | 20.197 |
| V$MYCMAX\_B (c3) Genes with promoter regions [-2kb,2kb] around transcription start site containing the motif GCCAYGYGSN which matches annotation for MYC: v-myc myelocytomatosis viral oncogene homolog (avian)  MAX: MYC associated factor X | 1.55148e-05 | 160/202 | 87 | 57.085 |
| V$TCF11\_01 | 1.59154e-05 | 148/174 | 96 | 65.268 |
| MUSCLE\_CELL\_DIFFERENTIATION (c5) Genes annotated by the GO term GO:0042692. The process whereby a relatively unspecialized cell acquires specialized features of a muscle cell. | 1.60226e-05 | 21/22 | 25 | 11.414 |
| HOX\_GENES (c2) HOX genes related to hematopoiesis | 1.64138e-05 | 48/53 | 25 | 11.651 |
| V$HNF3ALPHA\_Q6 (c3) Genes with promoter regions [-2kb,2kb] around transcription start site containing the motif TRTTTGYTYWN which matches annotation for FOXA1: forkhead box A1 | 1.76178e-05 | 141/160 | 68 | 42.882 |
| TISSUE\_REMODELING (c5) Genes annotated by the GO term GO:0048771. The reorganization or renovation of existing tissues. This process can either change the characteristics of a tissue such as in blood vessel remodeling, or result in the dynamic equilibrium of a tissue such as in bone remodeling. | 1.88265e-05 | 29/30 | 33 | 16.889 |
| UVB\_NHEK3\_C4 (c2) Regulated by UV-B light in normal human epidermal keratinocytes, cluster 4 | 1.89182e-05 | 10/12 | 16 | 6.459 |
| V$SP3\_Q3 (c3) Genes with promoter regions [-2kb,2kb] around transcription start site containing the motif ASMCTTGGGSRGGG which matches annotation for SP3: Sp3 transcription factor | 2.03619e-05 | 154/187 | 89 | 59.771 |
| HSA05222\_SMALL\_CELL\_LUNG\_CANCER (c2) Genes involved in small cell lung cancer | 2.16392e-05 | 82/86 | 172 | 130.677 |
| SODIUM\_CHANNEL\_ACTIVITY (c5) Genes annotated by the GO term GO:0005272. Catalysis of facilitated diffusion of a sodium ion (by an energy-independent process) involving passage through a transmembrane aqueous pore or channel without evidence for a carrier-mediated mechanism. | 2.16632e-05 | 14/17 | 7 | 1.685 |
| ABRAHAM\_MM\_VS\_AL\_UP (c2) Genes highly expressed in multiple myeloma (MM) versus immunoglobulin light chain amyloidosis (AL) in plasma cells. | 2.19188e-05 | 18/19 | 67 | 44.036 |
| CELL\_DEVELOPMENT (c5) Genes annotated by the GO term GO:0048468. The process whose specific outcome is the progression of the cell over time, from its formation to the mature structure. Cell development does not include the steps involved in committing a cell to a specific fate. | 2.20614e-05 | 549/571 | 482 | 414.308 |
| CELL\_SOMA (c5) Genes annotated by the GO term GO:0043025. The portion of a cell bearing surface projections such as axons, dendrites, cilia, or flagella that includes the nucleus, but excludes all cell projections. | 2.3235e-05 | 9/10 | 17 | 7.06 |
| TAATTA\_V$CHX10\_01 (c3) Genes with promoter regions [-2kb,2kb] around transcription start site containing the motif TAATTA which matches annotation for VSX1: visual system homeobox 1 homolog, CHX10-like (zebrafish) | 2.41439e-05 | 485/612 | 199 | 152.031 |
| V$TTF1\_Q6 (c3) Genes with promoter regions [-2kb,2kb] around transcription start site containing the motif NNNNCAAGNRNN which matches annotation for TITF1: thyroid transcription factor 1 | 2.43439e-05 | 160/198 | 93 | 64.069 |
| NEGATIVE\_REGULATION\_OF\_CELL\_DIFFERENTIATION (c5) Genes annotated by the GO term GO:0045596. Any process that stops, prevents or reduces the frequency, rate or extent of cell differentiation. | 2.50161e-05 | 26/28 | 38 | 21.227 |
| V$PBX1\_01 (c3) Genes with promoter regions [-2kb,2kb] around transcription start site containing the motif ANCAATCAW which matches annotation for PBX1: pre-B-cell leukemia transcription factor 1 | 2.60509e-05 | 164/191 | 88 | 59.278 |
