# Supplementary material for: Looking at Cerebellar Malformations through Text-Mined Interactomes of Mice and Humans
Source: PLoS Comput Biol. 2009 Nov 6;5(11):e1000559. doi: 10.1371/journal.pcbi.1000559 (PMC2767227; doi:10.1371/journal.pcbi.1000559)
Supplement: Dataset S2 — Human network files. (5.50 MB DOC) [file pcbi.1000559.s013.zip › individual_networks/Supplementary Files Documentation.doc]

human_action.txt

Column 1: hiid – unique human action id

Column 2: upstream GeneId

Column 3: downstream GeneId

Column 4: action type

Column 5: Number of GeneWays 70 actions mapped to this human action

Column 6: Number of Action Mentions linked to the this human action

Column 7: PLO (physical, logical or other) classification of the action type

Column 8: maximum (over all the action mentions of the this human action) extraction score

Column 9: maximum (over all the action mentions of the this human action) precision level

human_actionmention.txt

Column 1: hiid – id of the human action the action mention is mapped to

Column 2: actionmentionid – internal unique identified for the action mention

Column 3: sign of the action mention (1-negative action mention; 0-positive action mention)

Column 4: the upstream of the action mention as it appears in the text

Column 5: action type

Column 6: the downstream of the action mention as it appears in the text

Column 7: PubMed Id of the article or the abstract the action mention was extracted from

Column 8: isFullText—specifies if the full text of the article was processed (Y) or only the abstract (N)

Column 9: sentence number

Column 10: action mention extraction score

Column 11: action mention precision level

human_symbols.txt

Column 1: GeneId

Column 2: the gene symbol

H70-PL.txt, H70-P0.9.txt, and H70-PL0.9.txt

Column 1: GeneId 1

Column 2: GeneId 2

Column 3: Number of human actions linked to the pair

Column 4: Number of different GeneWays action linked to the pair

Column 5: Number of action mentions linked to the pair

Column 6: list of the different action types relating the pair of genes

Column 7: list of the different action type PLO classes relating the pair of genes (P – only physical action types, L – only logical action types, and L,P both physical and logical action types)

Column 8: list of the human action ids (hiid) linked to the pair

Column 9: maximum (over all the action mentions of the this pair of genes) extraction score

Column 10: maximum (over all the action mentions of the this pair of genes) precision level
