# Supplementary material for: Looking at Cerebellar Malformations through Text-Mined Interactomes of Mice and Humans
Source: PLoS Comput Biol. 2009 Nov 6;5(11):e1000559. doi: 10.1371/journal.pcbi.1000559 (PMC2767227; doi:10.1371/journal.pcbi.1000559)
Supplement: Dataset S6 — Combined human network. (6.42 MB ZIP) [file pcbi.1000559.s017.zip › combined_network/Documentation.doc]

# The global data structures.

**g_inter**

Column 1. geneid 1

Column 2. geneid 2

Column 3. 1 if the interaction in HPRD dataset; 0 otherwise

Column 4. 1 if the interaction in human dataset; 0 otherwise

Column 5. 1 if the interaction in mouse dataset; 0 otherwise

**g_gene_names**

It’s a hash table mapping geneid to gene symbol.

Example:

g_gene_names.get(10321)

ans =

crisp3

**NOTE:** There are 30 genes with no symbols---all of the them are obsolete geneids used in the HPRD dataset.

Example:

>> g_gene_names.get(4587)

ans =

[]

**g_inter_atts_names**

**g_inter_atts**

Complete set of attributes over all datasets and all interactions.

# Functions

load_union_network()

Loads everything but is somewhat slow – 1 minute on my (Ivan’s) computer.

load_union_network_fast()

Does not load the g_inter_atts_names and g_inter_atts.

# Files

H70-PL0.9.txt	the human dataset

HMD_Human5.rpt.txt	the human mouse orthologs

HPRD_Release_7_09012007.txt	the HPRD dataset

M70-PL0.9.txt	the mouse dataset

human_mouse_HPRD_union.txt	the union network with all attributes

human_mouse_HPRD_union_small.txt	the union without attributes

human_symbols.txt	the human symbols

uu.pl	the perl script creating the union networks

# The dataset attributes

| 0 | n1 | Human geneid 1 |
| --- | --- | --- |
| 1 | n2 | Human geneid 2 |
| 2 | F_hprd | Is in hprd? |
| 3 | F_human | Is in human? |
| 4 | F_mouse | Is in mouse? |
| 5 | hprd_experiment_type |  |
| 6 | hprd_pmid |  |
| 7 | human_ats | The human pair action types. |
| 8 | human_ga_n | The human pair action number. |
| 9 | human_gam_n | The human pair action mention number. |
| 10 | human_ha_n | The human pair human actions number |
| 11 | human_hiids | The human actions ids. |
| 12 | human_m_pr | The human pair maximum action mention precision. |
| 13 | human_m_sc | The human pair maximum extraction score. |
| 14 | human_plos | The human pair physical/logical classification: “P”, “L” or “P,L” |
| 15 | mouse_ats | The mouse pair action types. |
| 16 | mouse_ga_n | The mouse pair action number. |
| 17 | mouse_gam_n | The mouse pair action mention number. |
| 18 | mouse_ha_n | The mouse pair mouse actions number. |
| 19 | mouse_hiids | The mouse action ids. |
| 20 | mouse_m_pr | The mouse pair maximum actionmention precision. |
| 21 | mouse_m_sc | The mouse pair maximum actionmention score. |
| 22 | mouse_n1 | The mouse geneid 1 (the ortholog of human geneid 1) |
| 23 | mouse_n2 | The mouse gene 2 (the ortholog of the human geneid 2) |
| 24 | mouse_plos | The mouse pair physical/logical classification: “P”, “L”, or “P,L”. |
